# Antimicrobial Activity of Chalcones with a Chlorine Atom and Their Glycosides

**DOI:** 10.3390/ijms25179718

**Published:** 2024-09-08

**Authors:** Agnieszka Krawczyk-Łebek, Barbara Żarowska, Tomasz Janeczko, Edyta Kostrzewa-Susłow

**Affiliations:** 1 Department of Food Chemistry and Biocatalysis, Faculty of Biotechnology and Food Science, Wrocław University of Environmental and Life Sciences, 50-375 Wrocław, Poland; tomasz.janeczko@upwr.edu.pl (T.J.);; 2 Department of Biotechnology and Food Microbiology, Faculty of Biotechnology and Food Science, Wrocław University of Environmental and Life Sciences, 51-630 Wrocław, Poland; barbara.zarowska@upwr.edu.pl

**Keywords:** biotransformations, chalcones with a chlorine atom, glycosylated dihydrochalcones, *Isaria fumosorosea*, *Beauveria bassiana*, antimicrobial activity

## Abstract

Chalcones, secondary plant metabolites, exhibit various biological properties. The introduction of a chlorine and a glucosyl substituent to the chalcone could enhance its bioactivity and bioavailability. Such compounds can be obtained through a combination of chemical and biotechnological methods. Therefore, 4-chloro-2′-hydroxychalcone and 5′-chloro-2′-hydroxychalcone were obtained by synthesis and then glycosylated in two filamentous fungi strains cultures, i.e., *Isaria fumosorosea* KCH J2 and *Beauveria bassiana* KCH J1.5. The main site of the glycosylation of both compounds by *I. fumosorosea* KCH J2 was C-2′ and C-3 when the second strain was utilized. The pharmacokinetics of these compounds were predicted using chemoinformatics tools. Furthermore, antimicrobial activity tests were performed. Compounds significantly inhibited the growth of the bacteria strains *Escherichia coli* 10536, *Staphylococcus aureus* DSM 799, and yeast *Candida albicans* DSM 1386. Nevertheless, the bacterial strain *Pseudomonas aeruginosa* DSM 939 exhibited significant resistance to their effects. The growth of lactic acid bacteria strain *Lactococcus acidophilus* KBiMZ 01 bacteria was moderately inhibited, but strains *Lactococcus rhamnosus* GG and *Streptococcus thermophilus* KBM-1 were completely inhibited. In summary, chalcones substituted with a chlorine demonstrated greater efficacy in inhibiting the microbial strains under examination compared to 2′-hydroxychalcone, while aglycones and their glycosides exhibited similar effectiveness.

## 1. Introduction

Chalcones, secondary plant metabolites, exhibit various biological properties, including anticancer activity (e.g., isoliquiritigenin, butein, and xantohumol), anti-inflammatory activity (e.g., sappanchalcone, butein, and licochalcone A), α-Glucosidase inhibitory activity (e.g., isobavachalcone), and antibacterial activity (e.g., bavachalcone, xanthumol, and isobavachalcone) [1]. These compounds have a shared chemical scaffold, comprising two aromatic rings linked by a three-carbon α,β-unsaturated carbonyl system. Dihydrochalcones, which are α,β-hydrogenated derivatives, are also commonly found in nature, with most famous phlorizin being ubiquitous in *Malus* species [2,3,4]. Chalcones and dihydrochalcones may occur in the form of glycosides, making them more stable and soluble in water, which improves their biodistribution and storage in plants [5,6,7]. Introducing a chlorine atom into the chalcone structure may enhance its bioactivity., including antibacterial properties [8,9,10]. When a chlorine atom is introduced onto ring B of 2′-hydroxychalcone, it enhances its antituberculosis activity against strain *Mycobacterium tuberculosis* H37Rv [9]. Similarly, another flavonoid substituted with halogen, i.e., chlorflavonin, exhibited strong antituberculosis potential with (MIC_90_ 1.56 μM) and was superior to streptomycin treatment [11]. Halogenated chalcones, while not naturally occurring, can be synthesized through the Claisen–Schmidt condensation [8,9,12].

**Figure 1:**
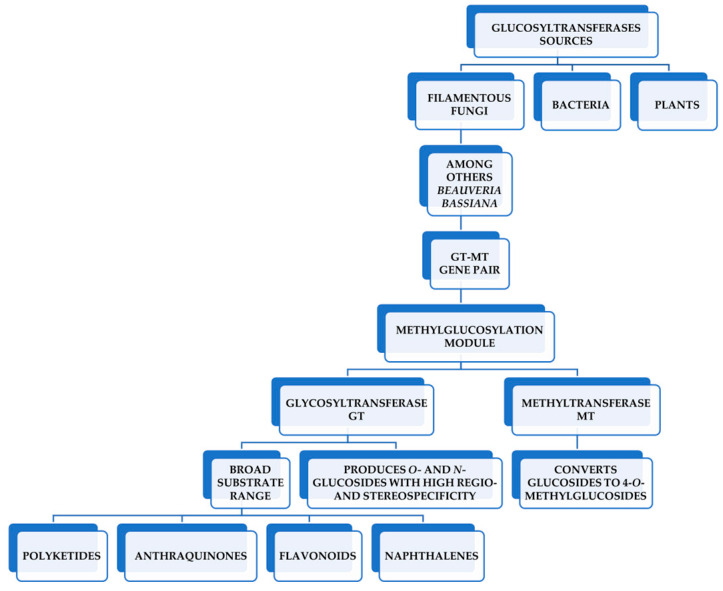
Functional modules of glycosyltransferase-methyltransferase (GT-MT) in fungi such as *B. bassiana* (developed based on Xie and coworkers [15]).

The promising strategy for the glycosylation of chalcones can be the use of microbial enzymes—among others—as well as whole-cell biotransformation using filamentous fungi as biocatalysts [13,14]. Xie and coworkers employed genome mining and heterologous expression techniques to discover functional modules of glycosyltransferase-methyltransferase (GT-MT) in these fungi. These modules exhibit substrate promiscuity and regiospecificity, allowing them to methylglucosylate flavonoids, as shown in Figure 1 [15].

The lack of in vivo data makes it difficult to generalize the influence of glycosylation on flavonoids bioactivity. Based on some reports, it appears that *O*-glycosylation may reduce anti-inflammation activity, antioxidant activity, and antimicrobial activity. Simultaneously, some data show that *O*-glycosylation can enhance specific bioactivity, including anti-HIV activity, tyrosinase inhibition, antirotavirus activity, and antiallergic activity. Furthermore, glycosylation positively affects their bioavailability [16]. The impact of glycosylation on flavonoid bioactivity in vitro may not necessarily mirror the effects observed in vivo. Consequently, additional research in this field is essential. Therefore, the purpose of this work was to obtain two chalcones with a chlorine atom, i.e., 4-chloro-2′-hydroxychalcone and 5′-chloro-2′-hydroxychalcone, and afterward glycosylate them in entomopathogenic filamentous fungi *I. fumosorosea* KCH J2 and *B. bassiana* KCH J1.5 cultures. As a result, we obtained novel flavonoid derivatives. Subsequently, we evaluated the pharmacokinetics of the received compounds using computer-aided simulations. Furthermore, the antimicrobial activities of the chalcones with chlorine atoms, their main biotransformation products, and 2′-hydroxychalcone used for comparison were assessed to explore how chlorine substitution and glucose attachment influence their effectiveness.

## 2. Results and Discussion

### 2.1. Synthesis of Biotransformation Substrates 4-Chloro-2′-Hydroxychalcone (***3***) and 5′-Chloro-2′-Hydroxychalcone (***6***)

**Figure 2:**
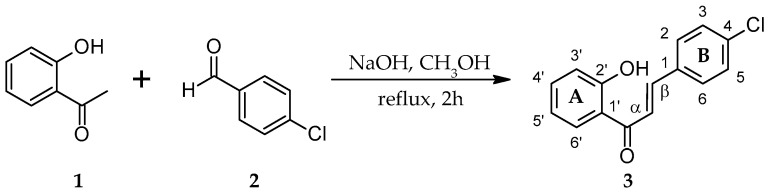
Synthesis of biotransformation substrate 4-chloro-2′-hydroxychalcone (**3**).

**Figure 3:**
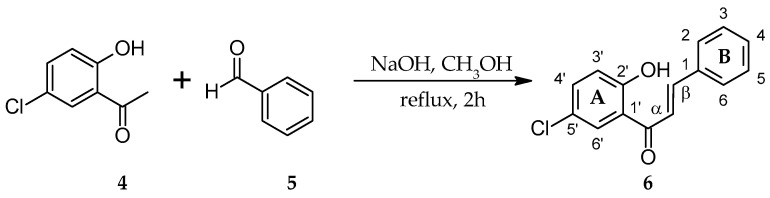
Synthesis of biotransformation substrate 5′-chloro-2′-hydroxychalcone (**6**).

The initial phase of the research was a synthesis of two chalcones with a chlorine atom 4-chloro-2′-hydroxychalcone (Figure 2) and 5′-chloro-2′-hydroxychalone (Figure 3) by Claisen–Schmidt condensation reactions.

The structures of synthetic products **3** and **6** were determined based on NMR spectroscopy (Table 1, Table 2, Table 3 and Table 4, Supplementary Information: Figures S3–S14 and S18–S29, respectively), and their molecular masses were verified using LC-MS spectroscopy (Supplementary Materials: Figures S1 and S16, respectively).

In the next step, entomopathogenic filamentous fungi *I. fumosorosea* KCH J2 and *B. bassiana* KCH J1.5 were applied to biotransform 4-chloro-2′-hydroxychalcone (**3**) and 5′-chloro-2′-hydroxychalcone (**6**) into their 4″-*O*-methylglucosylated derivatives. The biotransformation products were extracted from the reaction mixture and subsequently purified via preparative thin-layer chromatography (TLC). Product yields were calculated based on the quantities isolated. Nuclear magnetic resonance (NMR) spectroscopy was used to elucidate the chemical structures, with confirmation provided by liquid chromatography–mass spectrometry (LC-MS). The antimicrobial activity was evaluated for biotransformation substrates (**3** and **6**), their primary products (**3a**, **6a**, **6b**), together with a compound without chlorine substituent – 2′-hydroxychalcone (**7**).

### 2.2. Biotransformation of 4-Chloro-2′-Hydroxychalcone (***3***) in Culture of I. Fumosorosea KCH J2

**Figure 4:**
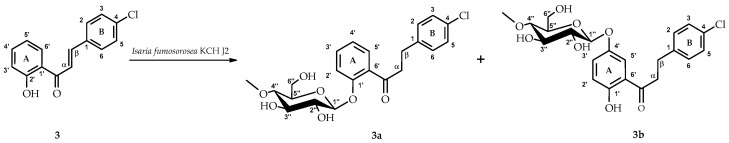
Biotransformation of 4-chloro-2′-hydroxychalcone (**3**) in *I. fumosorosea* KCH J2 culture.

The 4-Chloro-2′-hydroxychalcone (**3**) compund was biotransformed by enzymatic systems of *I. fumosorosea* KCH J2 into 4-chlorodihydrochalcone 2′-*O*-*β*-D-(4″-*O*-methyl)-glucopyranoside (**3a**), yielding 60.1% (50.7 mg), and 4-chloro-2′-hydroxydihydrochalcone 5′-*O*-*β*-D-(4″-*O*-methyl)-glucopyranoside (**3b**), yielding 4.9% (4.3 mg) (Figure 4).

**Figure 5:**
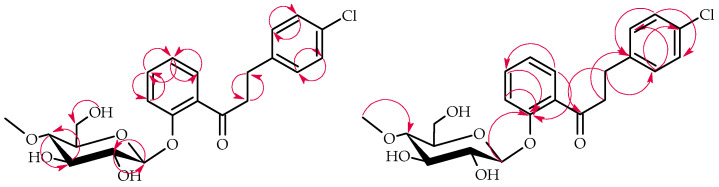
Key COSY (on the left) and HMBC (on the right) correlations of product **3a**.

**Figure 6:**
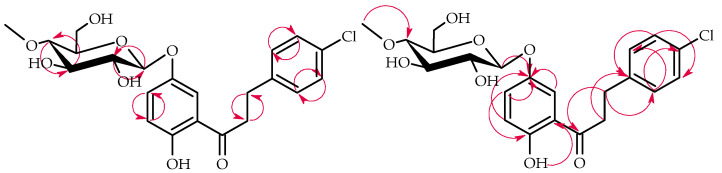
Key COSY (on the left) and HMBC (on the right) correlations of product **3b**.

The products **3a**–**3b** structures were elucidated via NMR spectroscopy (Table 1 and Table 2, Figure 5 and Figure 6 below showing key COSY and HMBC correlations) and confirmed using LC-MS (section Materials and Methods and Supplementary Materials: Figures S31 and S52). 

Five distinctive proton signals, with δH values between 3.22 and 3.84 ppm, verified the presence of a glucose moiety in biotransformation product **3a** in the ^1^H-NMR spectrum (Supplementary Materials: Figure S35), as well as five carbon signals in the region from δ = 80.0 ppm to δ = 62.0 ppm in the ^13^C-NMR spectrum (Supplementary Materials: Figure S38). In the ^1^H-NMR spectrum, a one-proton doublet from the proton at the anomeric carbon atom was present at δ = 5.08 ppm, with the coupling constant (*J* = 7.7 Hz) evidencing a *β*-configuration of the glucose (Supplementary Materials: Figure S35). The glucose molecule was also *O*-methylated at C-4″ because, in the ^1^H-NMR spectrum, a three-proton singlet at δ = 3.56 ppm, with the corresponding signal at δ = 60.6 ppm in the ^13^C-NMR spectra, was observed (Supplementary Materials: Figures S35 and S38). The correlation in the HMBC experiment between the three-proton singlet and the signal of C-4″ (δ = 80.0 ppm) proved the substitution site with the -*O*-CH_3_ group in the attached glucose group (Supplementary Materials: Figures S49). Furthermore, in product **3a**, a double bond between C-α and C-β was reduced, which shifted the protons at C-α from δ = 8.09 ppm (in **3**) to δ = 3.45 ppm (in **3a**) and at C-β from δ = 7.92 ppm (in **3**) to δ = 2.97 ppm (in **3a**) (Supplementary Materials: Figures S4 and S35). Moreover, these protons were correlated with the carbonyl group and signal of C-1 in the HMBC experiment (Supplementary Materials: Figures S47 and S50). The ^1^H-NMR spectrum of the compound **3** at δ = 12.83 ppm shows the signal from the hydroxyl group at C-2′, which was absent in the ^1^H-NMR spectrum of its microbial transformation product **3a** (Supplementary Materials: Figures S3 and S33), which indicates substitution with the 4″-*O*-methylglucopyranose. In the HMBC experiment, the shifted signal from C-2′ δ = 157.2 ppm correlated with the signal from H-1′’of the glucose moiety at δ = 5.08 ppm (Supplementary Materials: Figure S47) and also with H-6′ (δ = 7.58 ppm), H-4′ (δ = 7.48 ppm), and H-3′ (δ = 7.30 ppm (Supplementary Materials: Figure S46).

Product **3b** featured a 4″-*O*-methylglucose moiety in a *β*-configuration attached to the chalcone aglycone, alongside a reduction in the C-α and C-β double bond (Supplementary Materials: Figures S56 and S59). A shift in the ^1^H-NMR signal for the C-2′ hydroxyl group from δ = 12.83 ppm (substrate **3**) to δ = 11.89 ppm (product **3b**) indicated a 4″-*O*-methylglucosyl substitution on ring A of the chalcone (Supplementary Materials: Figures S3 and S54). The ^1^H-NMR spectrum of **3b** showed shifts in ring A proton signals compared to substrate **3**: H-6′ (δ 8.28 → 7.67 ppm), H-4′ (δ 7.58 → 7.29 ppm), and H-3′ (δ 7.00 → 6.87 ppm) (Supplementary Materials: Figures S4 and S55). The ^1^H-NMR spectrum showed the disappearance of the H-5′ signal, indicating C-5′ glucosyl substitution (Supplementary Materials: Figures S4 and S55). Furthermore, HMBC experiments revealed a correlation between H-1″ (δ 4.84 ppm) and a shifted signal at δ 150.8 ppm, which was assigned as C-5′. This C-5′ signal also correlated with H-6′ (δ 7.67 ppm), H-4′ (δ 7.29 ppm), and H-3′ (δ 6.87 ppm), confirming its assignment (Supplementary Materials: Figures S70 and S68).

### 2.3. Biotransformation of 4-Chloro-2′-Hydroxychalcone (***3***) in the Culture of B. Bassiana KCH J1.5

**Figure 7:**
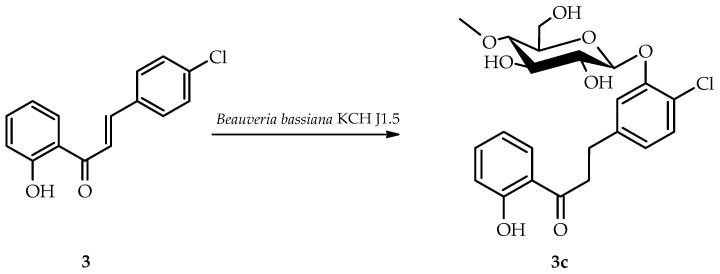
Biotransformation of 4-chloro-2′-hydroxychalcone (**3**) in *B. bassiana* KCH J1.5 culture.

*B. bassiana* KCH J1.5 biotransformed 4-chloro-2′-hydroxychalcone (**3**) less efficiently than *I. fumosorosea* KCH J2. The primary product, 4-chloro-2′-hydroxydihydrochalcone 3-*O*-*β*-D-(4″-*O*-methyl)-glucopyranoside (**3c**), was isolated, yielding 9.7% (8.5 mg) (Figure 7).

**Figure 8:**
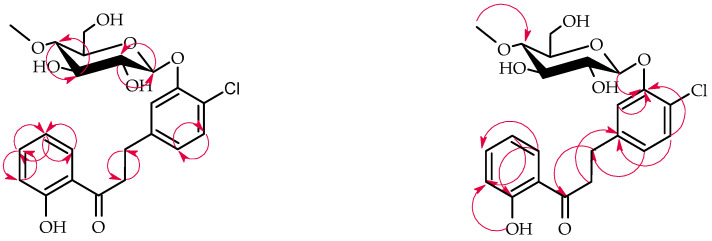
Key COSY (on the left) and HMBC (on the right) correlations of product **3c**.

NMR spectroscopy elucidated the structure of product **3c** (Table 1 and Table 2, key COSY and HMBC correlations in Figure 8). LC-MS confirmed its molecular mass (Materials and Methods, Supplementary Materials: Figure S73).

Product **3c** was identified as a dihydrochalcone due to a C-α and C-β double-bond reduction, which was evidenced by ^1^H-NMR signal shifts: H-α (δ 8.09 → 3.48 ppm) and H-β (δ 7.92 → 3.02 ppm) (Supplementary Materials: Figures S4 and S92). A 4″-*O*-methylglucopyranose substitution was confirmed by characteristic ^1^H-NMR and ^13^C-NMR signals, which were similar to products **3a** and **3b** (Supplementary Materials: Figures S77 and S80). The attachment at C-3 was evidenced by B ring proton shifts: H-2 (δ = 7.93 → 7.26 ppm), H-6 (δ = 7.93 → 6.96 ppm), H-5 (δ = 7.52 → 7.30 ppm), and H-3 signal absence (Supplementary Materials: Figures S4 and S76). HMBC correlations between H-5 (δ = 7.30 ppm), H-2 (δ = 7.26 ppm), and shifted C-3 (δ = 153.8 ppm)—plus glucose H-1″ (δ = 5.05 ppm) to C-3 correlation—further confirmed this attachment (Supplementary Materials: Figures S89 and S92).

### 2.4. Biotransformation of 5′-Chloro-2′-Hydroxychalcone (***6***) in the Culture of I. Fumosorosea KCH J2

**Figure 9:**
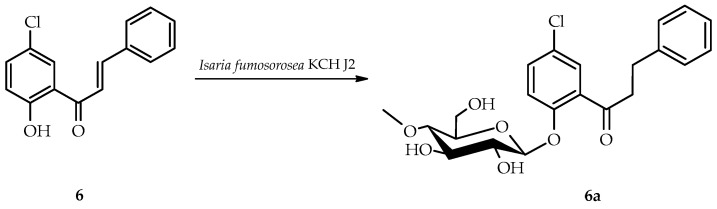
Biotransformation of 5′-chloro-2′-hydroxychalcone (**6**) in *I. fumosorosea* KCH J2 culture.

*I. fumosorosea* KCH J2 biotransformed 5′-chloro-2′-hydroxychalcone (**6**) into 5′-chlorodihydrochalcone 2′-*O*-*β*-D-(4″-*O*-methyl)-glucopyranoside (**6a**), yielding 76.1% (64.2 mg) (Figure 9).

**Figure 10:**
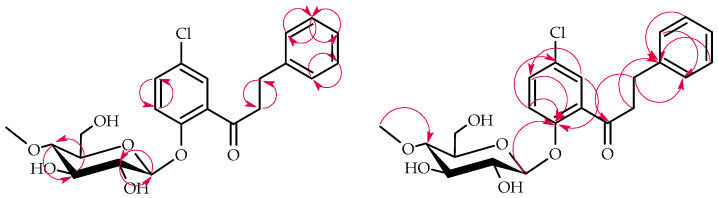
Key COSY (on the left) and HMBC (on the right) correlations of product **6a**.

The structure of product **6a** was elucidated via NMR spectroscopy (Table 3 and Table 4 in section, key COSY and HMBC correlations in Figure 10) and confirmed using LC-MS (section Materials and Methods and Supplementary Materials: Figure S94). 

Product **6a**, analogous to **3a** but with a different chlorine positioning, featured 4″-*O*-methylglucopyranose in a *β*-configuration at C-2′. This was evidenced by the disappearance of the 2′-OH signal and the presence of characteristic glucose signals in ^1^H-NMR (Supplementary Materials: Figures S96, S98 and S101). Additionally, a C-α and C-β double-bond reduction occurred, similarly to **3a**, which was indicated by characteristic shifts in the ^1^H-NMR and ^13^C-NMR spectra (Supplementary Materials: Figures S19, S21, S22, S98 and S101).

### 2.5. Biotransformation of 5′-Chloro-2′-Hydroxychalcone (***6***) in the Culture of B. Bassiana KCH J1.5

**Figure 11:**
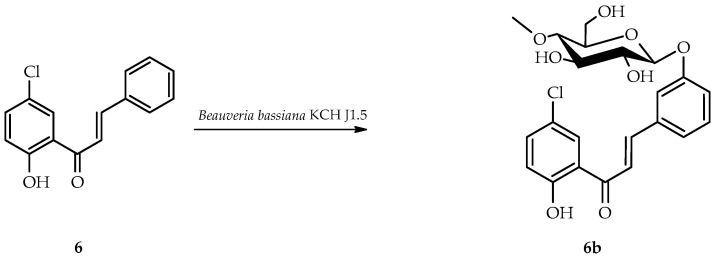
Biotransformation of 5′-chloro-2′-hydroxychalcone (**6**) in *B. bassiana* KCH J1.5 culture.

*B. bassiana* KCH J1.5 biotransformed 5′-chloro-2′-hydroxychalcone (**6**) into a single product known as 5′-chloro-2′-hydroxychlorochalcone 3-*O*-*β*-D-(4″-*O*-methyl)-glucopyranoside (**6b**), yielding 40.5% (35.3 mg) (Figure 11).

**Figure 12:**
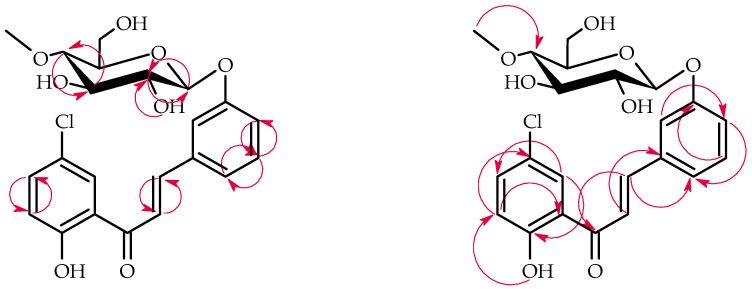
Key COSY (on the left) and HMBC (on the right) of product **6b**.

NMR spectroscopy elucidated the structure of product **6b** (Table 3 and Table 4, key COSY and HMBC correlations in Figure 12). LC-MS confirmed its molecular mass (Materials and Methods, Supplementary Materials: Figure S115).

The glycosylation of product **6b** was confirmed by characteristic 4″-*O*-methylglucosyl signals in the ^1^H-NMR and ^13^C-NMR spectra (Supplementary Materials: Figures S119 and S122). In contrast to other biotransformation products, **6b** retained its α,β-unsaturated double bond. C-3 glucosyl substitution in ring B was evidenced by shifts in proton signals: H-2 (δ 7.93 → 7.63 ppm), H-4 (δ 7.49 → 7.18 ppm), H-5 (δ 7.49 → 7.40 ppm), H-6 (δ 7.93 → 7.55 ppm), and the absence of H-3 signal in the ^1^H NMR spectrum (Supplementary Materials: Figures S19 and S76). In addition, the HMBC experiment indicated that shifted signal from C-3 (δ = 159.2 ppm) was correlated only with the H-1′’signal of the attached glucose moiety (δ = 5.04 ppm) and H-5 (δ = 7.40 ppm) (Supplementary Materials: Figure S132).

To summarize, compounds 4-chloro-2′-hydroxychalcone (**3**) and 5′-chloro-2′-hydroxychalcone (**6**) were glycosylated in cultures of both entomopathogenic fungi strains. The resulting compounds revealed different regioselectivity of the glycosyltransferase-methyltransferase functional modules of the two strains. In *I. fumosorosea* KCH J2, the primary products (**3a** and **6a**) resulted from attaching a 4″-*O*-methylglucosyl group to the C-2′ hydroxyl moiety in the A ring and reducing the C-α and C-β double bond. For 4-chloro-2′-hydroxychalcone (**3**), glycosylation also occurred at C-5′, yielding **3b**. Compound **3b** is analogous to the products obtained earlier in the cultures of this fungal strain, i.e., 2-chloro-2′-hydroxydihydrochalcone 5′-*O*-*β*-d-(4″-*O*-methyl)-glucopyranoside from 2-chloro-2′-hydroxychalcone and 3-chloro-2′-hydroxydihydrochalcone 5′-*O*-*β*-d-(4″-*O*-methyl)-glucopyranoside from 3-chloro-2′hydroxychalcone [17], and also 2′-hydroxy-4-methyldihydrochalcone 5′-*O*-*β*-D-(4″-*O*-methyl)-glucopyranoside from 2′-hydroxy-4-methylchalcone [18]. These results indicate that oxidation and subsequent glycosylation occurred in ring A when ring B was already substituted with a chlorine atom or a methyl group, and this may be related to some steric hindrance of the enzyme action. Conversely, *B. bassiana* KCH J1.5 attached the 4″-*O*-methylglucosyl moiety at C-3 in ring B of both biotransformation substrates **3** and **6** in a very similar reaction. It should also be emphasized that the introduction of the sugar unit was most likely preceded by hydroxylation of the C-3 carbon. However, substrate **3** was also hydrogenated to form **3c**, while an α,β-unsaturated double bond in **6b** remained intact. These results show that the enzyme systems of *B. bassiana* KCH J1.5 and *I. fumosrosoea* KCH J2 differ, with the former being able to introduce a 4″-O-methylglucopyranose unit, despite the presence of a substituent in the B ring. Previous studies on methylchalcones [18,19] showed less efficient glycosylation of the 2′-hydroxyl group by *I. fumosorosea* KCH J2, suggesting a positive influence of the chlorine substituent on the glycosylation site. Earlier work on chalcone glycosylation with a C-5′ methyl group in both *B. bassiana* KCH J1.5 and *I. fumosorosea* KCH J2 cultures yielded a product similar to **6b** but with a methyl group instead of chlorine [20].

Previous research on chalcone glycosylation by filamentous fungi primarily yielded 4′-*O*-*β*-D-glucopyranoside derivatives of xanthohumol. These were produced using *Penicillium chrysogenum* 6933 [21], *Absidia coerulea* AM93, and *Rhizopus nigricans* UPF701 [22]. However, certain *B. bassiana* strains (AM278 [23] and AM446 [22]) formed 4′-*O*-*β*-D-(4″-*O*-methyl)-glucopyranoside derivatives instead. The literature on chlorochalcone glycosylation is scarce. Our prior work demonstrated that *B. bassiana* KCH J1.5 could biotransform 3′-bromo-5′-chloro-2′-hydroxychalcone into 8-bromo-6-chloroflavanone 3′-*O*-*β*-D-(4″-*O*-methyl)-glucopyranoside [12]. In contrast, *I. fumosorosea* biotransformed 2′-hydroxychalcones with chlorine at C-2 or C-3, producing C-2′ glycosylated derivatives [17].

### 2.6. SwissADME Analysis: Pharmacokinetics and Drug-Likeness Prediction of 4-Chloro-2′-Hydroxychalcone (***3***), 5′-Chloro-2′-Hydroxychalcone (***6***) and Their Derivatives (***3a***–***3c***, ***6a***–***6b***)

The SwissADME online tool (http://www.swissadme.ch/), developed by the Swiss Institute of Bioinformatics’ Molecular Modeling Group (SIB) [24], was used to evaluate the pharmacokinetics, water solubility, and drug likeness of compounds **3**, **6**, their biotransformation products (**3a**–**3c**, **6a**–**6b**), and 2′-hydroxychalcone (**7**). The BOILED-Egg predictive model [25] predicted high gastrointestinal absorption for all tested molecules. Glycosylated derivatives (**3a**–**3c**, **6a**–**6b**) showed 8–18 times higher estimated aqueous solubility (ESOL method) than their aglycones (**3** and **6**). However, their estimated lipophilicity (consensus Log P_o/w_) decreased, potentially reducing their affinity for biological membranes and passive permeation in the bloodstream due to increased hydrophilicity [25]. Despite this, our previous studies on methylflavanone 4″-*O*-methylglucosides demonstrated their ability to bind in the hydrophilic region of phosphatidylcholine and erythrocyte membranes without disrupting their structure [26]. Simulations revealed that the glycosylated chalcones (**3a**–**3c**, **6a**–**6b**) lost their ability to passively permeate the blood–brain barrier and may instead have been actively transported by P-glycoprotein, unlike their aglycones (**3** and **6**). Compounds **3** and **6** may inhibit certain cytochrome P450 enzymes (CYP1A2, CYP2C9, CYP2C19) but not others (CYP2D6, CYP3A4). Conversely, their glycosylated derivatives likely do not inhibit CYP1A2, CYP2C9, and CYP2C19, but they may inhibit CYP2D6 (**3a** and **6a**) and CYP3A4 (**3a**, **3b**, **3c**, **6a**, and **6b**). All compounds passed the drug likeness (Lipinski, Ghose, Veber, Egan, and Muegge) estimators with zero violations. The Abbott bioavailability score (ABS) was 0.55 for all compounds, indicating a 55% probability of >10% bioavailability in rats or measurable Caco-2 permeability. In medicinal chemistry simulations, all compounds showed zero PAINS alerts. Detailed results are presented in Table 5.

### 2.7. Antimicrobial Effects of 2′-Hydroxychalcone and Its Derivatives (***3***, ***3a***–***3c***, ***6***, ***6a***–***6b***, and ***7***)

Antimicrobial activity tests were conducted using a Bioscreen C device (Growth Curves USA, Piscataway, NJ, USA) to evaluate the impact of chlorine atom introduction, its position, and 4′-O-methylglucopyranose attachment on 2′-hydroxychalcone activity. The following compounds were tested: 4-chloro-2′-hydroxychalcone (3), 5′-chloro-2′-hydroxychalcone (6), 4-chlorodihydrochalcone 2′-*O*-*β*-D-(4″-*O*-methyl)-glucopyranoside (**3a**), 5′-chlorodihydrochalcone 2′-*O*-*β*-D-(4″-*O*-methyl)-glucopyranoside (**6a**), 5′-chloro-2′-hydroxy-chlorochalcone 3-*O*-*β*-D-(4″-*O*-methyl)-glucopyranoside (**6b**), and 2′-hydroxychalcone (**7**) for comparison. The compounds were tested against bacteria *Escherichia coli* 10536 (Gram-), *Pseudomonas aeruginosa* DSM 939(Gram-), *Staphylococcus aureus* DSM 799 (Gram+), *Lactococcus acidophilus* KBiMZ 01 (Gram+), *Lactococcus rhamnosus* GG (Gram+), *Streptococcus thermophilus* KBM-1 (Gram+), and one strain of yeast *Candida albicans* DSM 1386. Table 6 and Table 7 present data on lag phase duration and biomass increase (ΔOD) for control, and compound-treated cultures were collected in Table 6 and Table 7.

**Figure 13:**
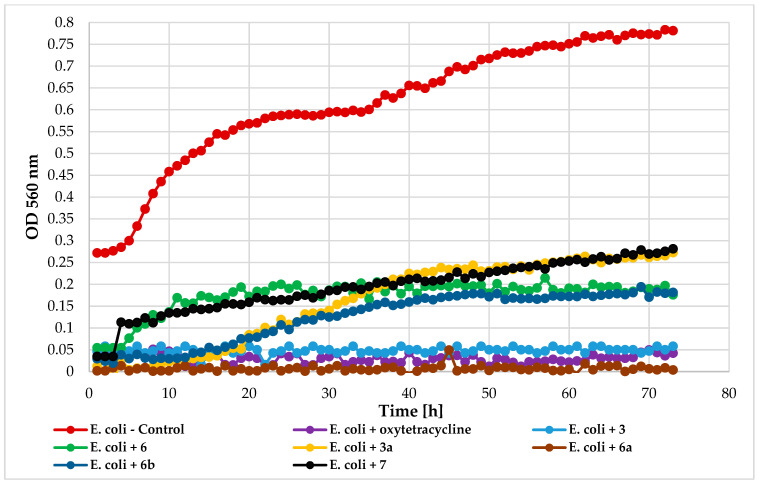
The impact of compounds **3**, **6**, **3a**, **6a**, **6b**, **7** on the growth of *E. coli* 10536.

Flavonoid aglycone **3** and glycoside **6a** exhibited the strongest inhibition against bacteria *E. coli* 10536 (ΔOD = 0). Compounds **3a** and **6b** (ΔOD = 0.15), as well as **6** (ΔOD = 0.12), also extended the microbial lag phase and significantly inhibited growth. Unsubstituted 2′-hydroxychalcone (**7**) showed slightly less effectiveness (ΔOD = 0.25). Figure 13 illustrates the *E. coli* growth in response to these compounds.

**Figure 14:**
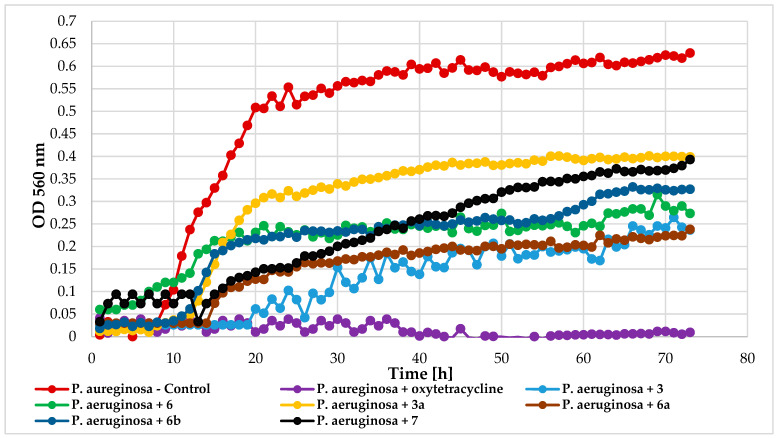
The impact of compounds **3**, **6**, **3a**, **6a**, **6b**, **7** on the growth of *P. aeruginosa* DSM 939.

All tested compounds exhibited some inhibitory effect on the relatively resistant bacteria *P. aeruginosa* DSM 939. Compounds **3**, **6**, and **6a** were most effective (ΔOD = 0.21), followed by **6b** (ΔOD = 0.31), **7** (ΔOD = 0.36), and **3a** (ΔOD = 0.39), compared to the control (ΔOD = 0.63). Figure 14 depicts *P. aeruginosa* growth in response to these compounds.

**Figure 15:**
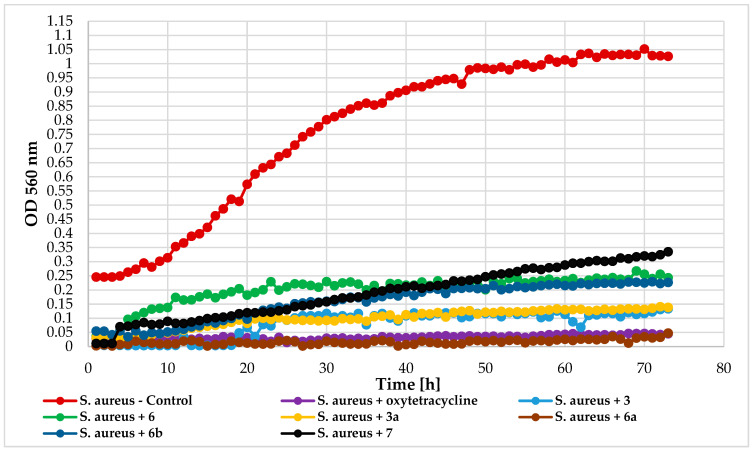
The impact of compounds **3**, **6**, **3a**, **6a**, **6b**, **7** on the growth of *S. aureus* DSM 799.

On the other hand, the bacteria growth of *S. aureus* DSM 799 was completely inhibited by compound **6a**. Other flavonoids with a chlorine atom also prolonged the microbial lag phase and significantly inhibited their growth. The least effective was the unsubstituted chlorine 2′-hydroxychalcone (ΔOD = 0.32). Figure 15 depicts the *S. aureus* growth in response to compounds **3**, **6**, **3a**, **6a**, **6b**, and **7**.

**Figure 16:**
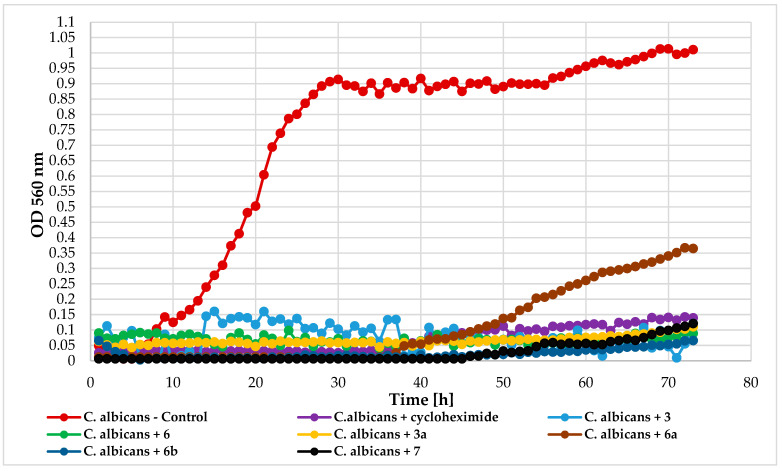
The impact of compounds **3**, **6**, **3a**, **6a**, **6b**, **7** on the growth of *C. albicans* DSM 1386.

*C. albicans* DSM 1386 yeast strain was highly sensitive to the tested compounds. Except for compounds **6a** and **7**, which allowed slight growth (ΔOD = 0.35 and ΔOD = 0.11, respectively), all others completely inhibited yeast growth. The *C. albicans* growth patterns under exposure to the tested compounds are presented in Figure 16.

**Figure 17:**
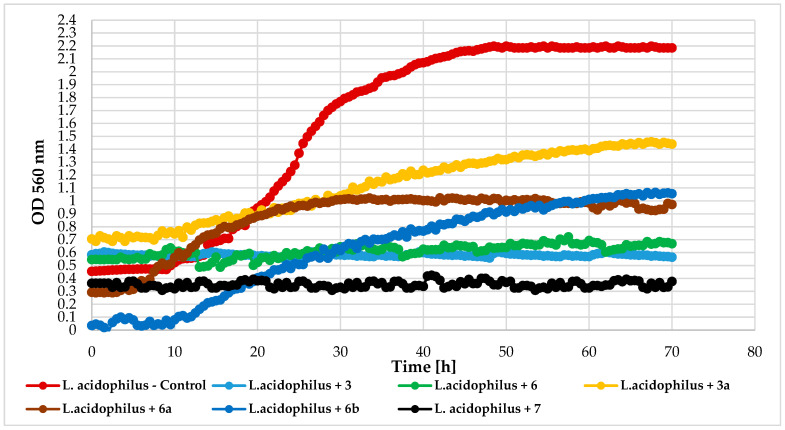
The impact of compounds **3**, **6**, **3a**, **6a**, **6b**, **7** on the growth of *L. acidophilus* KBiMZ 01.

**Figure 18:**
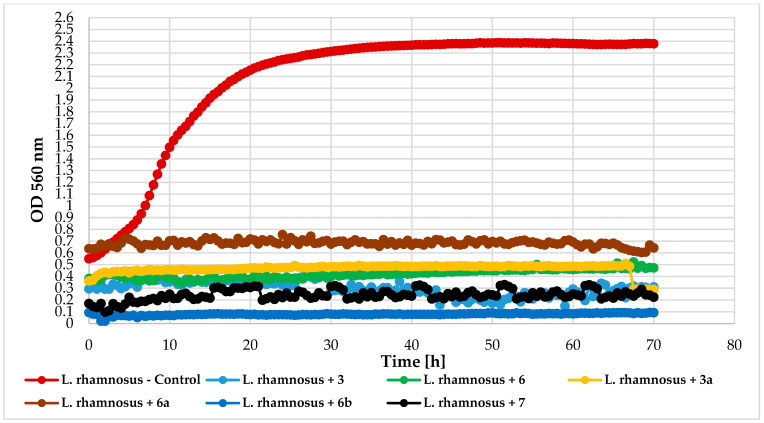
The impact of compounds **3**, **6**, **3a**, **6a**, **6b**, **7** on the growth of *L. rhamnosus* GG.

**Figure 19:**
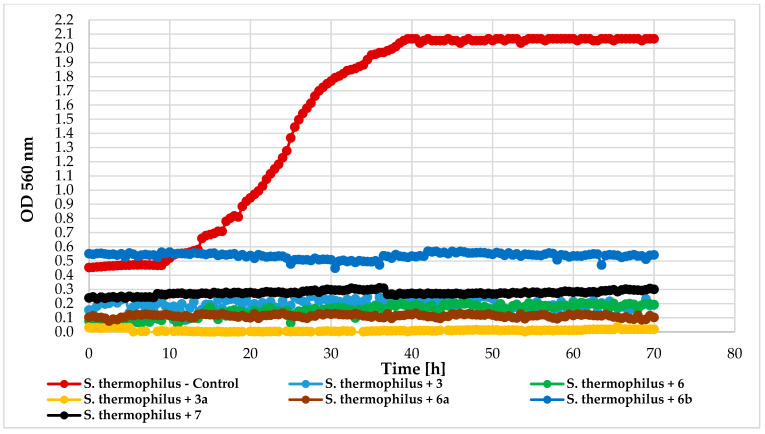
The impact of compounds **3**, **6**, **3a**, **6a**, **6b**, **7** on the growth of *S. thermophilus* KBM-1.

The effect of the tested compounds on the lactic acid bacteria varied by species. *L. rhamnosus* GG and *S. thermophilus* KBM-1 growth were totally inhibited by all compounds. However, complete growth inhibition of the *L. acidophilus* KBiMZ 01 bacteria occurred because of the action of compounds **3** and **7**, and significant inhibition occurred when molecule **6** was used (ΔOD = 0.12). The glycosylated flavonoids with a chlorine atom (**3a**, **6a**, and **6b**) prolonged the microbial lag phase and limited this lactic bacteria growth. The lowest level of inhibition was observed in the case of compound **6b**. Figure 17, Figure 18 and Figure 19 detail the growth of these bacteria in response to the compounds used.

Antimicrobial activity tests of the obtained compounds **3**, **6**, **3a**, **6a**, **6b**, and **7** showed that the substitution of a chlorine atom in the chalcone structure had a positive effect on their activity against the tested microorganisms: *E. coli* 10536, *S. aureus* DSM 799, *P. aeruginosa* DSM 939, and *C. albicans* DSM 1386. Prasad and coworkers also showed a positive effect of pharmacophores like chloro-, dichloro-, and fluoro-moiety on antibacterial activity against the used strain of *E. coli* bacteria [8]. On the other hand, antimicrobial activity tests against the *S. aureus* AM-176 strain performed by Alcaraz and coworkers showed that 4-chlorochalcone inhibited its growth less effectively than 2′-hydroxychalcone and chalcone (PID (Percent Inhibition Degree) = 34.7, 98.3, and 38.3 respectively) [27]. These results indicate that the hydroxyl group within the chalcone structure strongly influences its antimicrobial activity. However, researchers have not investigated how the combined effects of a 2′-hydroxyl moiety and a chlorine atom impact the antimicrobial properties of the resulting compound. In the presented work, chlorinated 2′-hydroxychalcones showed significantly stronger antibacterial activity against *S. aureus* DSM 799. Furthermore, their derivatives with the blocked 2′-hydroxyl group by the attached glucosyl moiety were not less effective. Konečná and coworkers also proved that introducing a bromine or chlorine atom into pyrazine-based chalcones yielded receiving compounds with strong antistaphylococcal and antienterococcal activity [10]. The impact of glucosyl groups on chalcones’ antimicrobial properties remains poorly understood. However, in the antimicrobial activity tests of another group of flavonoids with this moiety, i.e., flavonol 3-*O*-glycosides, researchers observed a potent suppression of Gram-positive bacteria and a weaker inhibition of Gram-negative bacteria [16]. In our studies, *E. coli* 10536 were exceptionally susceptible to the actions of chlorinated aglycones and the glycosides of 2′-hydroxychalcone. However, *P. aeruginosa* DSM 939 was quite opposing. In our previous studies with 2-chloro-2′-hydroxychalcone, 3-chloro-2′hydroxychalcones, and their glycosides, we also observed that chlorinated chalcones were more active as inhibitors of the tested microbial strains’ growth compared to their unchlorinated counterparts. However, aglycones showed slightly greater efficacy than their glycoside forms [17]. By comparing all the obtained results, one can observe differences in the inhibition of microbial growth depending on the chlorine atom substitution position in the tested chalcones. Comparing all of the results obtained, differences in microbial growth inhibition can be observed depending on a chlorine atom substitution position in chalcones tested. Interestingly, 5′-chlorodihydrochalcone 2′-*O*-*β*-D-(4‴-*O*-methyl)-glucopyranoside (**6a**) showed significant activity against all tested microbial strains but was the least effective against the *C. albicans* DSM 1386 strain.

## 3. Materials and Methods

### 3.1. General Procedure for the Synthesis of Biotransformation Substrates ***3*** and ***6***

An amount of 2′-Hydroxychalcones with a chlorine atom, i.e., 4-chloro-2′-hydroxychalcone (**3**) and 5′-chloro-2′-hydroxychalcone (**6**), were received in the Claisen–Schmidt condensation reaction in alkaline conditions, as previously described [17,28,29,30,31,32]. The Results and Discussion section above presents the chemical reaction schemes in Figures S1 and S2.

The physical data of compounds **3** and **6** (color, form, molecular ion mass, molecular formula, melting point (°C), retention time t_R_ (min), retardation factor Rf, and NMR spectral data) are presented below, in Table 1, Table 2, Table 3 and Table 4 in the Results and Discussion section, and in the Supplementary Materials.

The **4-Chloro-2′-hydroxychalcone** (**3**)**:** Yellow crystals (73.6%, 7.6 g); ESI/MS *m*/*z* 259.0 ([M + H]^+^, C_15_H_11_ClO_2_); mp = 147–149 °C; t_R_ = 18.58; Rf = 0.93; ^1^H-NMR, see Table 1, ^13^C-NMR, see Table 2, Supplementary Materials: Figures S3–S14.

The **5′-Chloro-2′-hydroxychalcone** (**6**)**:** Yellow crystals (90.5%, 9.4 g), ESI/MS *m*/*z* 259.0 ([M + H]^+^, C_15_H_11_ClO_2_), mp = 106–108 °C, t_R_ = 18.71, Rf = 0.93, ^1^H-NMR, see Table 3, ^13^C-NMR, see Table 4, Supplementary Materials: Figures S18–S29.

### 3.2. Microorganisms

Microbial glycosylation of chlorochalcones **3** and **6**, obtained by chemical synthesis, was achieved in cultures of *I. fumosorosea* KCH J2 and *B. bassiana* KCH J1.5 filamentous fungi belonging to the collection of the Faculty of Biotechnology and Food Microbiology of the Wrocław University of Environmental and Life Sciences in Poland. Our previous studies detailed the genetic identification, collection methods, and reproduction of these fungi [13,33].

### 3.3. Analysis

Thin-layer chromatography (TLC) and high-performance liquid chromatography (HPLC) were used to monitor biotransformation progress, specifically substrate transformation [18]. All compounds were 95%-98% pure according to HPLC analysis. The separation of biotransformation products on a semi-preparative scale was achieved using preparative silica gel TLC plates with thicknesses of 500 µm and 1000 µm (Supelco, Darmstadt, Germany) and mixture of chloroform and methanol (9:1 volume ratio) [17,19].

NMR analyses (^1^H-NMR, ^13^C-NMR, COSY, HMQC, and HMBC) were carried out using a DRX Avance^TM^ 600 MHz NMR spectrometer (Bruker, Billerica, MA, USA). All samples were dissolved in deuterated acetone for analysis.

The molecular formulas of all products (**3**, **3a**, **3b**, **3c**, **6**, **6a**, and **6b**) were confirmed through mass spectrometry analyses using a LC-MS 8045 SHIMADZU Triple Quadrupole Liquid Chromatograph Mass Spectrometer with electrospray ionization (ESI) source (Shimadzu, Kyoto, Japan), as described in our previous works [17,19].

### 3.4. Screening Procedure

A biotransformation screening procedure was conducted to determine the time required for the complete conversion of substrates **3** and **6** in preparation for subsequent experiments at a semi-preparative scale. Entomopathogenic fungi were cultivated using a modified Sabouraud medium. Fungal strains were initially grown for 72 h and then transferred to fresh medium. Substrates **3** or **6** (10 mg) were added to flasks containing either *Isaria fumosorosea* KCH J2 or *Beauveria bassiana* KCH J1.5, with a final concentration of 0.39 mM. Samples were collected after 3, 6, and 8 days. Biotransformation products were extracted with ethyl acetate, which were then dried and concentrated. The experiments were concluded after 8 days or when complete substrate conversion was confirmed. Controls included substrate stability and cultivation without substrates [17,19].

### 3.5. The Semi-Preparative Biotransformation

Semi-preparative biotransformation was conducted in 2 L flasks with 500 mL of modified Sabouraud medium to produce sufficient product for NMR analyses, structural determination, and antimicrobial testing. The process began with transferring 1 mL of a preincubated culture of *I. fumosorosea* KCH J2 or *B. bassiana* KCH J1.5 to the flasks, followed by a 72-h incubation. Then, 50 mg of substrate **3** or **6** (dissolved in 2.0 mL of dimethyl sulfoxide) was added, maintaining a final concentration of 0.39 mM. The flasks were incubated on a rotary shaker for 8 days. Post-reaction mixtures were extracted with ethyl acetate, which were then dried, filtered, and evaporated. The biotransformation products were separated and purified using preparative TLC plates, which were then visualized under UV light and extracted with ethyl acetate. The chemical structures were analyzed via spectroscopic methods, and yields were determined based on the mass of the isolated products [17,19].

### 3.6. Fungal Biotransformation Products

The physical data of compounds **3a**–**3c** and **6a**–**6b** (color, form, molecular ion mass, molecular formula, melting point (°C), retention time t_R_ (min), retardation factor Rf, and NMR spectral data) are presented below, in Table 1, Table 2, Table 3 and Table 4 in the Results and Discussion section, and in the Supplementary Materials.

The 4-Chlorodihydrochalcone 2′-*O*-*β*-D-(4‴-*O*-methyl)-glucopyranoside (**3a**): white crystals; ESI/MS *m*/*z* 435.1 ([M − H]^−^, C_22_H_25_ClO_7_); mp = 132–134 °C; t_R_ = 11.94, Rf = 0.39; ^1^H-NMR, see Table 1, ^13^C-NMR, see Table 2, Supplementary Information: Figures S31–S50.

The 4-Chloro-2′-hydroxydihydrochalcone 5′-*O*-*β*-D-(4‴-*O*-methyl)-glucopyranoside (**3b**): white crystals; ESI/MS *m*/*z* 451.1 ([M − H]^−^, C_22_H_25_ClO_8_); mp = 133–135 °C; t_R_ = 13.31, Rf = 0.33; ^1^H-NMR, see Table 1, ^13^C-NMR, see Table 2, Supplementary Information: Figures S52–S71.

The 4-Chloro-2′-hydroxydihydrochalcone 3-*O*-*β*-D-(4‴-*O*-methyl)-glucopyranoside (**3c**): light-yellow crystals; ESI/MS *m*/*z* 451.1 ([M − H]^−^, C_22_H_25_ClO_8_); mp = 89–91 °C; t_R_ = 11.24, Rf = 0.42; ^1^H-NMR, see Table 1, ^13^C-NMR, see Table 2, Supplementary Information: Figures S73–S92.

The 5′-Chlorodihydrochalcone 2′-*O*-*β*-D-(4‴-*O*-methyl)-glucopyranoside (**6a**): white crystals; ESI/MS *m*/*z* 435.1 ([M − H]^−^, C_22_H_25_ClO_7_); mp = 154–156 °C; t_R_ = 12.79, Rf = 0.26; ^1^H-NMR, see Table 3, ^13^C-NMR, see Table 4, Supplementary Information: Figures S94–S113.

The 5′-Chloro-2′-hydroxy-chlorochalcone 3-*O*-*β*-D-(4‴-*O*-methyl)-glucopyranoside (**6b**): light-yellow crystals; ESI/MS *m*/*z* 451.1 ([M + H]^+^, C_22_H_23_ClO_8_); mp = 207–209 °C; t_R_ = 13.47, Rf = 0.23; ^1^H-NMR, see Table 3, ^13^C-NMR, see Table 4, Supplementary Information: Figures S115–S133.

### 3.7. Pharmacokinetics and Drug Nature Predictions

The pharmacokinetic predictions, physicochemical features, and drug likeness of chalcone derivatives **3**, **3a**-**3c**, **6**, **6a**, **6b**, and **7** were computed using SwissADME (available online: http://www.swissadme.ch, accessed on 12 January and 14 March 2024). The molecular structures were constructed using ACD Chemsketch 2021.2.0 and then imported into this SwissADME online tool [18]. The detailed prediction results are presented in the Supplementary Materials: Figures S15 (**3**), S30 (**6**), S51 (**3a**), S72 (**3b**), S93 (**3c**), S114 (**6a**), S134 (**6b**), S135 (**7**).

### 3.8. Antimicrobial Activity Assays

Antimicrobial activity tests of compounds **3**, **3a**, **6**, **6a, 6b**, and **7** (Sigma-Aldrich, Sant Louis, MO, USA) were conducted using a Bioscreen C (Automated Microbiology Growth Curve Analysis System, Helsinki, Finland) against the following strains of bacteria: *E. coli* 10536 (Gram-negative), *P. aeruginosa* DSM 939 (Gram-negative), *S. aureus* DSM 799 (Gram-positive), Gram-positive lactic bacteria *S. thermophilus* KBM-1, *L. acidophilus* KBiMZ 01, *L. rhamnosus* GG, and yeast strain *C. albicans* DSM 1386 belonging to the collection of the Faculty of Biotechnology and Food Microbiology of Wrocław University of Environmental and Life Sciences. The microbiological cultures were prepared 48 h prior to the assays in media purchased from Merck, Darmstadt, Germany. Bacteria *E. coli* 10536, *P. aeruginosa* DSM 939, and *S. aureus* DSM 799 were cultured in LB broth. Yeast *C. albicans* DSM 1386 was grown in YPG medium. Bacterium *S. thermophilus* KBM-1 was cultured in M17 medium. Additionally, *L. acidophilus* KBiMZ 01 and *L. rhamnosus* GG were grown in MRS medium [17,34,35,36,37,38].

Assays were conducted using 100-well microtiter plates with a working volume of 300 µL per well. Each well contained 280 µL of culture medium, 10 µL of microorganism suspension (final density 1 × 10^6^ cells/mL), and 10 µL of flavonoids dissolved in dimethyl sulfoxide (final flavonoid concentration 0.1% (*m*/*v*)). The dimethyl sulfoxide final concentration in each well was 3.3% (*v*/*v*). The plates were incubated at 30 °C with optical density measurements at 560 nm taken every 60 min for 72 h for all microorganisms except lactic acid bacteria, for which the temperature was 37 °C, and measurements were taken every 30 min for 70 h. Each test was performed in triplicate with continuous shaking. Oxytetracycline (10 mg/mL) and cycloheximide (0.1% (*m*/*v*) purchased from Sigma-Aldrich (Saint Louis, MO, USA) were used as positive controls. Data were analyzed using Microsoft Excel, and growth curves were created based on the mean absorbance values over time. Antimicrobial activity was assessed by comparing the increase in optical density (ΔOD) of treated cultures to controls with only dimethyl sulfoxide [17].

## 4. Conclusions

In this study, we showed the ability of fungi strains *I. fumosorosea* KCH J2 and *B. bassiana* KCH J1.5 to yield 4-*O*-methylglucosyl derivatives of 4-chloro-2′-hydroxychalcone (**3**) and 5′-chloro-2′-hydroxychalcone (**6**). The main biotransformation product of **3** in cultures of *I. fumosorosea* KCH J2-4-chlorodihydrochalcone 2′-*O*-*β*-D-(4‴-*O*-methyl)-glucopyranoside (**3a**) was obtained with a good isolated yield of 60.1%. Similarly, enzymatic systems of this strain very effectively biotransformed 5′-chloro-2′-hydroxychalcone into only one product-5′-chlorodihydrochalcone 2′-*O*-*β*-D-(4‴-*O*-methyl)-glucopyranoside (**6a**), yielding 76.1%. This biotransformation substrate was also effectively biotransformed by *B. bassiana* KCH J1.5 into 5′-chloro-2′-hydroxy-chlorochalcone 3-*O*-*β*-D-(4‴-*O*-methyl)-glucopyranoside (**6b**), with a yield of 40.5%. All the resulting compounds have not been described in the literature until now. The techniques outlined in this study enable the efficient and cost-effective synthesis of substantial quantities of glycoside derivatives of chalcones containing a chlorine atom. These derivatives can then be investigated further for their biological activity and bioavailability. Simulation results performed in the SwissADME online tool showed changes in the pharmacokinetics and water solubility of glycosylated flavonoids concerning their aglycones. On the other hand, antimicrobial activity tests showed that the introduction of a chlorine atom and glucosyl moiety into the structure of 2′-hydroxychalcone affects its bioactivity. All chlorinated chalcones were more effective in inhibiting the tested microbial strains than 2′-hydroxychalcone. In contrast, chalcone aglycones with a chlorine atom and their glycosides were similarly effective. The highest antibacterial potential against the tested strain was demonstrated by 5′-chlorodihydrochalcone 2′-*O*-*β*-D-(4‴-*O*-methyl)-glucopyranoside. Further experiments are required to elucidate their mechanisms of action.

## Figures and Tables

**Table 1:** The ^1^H-NMR chemical shifts δ (ppm) and coupling constants *J* (Hz) of 4-chloro-2′-hydroxychalcone (**3**) and their biotransformation products **3a–3c** in Acetone-d6, 600 MHz (Supplementary Materials: Figures S3, S4, S33–S35, S54–S56 and S75–S77).

Proton	Compound			
	3	3a	3b	3c
H-α	8.09 (d) *J* = 15.5	3.45 (m)	3.48 (t) *J* = 7.3	3.48 (m)
H-β	7.92 (d) *J* = 15.5	2.97 (m)	3.04 (t) *J* = 7.4	3.02 (t) *J* = 7.6
H-2	7.93 (m)	7.30 (m)	7.36 (m)	7.26 (d) *J* = 1.8
H-3	7.52 (m)	7.30 (m)	7.31 (m)	-
H-5	7.52 (m)	7.30 (m)	7.31 (m)	7.30 (d) *J* =8.1
H-6	7.93 (m)	7.30 (m)	7.36 (m)	6.96 (m)
H-3′	7.00 (m)	7.30 (m)	6.87 (d) *J* = 9.0	6.96 (m)
H-4′	7.58 (m)	7.48 (ddd) *J* = 9.0, *J* = 7.3, *J* = 1.8	7.29 (dd) *J* = 9.0, *J* = 2.8	7.53 (m)
H-5′	7.00 (m)	7.10 (td) *J* = 7.7, *J* = 0.9	-	6.96 (m)
H-6′	8.28 (dd) *J* = 8.3, *J* = 1.4	7.58 (dd) *J* = 7.7, *J* = 1.6	7.67 (d) *J* = 2.9	8.00 (dd) *J* = 8.4, *J* = 1.6
H-1″	-	5.08 (d) *J* = 7.7	4.84 (d) *J* = 7.8	5.05 (d) *J* = 7.6
H-2″	-	3.51 (m)	3.43 (m)	3.52 (m)
H-3″	-	3.63 (dd) *J* = 8.9, *J* = 3.7	3.59 (m)	3.61 (m)
H-4″	-	3.22 (m)	3.13 (dd) *J* = 9.6, *J* = 9.0	3.20 (dd) *J* = 9.6, *J* = 8.9
H-5″	-	3.51 (m)	3.43 (m)	3.48 (m)
H-6″	-	3.84 (m) 3.69 (m)	3.85 (m) 3.85 (m)	3.82 (m) 3.67 (m)
4″-OCH_3_	-	3.56 (s)	3.54 (s)	3.55 (s)
C2′-OH	12.83 (s)	-	11.89 (s)	12.24 (s)
2″-OH	-	4.68 (d) *J* = 3.4	4.64 (d) *J* = 4.0	4.61 (d) *J* = 4.3
3″-OH	-	4.52 (d) *J* = 4.0	4.42 (d) *J* = 4.2	4.46 (d) *J* = 4.1
6″-OH	-	3.78 (m)	3.67 (m)	3.82 (m)

**Table 2:** The ^13^C-NMR chemical shifts δ (ppm) and coupling constants *J* (Hz) of 4-chloro-2′-hydroxychalcone (**3**) and their biotransformation products **3a–3c** in Acetone-d6, 151 MHz (Supplementary Materials: Figures S5–S7, S36–S38, S57–S59 and S78–S80).

Carbon	Compound			
	3	3a	3b	3c
C-α	122.3	45.4	40.0	40.2
C-β	144.6	30.2	29.5	30.1
C-1	134.6	141.7	141.0	142.5
C-2	131.5	131.1	131.2	117.4
C-3	130.0	129.1	129.2	153.8
C-4	137.1	131.8	130.6	121.0
C-5	130.0	129.1	129.2	130.6
C-6	131.5	131.1	131.2	123.7
C-1′	120.8	130.4	119.8	120.2
C-2′	164.5	157.2	158.4	163.1
C-3′	119.0	117.1	119.4	118.8
C-4′	137.6	134.1	127.6	137.3
C-5′	119.9	123.0	150.8	119.3
C-6′	131.5	130.4	118.1	131.6
C-1″	-	102.1	102.8	101.3
C-2″	-	75.0	74.9	74.8
C-3″	-	78.1	78.0	78.2
C-4″	-	80.0	80.4	80.1
C-5″	-	77.2	77.2	77.1
C-6″	-	62.0	62.3	62.1
4″-OCH_3_	-	60.6	60.6	60.6
C=O	194.9	201.9	206.4	206.7

**Table 3:** The ^1^H-NMR chemical shifts δ (ppm) and coupling constants *J* (Hz) of 5′-chloro-2′-hydroxychalcone (**6**) and their biotransformation products **6a, 6b** in Acetone-d6, 600 MHz (Supplementary Materials: Figures S18, S19, S96–S98 and S117–S119).

Proton	Compound		
	6	6a	6b
H-α	8.12 (d) *J* = 15.4	3.44 (t) *J* = 7.5	8.07 (d) *J* = 15.4
H-β	7.98 (d) *J* = 15.4	2.98 (t) *J* = 7.5	7.93 (d) *J* = 15.5
H-2	7.93 (m)	7.26 (m)	7.63 (m)
H-3	7.49 (m)	7.26 (m)	-
H-4	7.49 (m)	7.16 (m)	7.18 (ddd) *J* = 8.2, *J* = 2.4, *J* = 0.9
H-5	7.49 (m)	7.26 (m)	7.40 (t) *J* = 7.9
H-6	7.93 (m)	7.26 (m)	7.55 (d) *J* = 7.7
H-3′	7.03 (d) *J* = 8.9	7.34 (d) *J* = 8.8,	7.03 (d) *J* = 8.9
H-4′	7.57 (dd) *J* = 8.9, *J* = 2.6	7.48 (dd) *J* = 8.8, *J* = 2.8	7.58 (dd) *J* = 8.9, *J* = 2.6,
H-6′	8.32 (d) *J* = 2.6	7.51 (d) *J* = 2.7	8.35 (d) *J* = 2.6
H-1″	-	5.09 (d) *J* = 7.7	5.04 (d) *J* = 7.8
H-2″	-	3.51 (m)	3.49 (m)
H-3″	-	3.63 (m)	3.61 (m)
H-4″	-	3.21 (m)	3.21 (dd) *J* = 9.7, *J* = 8.9
H-5″	-	3.51 (m)	3.55 (m)
H-6″	-	3.83 (m) 3.68 (m)	3.90 (m) 3.90 (m)
4″-OCH_3_	-	3.55 (s)	3.57 (s)
C2′-OH	12.85 (s)	-	12.82 (s)
2″-OH	-	4.68 (d) *J* = 4.3	4.71 (d) *J* = 3.8
3″-OH	-	4.54 (d) *J* = 4.4	4.49 (d) *J* = 3.7
6″-OH	-	3.78 (m)	3.72 (m)

**Table 4:** The ^13^C-NMR chemical shifts δ (ppm) of 5′-chloro-2′-hydroxychalcone (**6**) and their biotransformation products **6a, 6b** in Acetone-d6, 151 MHz (Supplementary Materials: Figures S20–S22, S99–S101 and S12–S122).

Carbon	Compound		
	6	6a	6b
C-α	121.1	45.6	121.5
C-β	147.2	30.7	146.9
C-1	135.6	142.4	136.9
C-2	130.2	129.3	117.3
C-3	129.9	129.1	159.2
C-4	132.1	126.7	120.3
C-5	129.9	129.1	130.8
C-6	130.2	129.3	124.3
C-1′	124.2	131.9	124.2
C-2′	163.1	155.8	163.0
C-3′	120.9	119.1	120.8
C-4′	137.1	133.4	137.2
C-5′	121.7	127.7	121.6
C-6′	130.4	129.7	130.6
C-1″	-	102.2	101.5
C-2″	-	74.9	74.9
C-3″	-	78.0	78.2
C-4″	-	80.0	80.3
C-5″	-	77.3	77.1
C-6″	-	62.0	62.2
4″-OCH_3_	-	60.6	60.6
C=O	194.3	200.9	194.3

**Table 5:** SwissADME online tool analysis of pharmacokinetic properties and drug-likeness for compounds **3**, **3a**–**3c**, **6**, **6a**–**6b**, and **7**.

Activity/Parameter	3	3a	3b	3c	6	6a	6b	7
**Lipophilicity consensus Log Po/w**	3.70	2.14	1.97	1.89	3.70	1.95	1.78	3.13
**Water solubility [mg/mL]**	0.0068	0.125	0.0786	0.0786	0.0068	0.125	0.0525	0.0221
**Gastrointestinal absorption**	High	High	High	High	High	High	High	High
**BBB permeant**	Yes	No	No	No	Yes	No	No	Yes
**P-gp substrate**	No	Yes	Yes	Yes	No	Yes	Yes	No
**CYP1A2 inhibitor**	Yes	No	No	No	Yes	No	No	No
**CYP2C9 inhibitor**	Yes	No	No	No	Yes	No	No	Yes
**CYP2C19 inhibitor**	Yes	No	No	No	Yes	No	No	Yes
**CYP2D6 inhibitor**	No	Yes	No	No	No	Yes	No	No
**CYP3A4 inhibitor**	No	Yes	Yes	Yes	No	Yes	Yes	No
**Log Kp (skin permeation) [cm/s]**	−4.68	−7.58	−7.54	−7.54	−4.68	−7.58	−7.39	−4.91
**Drug-likeness (Lipinski, Ghose, Veber, Egan, and Muegge)**	Yes	Yes	Yes	Yes	Yes	Yes	Yes	Yes
**Abbott bioavailability score (ABS)**	0.55	0.55	0.55	0.55	0.55	0.55	0.55	0.55
**PAINS**	0 alert	0 alert	0 alert	0 alert	0 alert	0 alert	0 alert	0 alert

**Table 6:** Antimicrobial activity of compounds **3**, **6**, **3a**, **6a**, **6b**, **7** against *E. coli* 10536, *S. aureus* DSM 799, *P. aeruginosa* DSM 939, *C. albicans* DSM 1386.

Compounds and Standard Drugs	*E. coli* 10536 (Gram-)		*P. aeruginosa* DSM 939 (Gram-)		*S. aureus* DSM 799 (Gram+)		*C. albicans* DSM 1386 (Yeast)	
	Lag-Phase [h]	ΔOD	Lag-Phase [h]	ΔOD	Lag-Phase [h]	ΔOD	Lag-Phase [h]	ΔOD
**Control**	1.5	0.51	6.0	0.63	2.0	0.78	10	0.96
**Oxytetracycline**	-	0	-	0	-	0	-	-
**Cycloheximide**	-	-	-	-	-	-	-	0
**3**	-	0	18.0	0.21	16.0	0.13	-	0
**3a**	7.0	0.15	8.0	0.39	4.0	0.11	-	0
**6**	4.0	0.12	3.0	0.21	4.0	0.21	-	0
**6a**	-	0	14.0	0.21	-	0	-	0.35
**6b**	12.0	0.15	6.0	0.31	7.0	0.17	-	0
**7**	5.0	0.25	9.0	0.36	10	0.32	-	0.11

**Table 7:** Antimicrobial activity **3**, **6**, **3a**, **6a**, **6b**, **7** against lactic acid bacteria strains *L. acidophilus* KBiMZ 01, *L. rhamnosus* GG, *S. thermophilus* KBM-1.

Compounds	*L. acidophilus* KBiMZ 01 (Gram+)		*L. rhamnosus* GG (Gram+)		*S. thermophilus* KBM-1 (Gram+)	
	Lag-Phase [h]	ΔOD	Lag-Phase [h]	ΔOD	Lag-Phase [h]	ΔOD
**Control**	8.0	1.73	2.0	1.83	6.0	1.61
**3**	-	0	-	0	-	0
**3a**	7	0.74	-	0	-	0
**6**	-	0.12	-	0	-	0.12
**6a**	5	0.68	-	0	-	0
**6b**	8	1.02	-	0	-	0
**7**	-	0	-	0	-	0

## Data Availability

The original data presented in the study are included in the article and Supplementary Materials, and they are also openly available in the Wrocław University of Environmental and Life Sciences Repository at https://doi.org/10.57755/8dms-8k94 (accessed on 2 September 2024).

## Supplementary Materials

The following Supplementary Materials can be downloaded at https://www.mdpi.com/article/10.3390/ijms25179718/s1.
