# Supplementary material for: Antimicrobial Activity of Chalcones with a Chlorine Atom and Their Glycosides
Source: Int J Mol Sci. 2024 Sep 8;25(17):9718. doi: 10.3390/ijms25179718 (PMC11395246; doi:10.3390/ijms25179718)
Supplement: Supplementary file 1 [file ijms-25-09718-s001.zip › ijms-3172836-supplementary.pdf]

Antimicrobial Activity of Chalcones with a Chlorine Atom and Their Glycosides

Agnieszka Krawczyk-Łebek[1]*, Barbara Żarowska[2], Tomasz Janeczko[1] and Edyta Kostrzewa-Susłow[1]

[1]Department of Food Chemistry and Biocatalysis, Faculty of Biotechnology and Food Science, Wrocław University of Environmental and Life Sciences, 50-375 Wrocław, Poland

[2]Department of Biotechnology and Food Microbiology, Faculty of Biotechnology and Food Science, 51-630 Wrocław University of Environmental and Life Sciences, Wrocław, Poland

Table of contents

Figure S1. MS analysis of 4-chloro-2′-hydroxychalcone (**3**)
Figure S2. HPLC analysis of 4-chloro-2′-hydroxychalcone (**3**)
Figure S3. ^{1}H NMR spectrum (*δ*, acetone-d6, 600 MHz) of 4-chloro-2′-hydroxychalcone (**3**)
Figure S4 ^{1}H NMR spectrum expansion (*δ*, acetone-d6, 600 MHz) of 4-chloro-2′-hydroxychalcone (**3**)
Figure S5. ^{13}C NMR spectrum (*δ*, acetone-d6, 151 MHz) of 4-chloro-2′-hydroxychalcone (**3**)
Figure S6. ^{13}C NMR spectrum expansion (*δ*, acetone-d6, 151 MHz) of 4-chloro-2′-hydroxychalcone (**3**)
Figure S7. ^{13}C NMR spectrum expansion (*δ*, acetone-d6, 151 MHz) of 4-chloro-2′-hydroxychalcone (**3**)
Figure S8. COSY contour map – ^{1}H x ^{1}H of 4-chloro-2′-hydroxychalcone (**3**)
Figure S9. COSY contour map – ^{1}H x ^{1}H expansion of 4-chloro-2′-hydroxychalcone (**3**)
Figure S10. HMQC contour map – ^{1}H x ^{13}C of 4-chloro-2′-hydroxychalcone (**3**)
Figure S11. HMQC contour map – ^{1}H x ^{13}C expansion of 4-chloro-2′-hydroxychalcone (**3**)
Figure S12. HMBC contour map – ^{1}H x ^{13}C of 4-chloro-2′-hydroxychalcone (**3**)
Figure S13. HMBC contour map – ^{1}H x ^{13}C expansion of 4-chloro-2′-hydroxychalcone (**3**)
Figure S14. HMBC contour map – ^{1}H x ^{13}C expansion of 4-chloro-2′-hydroxychalcone (**3**)
Figure S15. 4-Chloro-2′-hydroxychalcone (**3**) physicochemical and ADME parameters prediction using the SwissADME modelling

Figure S16. MS analysis of 5′-chloro-2′-hydroxychalcone (**6**)
Figure S17. HPLC analysis of 5′-chloro-2′-hydroxychalcone (**6**)
Figure S18. ^{1}H NMR spectrum (*δ*, acetone-d6, 600 MHz) of 5′-chloro-2′-hydroxychalcone (**6**)
Figure S19. ^{1}H NMR spectrum expansion (*δ*, acetone-d6, 600 MHz) of 5′-chloro-2′-hydroxychalcone (**6**)
Figure S20. ^{13}C NMR spectrum (*δ*, acetone-d6, 151 MHz) of 5′-chloro-2′-hydroxychalcone (**6**)
Figure S21. ^{13}C NMR spectrum expansion (*δ*, acetone-d6, 151 MHz) of 5′-chloro-2′-hydroxychalcone (**6**)
Figure S22. ^{13}C NMR spectrum expansion (*δ*, acetone-d6, 151 MHz) of 5′-chloro-2′-hydroxychalcone (**6**)
Figure S23. COSY contour map – ^{1}H x ^{1}H of 5′-chloro-2′-hydroxychalcone (**6**)
Figure S24. COSY contour map – ^{1}H x ^{1}H expansion of 5′-chloro-2′-hydroxychalcone (**6**)
Figure S25. HMQC contour map – ^{1}H x ^{13}C of 5′-chloro-2′-hydroxychalcone (**6**)
Figure S26. HMQC contour map – ^{1}H x ^{13}C expansion of 5′-chloro-2′-hydroxychalcone (**6**)
Figure S27. HMBC contour map – ^{1}H x ^{13}C of 5′-chloro-2′-hydroxychalcone (**6**)
Figure S28. HMBC contour map – ^{1}H x ^{13}C expansion of 5′-chloro-2′-hydroxychalcone (**6**)
Figure S29. HMBC contour map – ^{1}H x ^{13}C expansion of 5′-chloro-2′-hydroxychalcone (**6**)
Figure S30. 5′-Chloro-2′-hydroxychalcone (**6**) physicochemical and ADME parameters prediction using the SwissADME modelling

Figure S31. MS analysis of 4-chlorodihydrochalcone 2′-*O*-*β*-D-(4′′-*O*-methyl)-glucopyranoside (**3a**)
Figure S32. HPLC analysis of 4-chlorodihydrochalcone 2′-*O*-*β*-D-(4′′-*O*-methyl)-glucopyranoside (**3a**)
Figure S33. ^{1}H NMR spectrum (*δ*, acetone-d6, 600 MHz) of 4-chlorodihydrochalcone 2′-*O*-*β*-D-(4′′-*O*-methyl)-glucopyranoside (**3a**)

Figure S34. ^{1}H NMR spectrum expansion (*δ*, acetone-d6, 600 MHz) of 4-chlorodihydrochalcone 2′-*O*-*β*-D-(4′′-*O*-methyl)-glucopyranoside (**3a**)
Figure S35. ^{1}H NMR spectrum expansion (*δ*, acetone-d6, 600 MHz) of 4-chlorodihydrochalcone 2′-*O*-*β*-D-(4′′-*O*-methyl)-glucopyranoside (**3a**)
Figure S36. ^{13}C NMR spectrum (*δ*, acetone-d6, 151 MHz) of 4-chlorodihydrochalcone 2′-*O*-*β*-D-(4′′-*O*-methyl)-glucopyranoside (**3a**)
Figure S37. ^{13}C NMR spectrum expansion (*δ*, acetone-d6, 151 MHz) of 4-chlorodihydrochalcone 2′-*O*-*β*-D-(4′′-*O*-methyl)-glucopyranoside (**3a**)
Figure S38. ^{13}C NMR spectrum expansion (*δ*, acetone-d6, 151 MHz) of 4-chlorodihydrochalcone 2′-*O*-*β*-D-(4′′-*O*-methyl)-glucopyranoside (**3a**)
Figure S39. COSY contour map – ^{1}H x ^{1}H of 4-chlorodihydrochalcone 2′-*O*-*β*-D-(4′′-*O*-methyl)-glucopyranoside (**3a**)
Figure S40. COSY contour map – ^{1}H x ^{1}H expansion of 4-chlorodihydrochalcone 2′-*O*-*β*-D-(4′′-*O*-methyl)-glucopyranoside (**3a**)
Figure S41. COSY contour map – ^{1}H x ^{1}H expansion of 4-chlorodihydrochalcone 2′-*O*-*β*-D-(4′′-*O*-methyl)-glucopyranoside (**3a**)
Figure S42. HMQC contour map – ^{1}H x ^{13}C of 4-chlorodihydrochalcone 2′-*O*-*β*-D-(4′′-*O*-methyl)-glucopyranoside (**3a**)
Figure S43. HMQC contour map – ^{1}H x ^{13}C expansion of 4-chlorodihydrochalcone 2′-*O*-*β*-D-(4′′-*O*-methyl)-glucopyranoside (**3a**)
Figure S44. HMQC contour map – ^{1}H x ^{13}C expansion of 4-chlorodihydrochalcone 2′-*O*-*β*-D-(4′′-*O*-methyl)-glucopyranoside (**3a**)
Figure S45. HMBC contour map – ^{1}H x ^{13}C of 4-chlorodihydrochalcone 2′-*O*-*β*-D-(4′′-*O*-methyl)-glucopyranoside (**3a**)
Figure S46. HMBC contour map – ^{1}H x ^{13}C expansion of 4-chlorodihydrochalcone 2′-*O*-*β*-D-(4′′-*O*-methyl)-glucopyranoside (**3a**)
Figure S47. HMBC contour map – ^{1}H x ^{13}C expansion of 4-chlorodihydrochalcone 2′-*O*-*β*-D-(4′′-*O*-methyl)-glucopyranoside (**3a**)
Figure S48. HMBC contour map – ^{1}H x ^{13}C expansion of 4-chlorodihydrochalcone 2′-*O*-*β*-D-(4′′-*O*-methyl)-glucopyranoside (**3a**)
Figure S49. HMBC contour map – ^{1}H x ^{13}C expansion of 4-chlorodihydrochalcone 2′-*O*-*β*-D-(4′′-*O*-methyl)-glucopyranoside (**3a**)
Figure S50. HMBC contour map – ^{1}H x ^{13}C expansion of 4-chlorodihydrochalcone 2′-*O*-*β*-D-(4′′-*O*-methyl)-glucopyranoside (**3a**)
Figure S51. 4-Chlorodihydrochalcone 2′-*O*-*β*-D-(4′′-*O*-methyl)-glucopyranoside (**3a**) physicochemical and ADME parameters prediction using the SwissADME modelling

Figure S52. MS analysis of 4-chloro-2′-hydroxydihydrochalcone 5′-*O*-*β*-D-(4′′-*O*-methyl)-glucopyranoside (**3b**)
Figure S53. HPLC analysis of 4-chloro-2′-hydroxydihydrochalcone 5′-*O*-*β*-D-(4′′-*O*-methyl)-glucopyranoside (**3b**)
Figure S54. ^{1}H NMR spectrum (*δ*, acetone-d6, 600 MHz) of 4-chloro-2′-hydroxydihydrochalcone 5′-*O*-*β*-D-(4′′-*O*-methyl)-glucopyranoside (**3b**)
Figure S55. ^{1}H NMR spectrum expansion (*δ*, acetone-d6, 600 MHz) of 4-chloro-2′-hydroxydihydrochalcone 5′-*O*-*β*-D-(4′′-*O*-methyl)-glucopyranoside (**3b**)
Figure S56. ^{1}H NMR spectrum expansion (*δ*, acetone-d6, 600 MHz) of 4-chloro-2′-hydroxydihydrochalcone 5′-*O*-*β*-D-(4′′-*O*-methyl)-glucopyranoside (**3b**)
Figure S57. ^{13}C NMR spectrum (*δ*, acetone-d6, 151 MHz) of 4-chloro-2′-hydroxydihydrochalcone 5′-*O*-*β*-D-(4′′-*O*-methyl)-glucopyranoside (**3b**)
Figure S58. ^{13}C NMR spectrum expansion (*δ*, acetone-d6, 151 MHz) of 4-chloro-2′-hydroxydihydrochalcone 5′-*O*-*β*-D-(4′′-*O*-methyl)-glucopyranoside (**3b**)
Figure S59. ^{13}C NMR spectrum expansion (*δ*, acetone-d6, 151 MHz) of 4-chloro-2′-hydroxydihydrochalcone 5′-*O*-*β*-D-(4′′-*O*-methyl)-glucopyranoside (**3b**)
Figure S60. COSY contour map – ^{1}H x ^{1}H of 4-chloro-2′-hydroxydihydrochalcone 5′-*O*-*β*-D-(4′′-*O*-methyl)-glucopyranoside (**3b**)
Figure S61. COSY contour map – ^{1}H x ^{1}H expansion of 4-chloro-2′-hydroxydihydrochalcone 5′-*O*-*β*-D-(4′′-*O*-methyl)-glucopyranoside (**3b**)
Figure S62. COSY contour map – ^{1}H x ^{1}H expansion of 4-chloro-2′-hydroxydihydrochalcone 5′-*O*-*β*-D-(4′′-*O*-methyl)-glucopyranoside (**3b**)
Figure S63. HMQC contour map – ^{1}H x ^{13}C of 4-chloro-2′-hydroxydihydrochalcone 5′-*O*-*β*-D-(4′′-*O*-methyl)-glucopyranoside (**3b**)

Figure S64. HMQC contour map – ^{1}H x ^{13}C expansion of 4-chloro-2′-hydroxydihydrochalcone 5′-*O*-*β*-D-(4′′-*O*-methyl)-glucopyranoside (**3b**)
Figure S65. HMQC contour map – ^{1}H x ^{13}C expansion of 4-chloro-2′-hydroxydihydrochalcone 5′-*O*-*β*-D-(4′′-*O*-methyl)-glucopyranoside (**3b**)
Figure S66. HMBC contour map – ^{1}H x ^{13}C of 4-chloro-2′-hydroxydihydrochalcone 5′-*O*-*β*-D-(4′′-*O*-methyl)-glucopyranoside (**3b**)
Figure S67. HMBC contour map – ^{1}H x ^{13}C expansion of 4-chloro-2′-hydroxydihydrochalcone 5′-*O*-*β*-D-(4′′-*O*-methyl)-glucopyranoside (**3b**)
Figure S68. HMBC contour map – ^{1}H x ^{13}C expansion of 4-chloro-2′-hydroxydihydrochalcone 5′-*O*-*β*-D-(4′′-*O*-methyl)-glucopyranoside (**3b**)
Figure S69. HMBC contour map – ^{1}H x ^{13}C expansion of 4-chloro-2′-hydroxydihydrochalcone 5′-*O*-*β*-D-(4′′-*O*-methyl)-glucopyranoside (**3b**)
Figure S70. HMBC contour map – ^{1}H x ^{13}C expansion of 4-chloro-2′-hydroxydihydrochalcone 5′-*O*-*β*-D-(4′′-*O*-methyl)-glucopyranoside (**3b**)
Figure S71. HMBC contour map – ^{1}H x ^{13}C expansion of 4-chloro-2′-hydroxydihydrochalcone 5′-*O*-*β*-D-(4′′-*O*-methyl)-glucopyranoside (**3b**)
Figure S72. 4-Chloro-2′-hydroxydihydrochalcone 5′-*O*-*β*-D-(4′′-*O*-methyl)-glucopyranoside (**3b**) physicochemical and ADME parameters prediction using the SwissADME modelling

Figure S73. MS analysis of 4-chloro-2′-hydroxydihydrochalcone 3-*O*-*β*-D-(4′′-*O*-methyl)-glucopyranoside (**3c**)
Figure S74. HPLC analysis of 4-chloro-2′-hydroxydihydrochalcone 3-*O*-*β*-D-(4′′-*O*-methyl)-glucopyranoside (**3c**)
Figure S75. ^{1}H NMR spectrum (*δ*, acetone-d6, 600 MHz) of 4-chloro-2′-hydroxydihydrochalcone 3-*O*-*β*-D-(4′′-*O*-methyl)-glucopyranoside (**3c**)
Figure S76. ^{1}H NMR spectrum expansion (*δ*, acetone-d6, 600 MHz) of 4-chloro-2′-hydroxydihydrochalcone 3-*O*-*β*-D-(4′′-*O*-methyl)-glucopyranoside (**3c**)
Figure S77. ^{1}H NMR spectrum expansion (*δ*, acetone-d6, 600 MHz) of 4-chloro-2′-hydroxydihydrochalcone 3-*O*-*β*-D-(4′′-*O*-methyl)-glucopyranoside (**3c**)
Figure S78. ^{13}C NMR spectrum (*δ*, acetone-d6, 151 MHz) of 4-chloro-2′-hydroxydihydrochalcone 3-*O*-*β*-D-(4′′-*O*-methyl)-glucopyranoside (**3c**)
Figure S79. ^{13}C NMR spectrum expansion (*δ*, acetone-d6, 151 MHz) of 4-chloro-2′-hydroxydihydrochalcone 3-*O*-*β*-D-(4′′-*O*-methyl)-glucopyranoside (**3c**)
Figure S80. ^{13}C NMR spectrum expansion (*δ*, acetone-d6, 151 MHz) of 4-chloro-2′-hydroxydihydrochalcone 3-*O*-*β*-D-(4′′-*O*-methyl)-glucopyranoside (**3c**)
Figure S81. COSY contour map – ^{1}H x ^{1}H of 4-chloro-2′-hydroxydihydrochalcone 3-*O*-*β*-D-(4′′-*O*-methyl)-glucopyranoside (**3c**)
Figure S82. COSY contour map – ^{1}H x ^{1}H expansion of 4-chloro-2′-hydroxydihydrochalcone 3-*O*-*β*-D-(4′′-*O*-methyl)-glucopyranoside (**3c**)
Figure S83. COSY contour map – ^{1}H x ^{1}H expansion of 4-chloro-2′-hydroxydihydrochalcone 3-*O*-*β*-D-(4′′-*O*-methyl)-glucopyranoside (**3c**)
Figure S84. HMQC contour map – ^{1}H x ^{13}C of 4-chloro-2′-hydroxydihydrochalcone 3-*O*-*β*-D-(4′′-*O*-methyl)-glucopyranoside (**3c**)
Figure S85. HMQC contour map – ^{1}H x ^{13}C expansion of 4-chloro-2′-hydroxydihydrochalcone 3-*O*-*β*-D-(4′′-*O*-methyl)-glucopyranoside (**3c**)
Figure S86. HMQC contour map – ^{1}H x ^{13}C expansion of 4-chloro-2′-hydroxydihydrochalcone 3-*O*-*β*-D-(4′′-*O*-methyl)-glucopyranoside (**3c**)
Figure S87. HMBC contour map – ^{1}H x ^{13}C of 4-chloro-2′-hydroxydihydrochalcone 3-*O*-*β*-D-(4′′-*O*-methyl)-glucopyranoside (**3c**)
Figure S88. HMBC contour map – ^{1}H x ^{13}C expansion of 4-chloro-2′-hydroxydihydrochalcone 3-*O*-*β*-D-(4′′-*O*-methyl)-glucopyranoside (**3c**)
Figure S89. HMBC contour map – ^{1}H x ^{13}C expansion of 4-chloro-2′-hydroxydihydrochalcone 3-*O*-*β*-D-(4′′-*O*-methyl)-glucopyranoside (**3c**)
Figure S90. HMBC contour map – ^{1}H x ^{13}C expansion of 4-chloro-2′-hydroxydihydrochalcone 3-*O*-*β*-D-(4′′-*O*-methyl)-glucopyranoside (**3c**)
Figure S91. HMBC contour map – ^{1}H x ^{13}C expansion of 4-chloro-2′-hydroxydihydrochalcone 3-*O*-*β*-D-(4′′-*O*-methyl)-glucopyranoside (**3c**)
Figure S92. HMBC contour map – ^{1}H x ^{13}C expansion of 4-chloro-2′-hydroxydihydrochalcone 3-*O*-*β*-D-(4′′-*O*-methyl)-glucopyranoside (**3c**)

Figure S93. 4-Chloro-2′-hydroxydihydrochalcone 3-*O*-*β*-D-(4′′-*O*-methyl)-glucopyranoside (**3c**) physicochemical and ADME parameters prediction using the SwissADME modelling

Figure S94. MS analysis of 5′-chlorodihydrochalcone 2′-*O*-*β*-D-(4′′-*O*-methyl)-glucopyranoside (**6a**)
Figure S95. HPLC analysis of 5′-chlorodihydrochalcone 2′-*O*-*β*-D-(4′′-*O*-methyl)-glucopyranoside (**6a**)
Figure S96. ^{1}H NMR spectrum (*δ*, acetone-d6, 600 MHz) of 5′-chlorodihydrochalcone 2′-*O*-*β*-D-(4′′-*O*-methyl)-glucopyranoside (**6a**)
Figure S97. ^{1}H NMR spectrum expansion (*δ*, acetone-d6, 600 MHz) of 5′-chlorodihydrochalcone 2′-*O*-*β*-D-(4′′-*O*-methyl)-glucopyranoside (**6a**)
Figure S98. ^{1}H NMR spectrum expansion (*δ*, acetone-d6, 600 MHz) of 4-chlorodihydrochalcone 2′-*O*-*β*-D-(4′′-*O*-methyl)-glucopyranoside (**6a**)
Figure S99. ^{13}C NMR spectrum (*δ*, acetone-d6, 151 MHz) of 5′-chlorodihydrochalcone 2′-*O*-*β*-D-(4′′-*O*-methyl)-glucopyranoside (**6a**)
Figure S100. ^{13}C NMR spectrum expansion (*δ*, acetone-d6, 151 MHz) of 5′-chlorodihydrochalcone 2′-*O*-*β*-D-(4′′-*O*-methyl)-glucopyranoside (**6a**)
Figure S101. ^{13}C NMR spectrum expansion (*δ*, acetone-d6, 151 MHz) of 5′-chlorodihydrochalcone 2′-*O*-*β*-D-(4′′-*O*-methyl)-glucopyranoside (**6a**)
Figure S102. COSY contour map – ^{1}H x ^{1}H of 5′-chlorodihydrochalcone 2′-*O*-*β*-D-(4′′-*O*-methyl)-glucopyranoside (**6a**)
Figure S103. COSY contour map – ^{1}H x ^{1}H expansion of 5′-chlorodihydrochalcone 2′-*O*-*β*-D-(4′′-*O*-methyl)-glucopyranoside (**6a**)
Figure S104. COSY contour map – ^{1}H x ^{1}H expansion of 4-chlorodihydrochalcone 2′-*O*-*β*-D-(4′′-*O*-methyl)-glucopyranoside (**6a**)
Figure S105. HMQC contour map – ^{1}H x ^{13}C of 5′-chlorodihydrochalcone 2′-*O*-*β*-D-(4′′-*O*-methyl)-glucopyranoside (**6a**)
Figure S106. HMQC contour map – ^{1}H x ^{13}C expansion of 5′-chlorodihydrochalcone 2′-*O*-*β*-D-(4′′-*O*-methyl)-glucopyranoside (**6a**)
Figure S107. HMQC contour map – ^{1}H x ^{13}C expansion of 5′-chlorodihydrochalcone 2′-*O*-*β*-D-(4′′-*O*-methyl)-glucopyranoside (**6a**)
Figure S108. HMBC contour map – ^{1}H x ^{13}C of 5′-chlorodihydrochalcone 2′-*O*-*β*-D-(4′′-*O*-methyl)-glucopyranoside (**6a**)
Figure S109. HMBC contour map – ^{1}H x ^{13}C expansion of 5′-chlorodihydrochalcone 2′-*O*-*β*-D-(4′′-*O*-methyl)-glucopyranoside (**6a**)
Figure S110. HMBC contour map – ^{1}H x ^{13}C expansion of 5′-chlorodihydrochalcone 2′-*O*-*β*-D-(4′′-*O*-methyl)-glucopyranoside (**6a**)
Figure S111. HMBC contour map – ^{1}H x ^{13}C expansion of 5′-chlorodihydrochalcone 2′-*O*-*β*-D-(4′′-*O*-methyl)-glucopyranoside (**6a**)
Figure S112. HMBC contour map – ^{1}H x ^{13}C expansion of 5′-chlorodihydrochalcone 2′-*O*-*β*-D-(4′′-*O*-methyl)-glucopyranoside (**6a**)
Figure S113. HMBC contour map – ^{1}H x ^{13}C expansion of 5′-chlorodihydrochalcone 2′-*O*-*β*-D-(4′′-*O*-methyl)-glucopyranoside (**6a**)
Figure S114. 5′-Chlorodihydrochalcone 2′-*O*-*β*-D-(4′′-*O*-methyl)-glucopyranoside (**6a**) physicochemical and ADME parameters prediction using the SwissADME modelling

Figure S115. MS analysis of 5′-chloro-2′-hydroxychalcone 3-*O*-*β*-D-(4′′-*O*-methyl)-glucopyranoside (**6b**)
Figure S116. HPLC analysis of 5′-chloro-2′-hydroxychalcone 3-*O*-*β*-D-(4′′-*O*-methyl)-glucopyranoside (**6b**)
Figure S117. ^{1}H NMR spectrum (*δ*, acetone-d6, 600 MHz) of 5′-chloro-2′-hydroxychalcone 3-*O*-*β*-D-(4′′-*O*-methyl)-glucopyranoside (**6b**)
Figure S118. ^{1}H NMR spectrum expansion (*δ*, acetone-d6, 600 MHz) of 5′-chloro-2′-hydroxychalcone 3-*O*-*β*-D-(4′′-*O*-methyl)-glucopyranoside (**6b**)
Figure S119. ^{1}H NMR spectrum expansion (*δ*, acetone-d6, 600 MHz) of 5′-chloro-2′-hydroxychalcone 3-*O*-*β*-D-(4′′-*O*-methyl)-glucopyranoside (**6b**)
Figure S120. ^{13}C NMR spectrum (*δ*, acetone-d6, 151 MHz) of 5′-chloro-2′-hydroxychalcone 3-*O*-*β*-D-(4′′-*O*-methyl)-glucopyranoside (**6b**)
Figure S121. ^{13}C NMR spectrum expansion (*δ*, acetone-d6, 151 MHz) of 5′-chloro-2′-hydroxychalcone 3-*O*-*β*-D-(4′′-*O*-methyl)-glucopyranoside (**6b**)
Figure S122. ^{13}C NMR spectrum expansion (*δ*, acetone-d6, 151 MHz) of 5′-chloro-2′-hydroxychalcone 3-*O*-*β*-D-(4′′-*O*-methyl)-glucopyranoside (**6b**)

Figure S123. COSY contour map – ^{1}H x ^{1}H of 4-chloro-2′-hydroxydihydrochalcone 5′-chloro-2′-hydroxychalcone 3-*O*-*β*-D-(4′′-*O*-methyl)-glucopyranoside (**6b**)
Figure S124. COSY contour map – ^{1}H x ^{1}H expansion of 5′-chloro-2′-hydroxychalcone 3-*O*-*β*-D-(4′′-*O*-methyl)-glucopyranoside (**6b**)
Figure S125. COSY contour map – ^{1}H x ^{1}H expansion of 5′-chloro-2′-hydroxychalcone 3-*O*-*β*-D-(4′′-*O*-methyl)-glucopyranoside (**6b**)
Figure S126. HMQC contour map – ^{1}H x ^{13}C of 5′-chloro-2′-hydroxychalcone 3-*O*-*β*-D-(4′′-*O*-methyl)-glucopyranoside (**6b**)
Figure S127. HMQC contour map – ^{1}H x ^{13}C expansion of 5′-chloro-2′-hydroxychalcone 3-*O*-*β*-D-(4′′-*O*-methyl)-glucopyranoside (**6b**)
Figure S128. HMQC contour map – ^{1}H x ^{13}C expansion of 5′-chloro-2′-hydroxychalcone 3-*O*-*β*-D-(4′′-*O*-methyl)-glucopyranoside (**6b**)
Figure S129. HMBC contour map – ^{1}H x ^{13}C of 5′-chloro-2′-hydroxychalcone 3-*O*-*β*-D-(4′′-*O*-methyl)-glucopyranoside (**6b**)
Figure S130. HMBC contour map – ^{1}H x ^{13}C expansion of 5′-chloro-2′-hydroxychalcone 3-*O*-*β*-D-(4′′-*O*-methyl)-glucopyranoside (**6b**)
Figure S131. HMBC contour map – ^{1}H x ^{13}C expansion of 5′-chloro-2′-hydroxychalcone 3-*O*-*β*-D-(4′′-*O*-methyl)-glucopyranoside (**6b**)
Figure S132. HMBC contour map – ^{1}H x ^{13}C expansion of 5′-chloro-2′-hydroxychalcone 3-*O*-*β*-D-(4′′-*O*-methyl)-glucopyranoside (**6b**)
Figure S133. HMBC contour map – ^{1}H x ^{13}C expansion of 5′-chloro-2′-hydroxychalcone 3-*O*-*β*-D-(4′′-*O*-methyl)-glucopyranoside (**6b**)
Figure S134. 5′-Chloro-2′-hydroxychalcone 3-*O*-*β*-D-(4′′-*O*-methyl)-glucopyranoside (**6b**) physicochemical and ADME parameters prediction using the SwissADME modelling

Figure S135. 2′-Hydroxychalcone (**7**) physicochemical and ADME parameters prediction using the SwissADME modelling

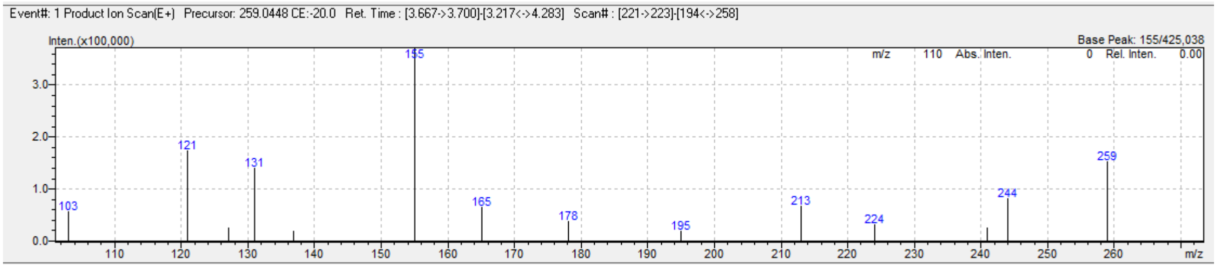

Figure S1. MS analysis of 4-chloro-2′-hydroxychalcone (**3**)

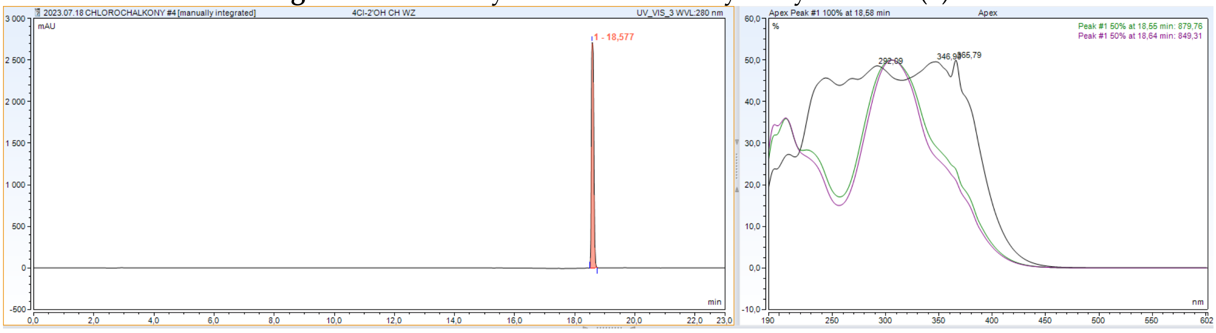

Figure S2. HPLC analysis of 4-chloro-2′-hydroxychalcone (**3**)

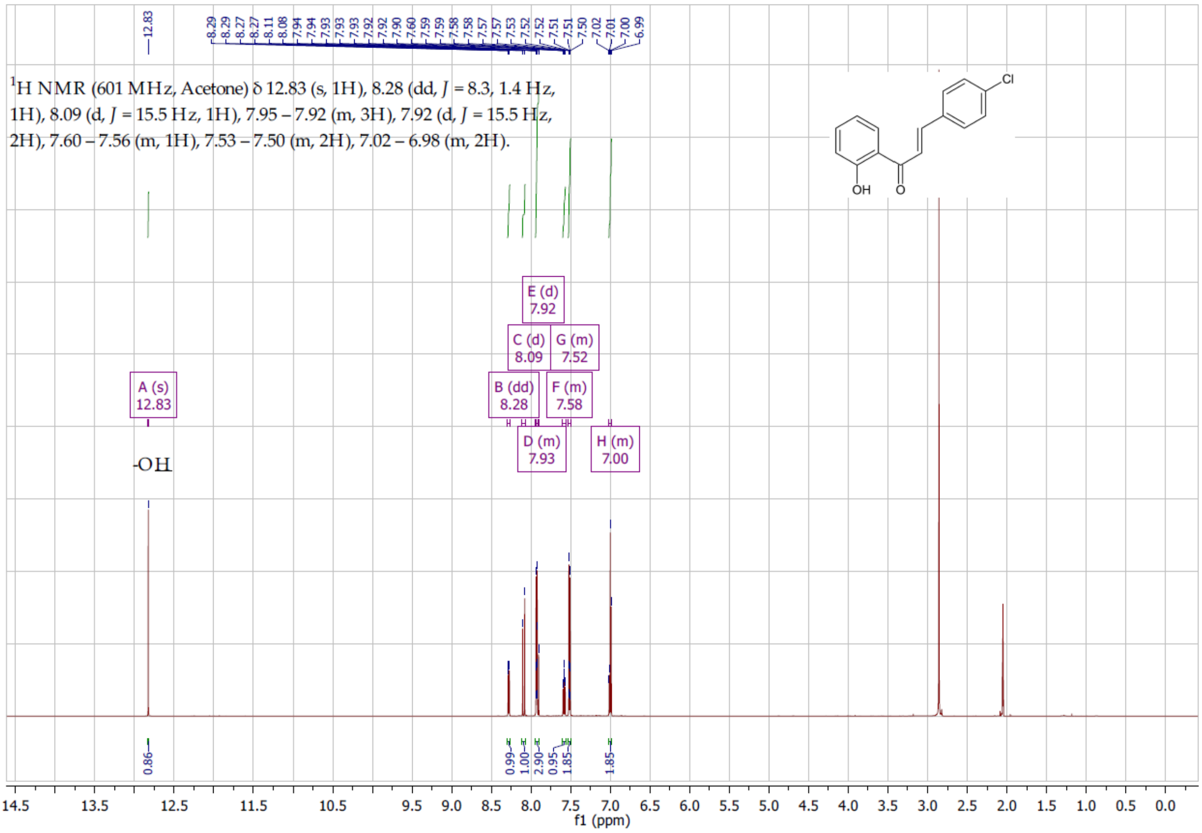

Figure S3. ^{1}H NMR spectrum (δ, acetone-d6, 600 MHz) of 4-chloro-2′-hydroxychalcone (**3**)

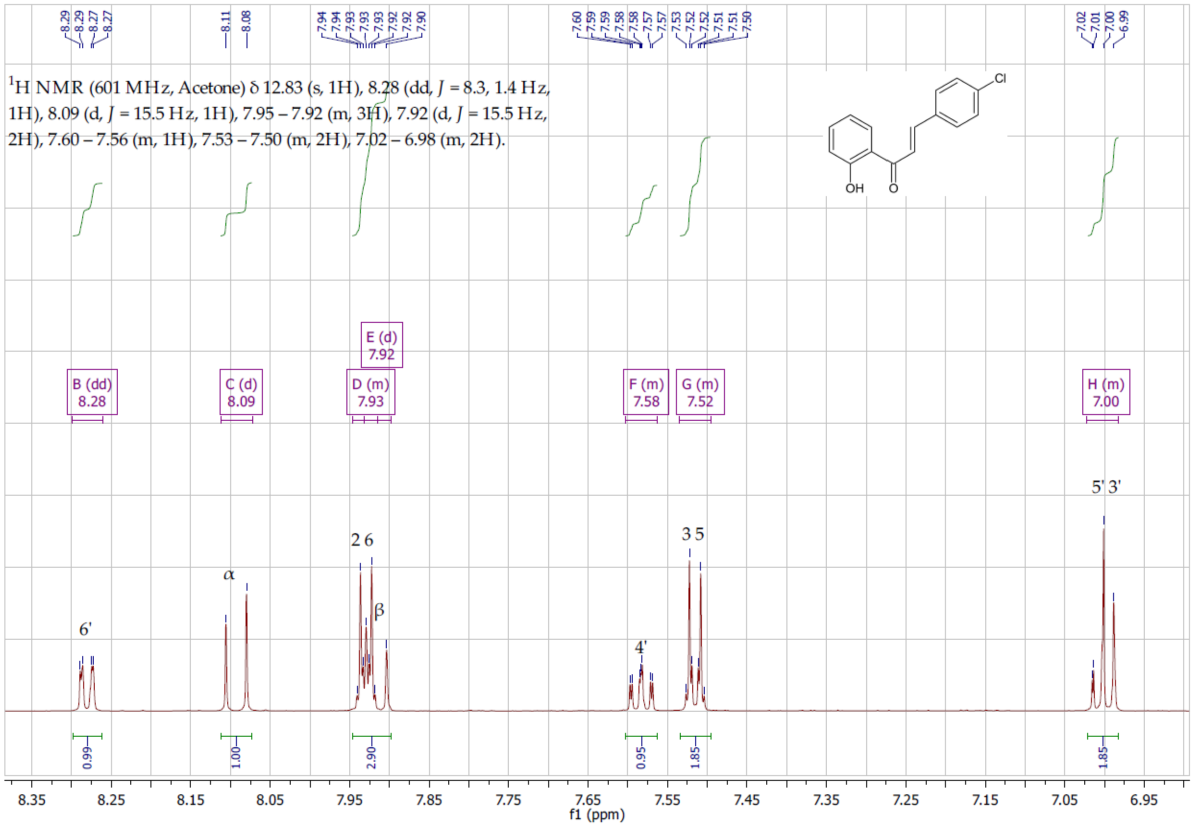

Figure S4 ^{1}H NMR spectrum expansion (*δ*, acetone-d6, 600 MHz) of 4-chloro-2′-hydroxychalcone (**3**)

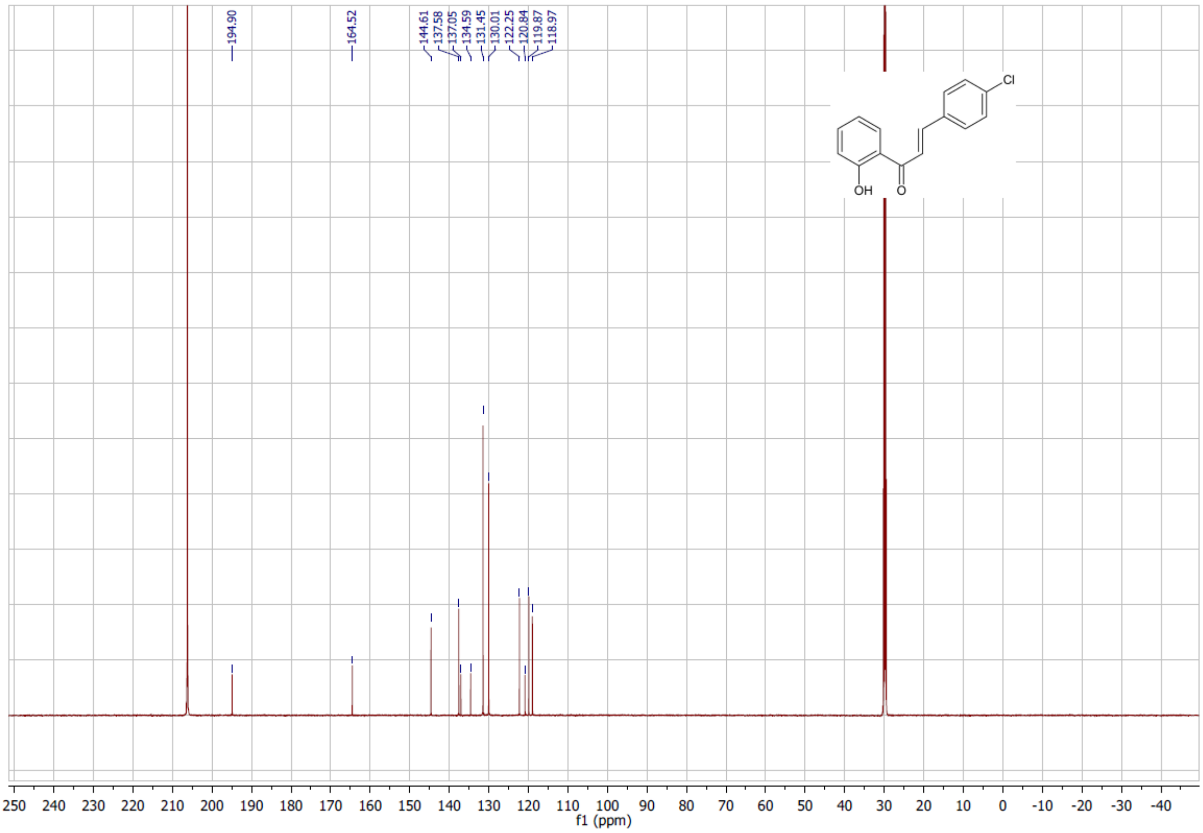

Figure S5. ^{13}C NMR spectrum (*δ*, acetone-d6, 151 MHz) of 4-chloro-2′-hydroxychalcone (**3**)

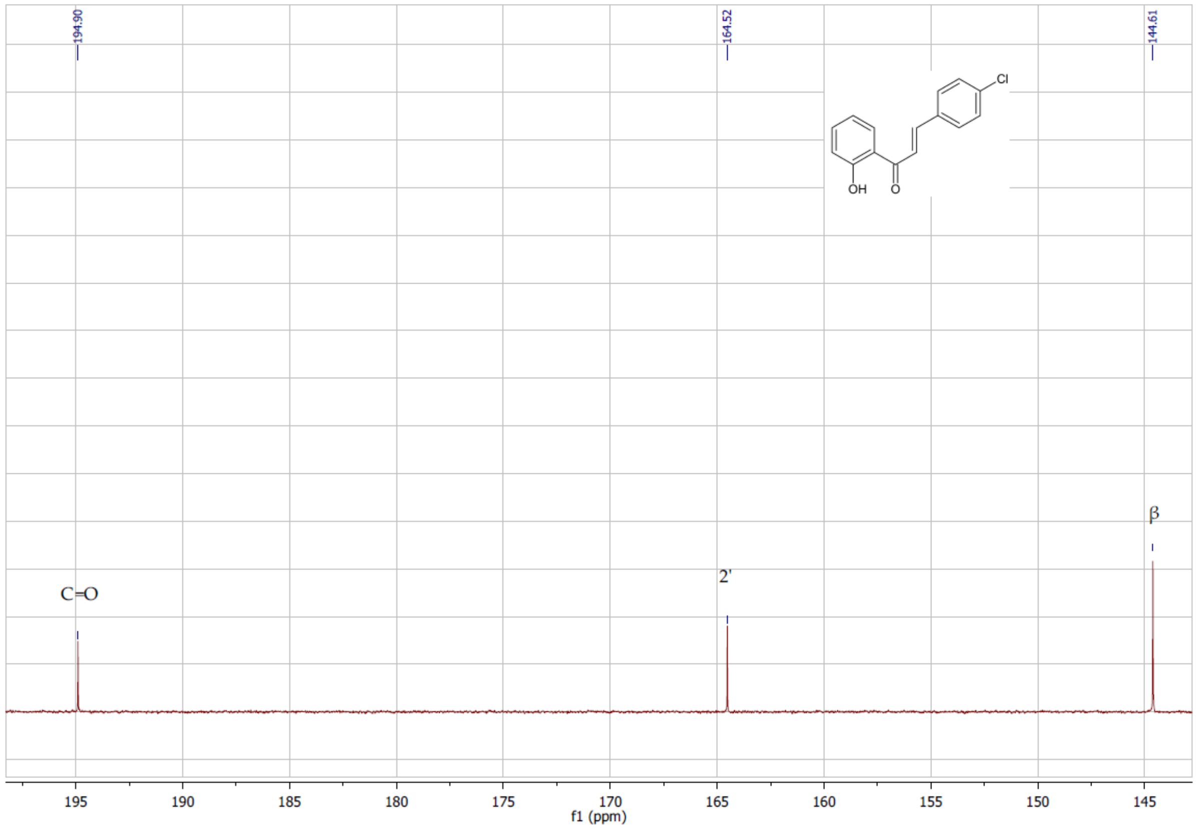

Figure S6. ^{13}C NMR spectrum expansion (δ, acetone-d6, 151 MHz) of 4-chloro-2′-hydroxychalcone (**3**)

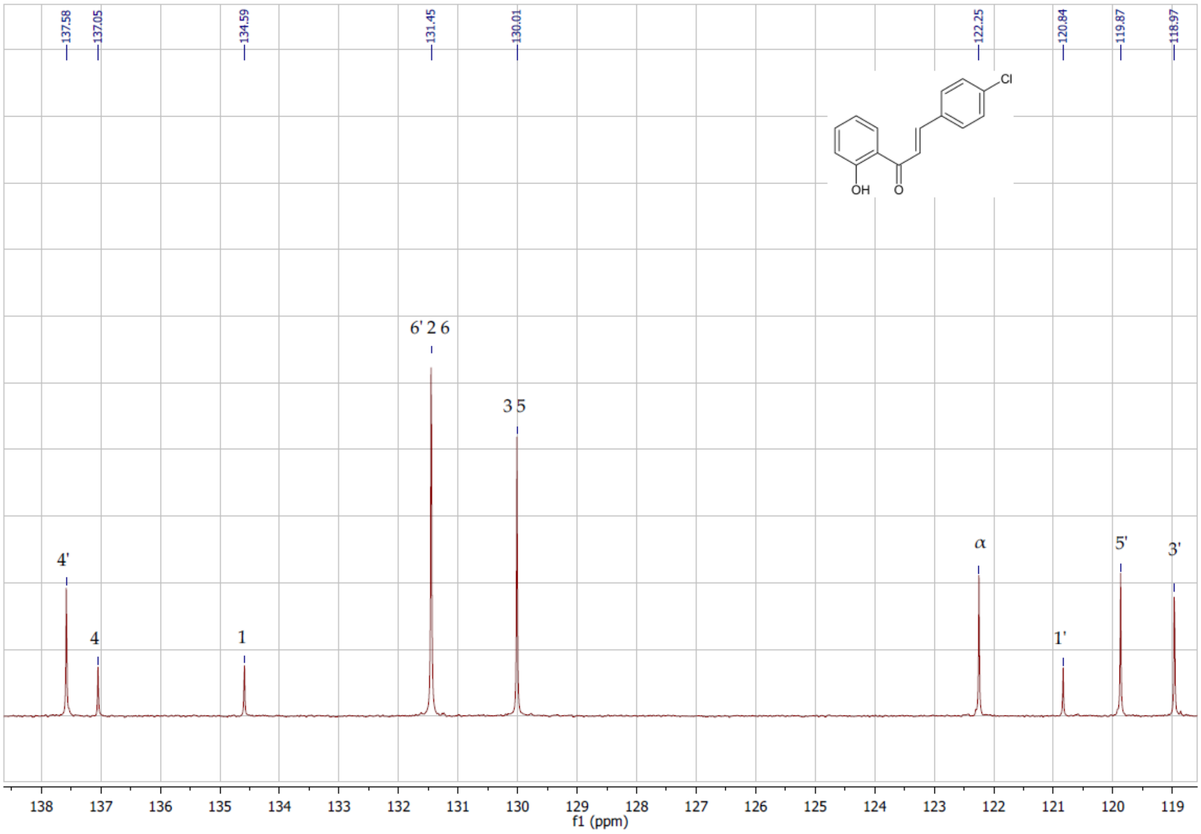

Figure S7. ^{13}C NMR spectrum expansion (δ, acetone-d6, 151 MHz) of 4-chloro-2′-hydroxychalcone (**3**)

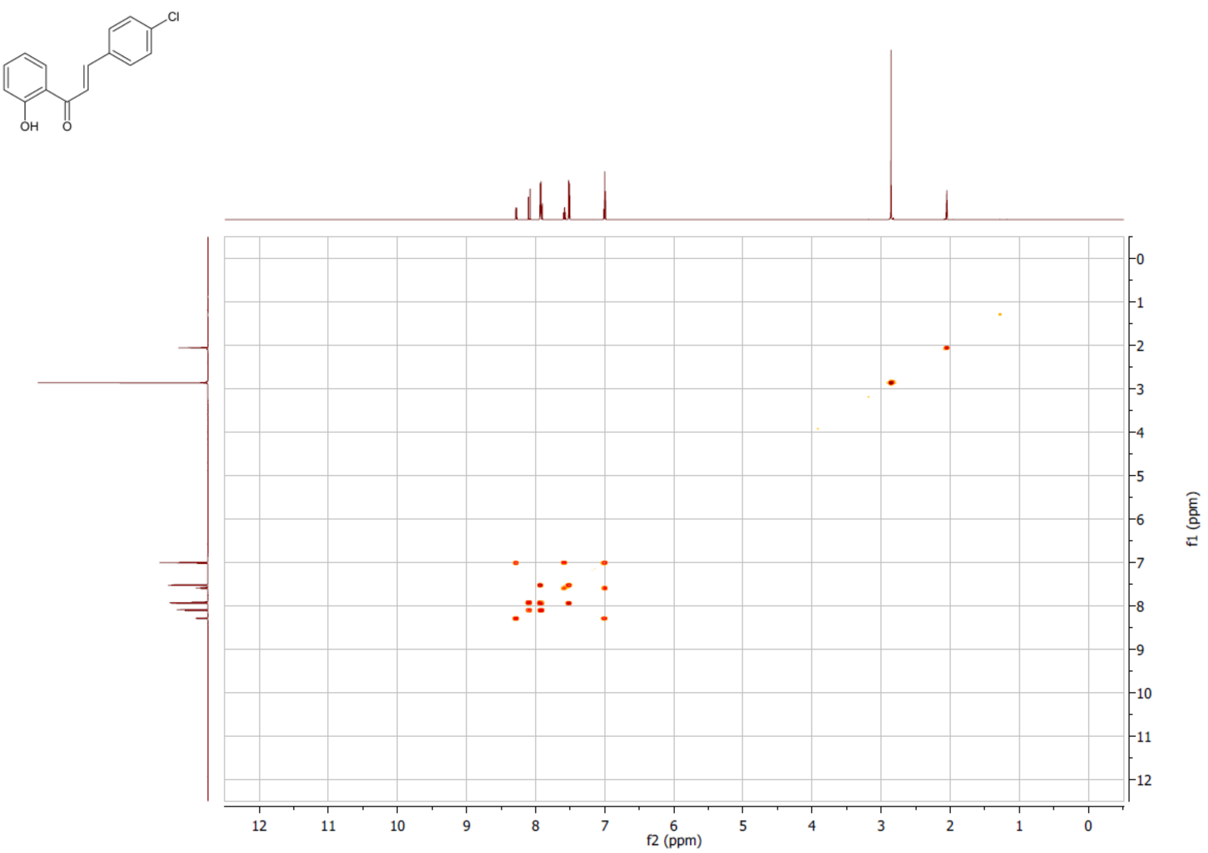

Figure S8. COSY contour map – ^{1}H x ^{1}H of 4-chloro-2′-hydroxychalcone (**3**)

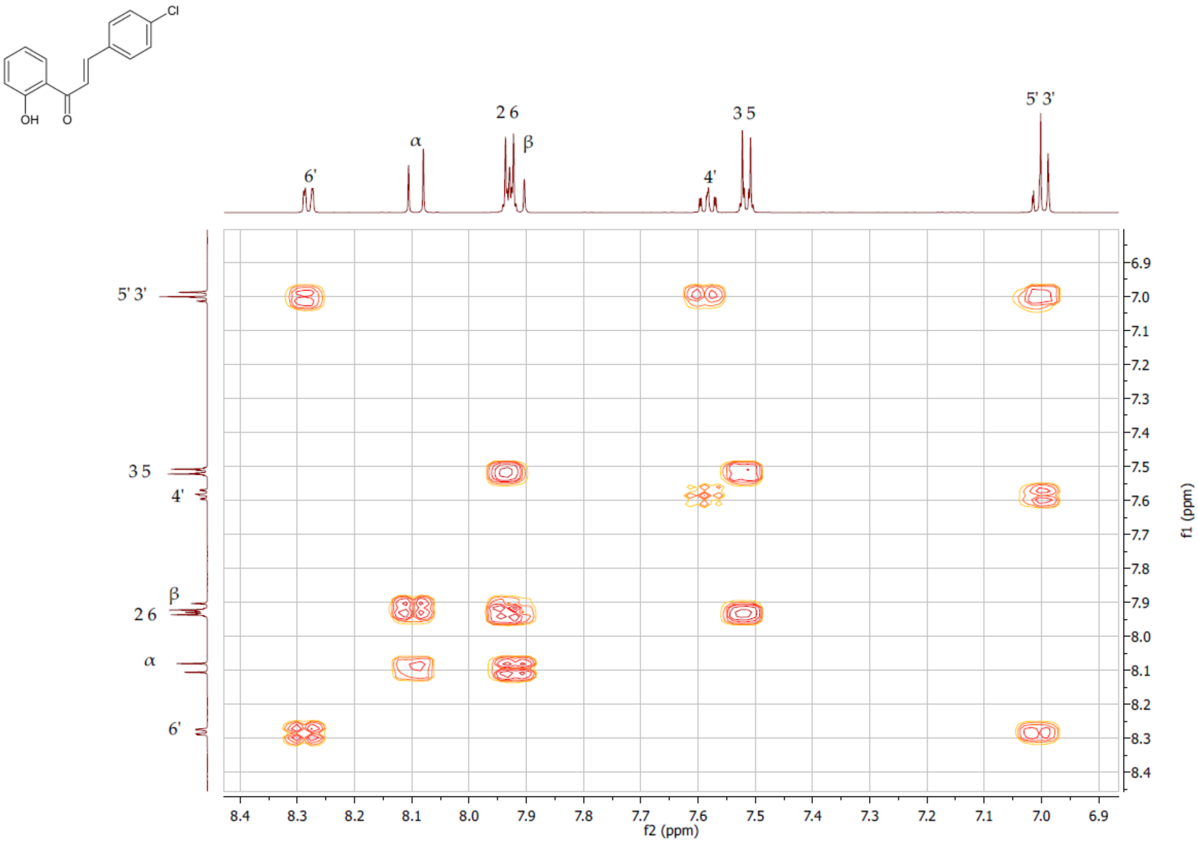

Figure S9. COSY contour map – ^{1}H x ^{1}H expansion of 4-chloro-2′-hydroxychalcone (**3**)

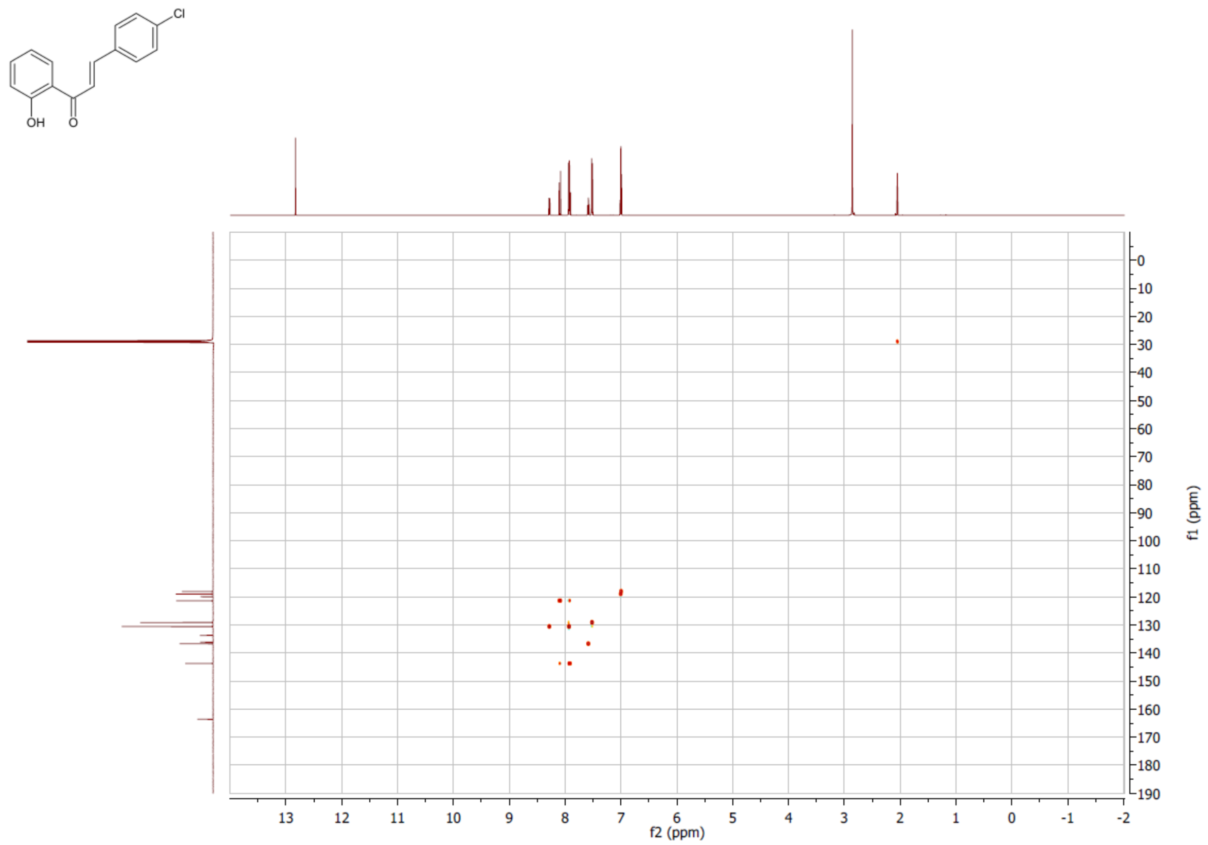

Figure S10. HMQC contour map – [1]H x ^{13}C of 4-chloro-2′-hydroxychalcone (**3**)

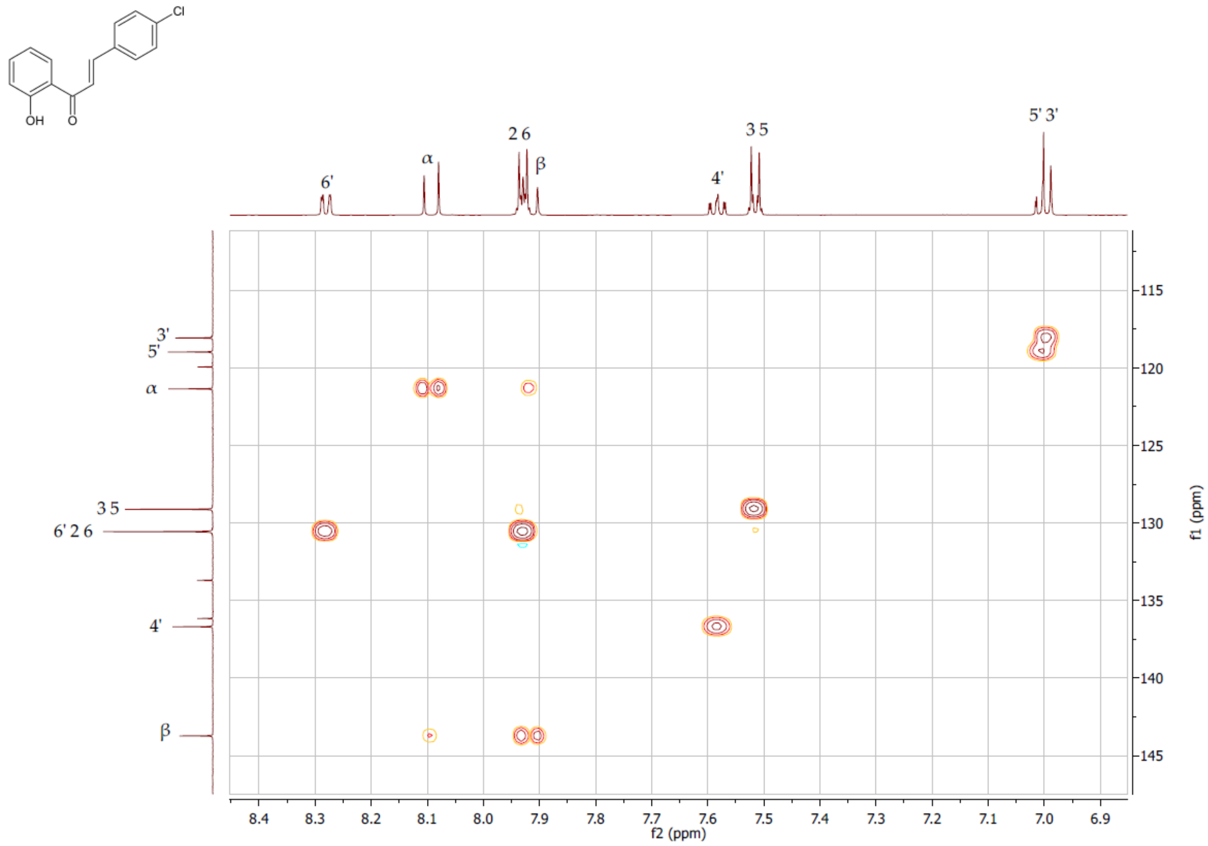

Figure S11. HMQC contour map – [1]H x ^{13}C expansion of 4-chloro-2′-hydroxychalcone (**3**)

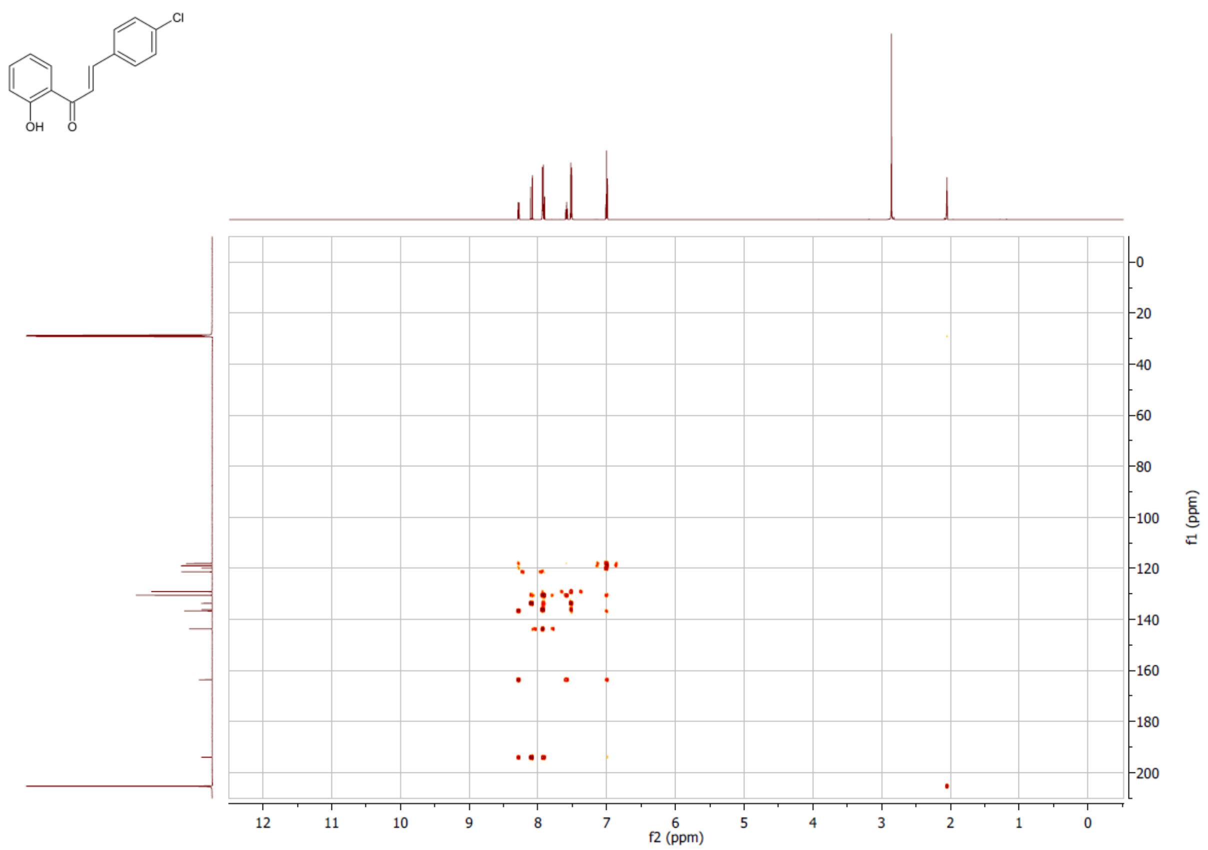

Figure S12. HMBC contour map – ^{1}H x ^{13}C of 4-chloro-2′-hydroxychalcone (**3**)

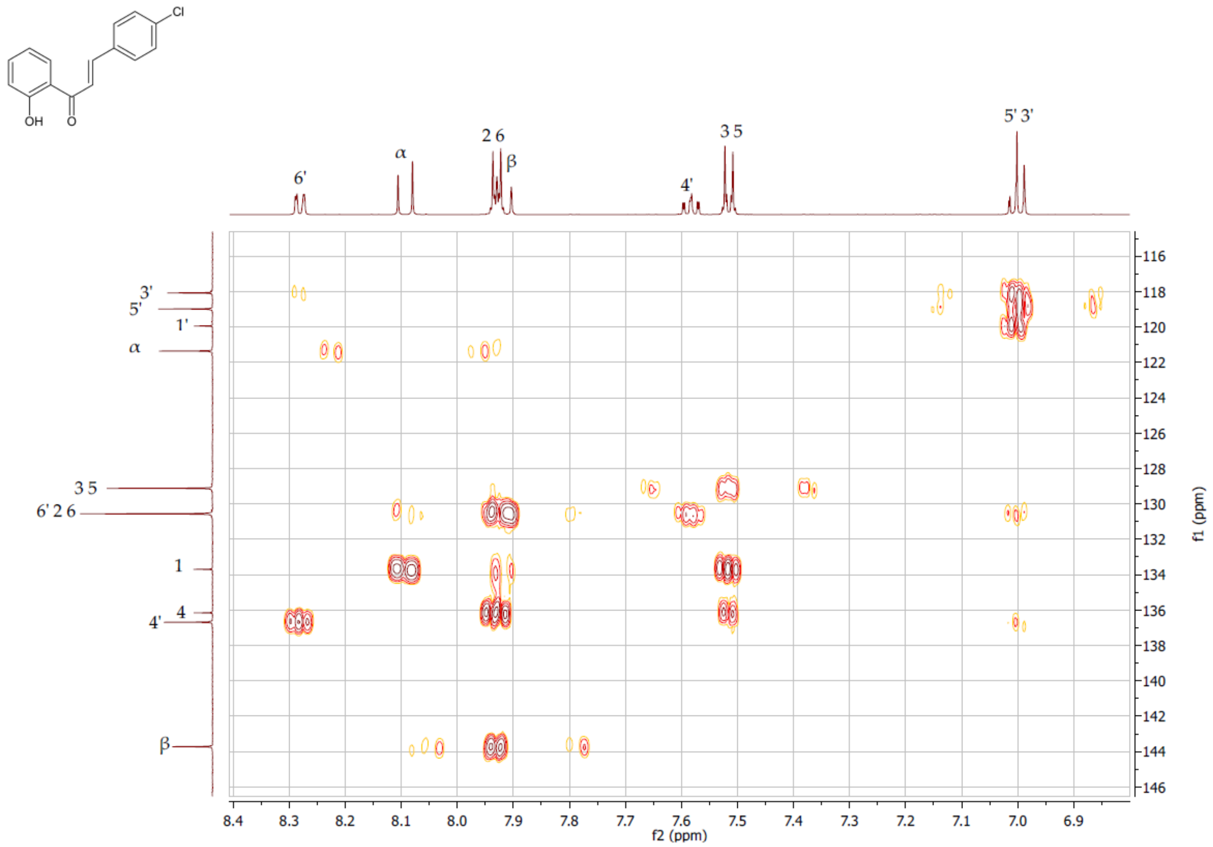

Figure S13. HMBC contour map – ^{1}H x ^{13}C expansion of 4-chloro-2′-hydroxychalcone (**3**)

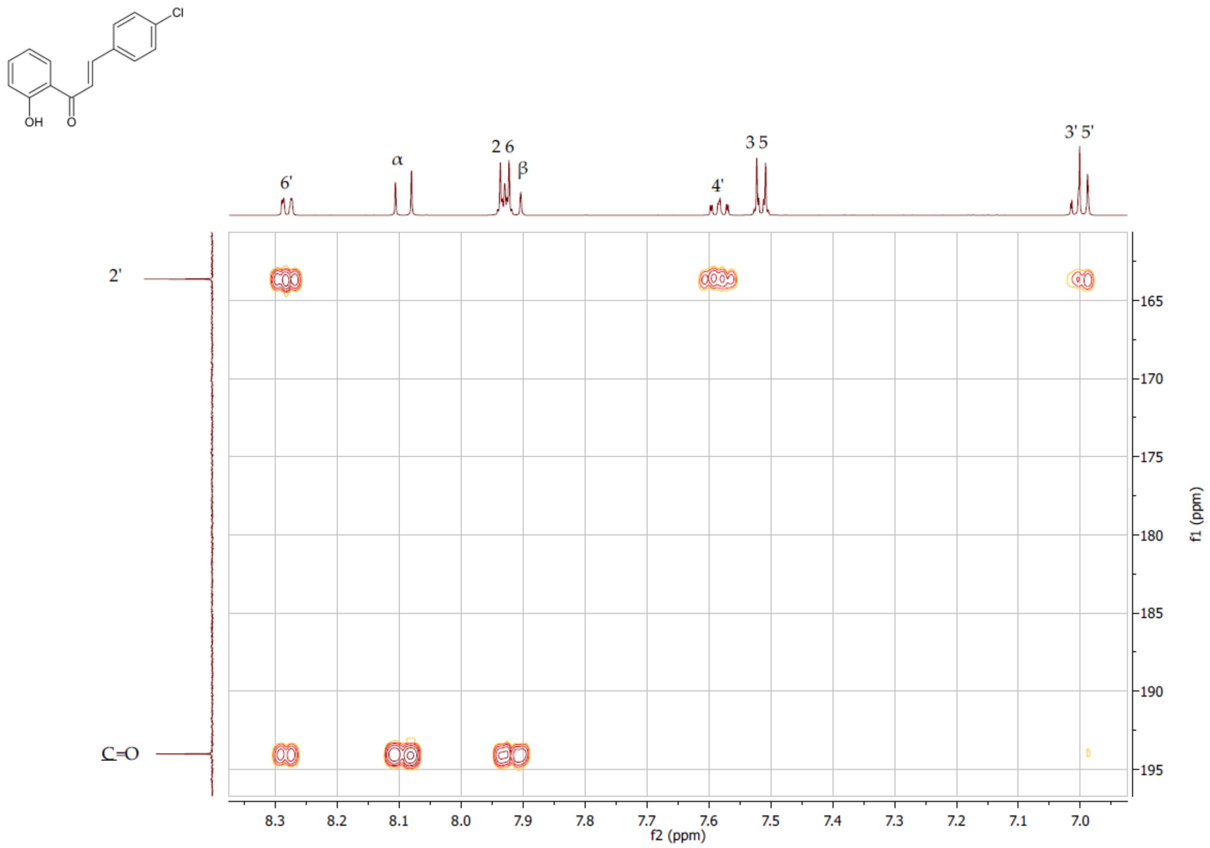

Figure S14. HMBC contour map – ^{1}H x ^{13}C expansion of 4-chloro-2′-hydroxychalcone (**3**)

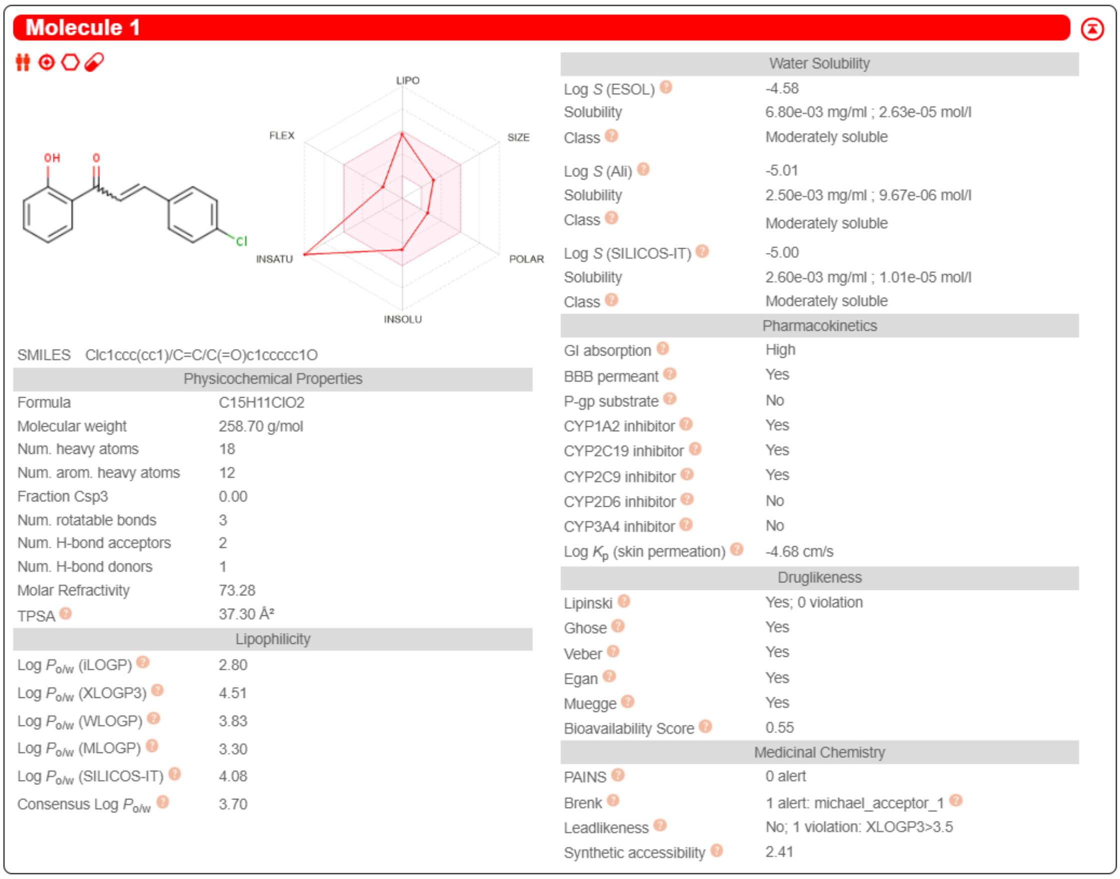

Figure S15. 4-Chloro-2′-hydroxychalcone (**3**) physicochemical and ADME parameters prediction using the SwissADME modelling

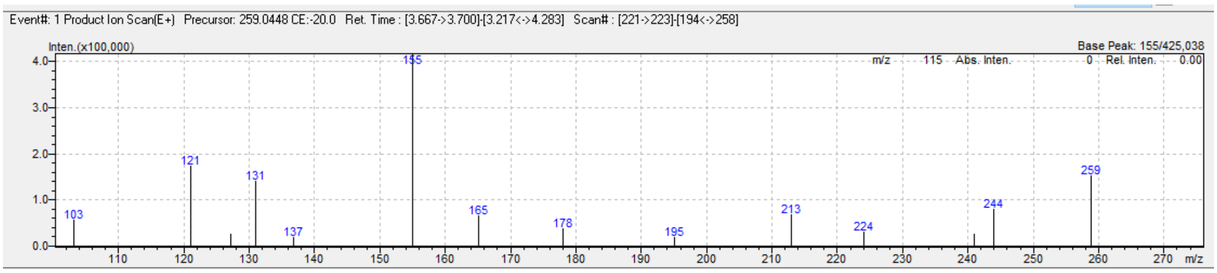

Figure S16. MS analysis of 5′-chloro-2′-hydroxychalcone (**6**)

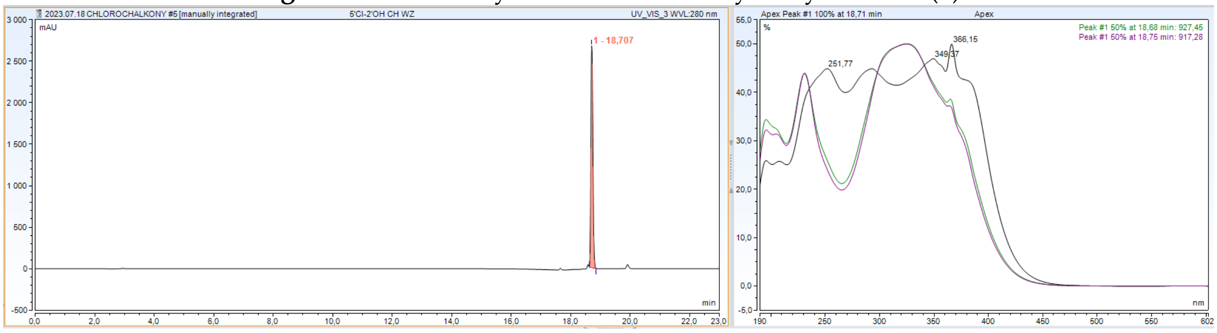

Figure S17. HPLC analysis of 5′-chloro-2′-hydroxychalcone (**6**)

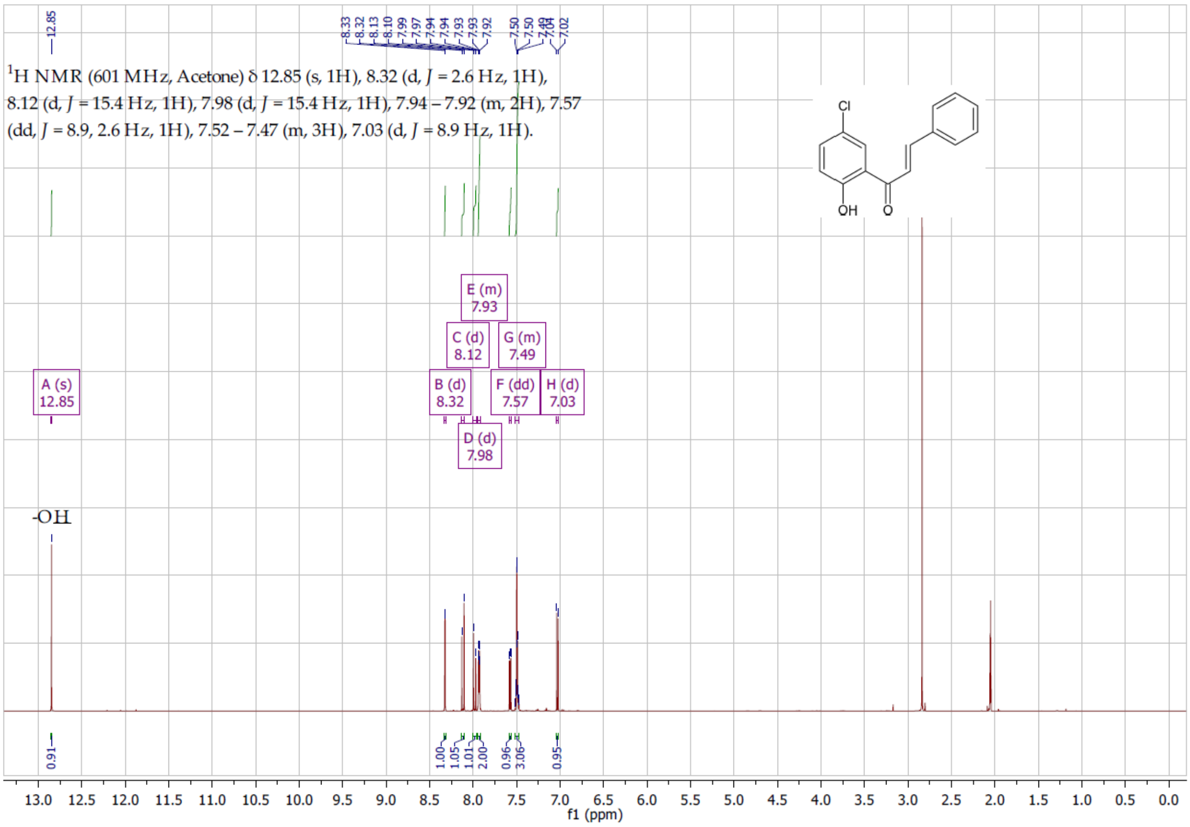

Figure S18. ^{1}H NMR spectrum (δ, acetone-d6, 600 MHz) of 5′-chloro-2′-hydroxychalcone (**6**)

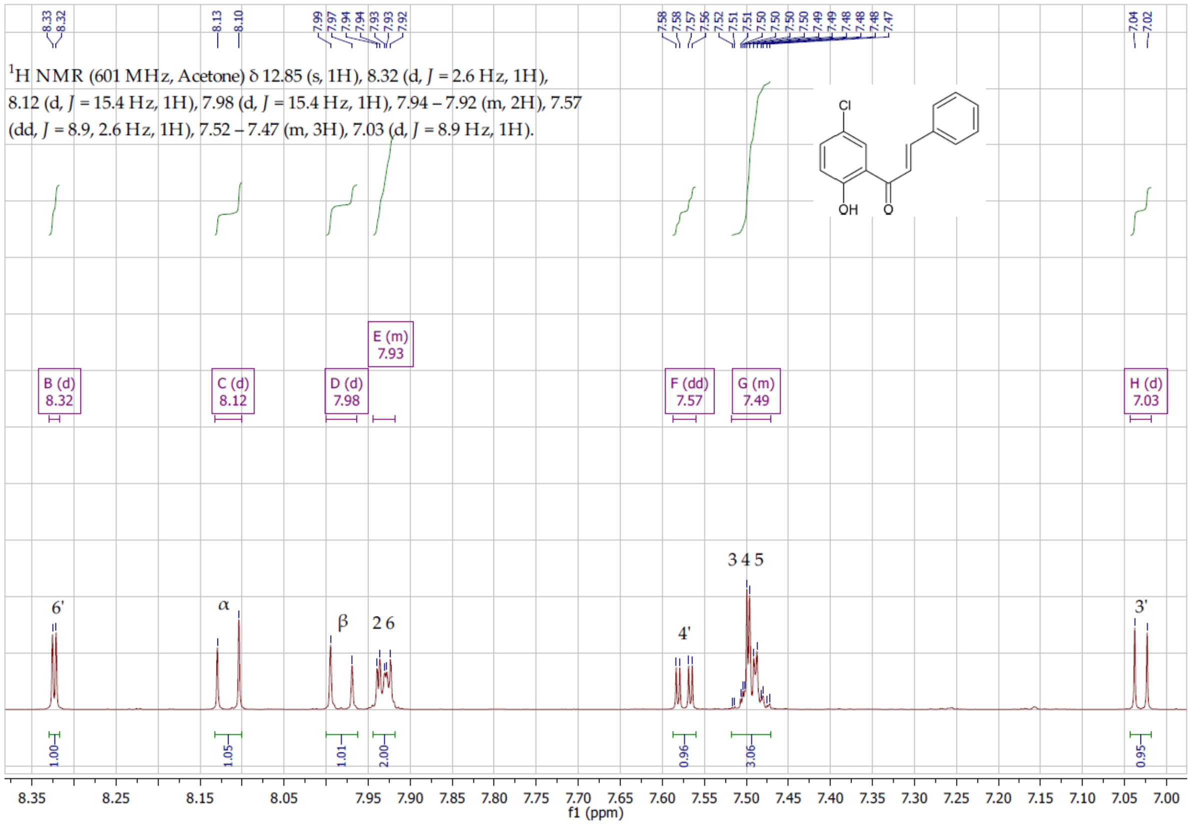

Figure S19. ^{1}H NMR spectrum expansion (*δ*, acetone-d6, 600 MHz) of 5′-chloro-2′-hydroxychalcone (**6**)

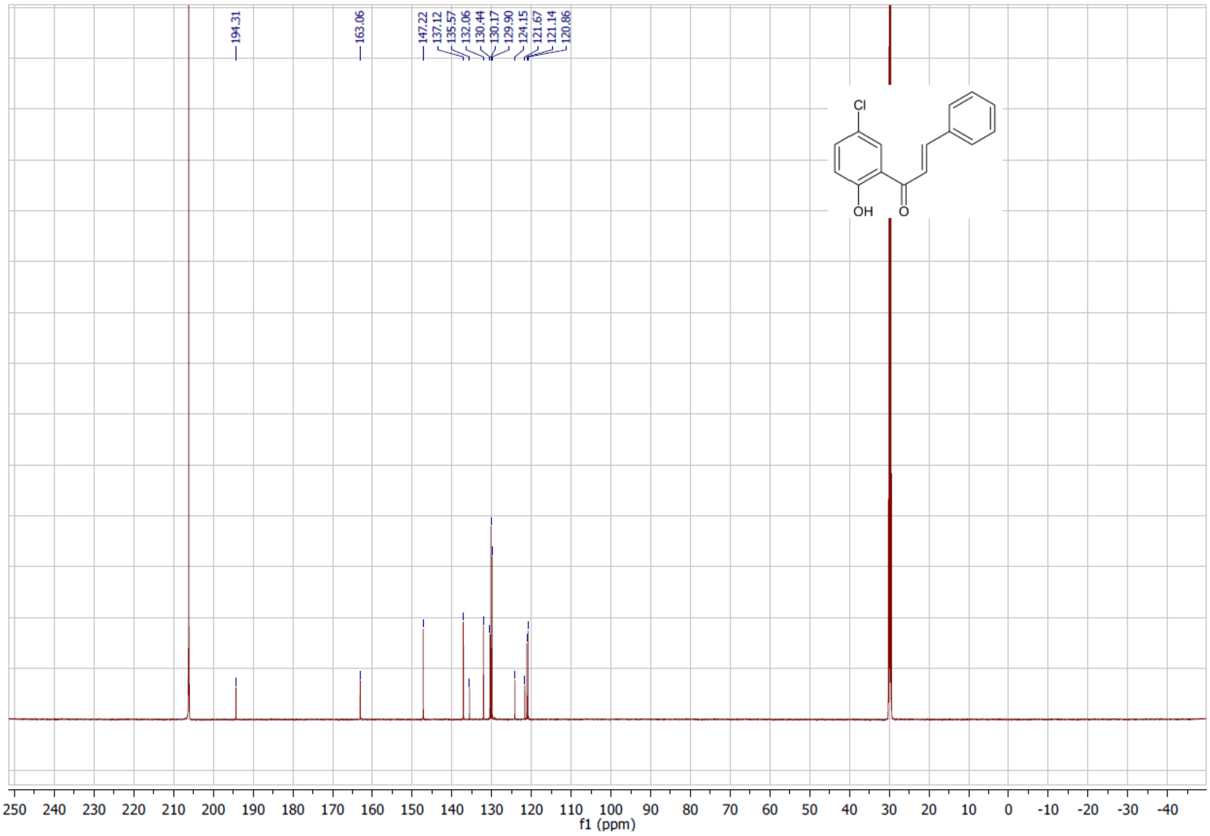

Figure S20. ^{13}C NMR spectrum (*δ*, acetone-d6, 151 MHz) of 5′-chloro-2′-hydroxychalcone (**6**)

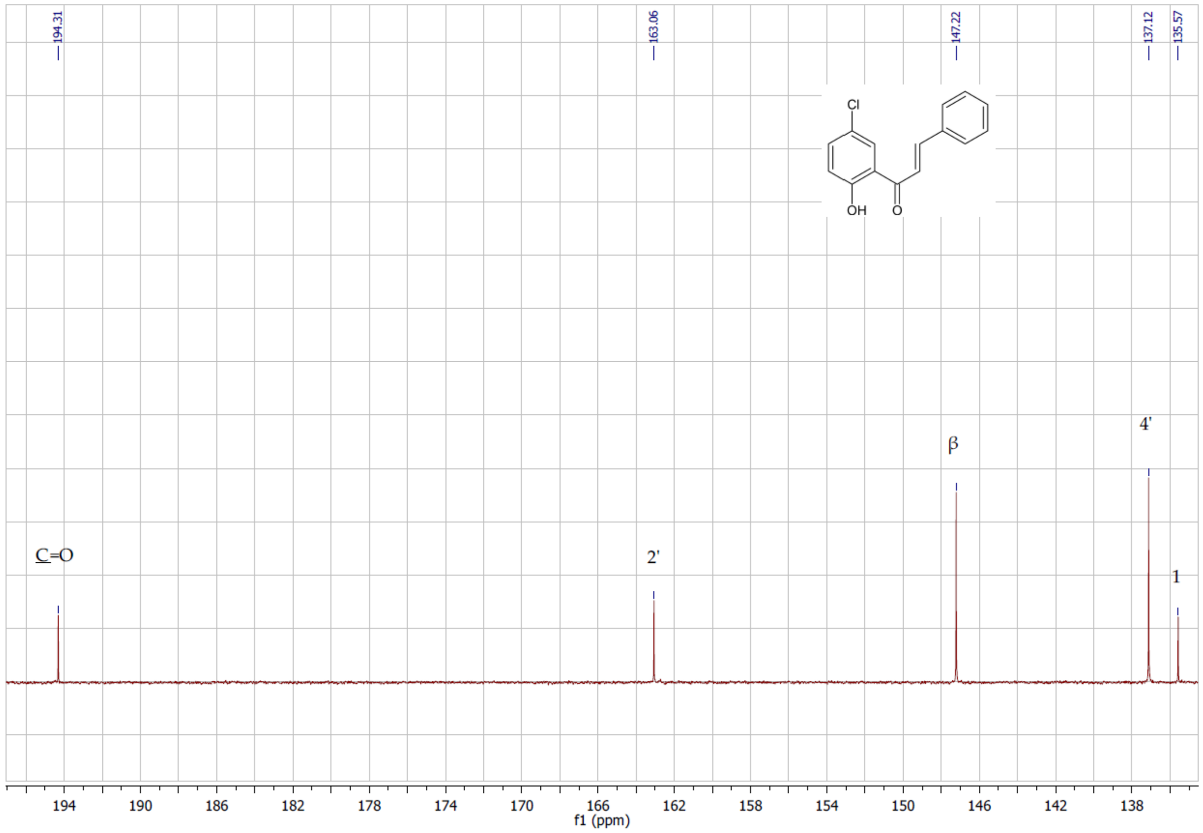

Figure S21. ^{13}C NMR spectrum expansion (δ, acetone-d6, 151 MHz) of 5′-chloro-2′-hydroxychalcone (**6**)

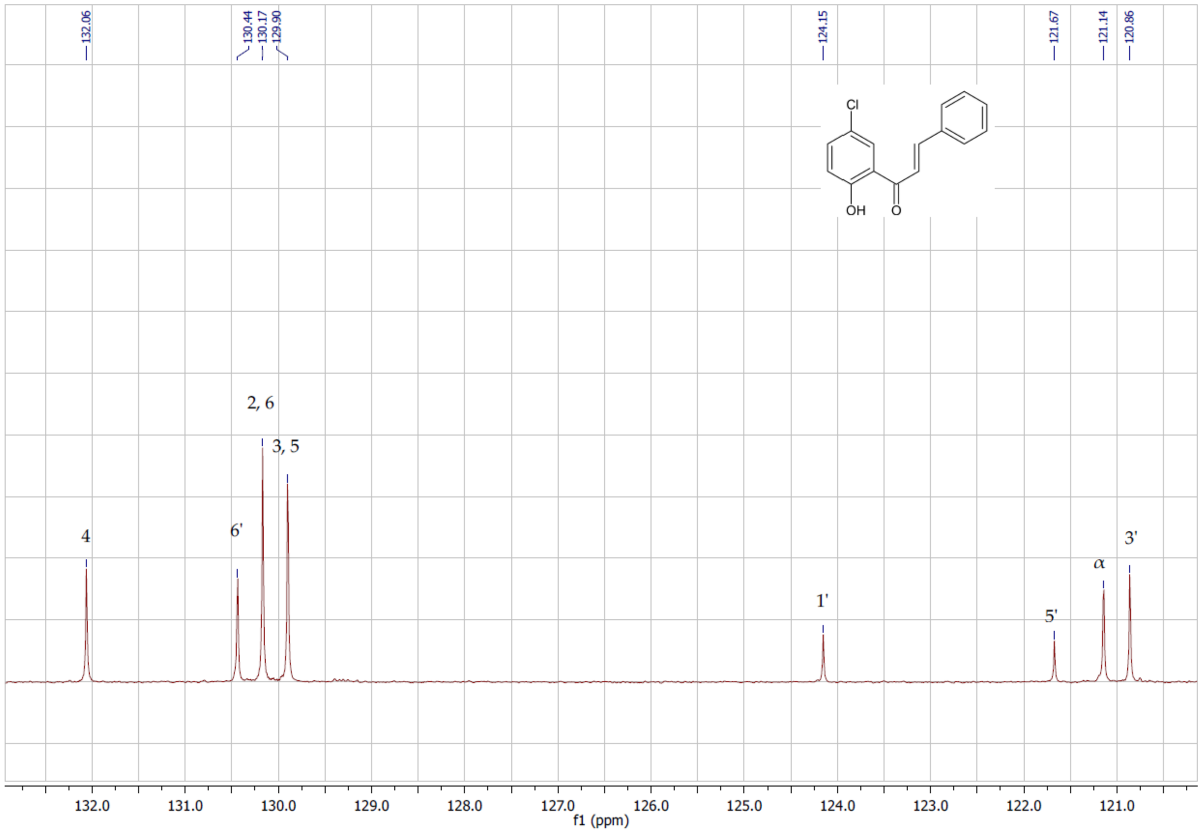

Figure S22. ^{13}C NMR spectrum expansion (δ, acetone-d6, 151 MHz) of 5′-chloro-2′-hydroxychalcone (**6**)

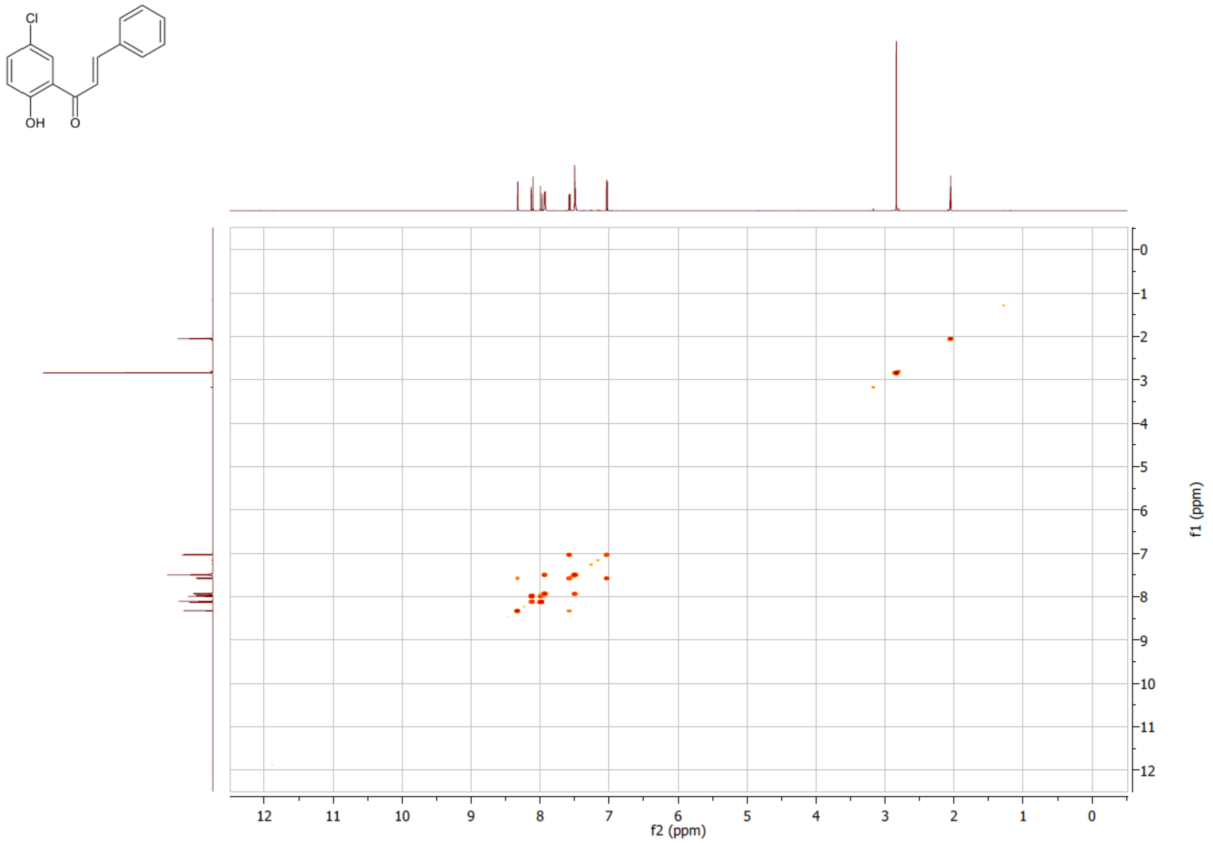

Figure S23. COSY contour map – ^{1}H x ^{1}H of 5′-chloro-2′-hydroxychalcone (**6**)

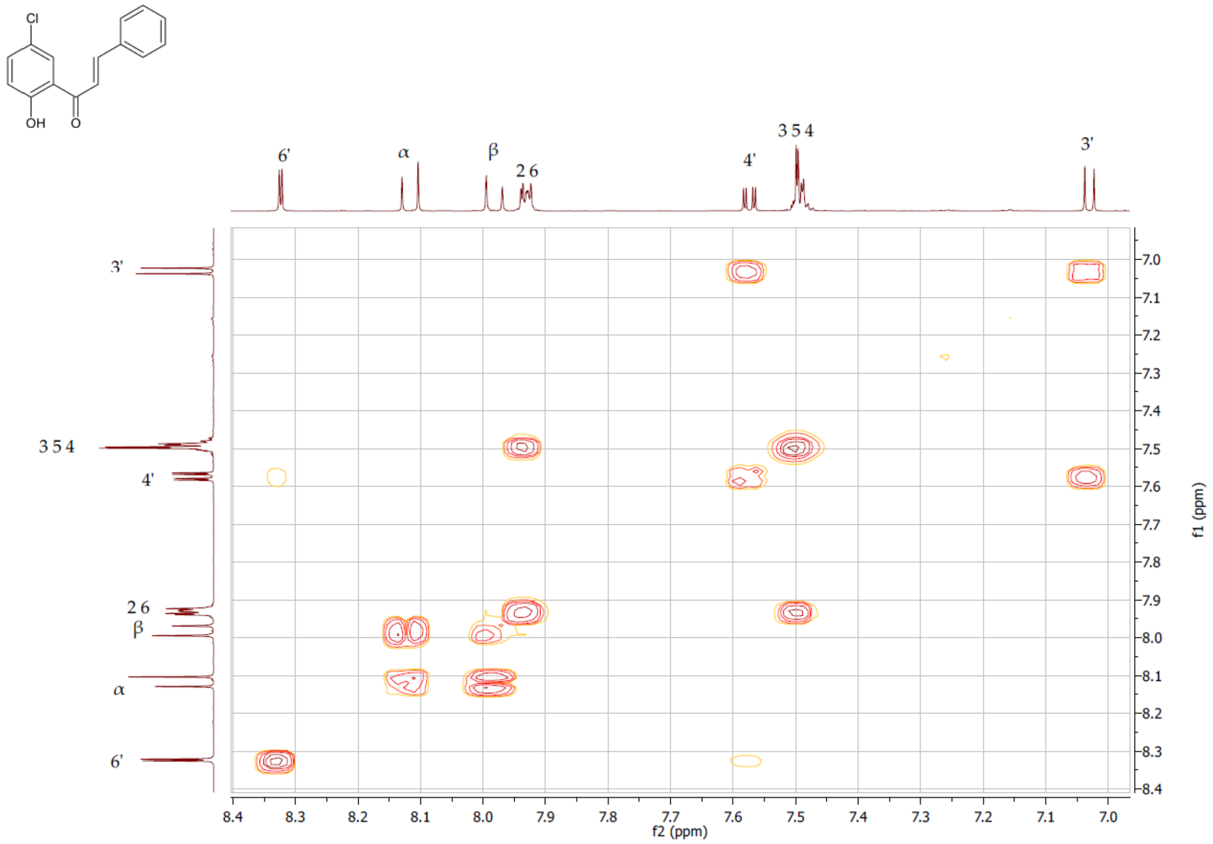

Figure S24. COSY contour map – ^{1}H x ^{1}H expansion of 5′-chloro-2′-hydroxychalcone (**6**)

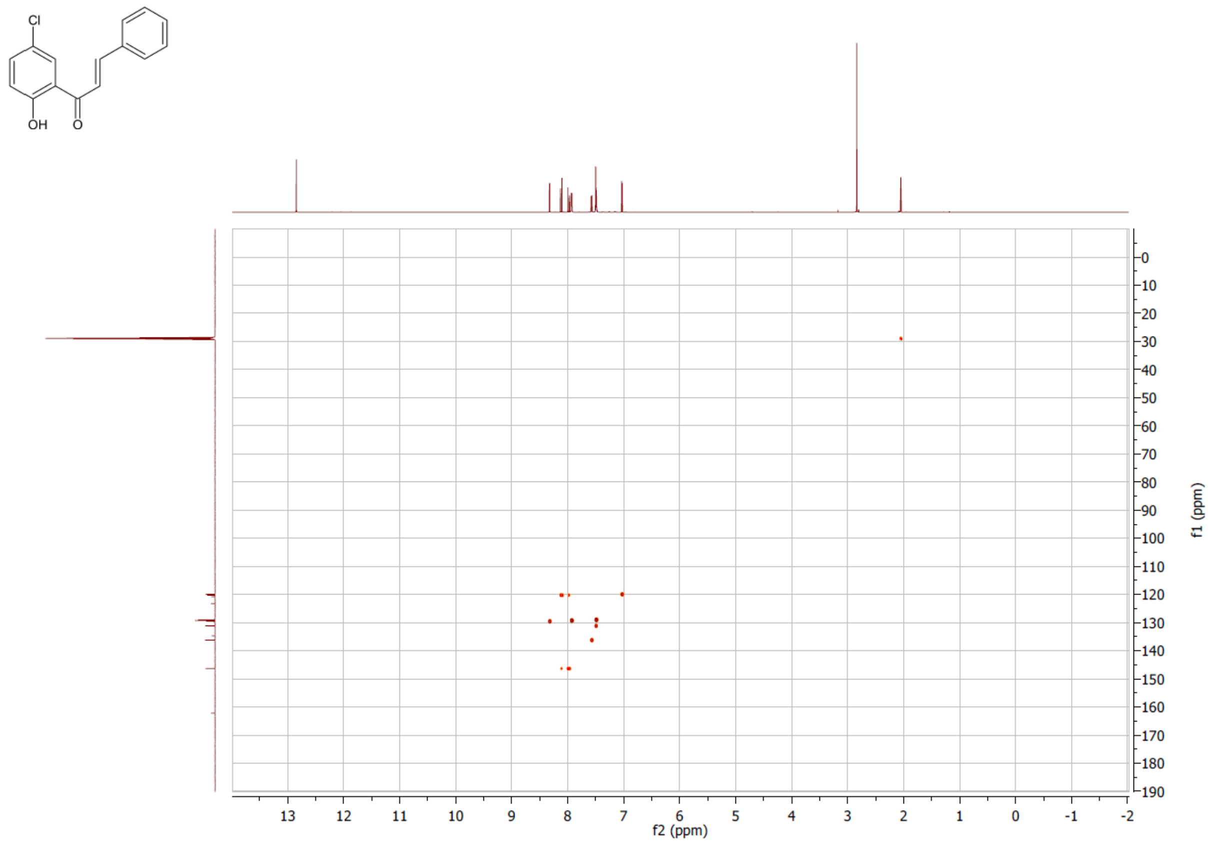

Figure S25. HMQC contour map – ^{1}H x ^{13}C of 5′-chloro-2′-hydroxychalcone (**6**)

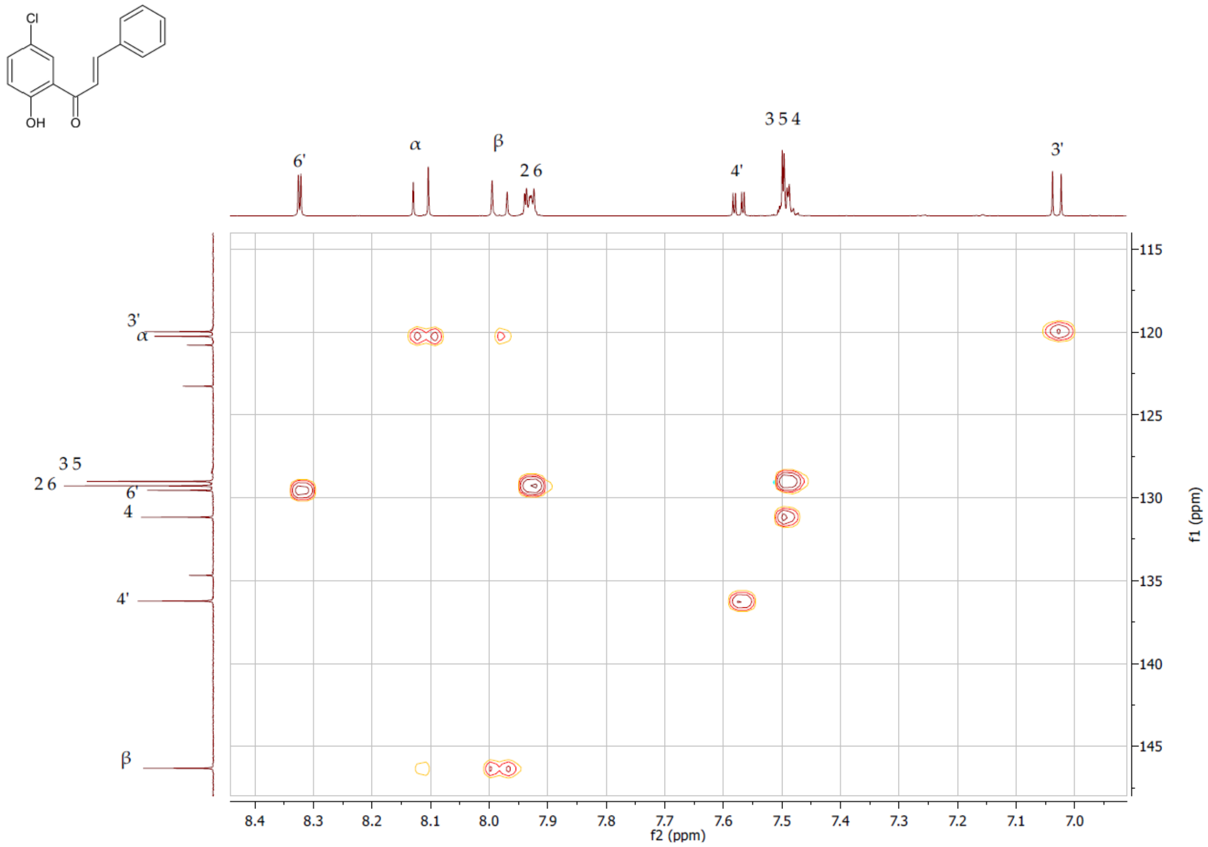

Figure S26. HMQC contour map – ^{1}H x ^{13}C expansion of 5′-chloro-2′-hydroxychalcone (**6**)

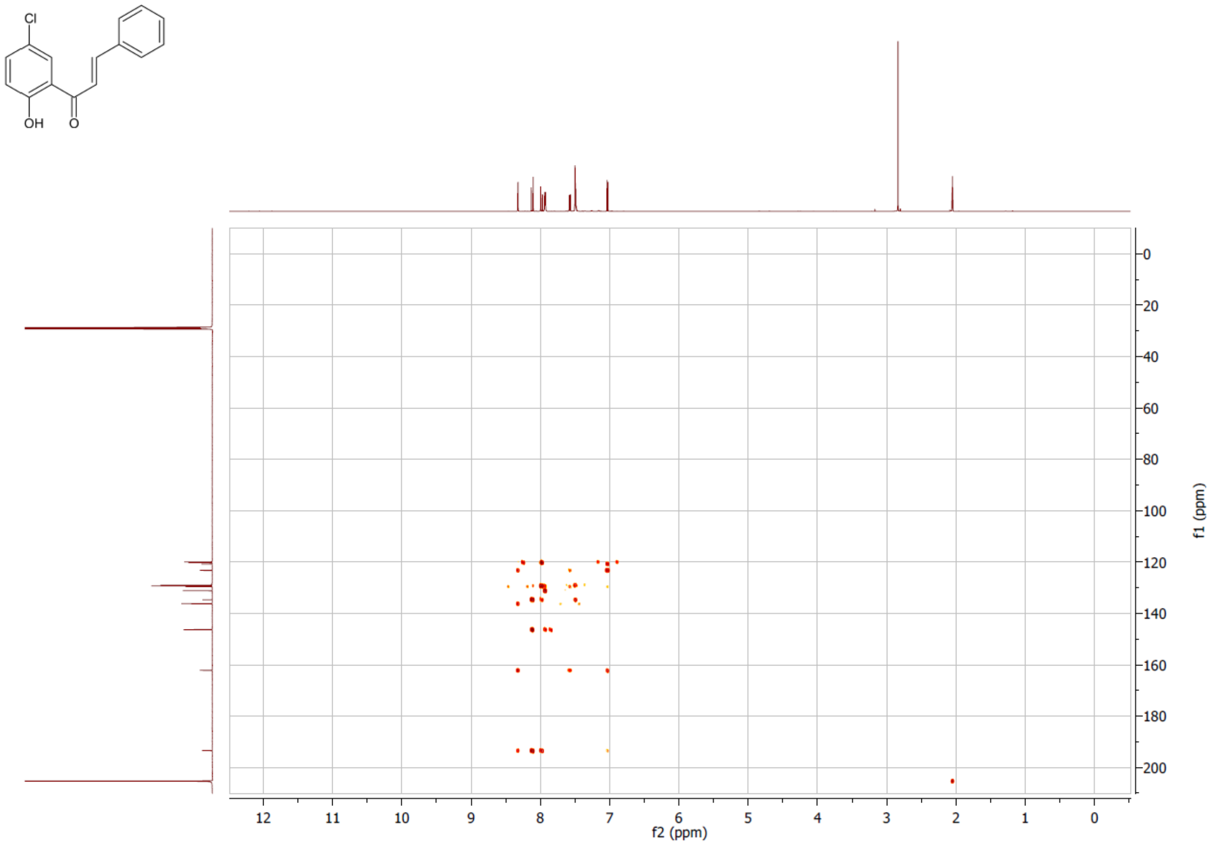

Figure S27. HMBC contour map – ^{1}H x ^{13}C of 5′-chloro-2′-hydroxychalcone (**6**)

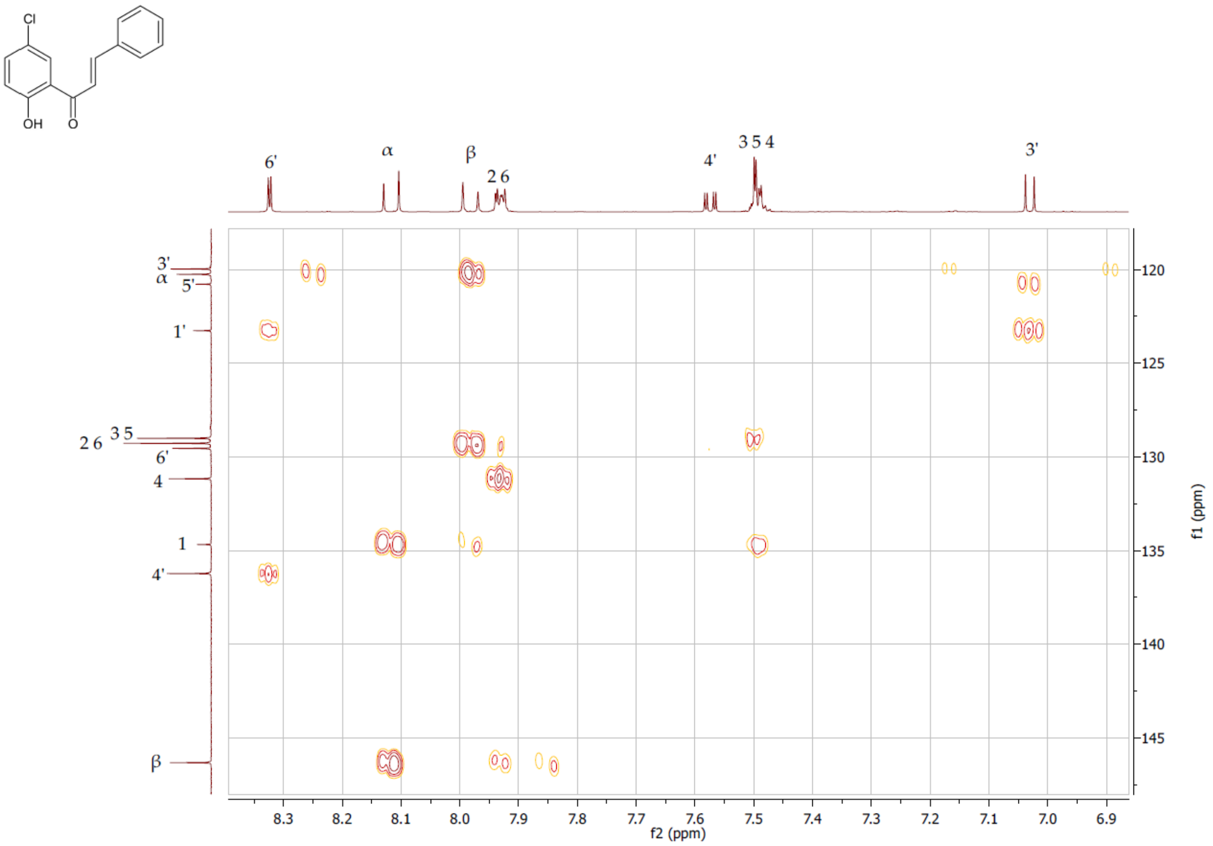

Figure S28. HMBC contour map – ^{1}H x ^{13}C expansion of 5′-chloro-2′-hydroxychalcone (**6**)

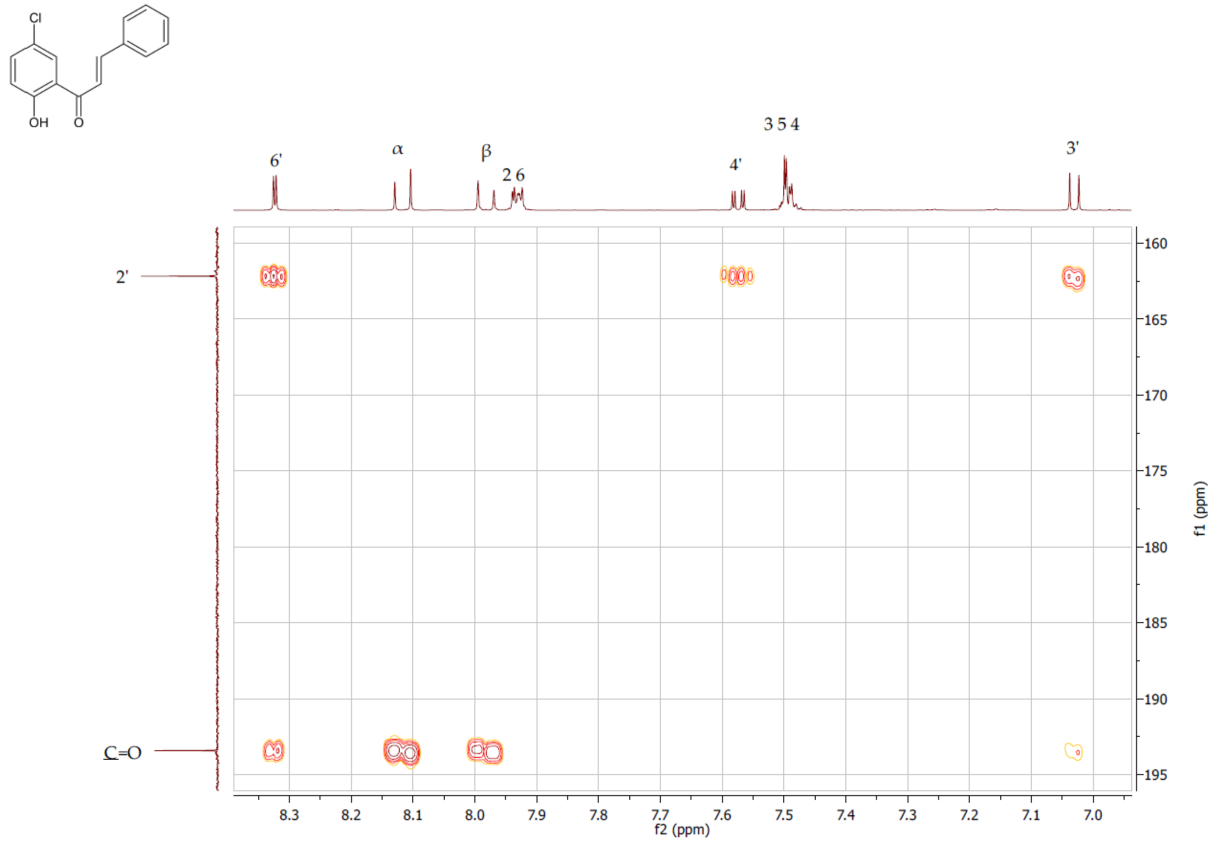

Figure S29. HMBC contour map – ^{1}H x ^{13}C expansion of 5′-chloro-2′-hydroxychalcone (**6**)

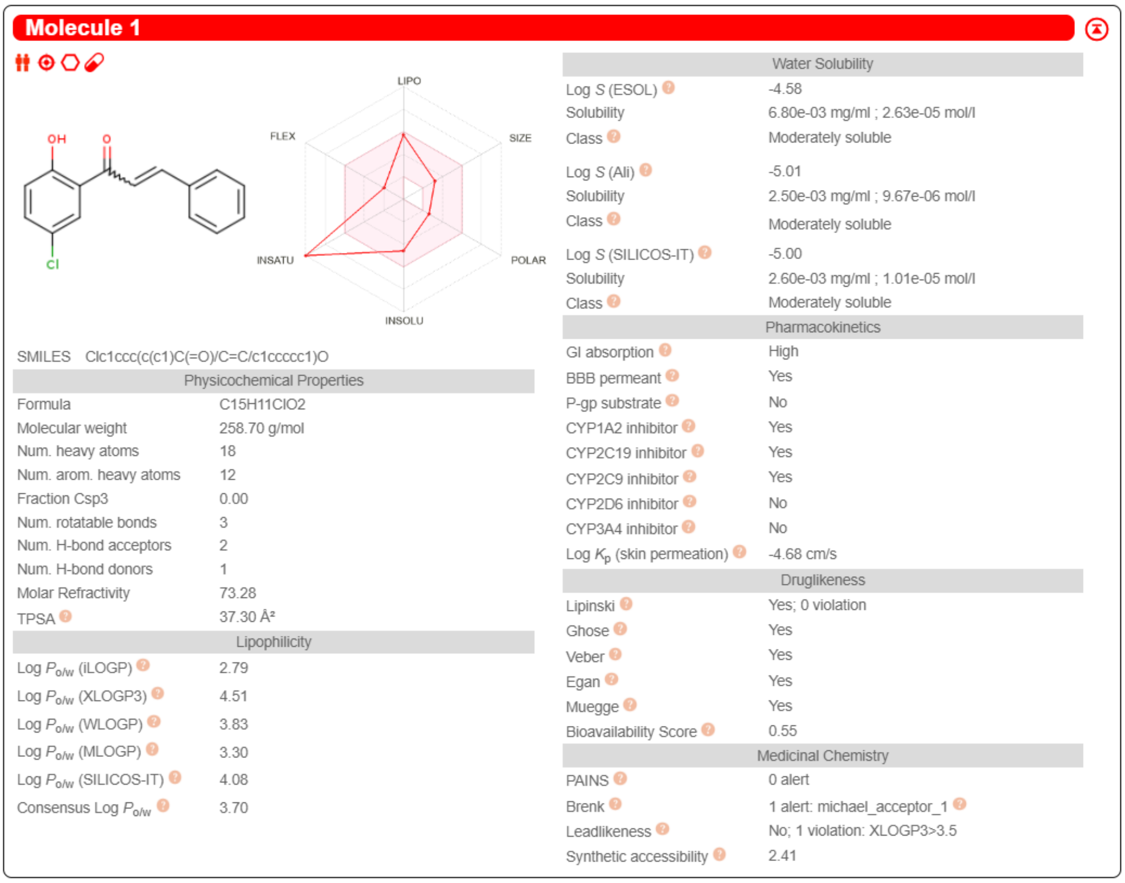

Figure S30. 5′-Chloro-2′-hydroxychalcone (**6**) physicochemical and ADME parameters prediction using the SwissADME modelling

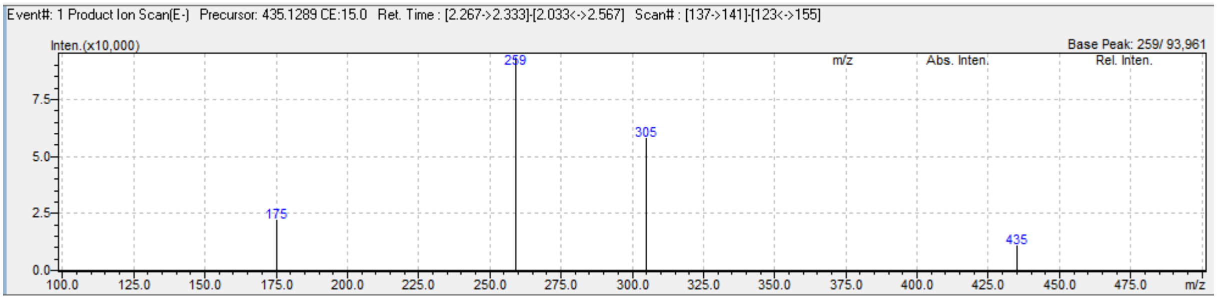

Figure S31. MS analysis of 4-chlorodihydrochalcone 2′-*O*-*β*-D-(4′′-*O*-methyl)-glucopyranoside (**3a**)

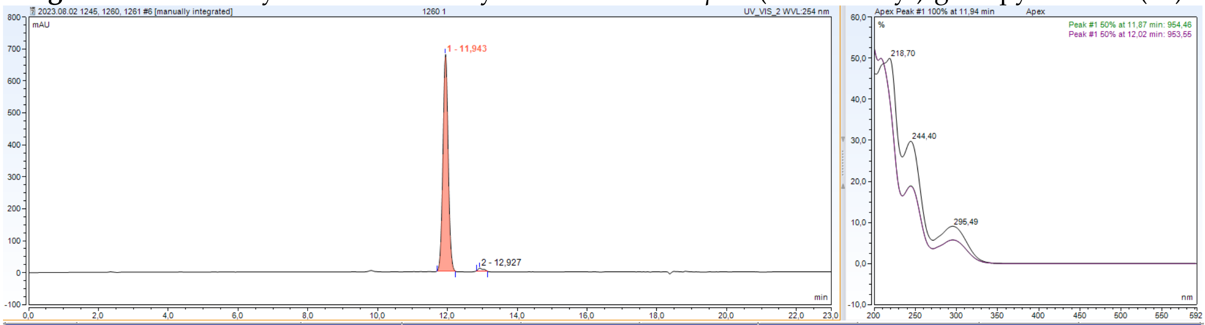

Peak No.	Peak Name	Ret.Time min	Rel.Area %	Area mAU*min	Height mAU	Match	RSD Match %	PPI nm	RSD PPI %
1		*11,943*	*98,63*	*117,2608*	*679,23*	*955*	*3,88*	*216,7*	*1,58*
2		12,927	1,37	1,6334	8,95	993	0,33	212,2	0,41
Maximum			98,63	117,2608	679,23	993	3,88		1,58
Minimum			1,37	1,6334	8,95	955	0,33		0,41

Figure S32. HPLC analysis of 4-chlorodihydrochalcone 2′-*O*-*β*-D-(4′′-*O*-methyl)-glucopyranoside (**3a**)

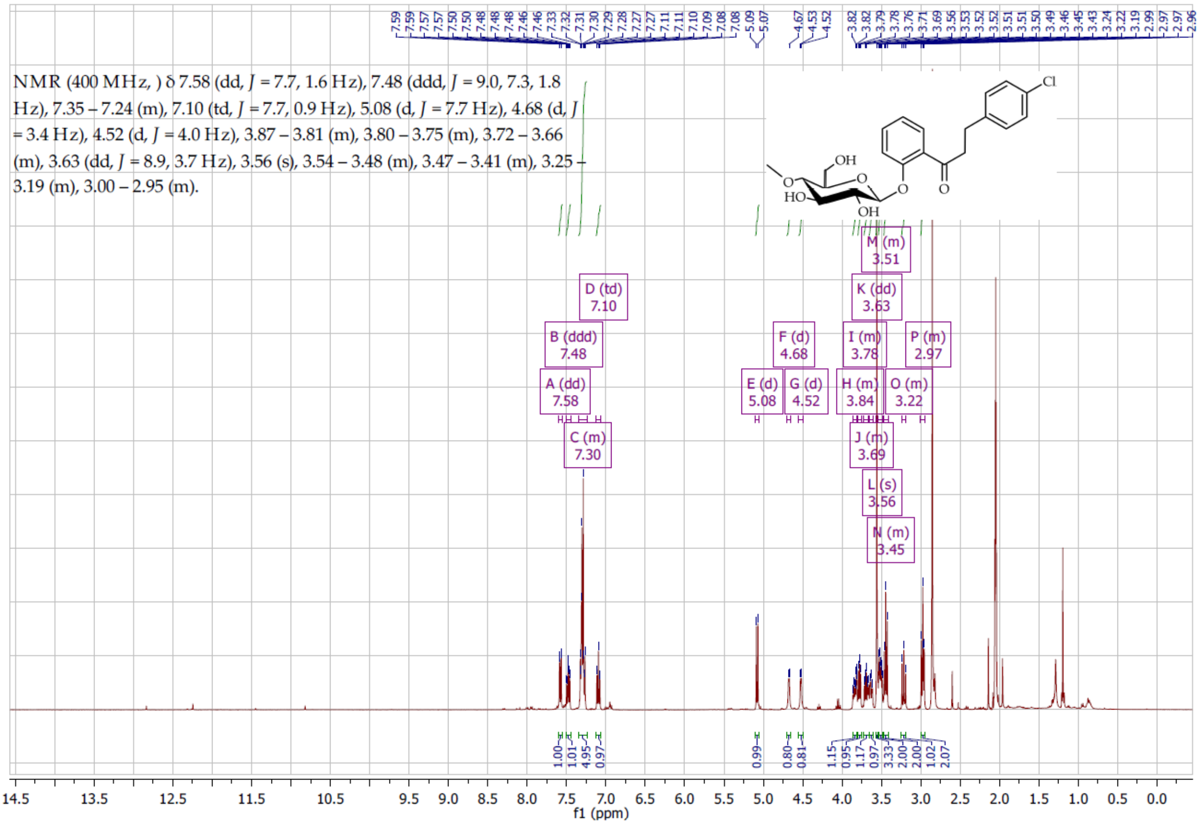

Figure S33. ^{1}H NMR spectrum (*δ*, acetone-d6, 600 MHz) of 4-chlorodihydrochalcone 2′-*O*-*β*-D-(4′′-*O*-methyl)-glucopyranoside (**3a**)

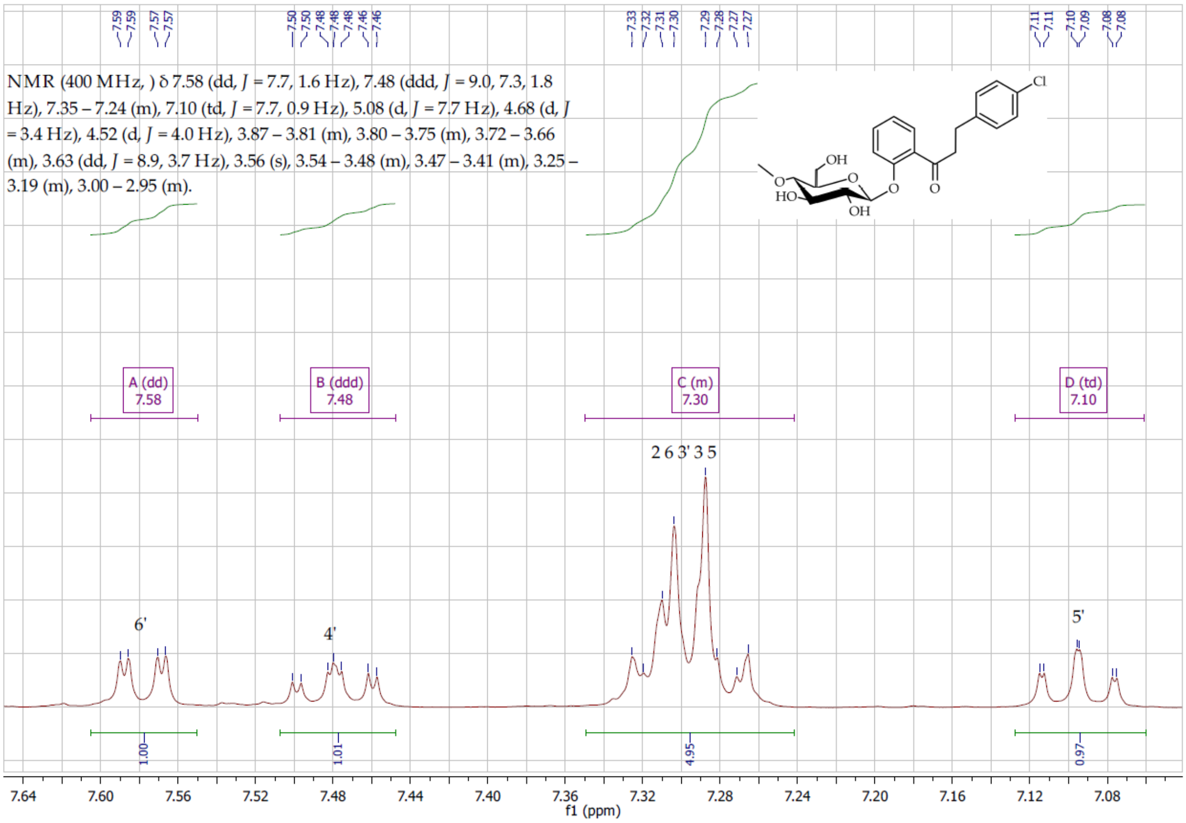

Figure S34. ^{1}H NMR spectrum expansion (δ, acetone-d6, 600 MHz) of 4-chlorodihydrochalcone 2′-*O*-*β*-D-(4′′-*O*-methyl)-glucopyranoside (**3a**)

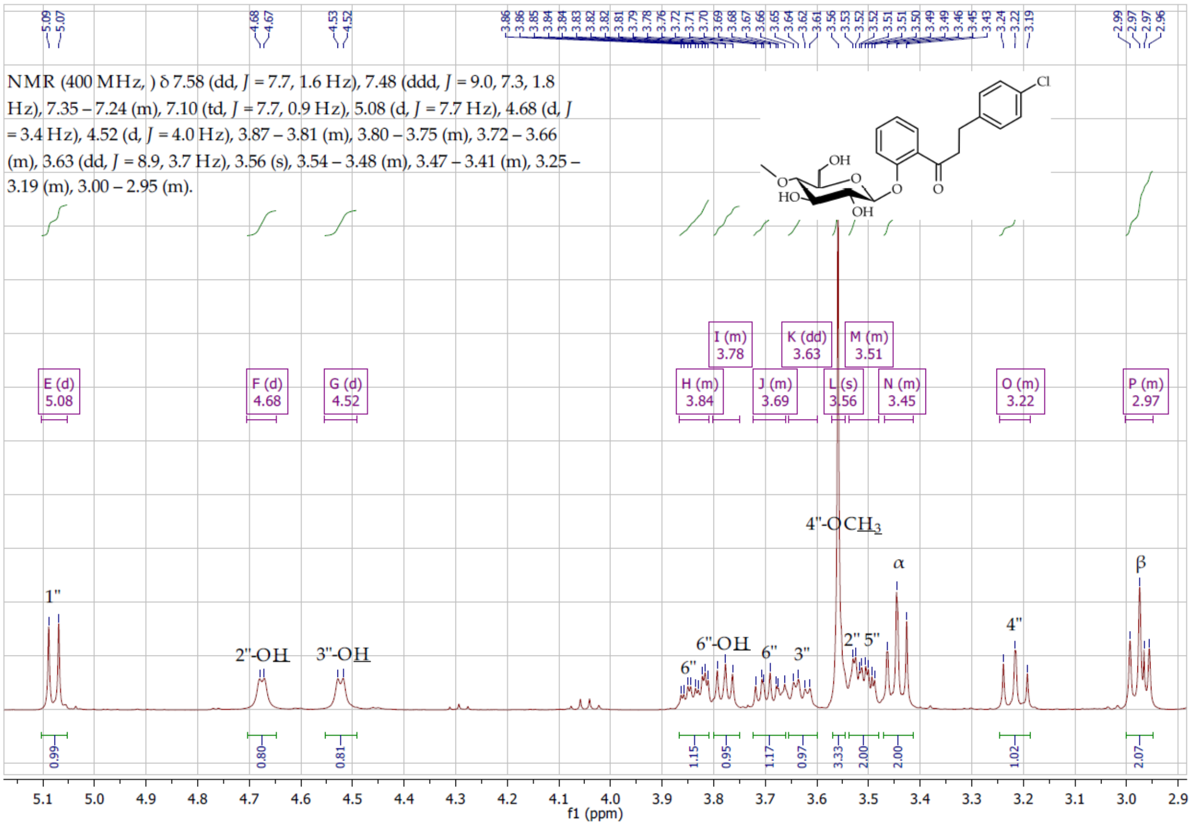

Figure S35. ^{1}H NMR spectrum expansion (δ, acetone-d6, 600 MHz) of 4-chlorodihydrochalcone 2′-*O*-*β*-D-(4′′-*O*-methyl)-glucopyranoside (**3a**)

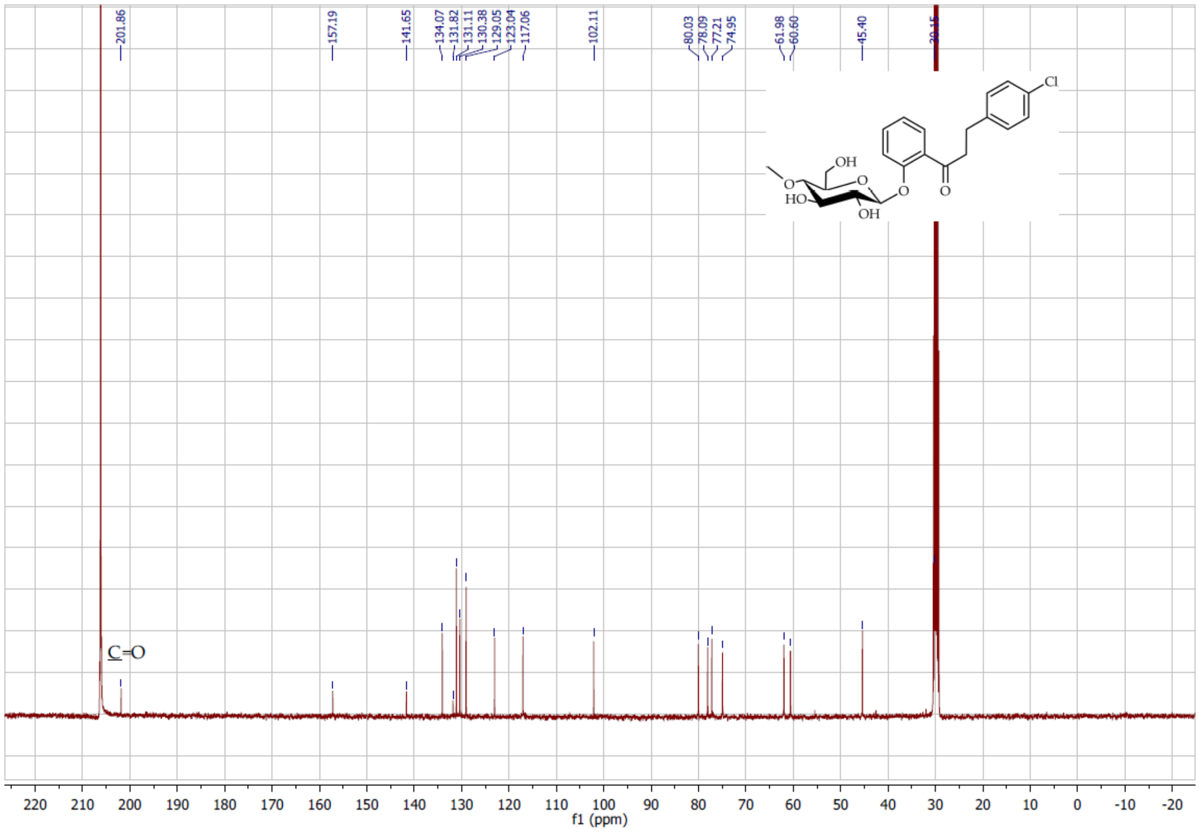

Figure S36. ^{13}C NMR spectrum (*δ*, acetone-d6, 151 MHz) of 4-chlorodihydrochalcone 2′-*O*-*β*-D-(4′′-*O*-methyl)-glucopyranoside (**3a**)

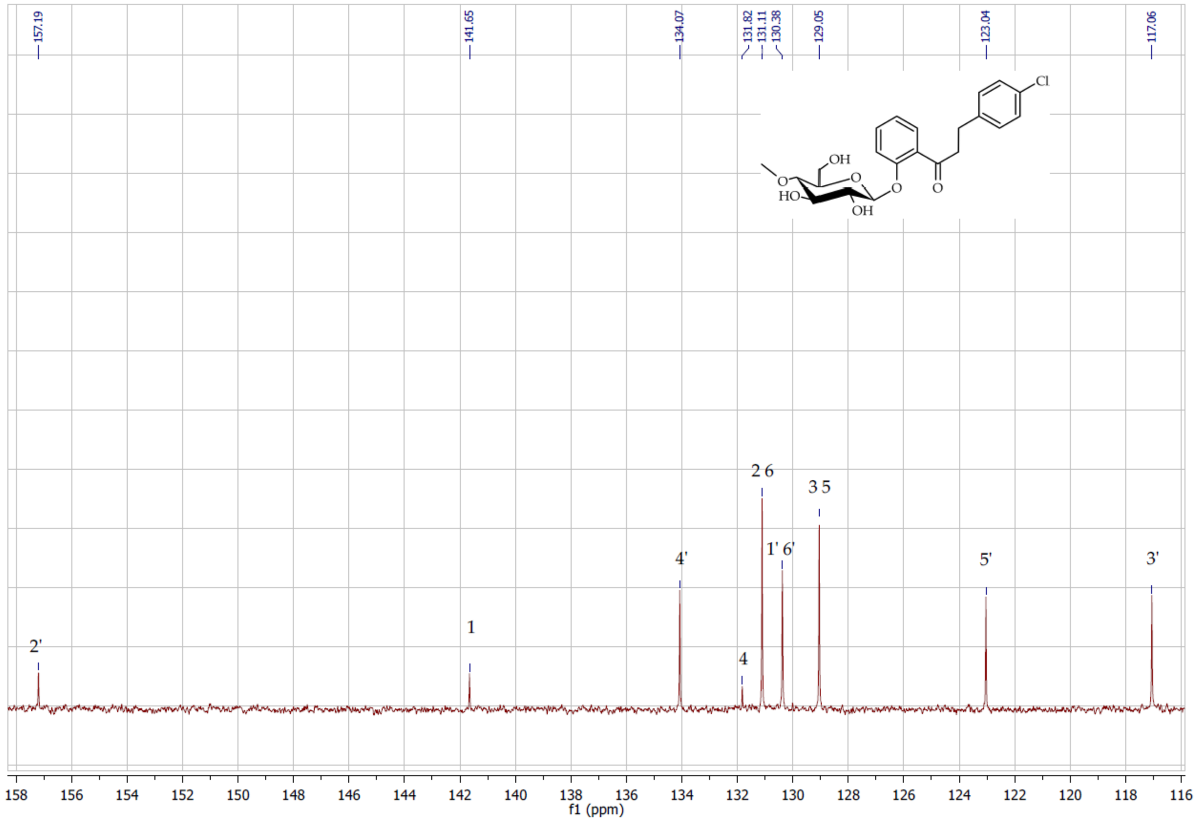

Figure S37. ^{13}C NMR spectrum expansion (*δ*, acetone-d6, 151 MHz) of 4-chlorodihydrochalcone 2′-*O*-*β*-D-(4′′-*O*-methyl)-glucopyranoside (**3a**)

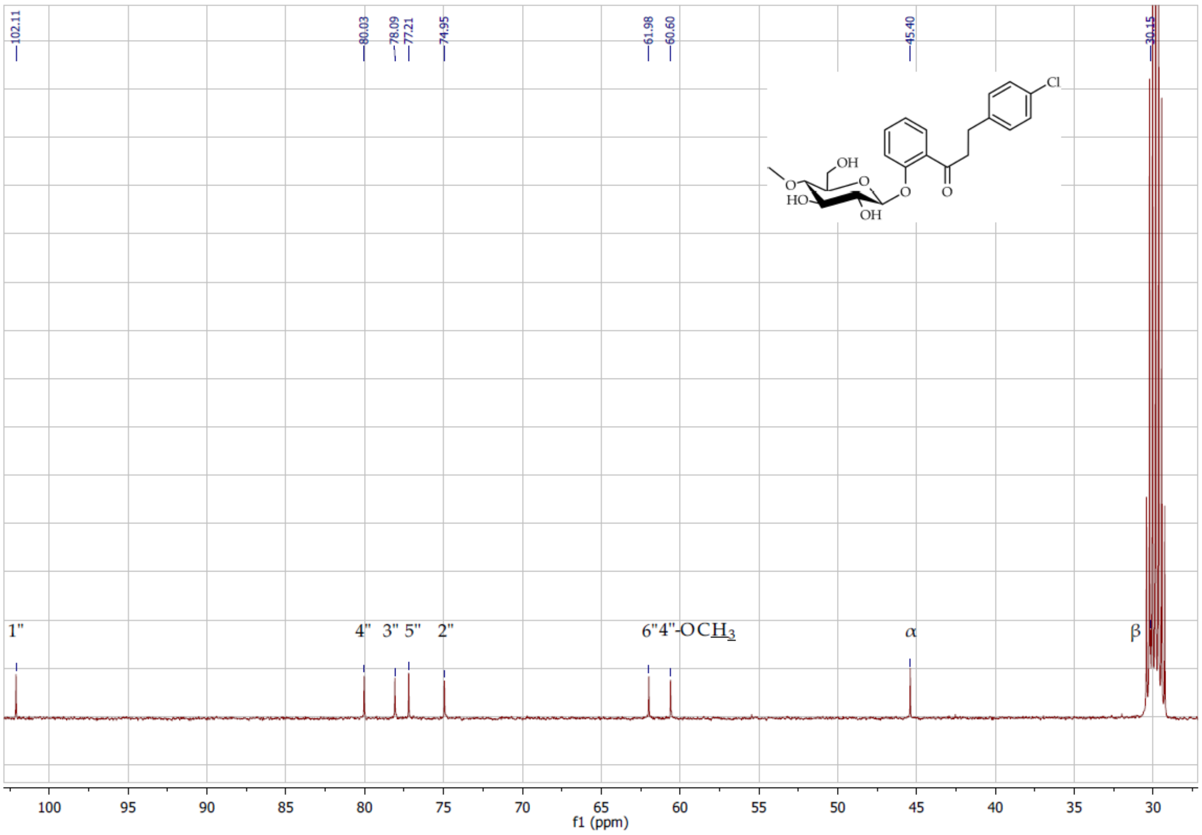

Figure S38. ^{13}C NMR spectrum expansion (δ, acetone-d6, 151 MHz) of 4-chlorodihydrochalcone 2′-*O*-*β*-D-(4′′-*O*-methyl)-glucopyranoside (**3a**)

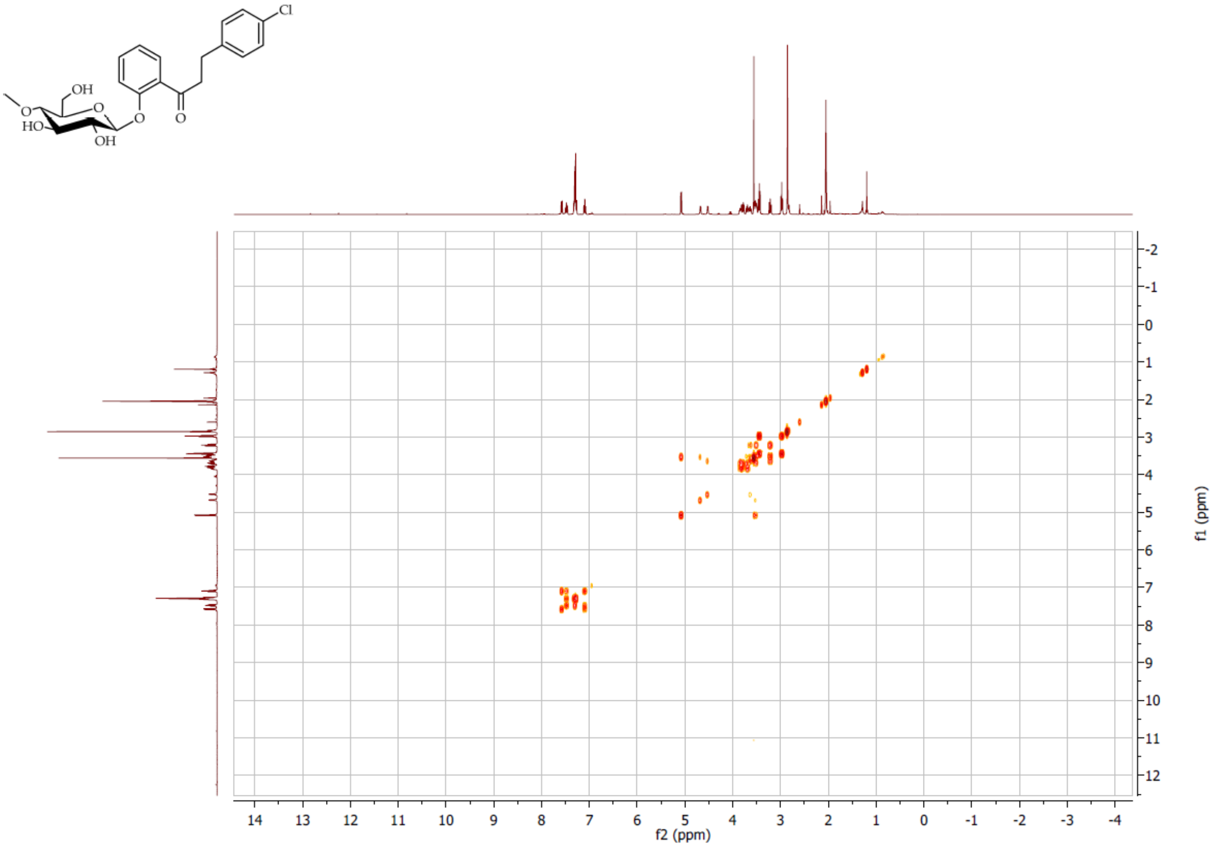

Figure S39. COSY contour map – ^{1}H x ^{1}H of 4-chlorodihydrochalcone 2′-*O*-*β*-D-(4′′-*O*-methyl)-glucopyranoside (**3a**)

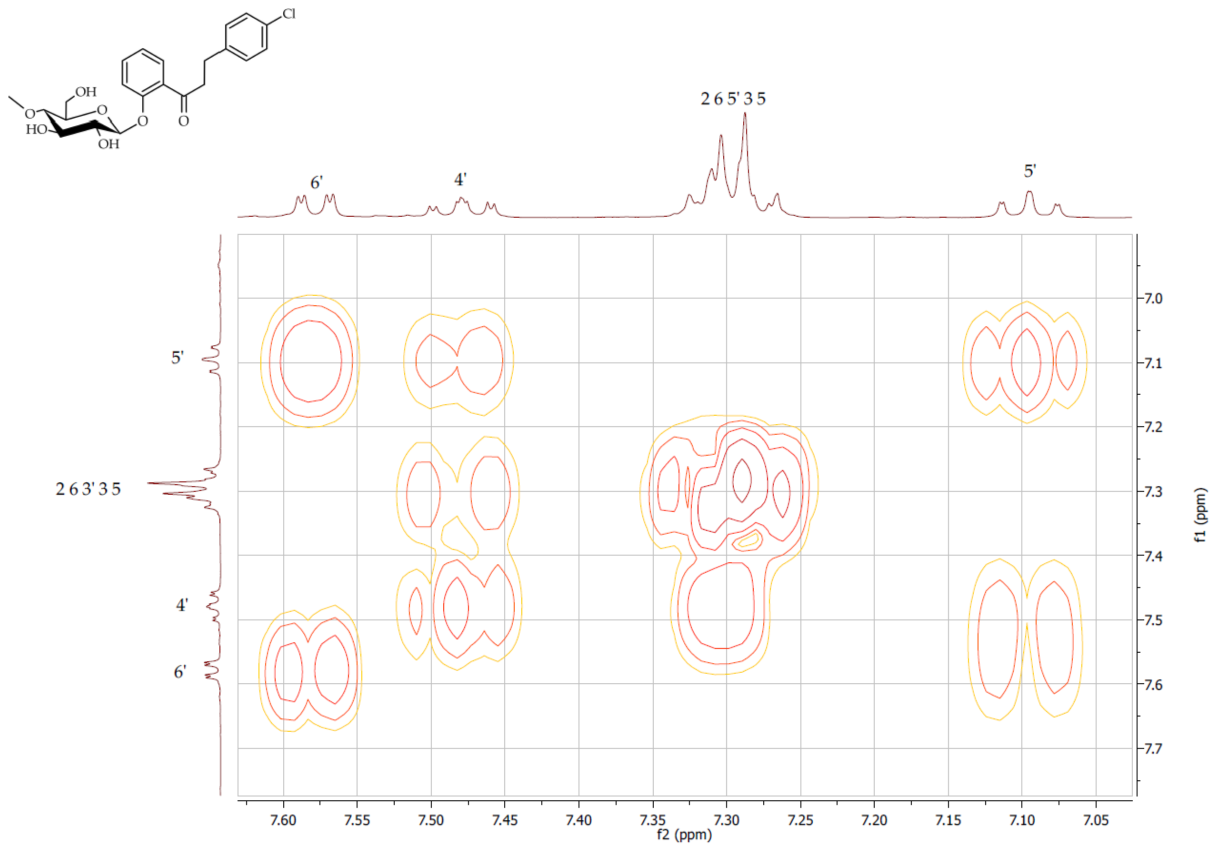

Figure S40. COSY contour map – ¹H x ¹H expansion of 4-chlorodihydrochalcone 2′-*O*-*β*-D-(4′′-*O*-methyl)-glucopyranoside (**3a**)

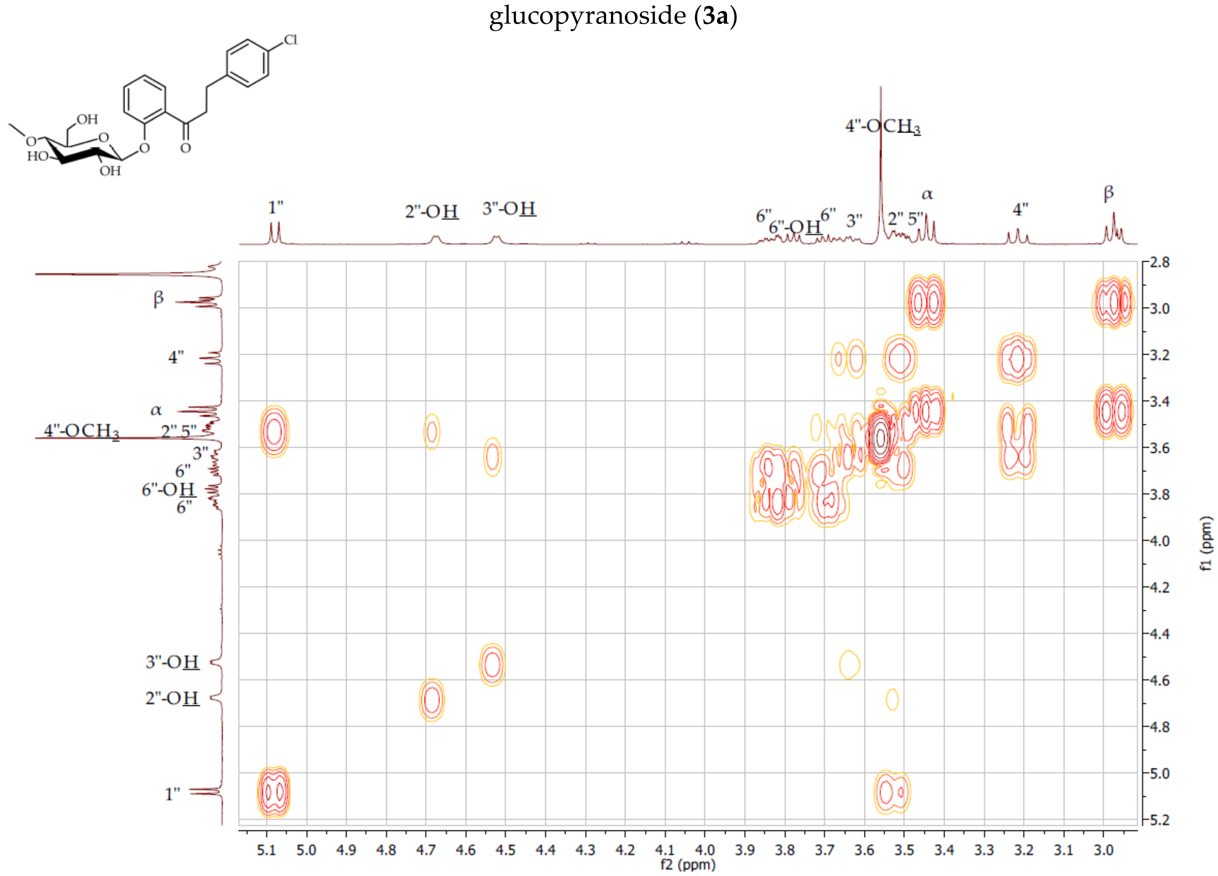

Figure S41. COSY contour map – ¹H x ¹H expansion of 4-chlorodihydrochalcone 2′-*O*-*β*-D-(4′′-*O*-methyl)-glucopyranoside (**3a**)

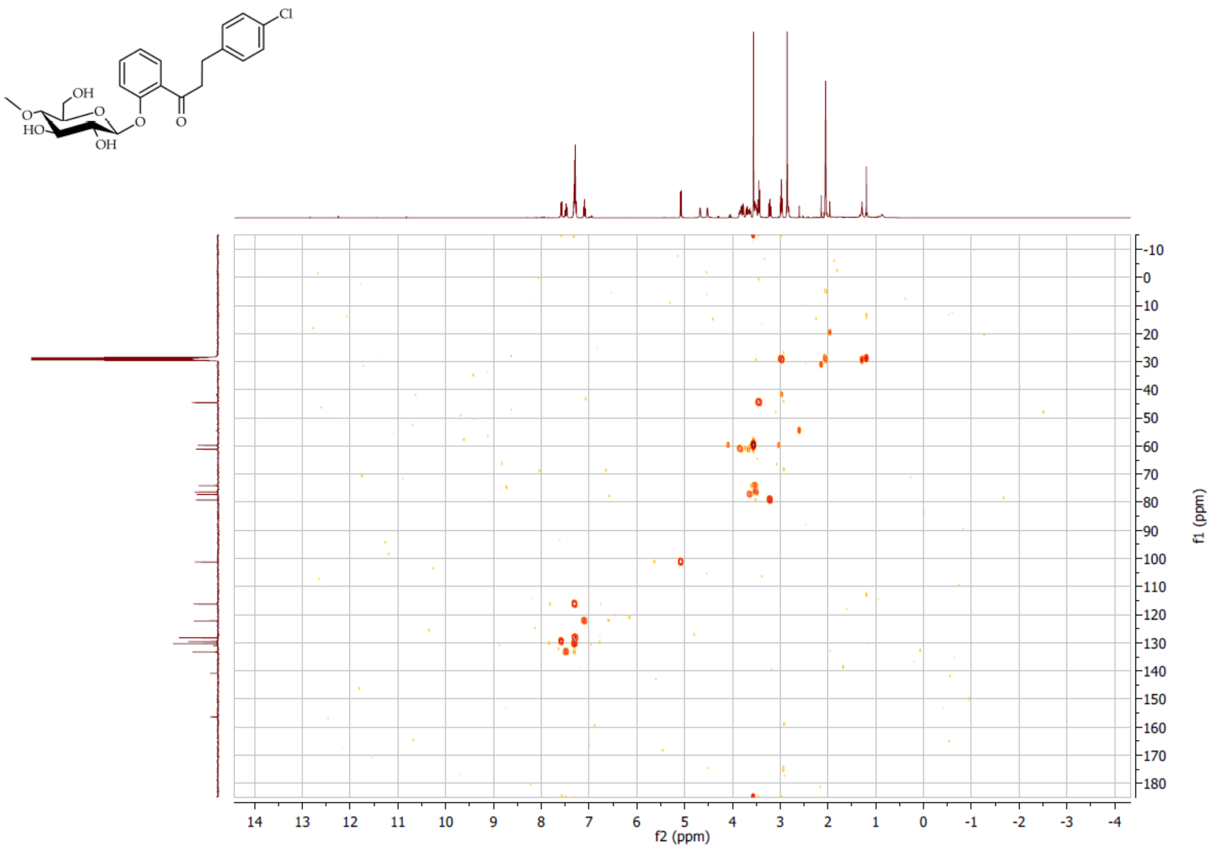

Figure S42. HMQC contour map – ^{1}H x ^{13}C of 4-chlorodihydrochalcone 2′-*O*-*β*-D-(4′′-*O*-methyl)-glucopyranoside (**3a**)

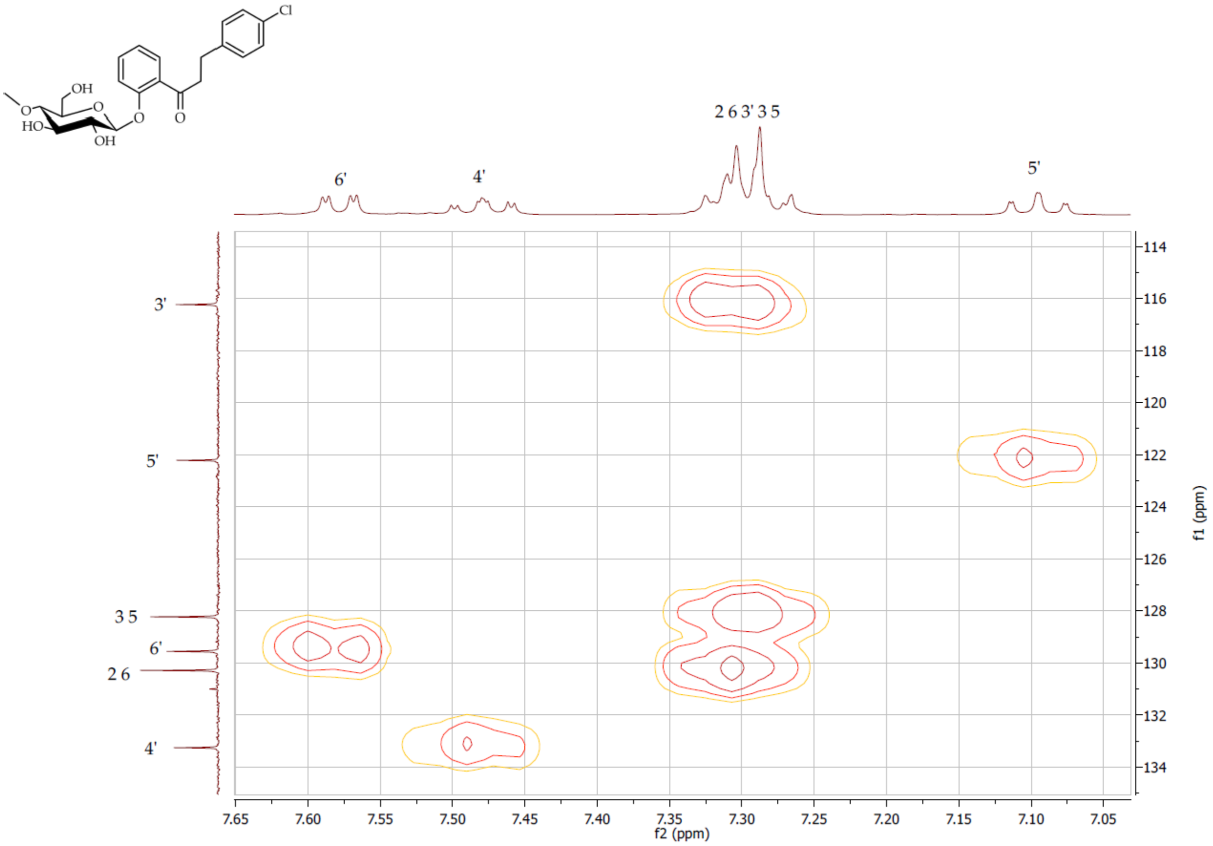

Figure S43. HMQC contour map – ^{1}H x ^{13}C expansion of 4-chlorodihydrochalcone 2′-*O*-*β*-D-(4′′-*O*-methyl)-glucopyranoside (**3a**)

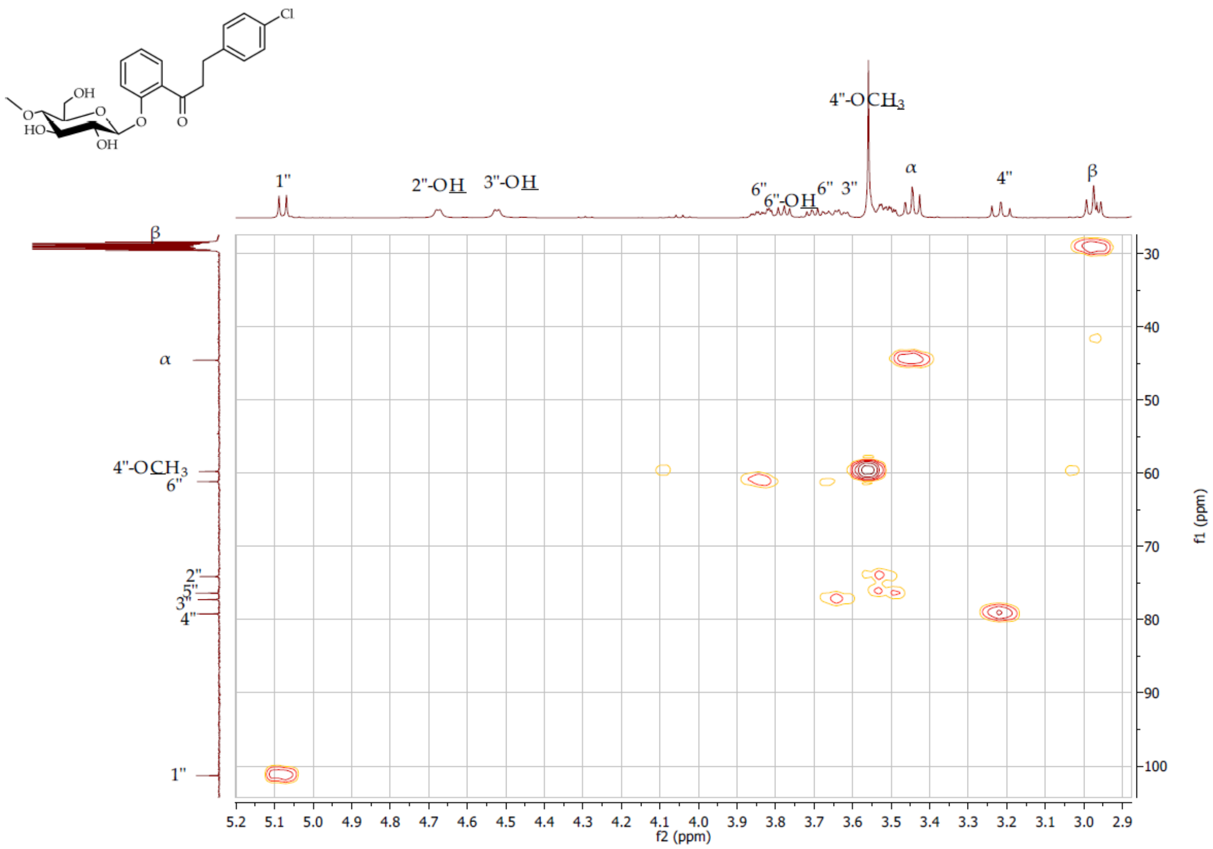

Figure S44. HMQC contour map – [1]H x ^{13}C expansion of 4-chlorodihydrochalcone 2′-*O*-*β*-D-(4′′-*O*-methyl)-glucopyranoside (**3a**)

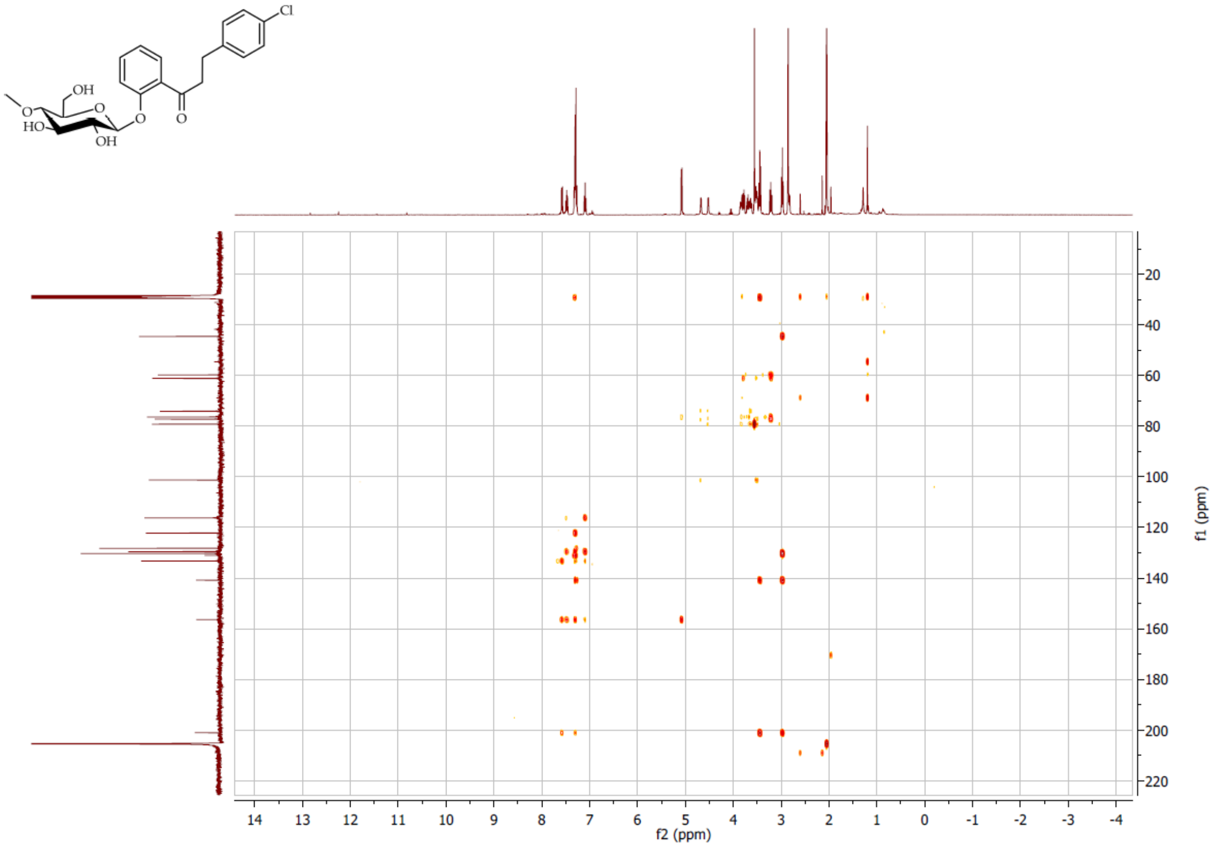

Figure S45. HMBC contour map – ^{1}H x ^{13}C of 4-chlorodihydrochalcone 2′-*O*-*β*-D-(4′′-*O*-methyl)-glucopyranoside (**3a**)

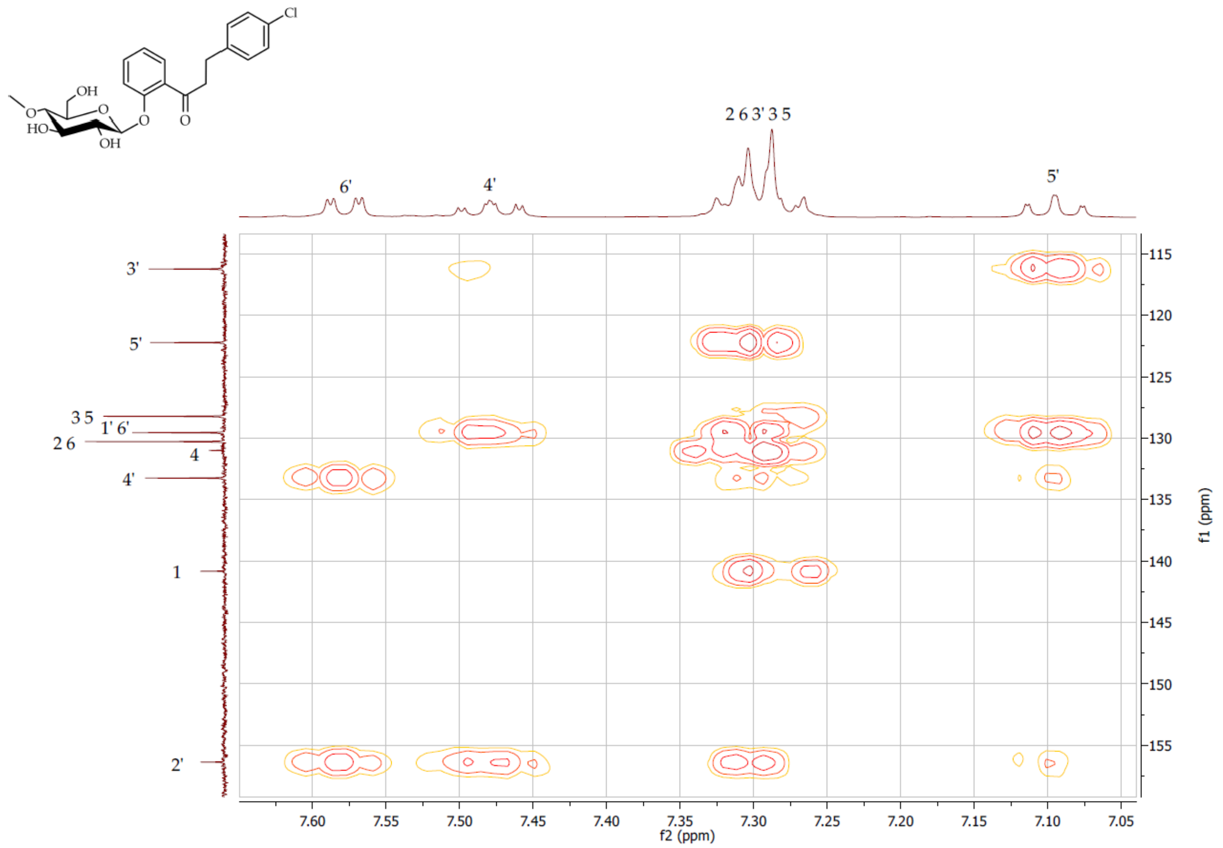

Figure S46. HMBC contour map – ^{1}H x ^{13}C expansion of 4-chlorodihydrochalcone 2′-*O*-*β*-D-(4′′-*O*-methyl)-glucopyranoside (**3a**)

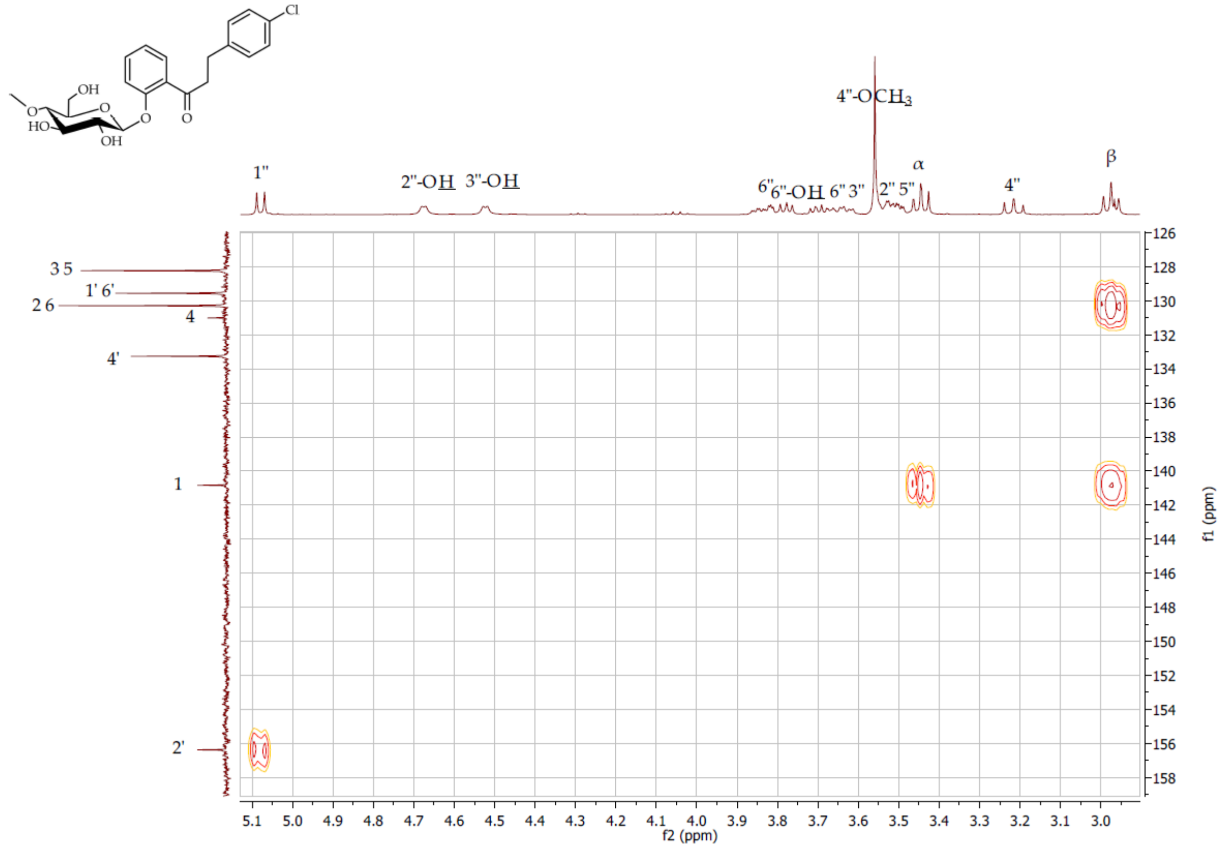

Figure S47. HMBC contour map – ^{1}H x ^{13}C expansion of 4-chlorodihydrochalcone 2′-*O*-*β*-D-(4′′-*O*-methyl)-glucopyranoside (**3a**)

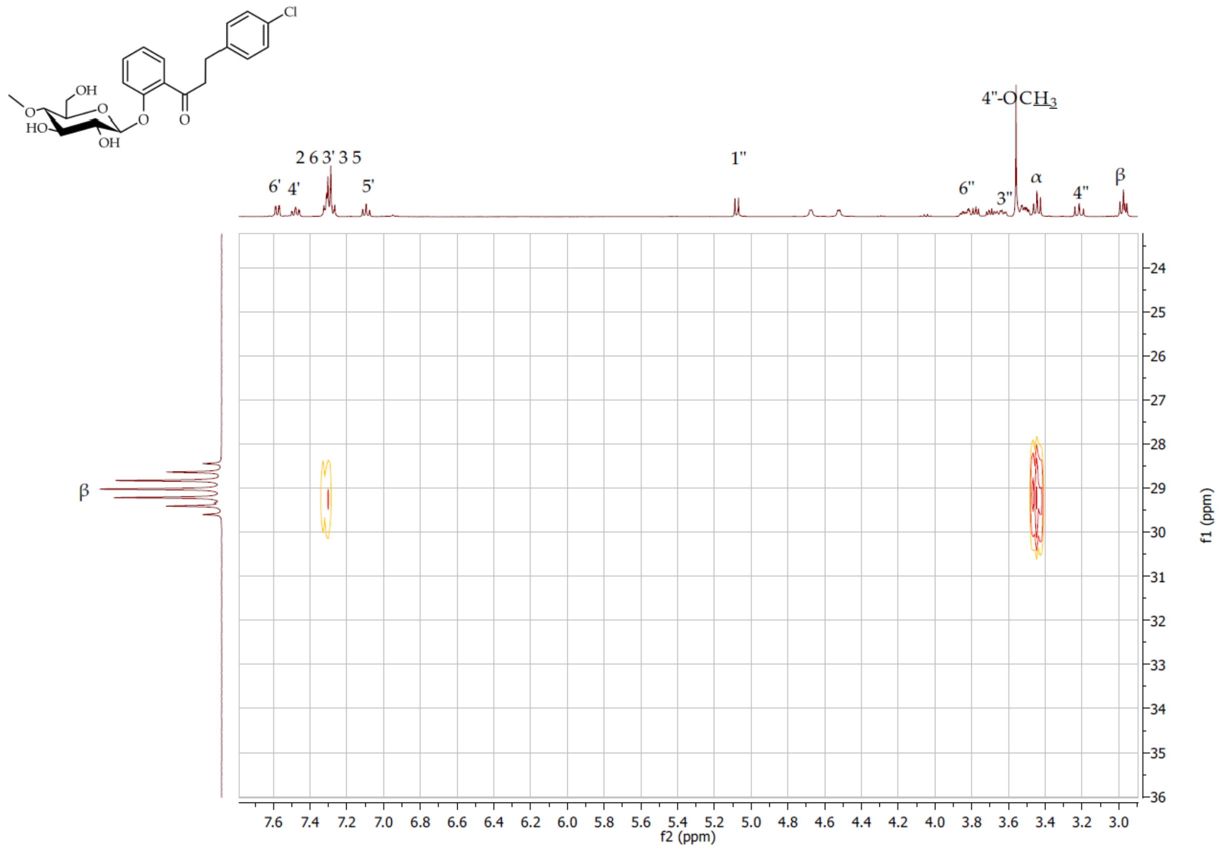

Figure S48. HMBC contour map – ^{1}H x ^{13}C expansion of 4-chlorodihydrochalcone 2′-*O*-*β*-D-(4′′-*O*-methyl)-glucopyranoside (**3a**)

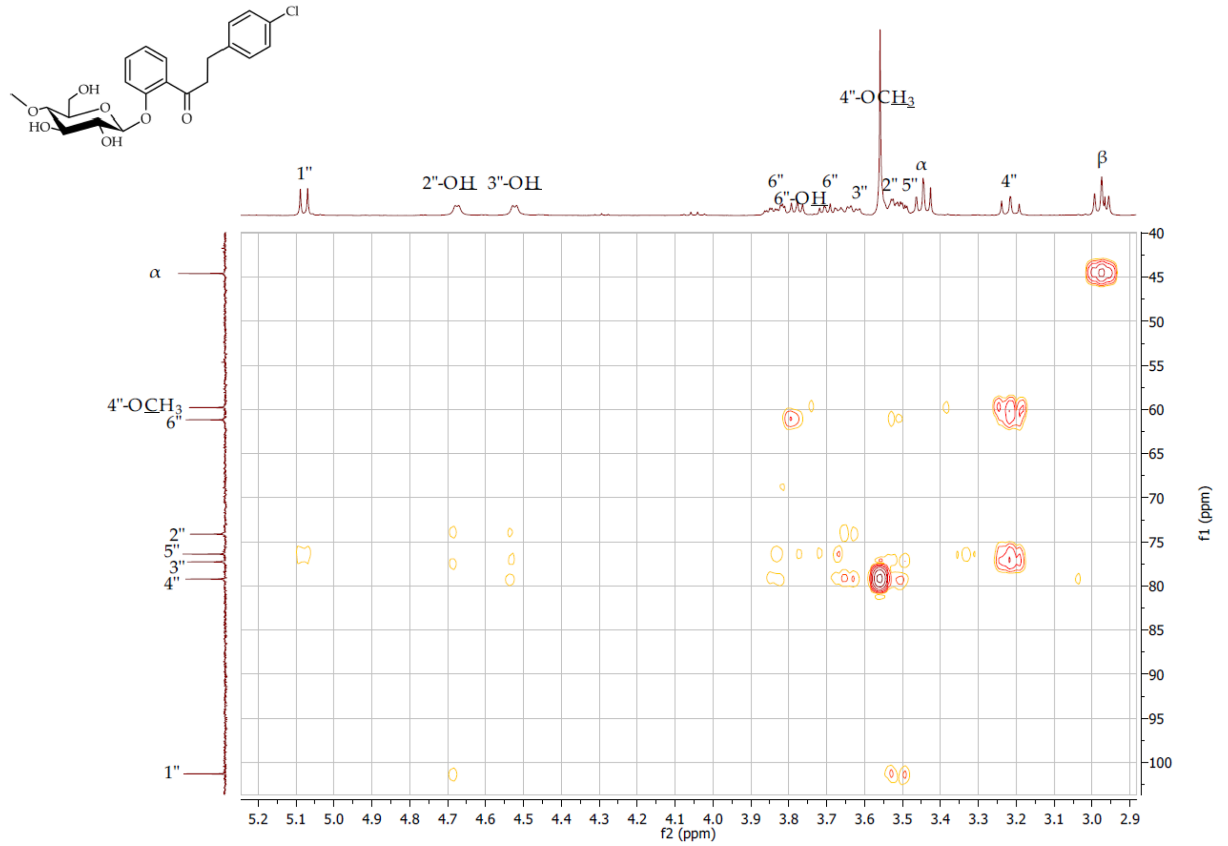

Figure S49. HMBC contour map – ^{1}H x ^{13}C expansion of 4-chlorodihydrochalcone 2′-*O*-*β*-D-(4′′-*O*-methyl)-glucopyranoside (**3a**)

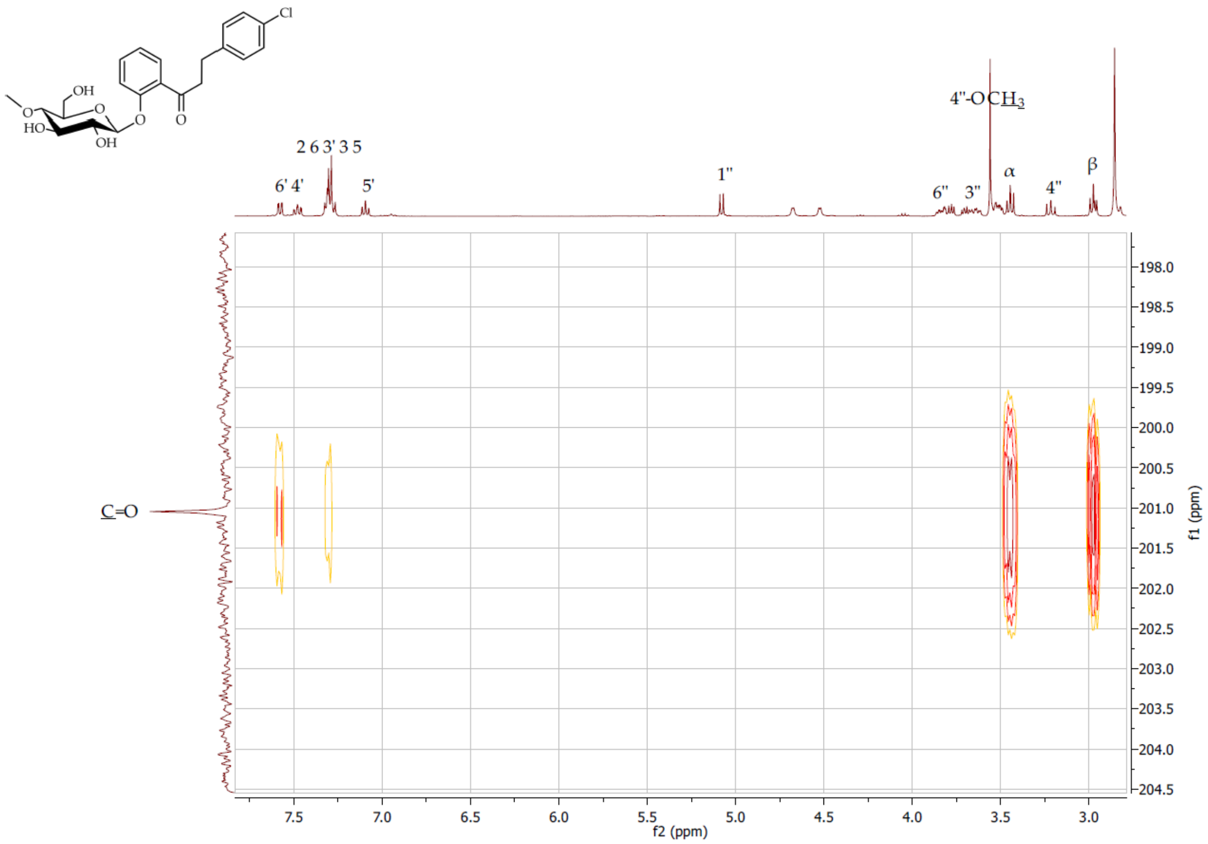

Figure S50. HMBC contour map – ^{1}H x ^{13}C expansion of 4-chlorodihydrochalcone 2′-*O*-*β*-D-(4′′-*O*-methyl)-glucopyranoside (**3a**)

Molecule 1

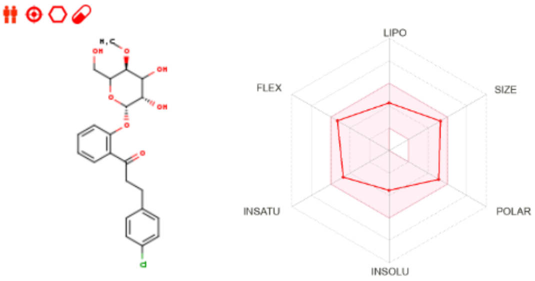

SMILES OCC1O[C@@H](Oc2ccccc2C(=O)CCc2ccc(cc2)Cl)[C@H](C([C@@H]1OC)O)O

Physicochemical Properties	
Formula	C22H25ClO7
Molecular weight	436.88 g/mol
Num. heavy atoms	30
Num. arom. heavy atoms	12
Fraction Csp3	0.41
Num. rotatable bonds	8
Num. H-bond acceptors	7
Num. H-bond donors	3
Molar Refractivity	109.82
TPSA	105.45 Å²

Lipophilicity	
Log $P_{o/w}$ (iLOGP)	3.19
Log $P_{o/w}$ (XLOGP3)	1.95
Log $P_{o/w}$ (WLOGP)	1.99
Log $P_{o/w}$ (MLOGP)	0.81
Log $P_{o/w}$ (SILICOS-IT)	2.73
Consensus Log $P_{o/w}$	2.14

Water Solubility	
Log *S* (ESOL)	-3.55
Solubility	1.25e-01 mg/ml ; 2.85e-04 mol/l
Class	Soluble
Log *S* (Ali)	-3.79
Solubility	7.10e-02 mg/ml ; 1.62e-04 mol/l
Class	Soluble
Log *S* (SILICOS-IT)	-4.70
Solubility	8.70e-03 mg/ml ; 1.99e-05 mol/l
Class	Moderately soluble

Pharmacokinetics	
GI absorption	High
BBB permeant	No
P-gp substrate	Yes
CYP1A2 inhibitor	No
CYP2C19 inhibitor	No
CYP2C9 inhibitor	No
CYP2D6 inhibitor	Yes
CYP3A4 inhibitor	Yes
Log K_p (skin permeation)	-7.58 cm/s

Druglikeness	
Lipinski	Yes; 0 violation
Ghose	Yes
Veber	Yes
Egan	Yes
Muegge	Yes
Bioavailability Score	0.55

Medicinal Chemistry	
PAINS	0 alert
Brenk	0 alert
Leadlikeness	No; 2 violations: MW>350, Rotors>7
Synthetic accessibility	4.85

Figure S51. 4-Chlorodihydrochalcone 2′-*O*-*β*-D-(4′′-*O*-methyl)-glucopyranoside (**3a**) physicochemical and ADME parameters prediction using the SwissADME modelling

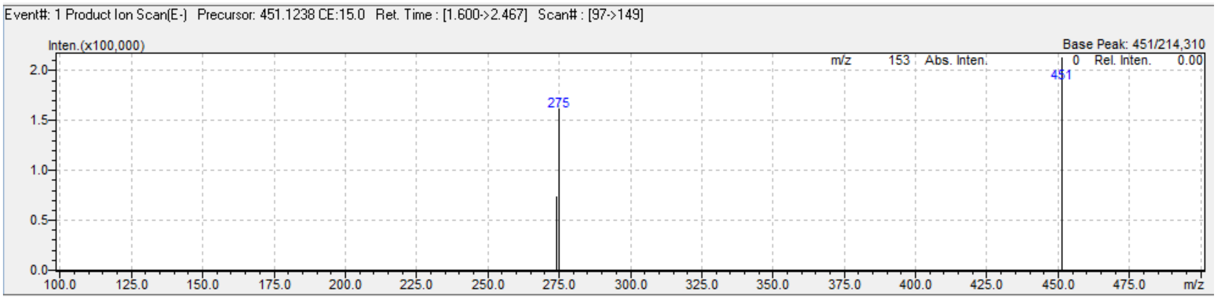

Figure S52. MS analysis of 4-chloro-2′-hydroxydihydrochalcone 5′-*O*-*β*-D-(4′′-*O*-methyl)-glucopyranoside (**3b**)

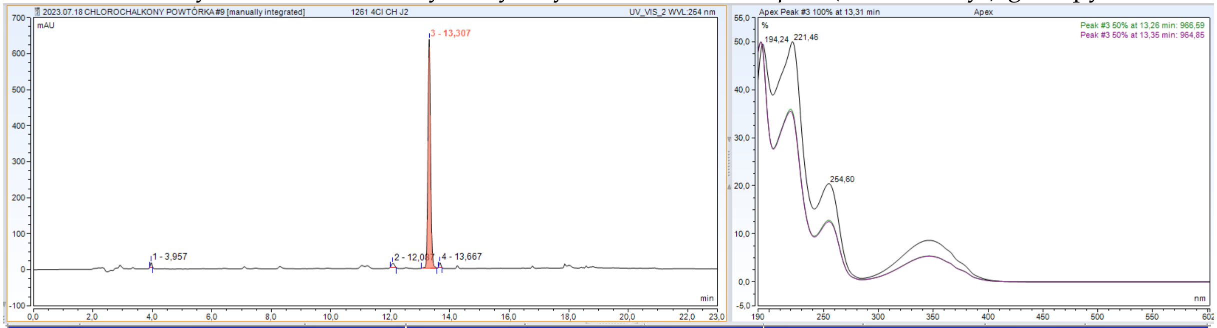

Peak No.	Peak Name	Ret.Time min	Rel.Area %	Area mAU*min	Height mAU	Match	RSD Match %	PPI nm	RSD PPI %
1		3,957	1,40	0,9341	15,17	999	0,07	229,5	0,50
2		12,087	2,01	1,3428	11,57	1000	0,02	200,1	0,12
3		*13,307*	*95,21*	*63,5668*	*636,27*	*955*	*4,31*	*211,8*	*2,00*
4		13,667	1,38	0,9216	13,75	1000	0,01	266,8	0,23
Maximum			95,21	63,5668	636,27	1000	4,31		2,00
Minimum			1,38	0,9216	11,57	955	0,01		0,12

Figure S53. HPLC analysis of 4-chloro-2′-hydroxydihydrochalcone 5′-*O*-*β*-D-(4′′-*O*-methyl)-glucopyranoside (**3b**)

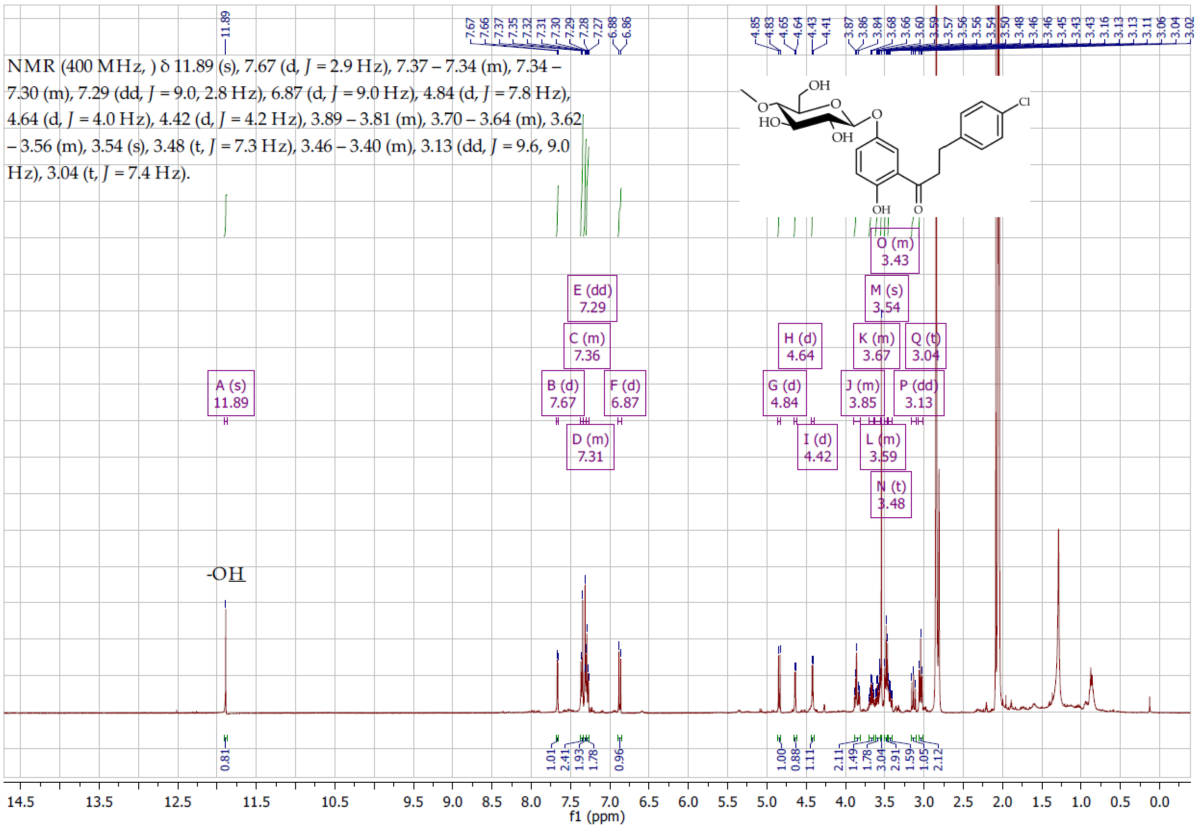

Figure S54. ^{1}H NMR spectrum (*δ*, acetone-d6, 600 MHz) of 4-chloro-2′-hydroxydihydrochalcone 5′-*O*-*β*-D-(4′′-*O*-methyl)-glucopyranoside (**3b**)

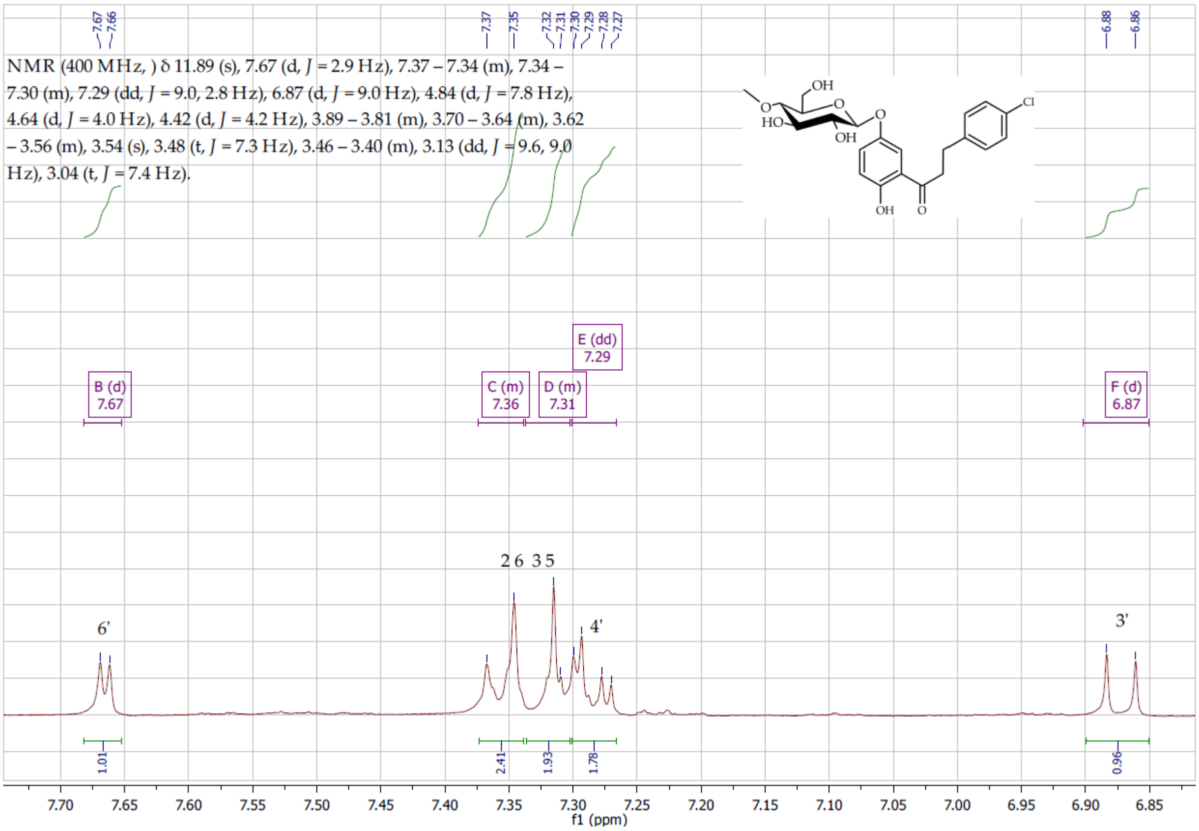

Figure S55. ^{1}H NMR spectrum expansion (*δ*, acetone-d6, 600 MHz) of 4-chloro-2′-hydroxydihydrochalcone 5′-*O*-*β*-D-(4′′-*O*-methyl)-glucopyranoside (**3b**)

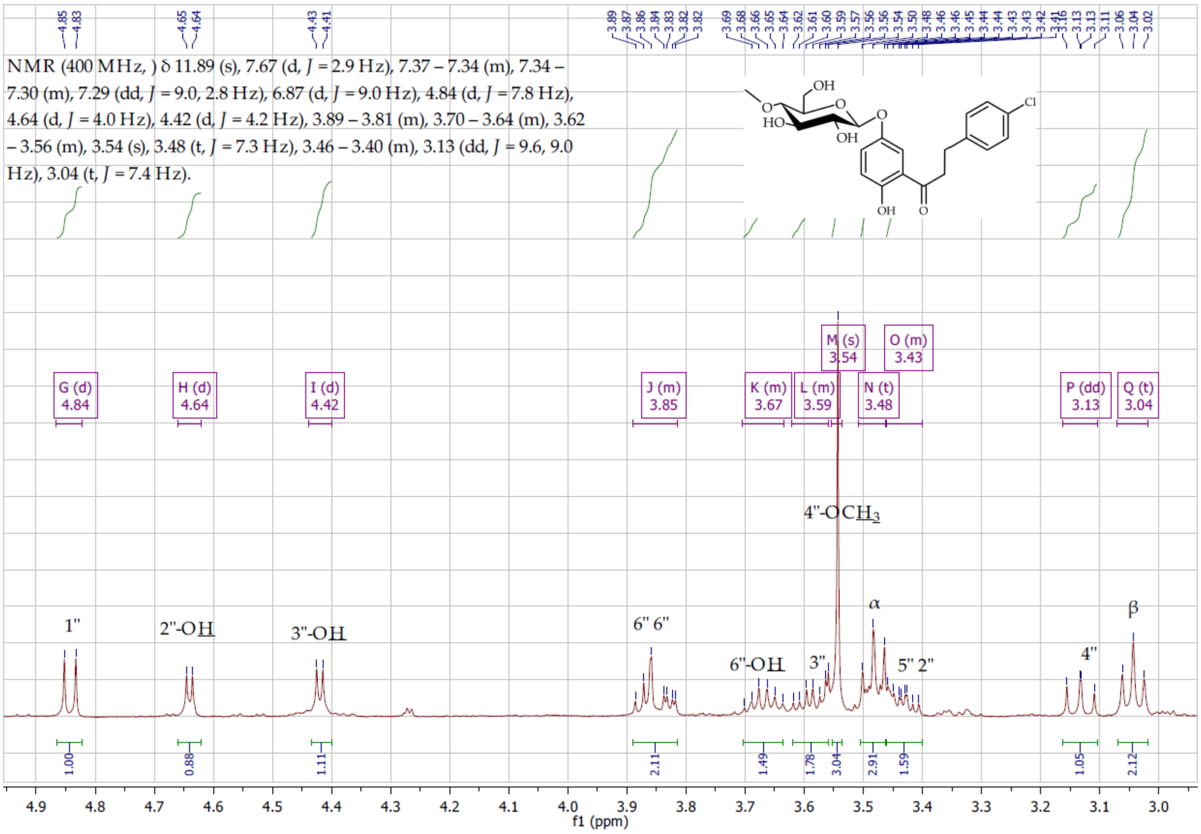

Figure S56. ^{1}H NMR spectrum expansion (*δ*, acetone-d6, 600 MHz) of 4-chloro-2′-hydroxydihydrochalcone 5′-*O*-*β*-D-(4′′-*O*-methyl)-glucopyranoside (**3b**)

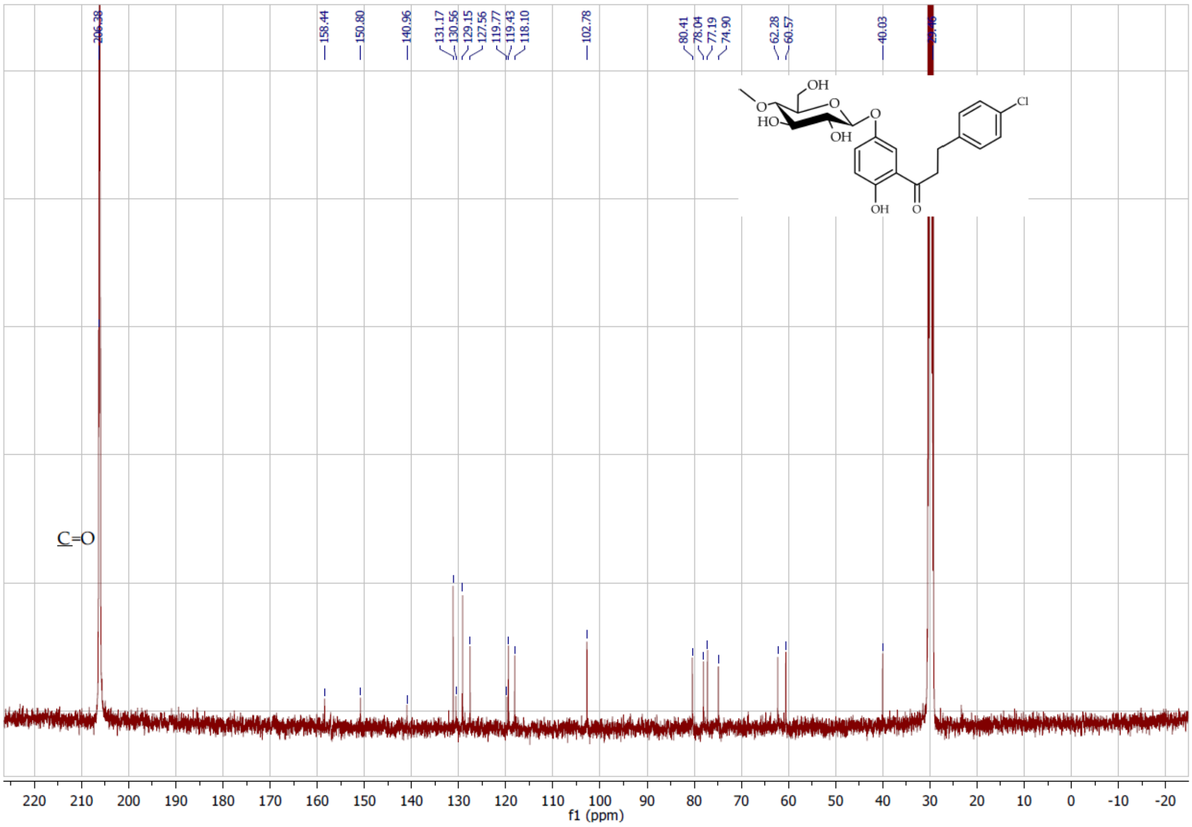

Figure S57. ^{13}C NMR spectrum (*δ*, acetone-d6, 151 MHz) of 4-chloro-2′-hydroxydihydrochalcone 5′-*O*-*β*-D-(4′′-*O*-methyl)-glucopyranoside (**3b**)

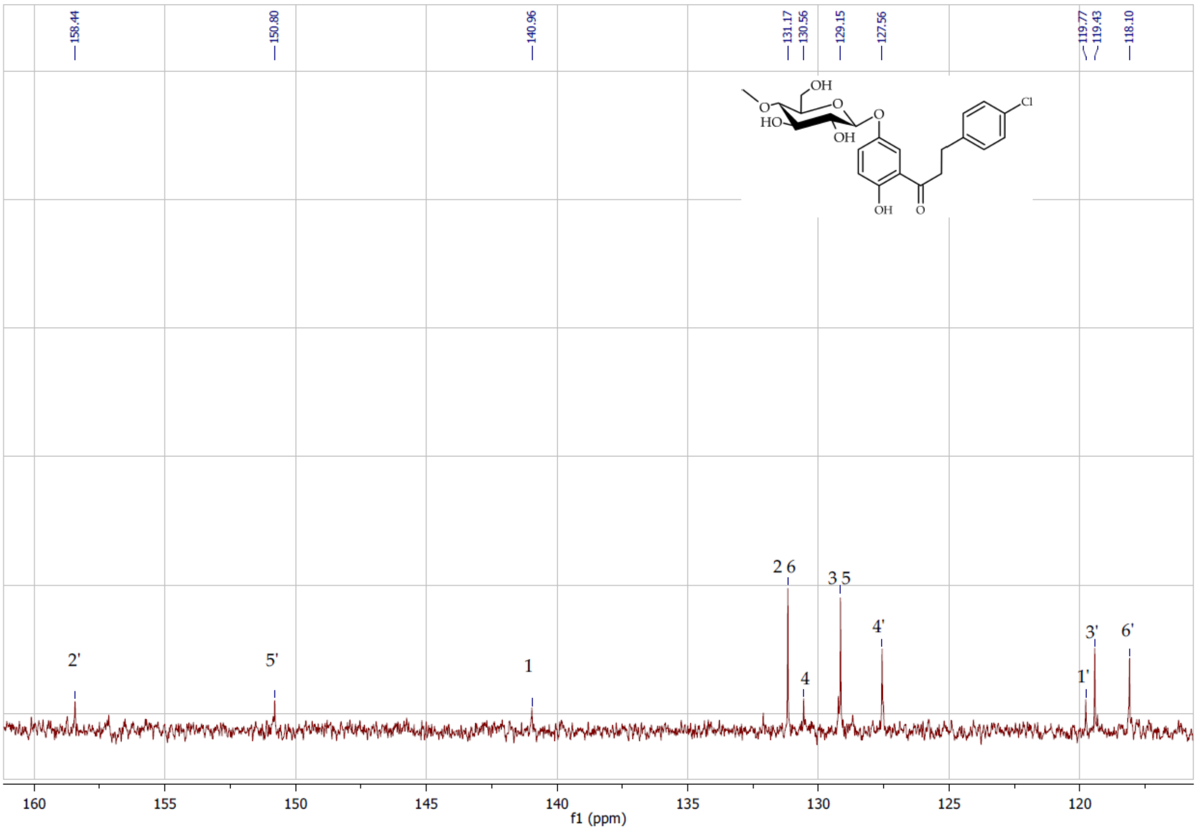

Figure S58. ^{13}C NMR spectrum expansion (*δ*, acetone-d6, 151 MHz) of 4-chloro-2′-hydroxydihydrochalcone 5′-*O*-*β*-D-(4′′-*O*-methyl)-glucopyranoside (**3b**)

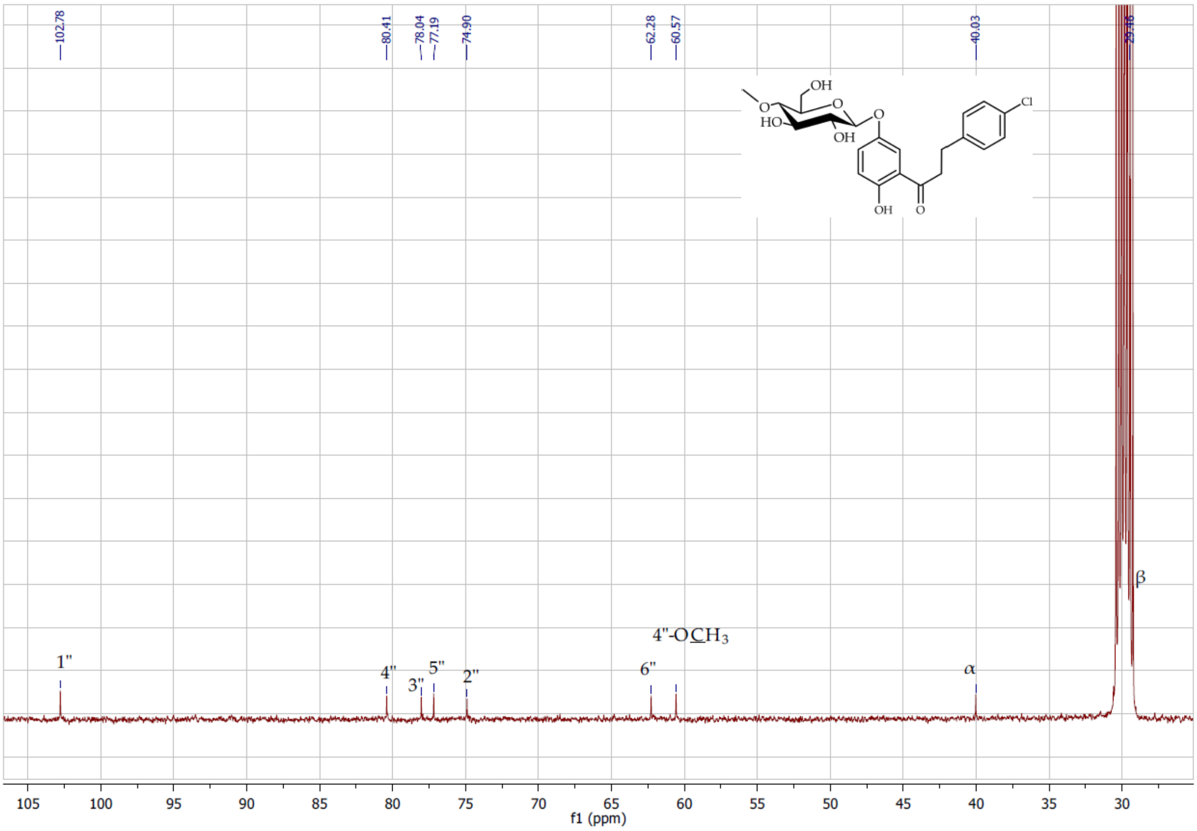

Figure S59. ^{13}C NMR spectrum expansion (*δ*, acetone-d6, 151 MHz) of 4-chloro-2′-hydroxydihydrochalcone 5′-*O*-*β*-D-(4′′-*O*-methyl)-glucopyranoside (**3b**)

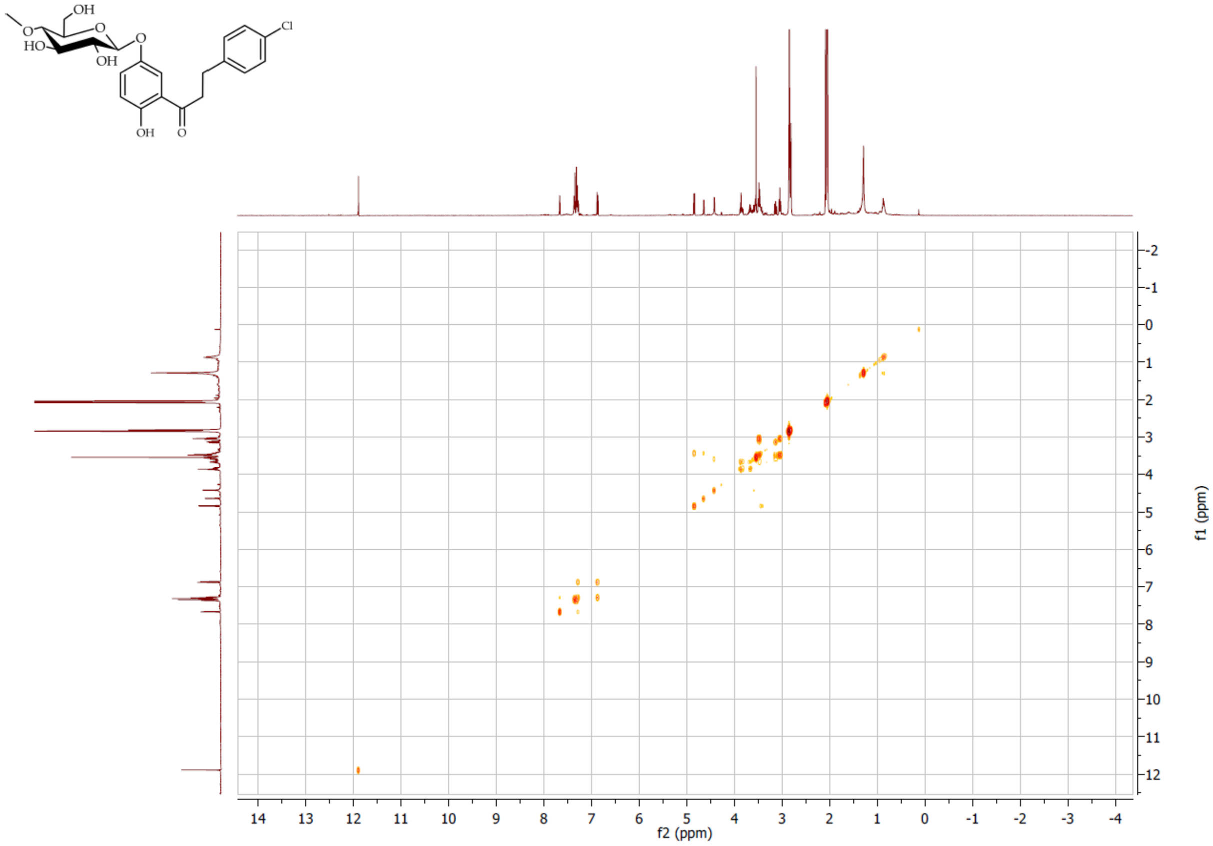

Figure S60. COSY contour map – ^{1}H x ^{1}H of 4-chloro-2′-hydroxydihydrochalcone 5′-*O*-*β*-D-(4′′-*O*-methyl)-glucopyranoside (**3b**)

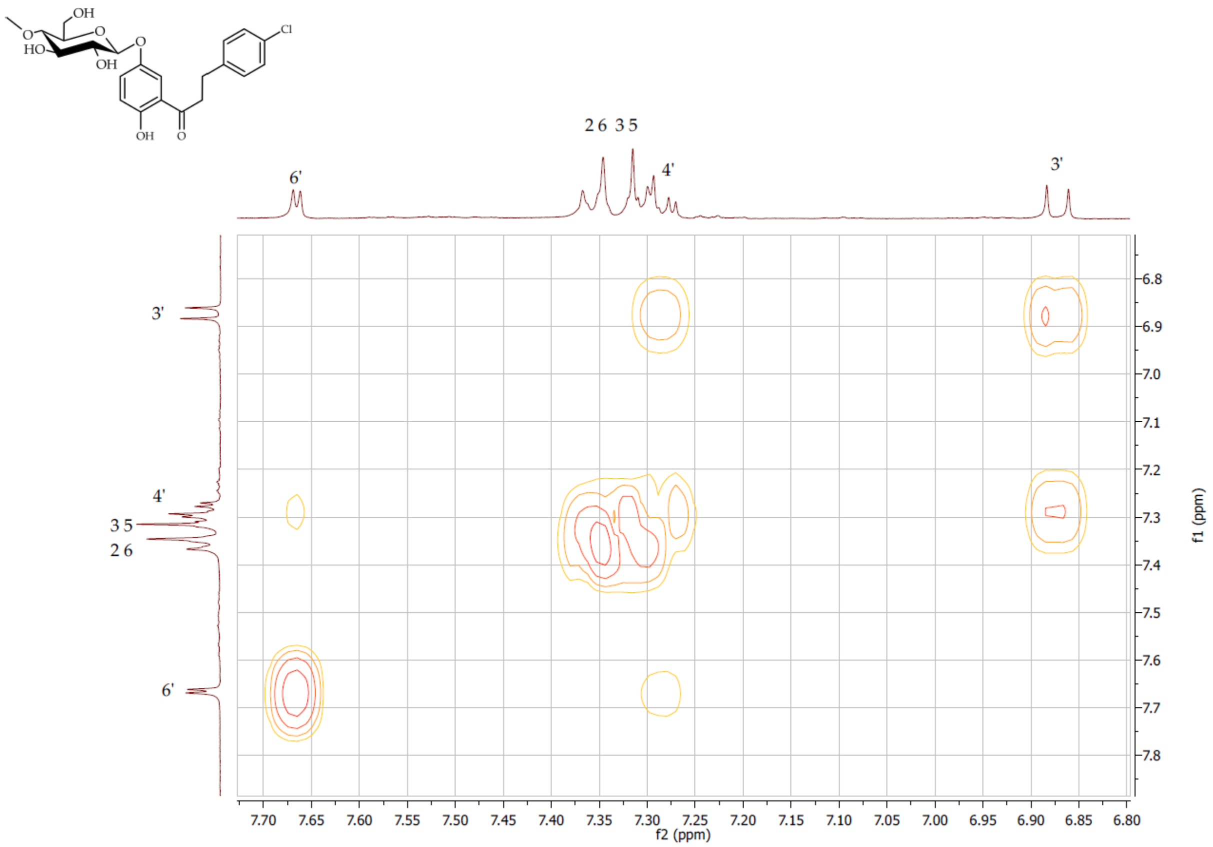

Figure S61. COSY contour map – ^{1}H x ^{1}H expansion of 4-chloro-2′-hydroxydihydrochalcone 5′-*O*-*β*-D-(4′′-*O*-methyl)-glucopyranoside (**3b**)

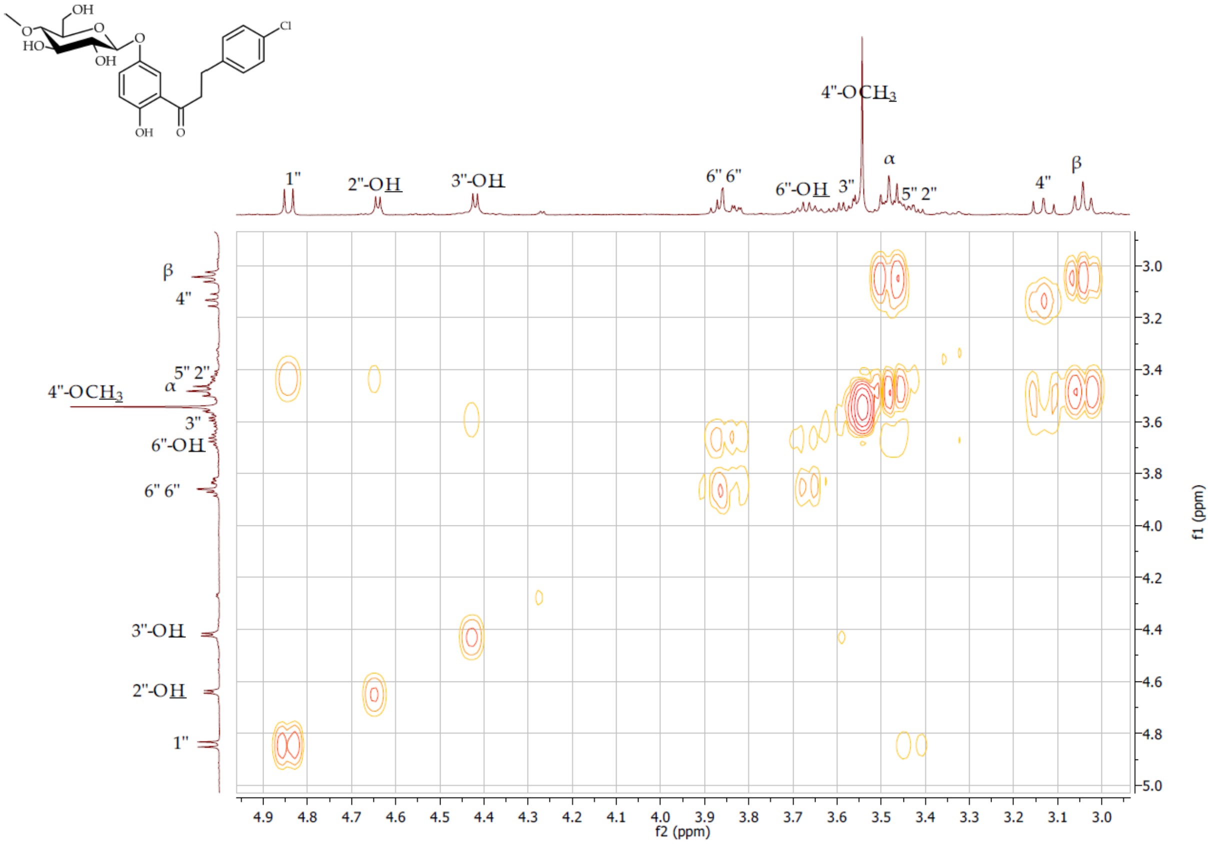

Figure S62. COSY contour map – ^{1}H x ^{1}H expansion of 4-chloro-2′-hydroxydihydrochalcone 5′-*O*-*β*-D-(4′′-*O*-methyl)-glucopyranoside (**3b**)

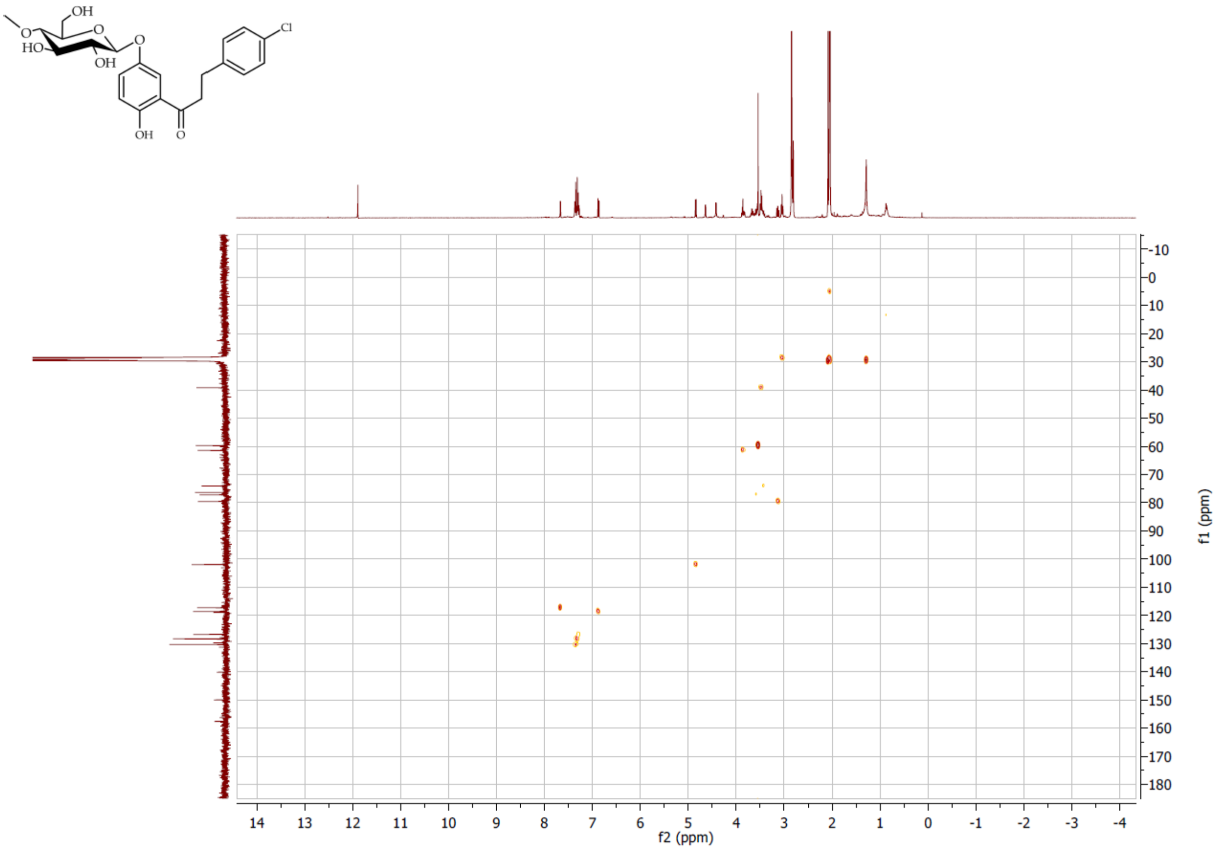

Figure S63. HMQC contour map – [1]H x ^{13}C of 4-chloro-2′-hydroxydihydrochalcone 5′-*O*-*β*-D-(4′′-*O*-methyl)-glucopyranoside (**3b**)

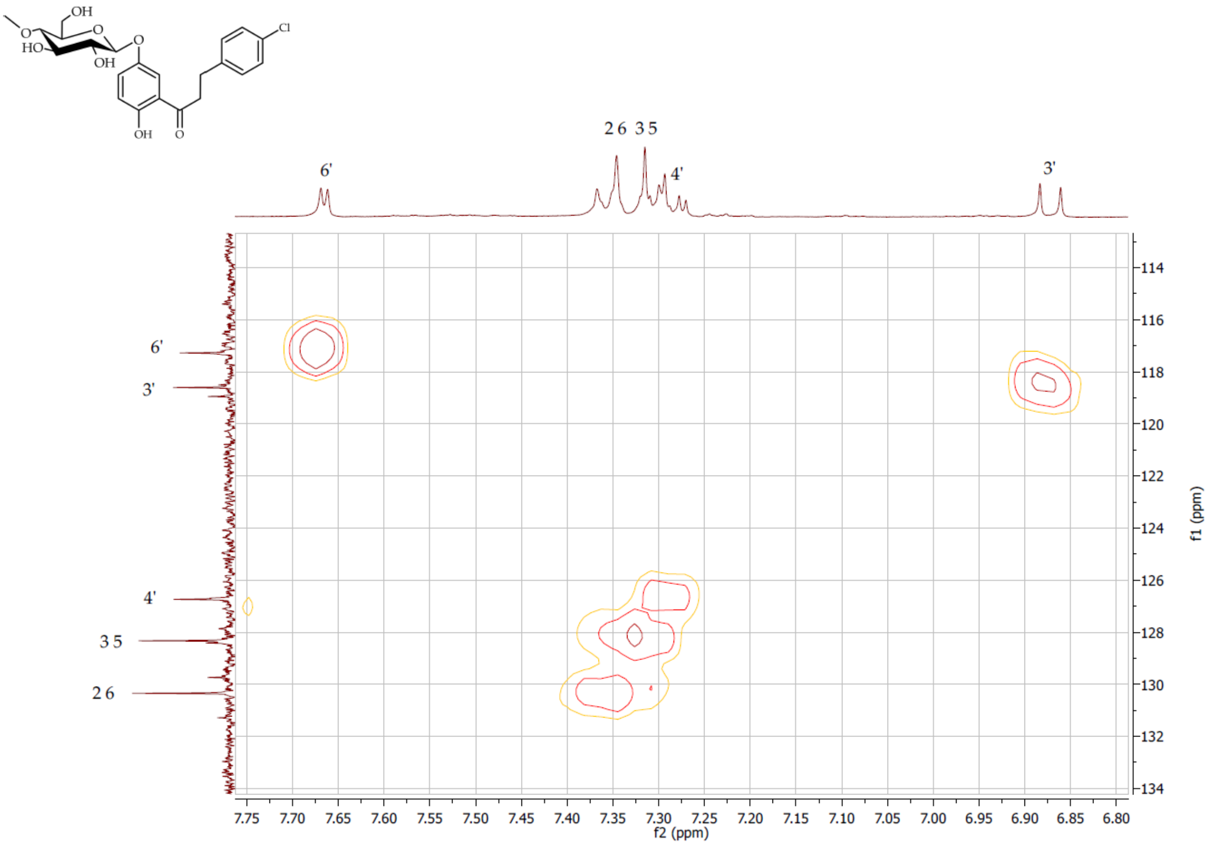

Figure S64. HMQC contour map – [1]H x ^{13}C expansion of 4-chloro-2′-hydroxydihydrochalcone 5′-*O*-*β*-D-(4′′-*O*-methyl)-glucopyranoside (**3b**)

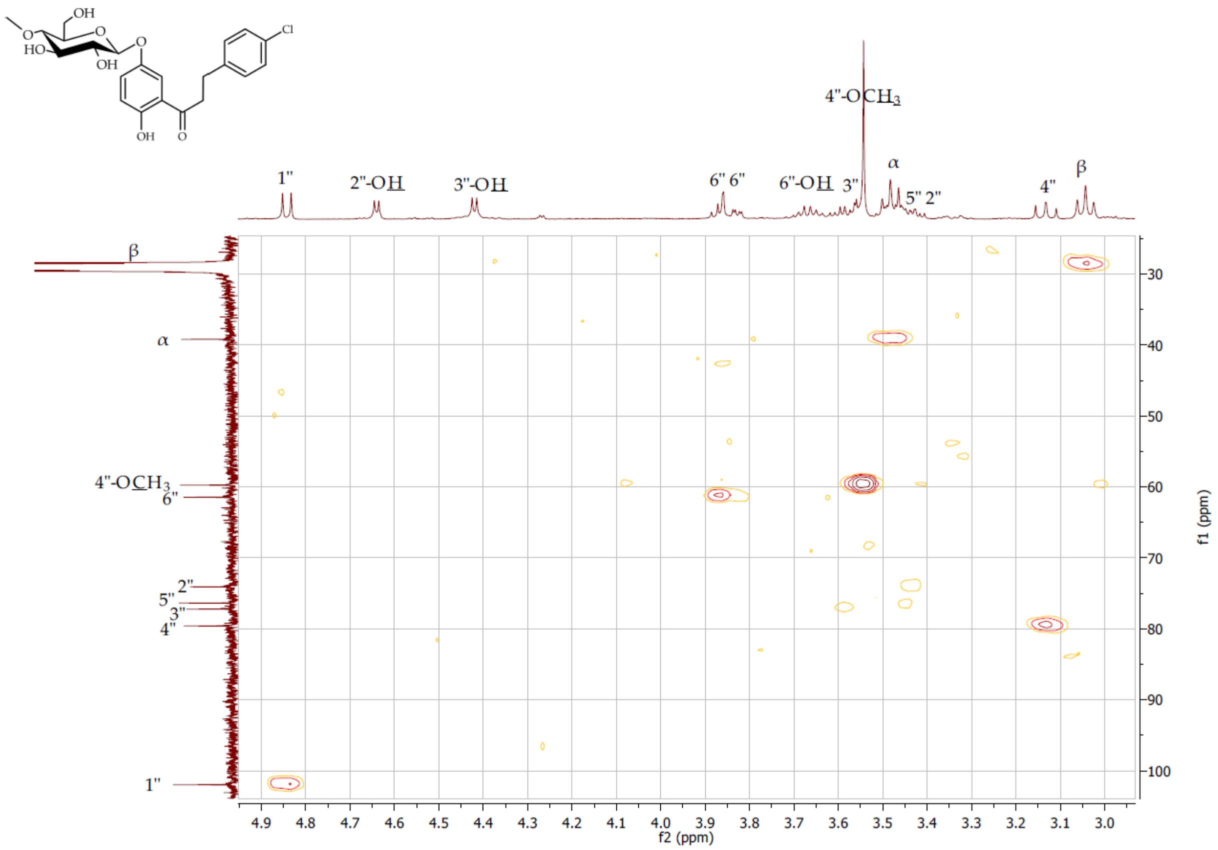

Figure S65. HMQC contour map – [1]H x ^{13}C expansion of 4-chloro-2′-hydroxydihydrochalcone 5′-*O*-*β*-D-(4′′-*O*-methyl)-glucopyranoside (**3b**)

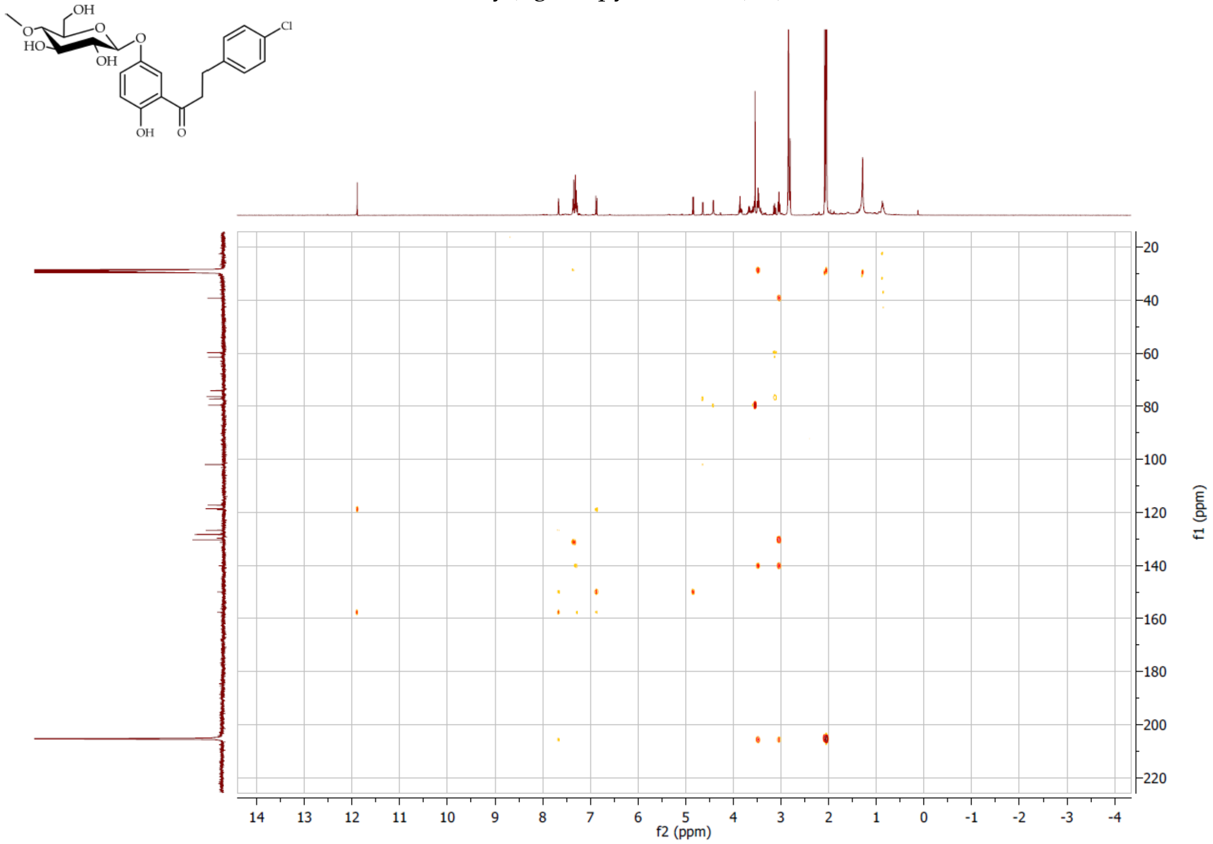

Figure S66. HMBC contour map – ^{1}H x ^{13}C of 4-chloro-2′-hydroxydihydrochalcone 5′-*O*-*β*-D-(4′′-*O*-methyl)-glucopyranoside (**3b**)

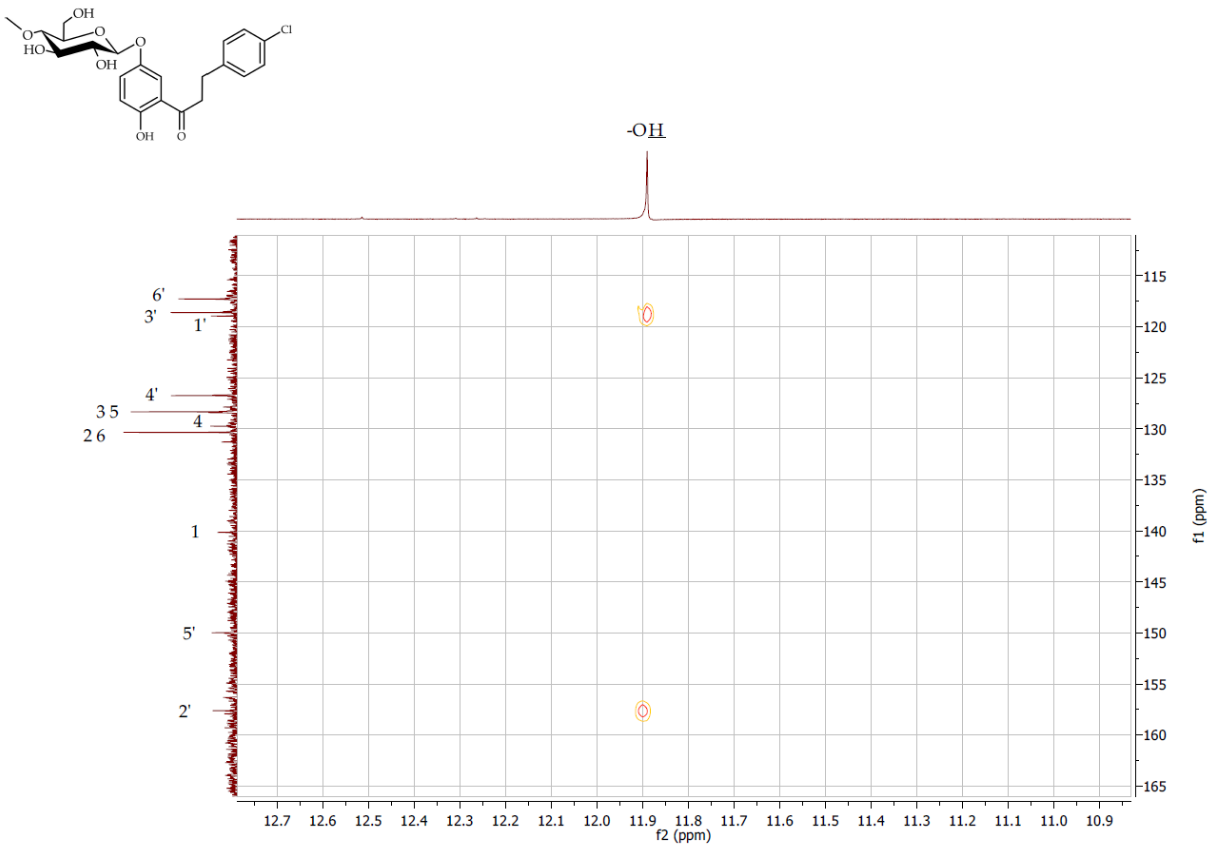

Figure S67. HMBC contour map – ^{1}H x ^{13}C expansion of 4-chloro-2′-hydroxydihydrochalcone 5′-*O*-*β*-D-(4′′-*O*-methyl)-glucopyranoside (**3b**)

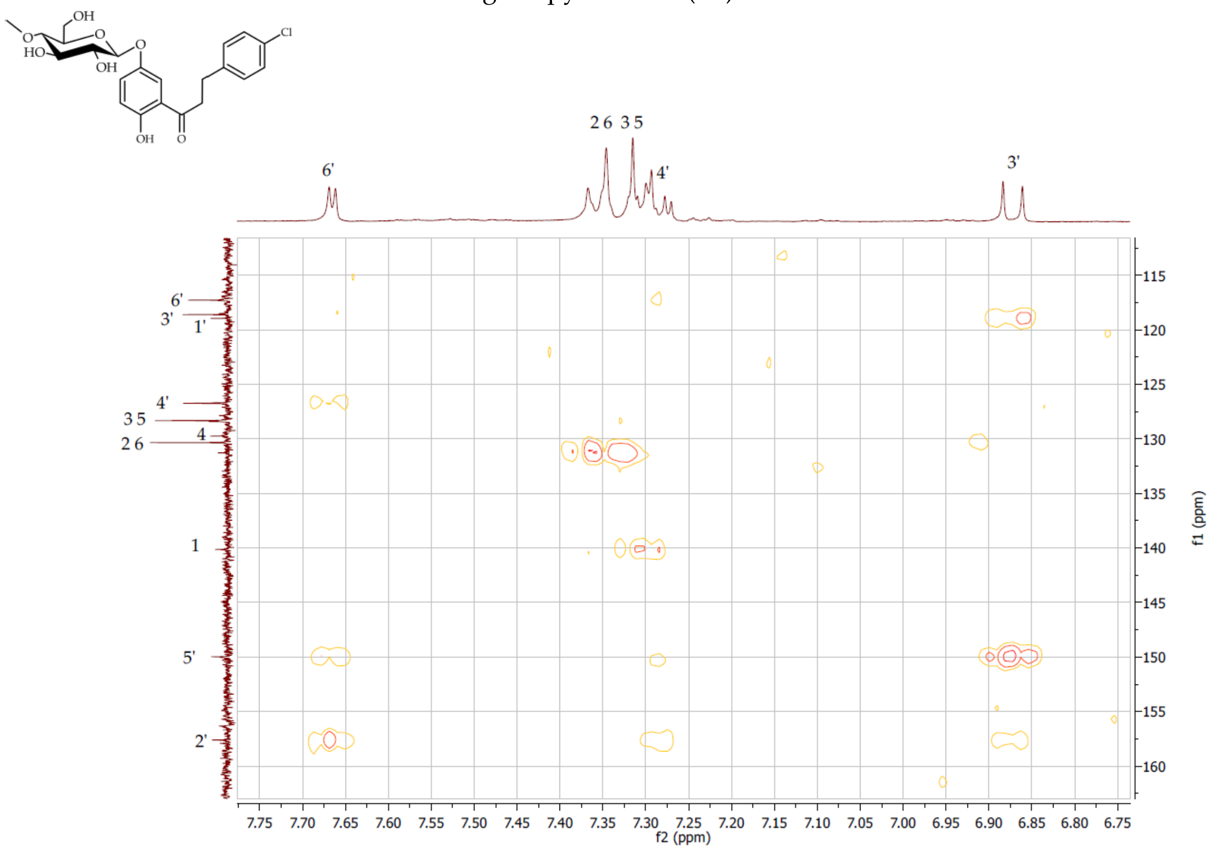

Figure S68. HMBC contour map – ^{1}H x ^{13}C expansion of 4-chloro-2′-hydroxydihydrochalcone 5′-*O*-*β*-D-(4′′-*O*-methyl)-glucopyranoside (**3b**)

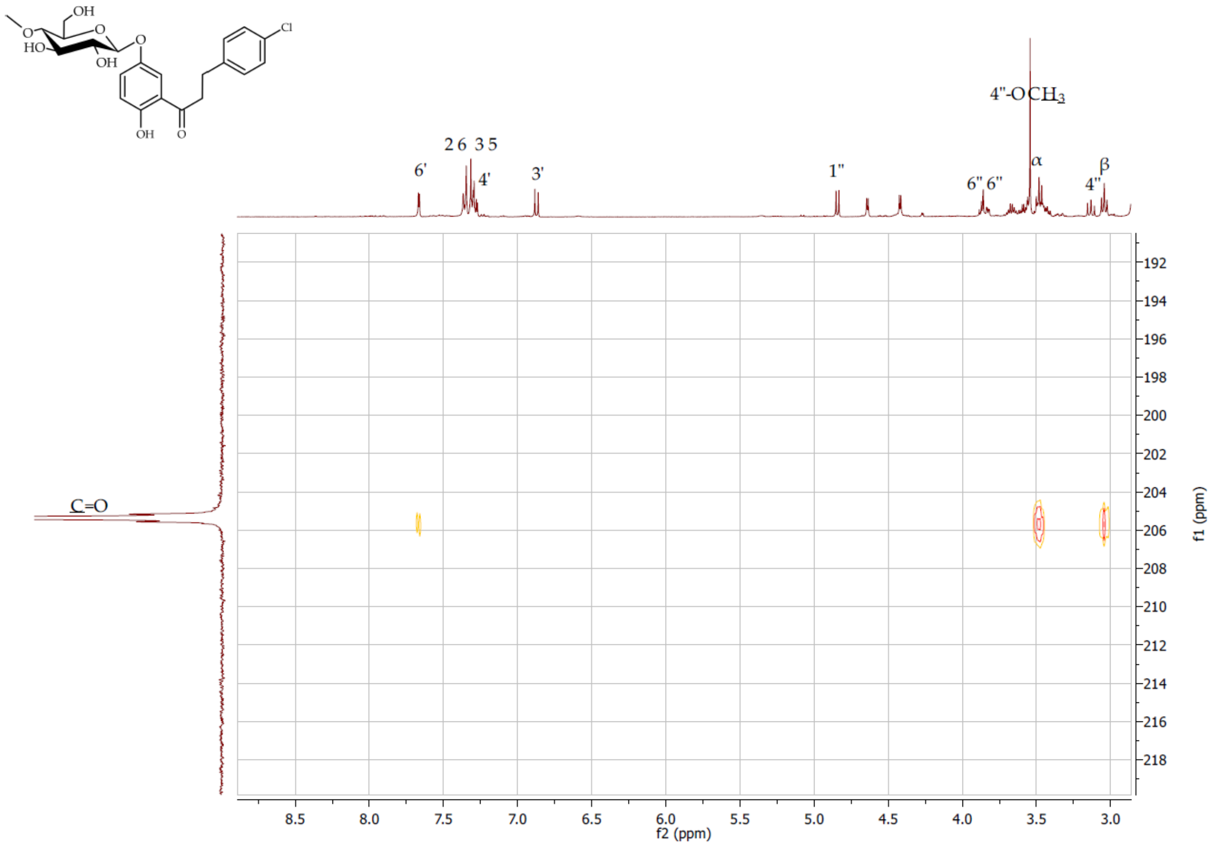

Figure S69. HMBC contour map – ^{1}H x ^{13}C expansion of 4-chloro-2′-hydroxydihydrochalcone 5′-*O*-*β*-D-(4′′-*O*-methyl)-glucopyranoside (**3b**)

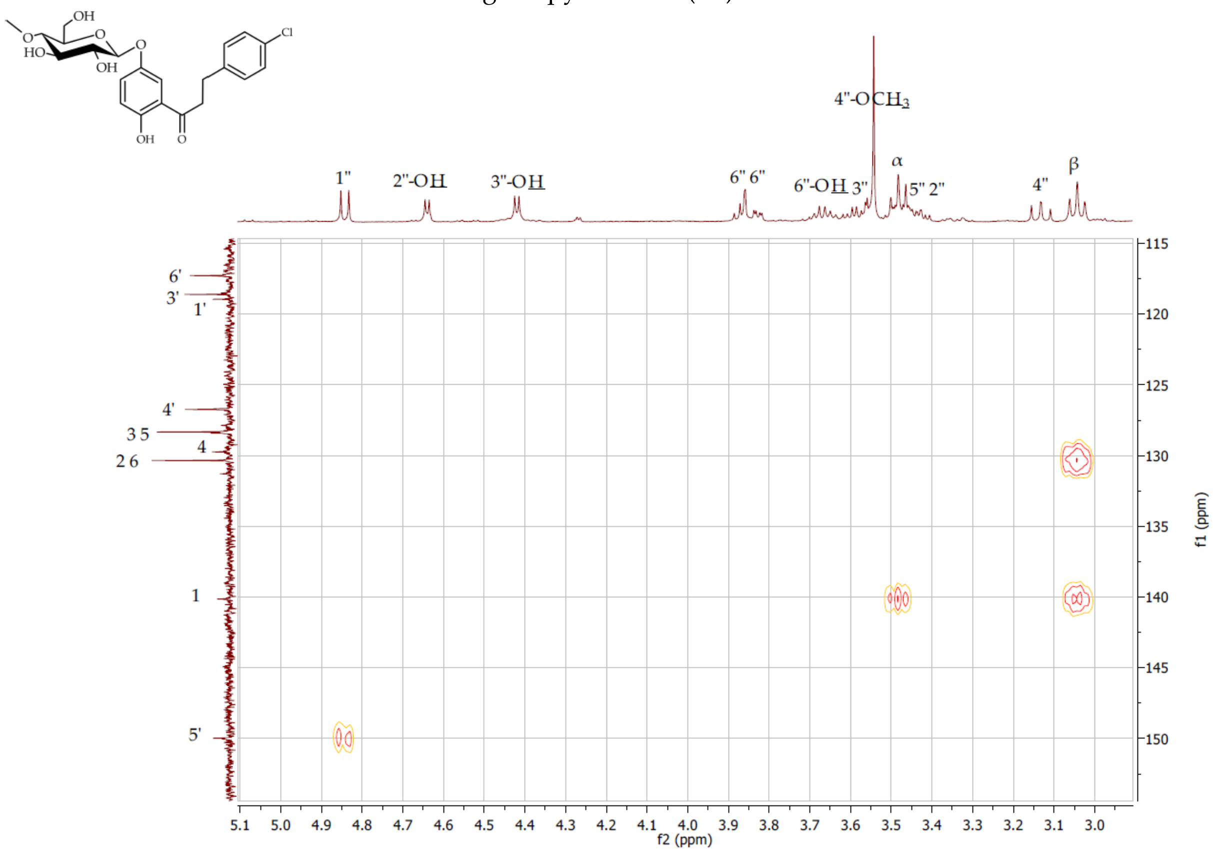

Figure S70. HMBC contour map – ^{1}H x ^{13}C expansion of 4-chloro-2′-hydroxydihydrochalcone 5′-*O*-*β*-D-(4′′-*O*-methyl)-glucopyranoside (**3b**)

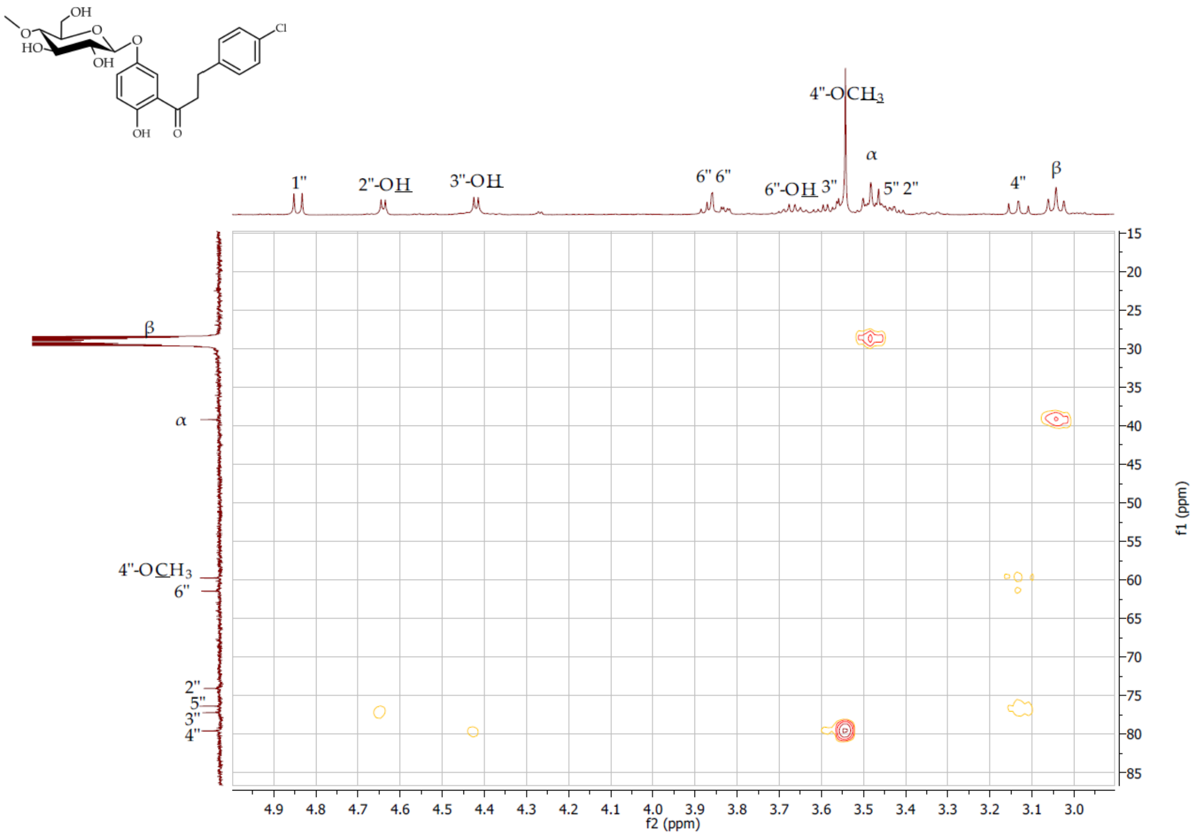

Figure S71. HMBC contour map – ^{1}H x ^{13}C expansion of 4-chloro-2′-hydroxydihydrochalcone 5′-*O*-*β*-D-(4′′-*O*-methyl)-glucopyranoside (**3b**)

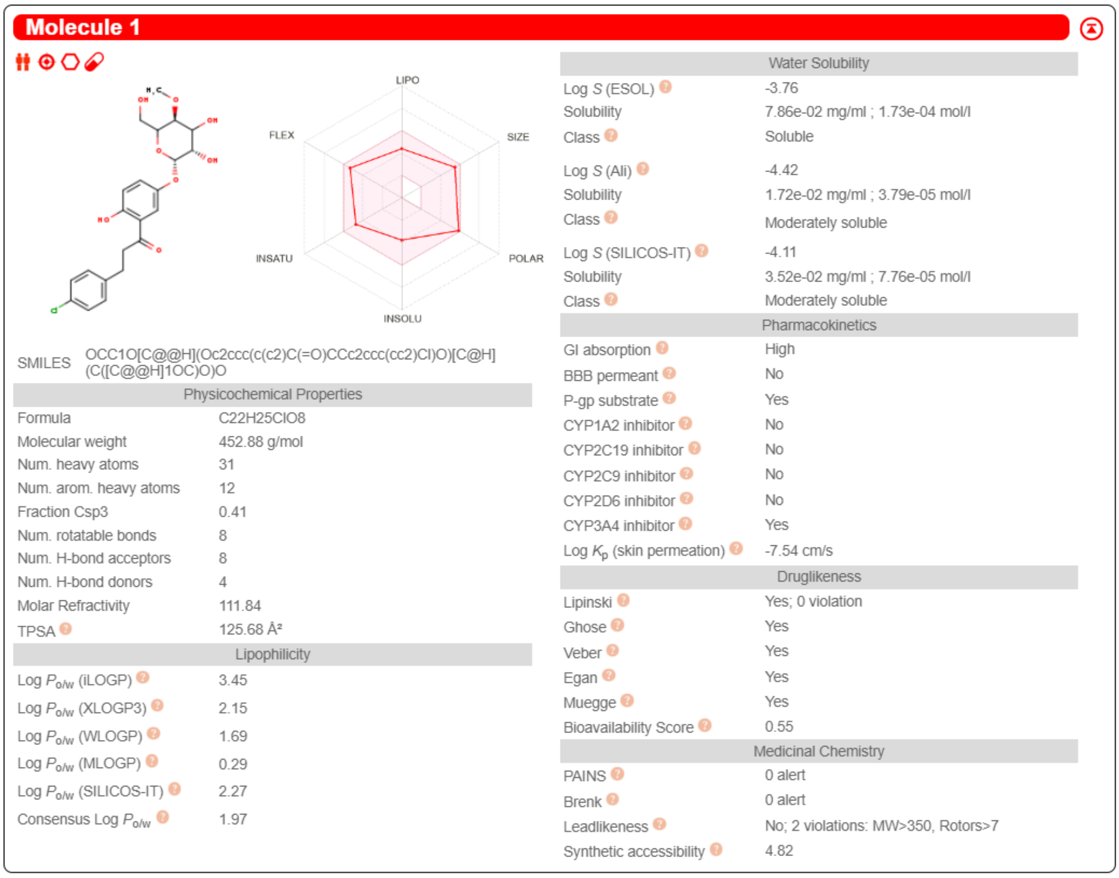
Molecule 1

LIPO, SIZE, POLAR, INSOLU, INSATU, FLEX

SMILES: OCC1O[C@@H](Oc2ccc(c(c2)C(=O)CCc2ccc(cc2)Cl)O)[C@H](C([C@@H]1OC)O)O

Physicochemical Properties	
Formula	C22H25ClO8
Molecular weight	452.88 g/mol
Num. heavy atoms	31
Num. arom. heavy atoms	12
Fraction Csp3	0.41
Num. rotatable bonds	8
Num. H-bond acceptors	8
Num. H-bond donors	4
Molar Refractivity	111.84
TPSA	125.68 Å²

Lipophilicity	
Log $P_{o/w}$ (iLOGP)	3.45
Log $P_{o/w}$ (XLOGP3)	2.15
Log $P_{o/w}$ (WLOGP)	1.69
Log $P_{o/w}$ (MLOGP)	0.29
Log $P_{o/w}$ (SILICOS-IT)	2.27
Consensus Log $P_{o/w}$	1.97

Water Solubility	
Log *S* (ESOL)	-3.76
Solubility	7.86e-02 mg/ml ; 1.73e-04 mol/l
Class	Soluble
Log *S* (Ali)	-4.42
Solubility	1.72e-02 mg/ml ; 3.79e-05 mol/l
Class	Moderately soluble
Log *S* (SILICOS-IT)	-4.11
Solubility	3.52e-02 mg/ml ; 7.76e-05 mol/l
Class	Moderately soluble

Pharmacokinetics	
GI absorption	High
BBB permeant	No
P-gp substrate	Yes
CYP1A2 inhibitor	No
CYP2C19 inhibitor	No
CYP2C9 inhibitor	No
CYP2D6 inhibitor	No
CYP3A4 inhibitor	Yes
Log K_p (skin permeation)	-7.54 cm/s

Druglikeness	
Lipinski	Yes; 0 violation
Ghose	Yes
Veber	Yes
Egan	Yes
Muegge	Yes
Bioavailability Score	0.55

Medicinal Chemistry	
PAINS	0 alert
Brenk	0 alert
Leadlikeness	No; 2 violations: MW>350, Rotors>7
Synthetic accessibility	4.82

Figure S72. 4-Chloro-2′-hydroxydihydrochalcone 5′-*O*-*β*-D-(4′′-*O*-methyl)-glucopyranoside (**3b**) physicochemical and ADME parameters prediction using the SwissADME modelling

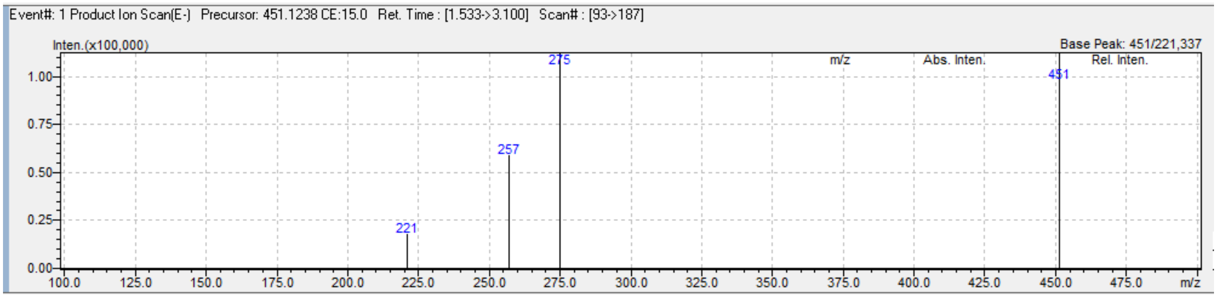

Figure S73. MS analysis of 4-chloro-2′-hydroxydihydrochalcone 3-*O*-*β*-D-(4′′-*O*-methyl)-glucopyranoside (**3c**)

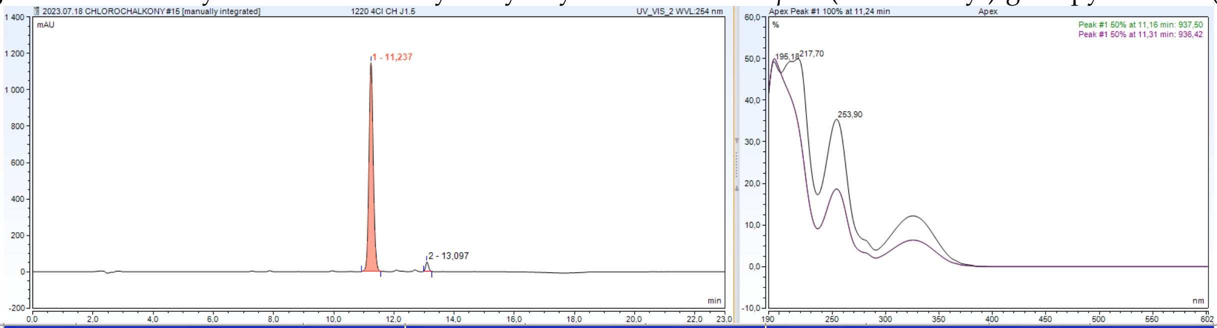

Peak No.	Peak Name	Ret.Time min	Rel.Area %	Area mAU*min	Height mAU	Match	RSD Match %	PPI nm	RSD PPI %
1		11,237	97,03	188,7442	1148,41	924	7,34	212,9	3,07
2		13,097	2,97	5,7721	52,39	999	0,09	204,4	0,24
Maximum			97,03	188,7442	1148,41	999	7,34		3,07
Minimum			2,97	5,7721	52,39	924	0,09		0,24

Figure S74. HPLC analysis of 4-chloro-2′-hydroxydihydrochalcone 3-*O*-*β*-D-(4′′-*O*-methyl)-glucopyranoside (**3c**)

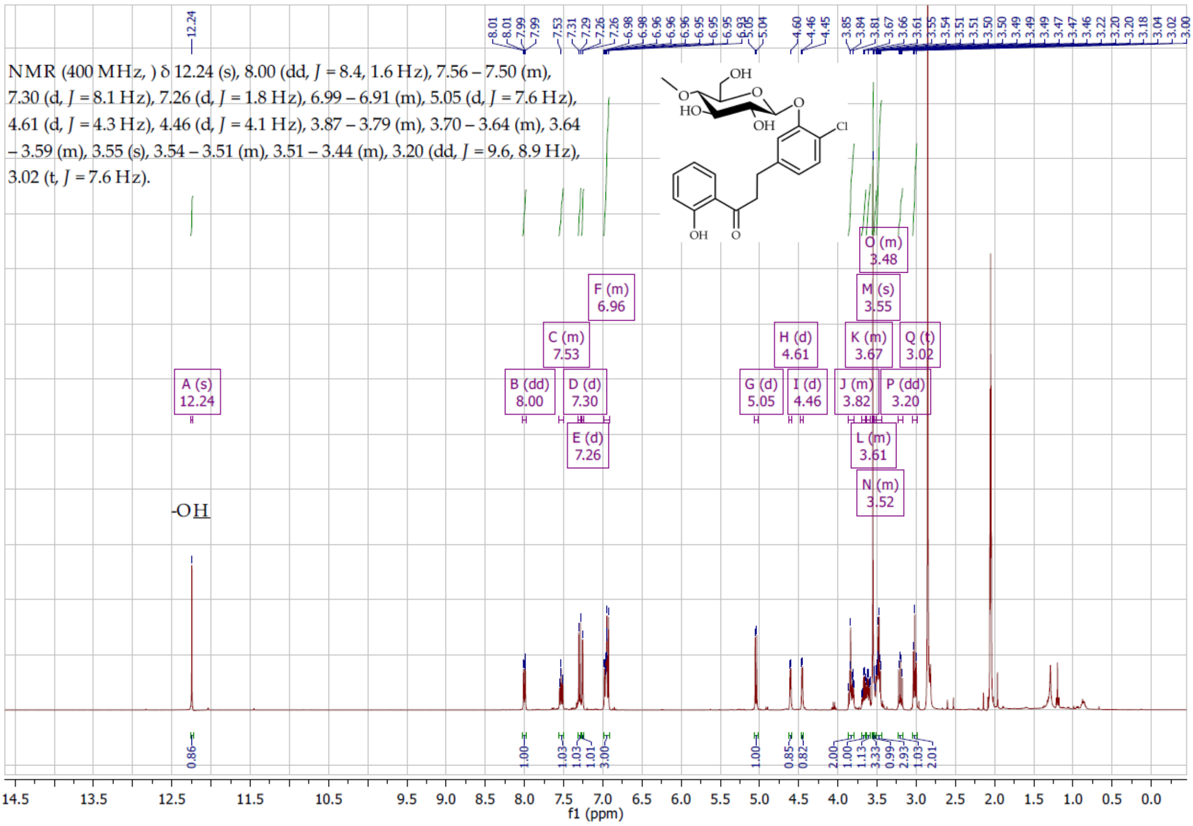

Figure S75. ^{1}H NMR spectrum (*δ*, acetone-d6, 600 MHz) of 4-chloro-2′-hydroxydihydrochalcone 3-*O*-*β*-D-(4′′-*O*-methyl)-glucopyranoside (**3c**)

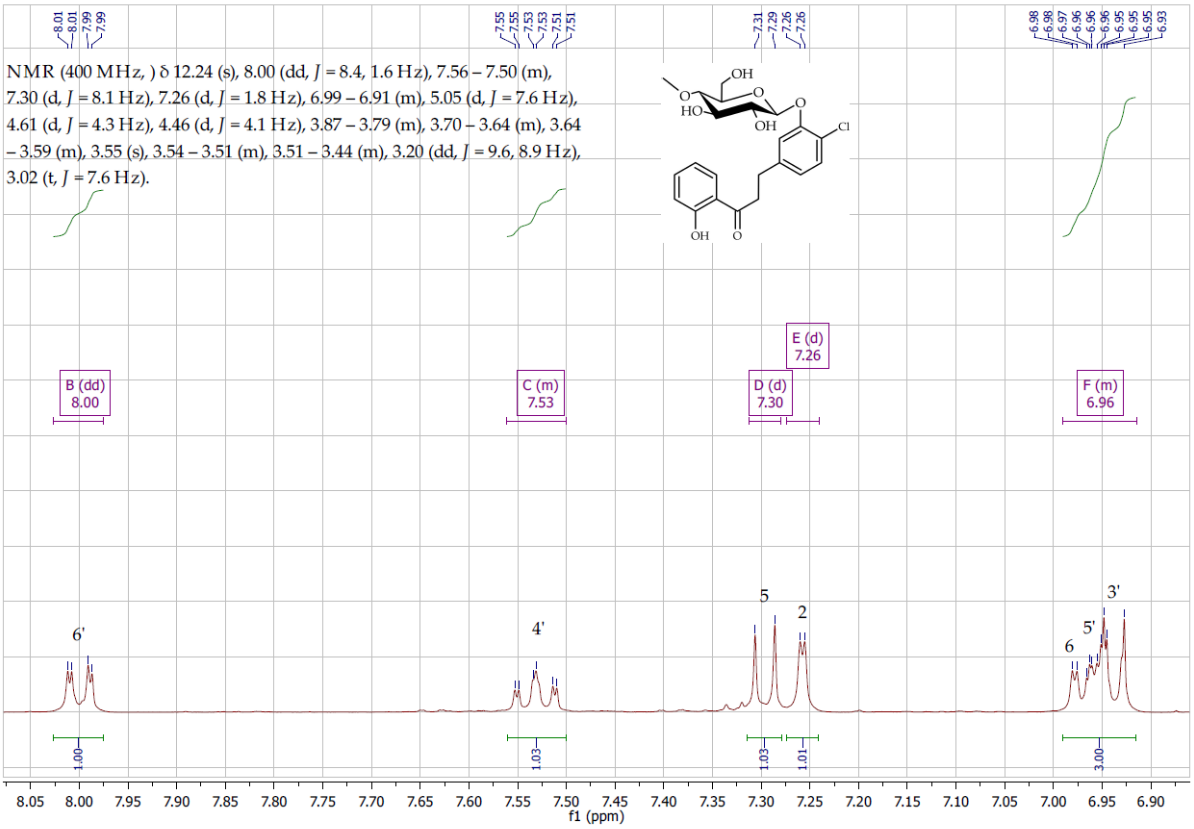

Figure S76. ^{1}H NMR spectrum expansion (*δ*, acetone-d6, 600 MHz) of 4-chloro-2′-hydroxydihydrochalcone 3-*O*-*β*-D-(4′′-*O*-methyl)-glucopyranoside (**3c**)

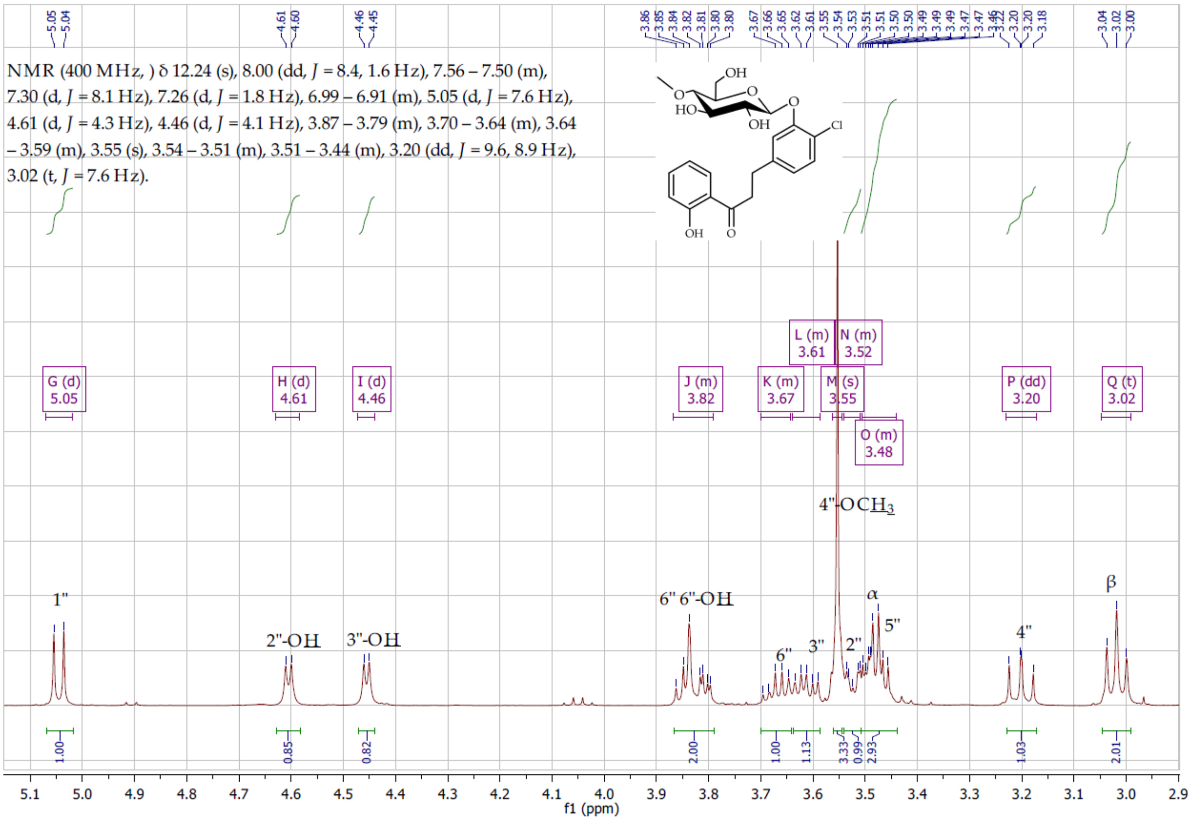

Figure S77. ^{1}H NMR spectrum expansion (*δ*, acetone-d6, 600 MHz) of 4-chloro-2′-hydroxydihydrochalcone 3-*O*-*β*-D-(4′′-*O*-methyl)-glucopyranoside (**3c**)

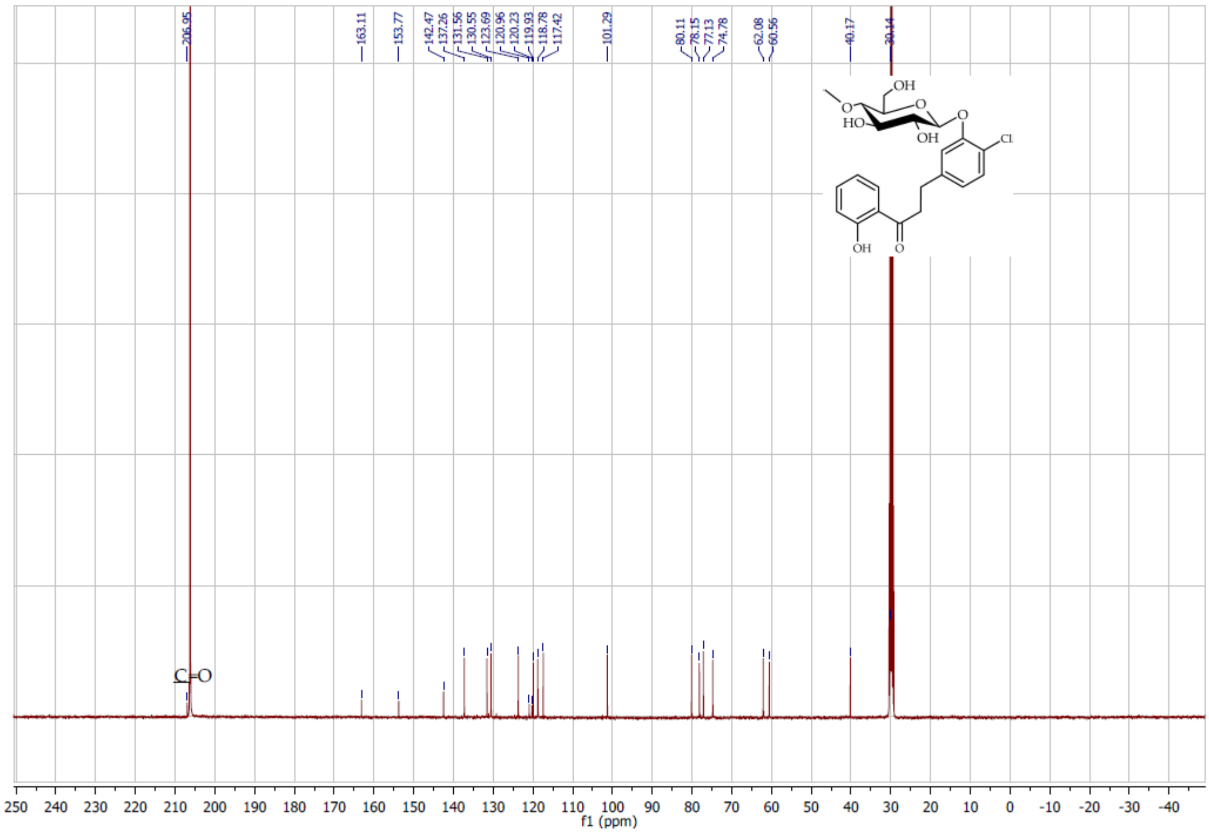

Figure S78. ^{13}C NMR spectrum (δ, acetone-d6, 151 MHz) of 4-chloro-2′-hydroxydihydrochalcone 3-*O*-β-D-(4′′-*O*-methyl)-glucopyranoside (**3c**)

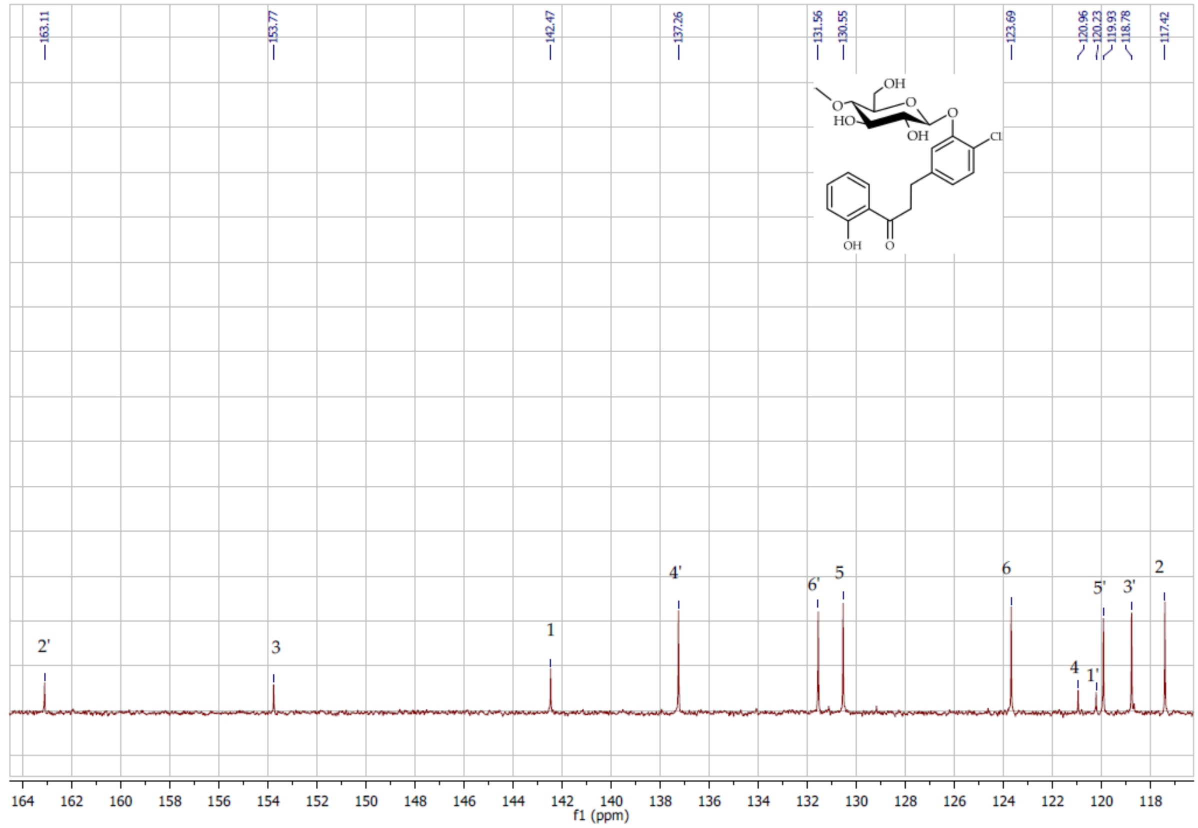

Figure S79. ^{13}C NMR spectrum expansion (δ, acetone-d6, 151 MHz) of 4-chloro-2′-hydroxydihydrochalcone 3-*O*-β-D-(4′′-*O*-methyl)-glucopyranoside (**3c**)

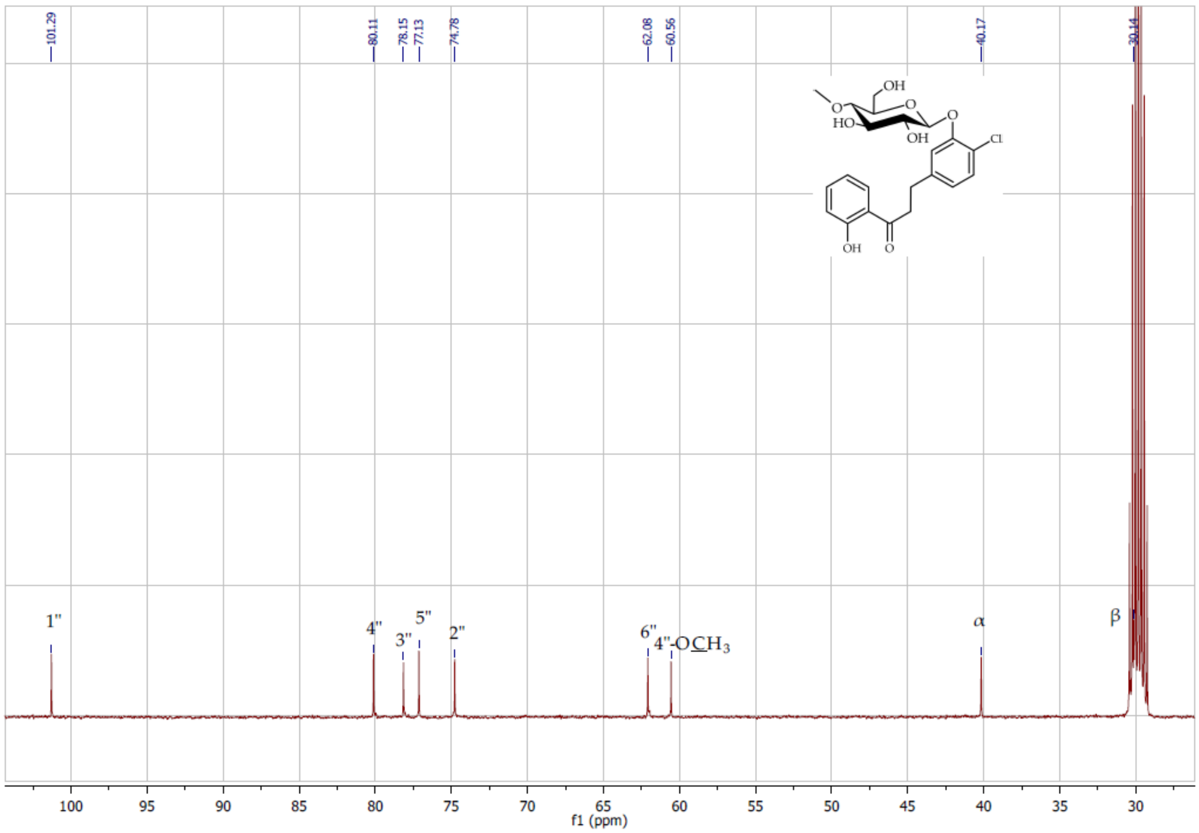

Figure S80. ^{13}C NMR spectrum expansion (*δ*, acetone-d6, 151 MHz) of 4-chloro-2′-hydroxydihydrochalcone 3-*O*-*β*-D-(4′′-*O*-methyl)-glucopyranoside (**3c**)

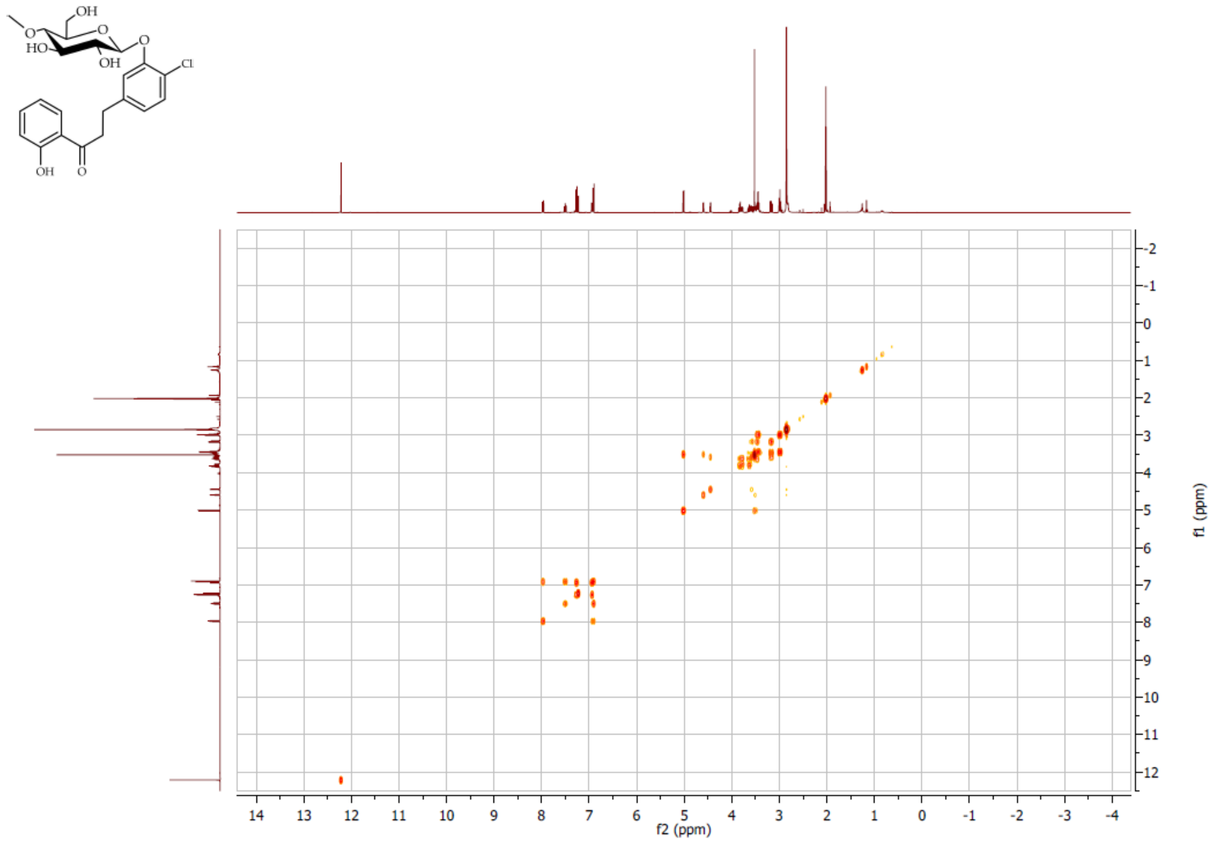

Figure S81. COSY contour map – ^{1}H x ^{1}H of 4-chloro-2′-hydroxydihydrochalcone 3-*O*-*β*-D-(4′′-*O*-methyl)-glucopyranoside (**3c**)

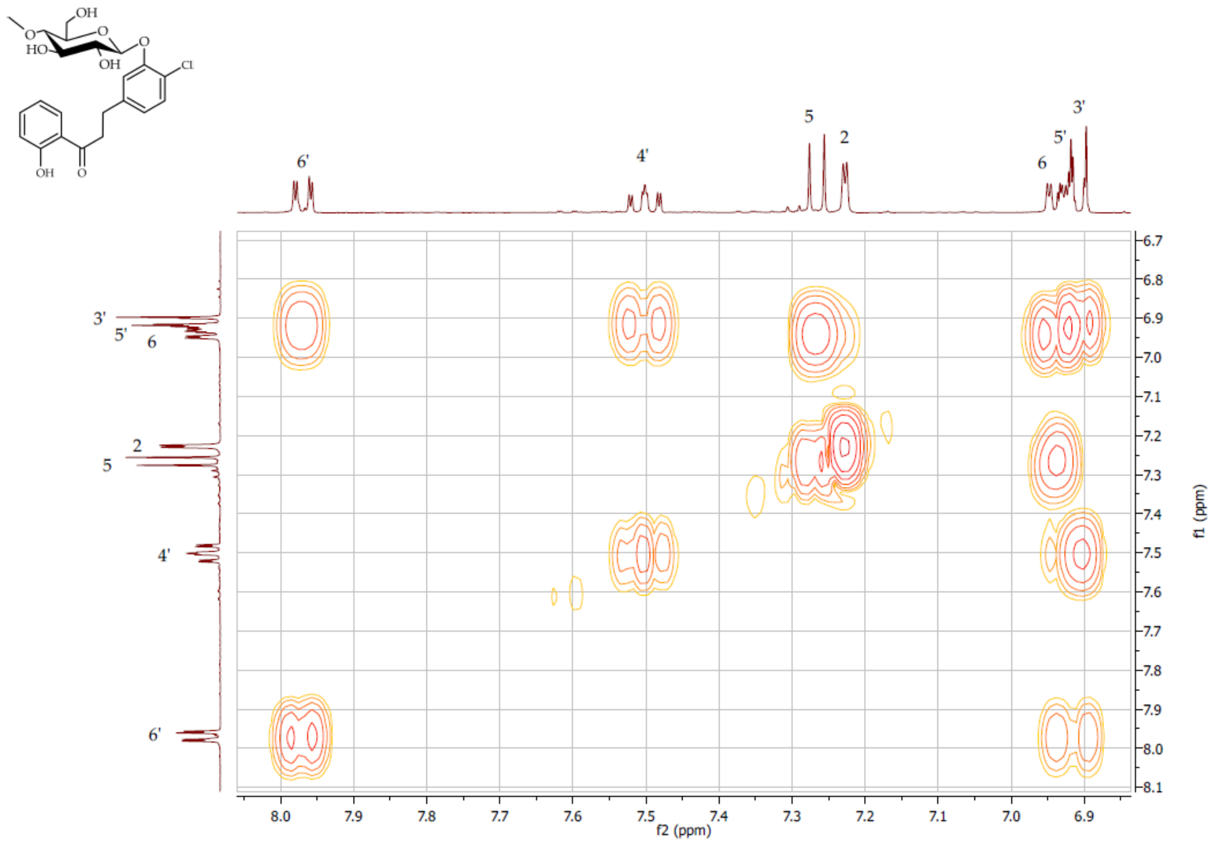

Figure S82. COSY contour map – ¹H x ¹H expansion of 4-chloro-2′-hydroxydihydrochalcone 3-*O*-*β*-D-(4′′-*O*-methyl)-glucopyranoside (**3c**)

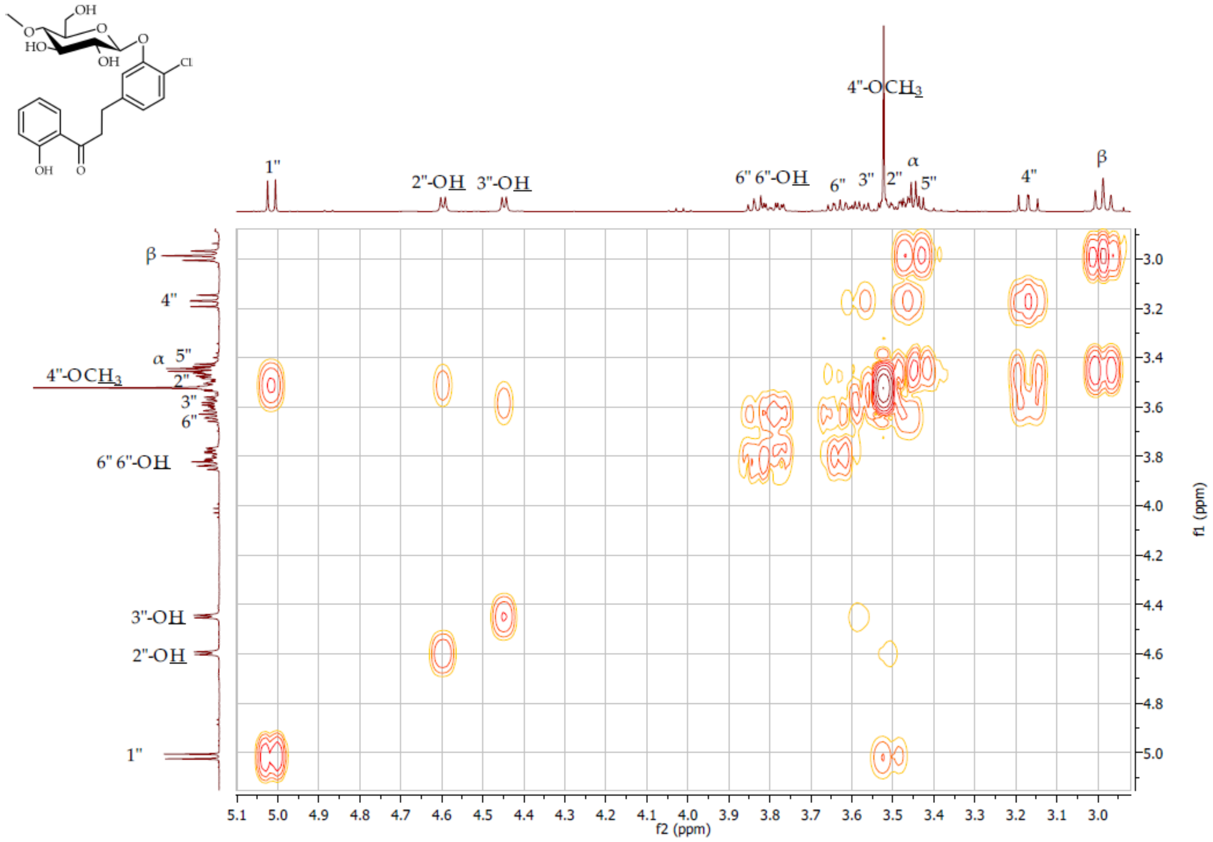

Figure S83. COSY contour map – ¹H x ¹H expansion of 4-chloro-2′-hydroxydihydrochalcone 3-*O*-*β*-D-(4′′-*O*-methyl)-glucopyranoside (**3c**)

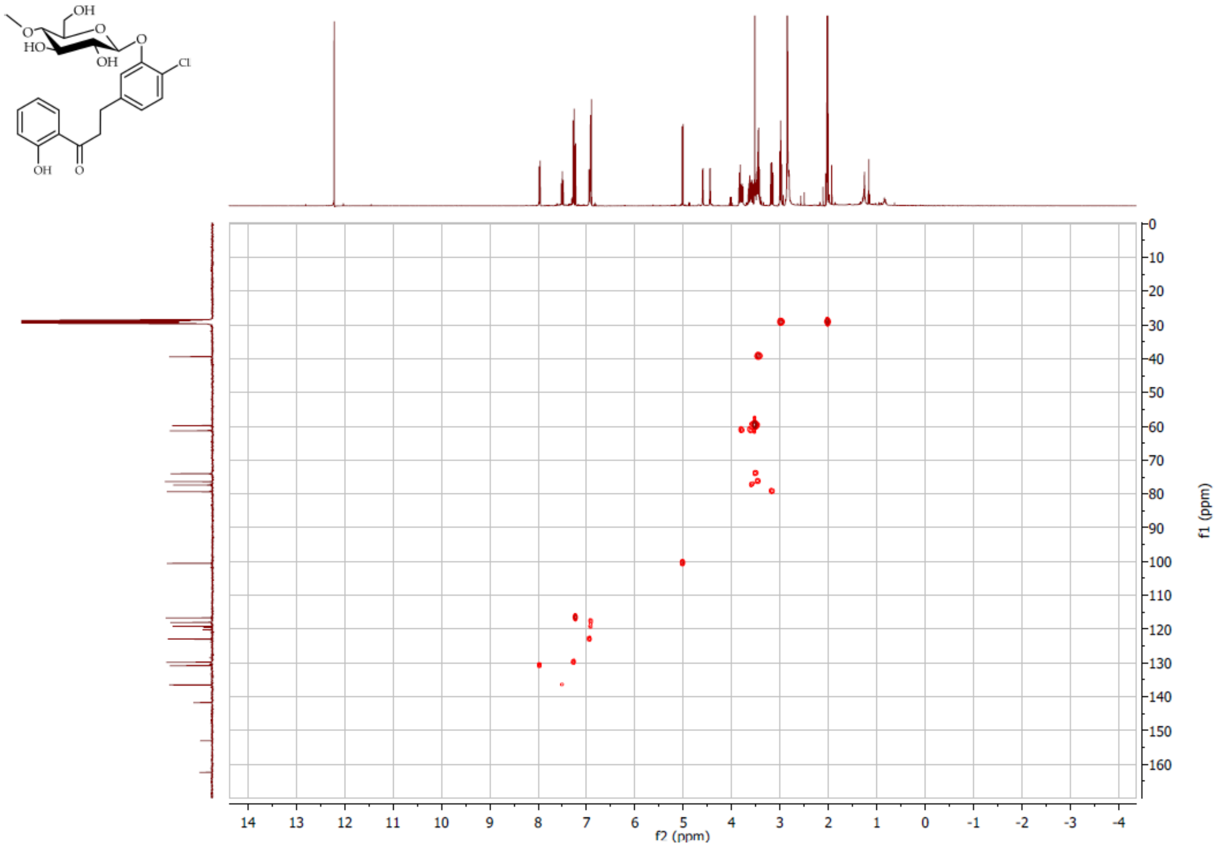

Figure S84. HMQC contour map – [1]H x ^{13}C of 4-chloro-2′-hydroxydihydrochalcone 3-*O*-*β*-D-(4′′-*O*-methyl)-glucopyranoside (**3c**)

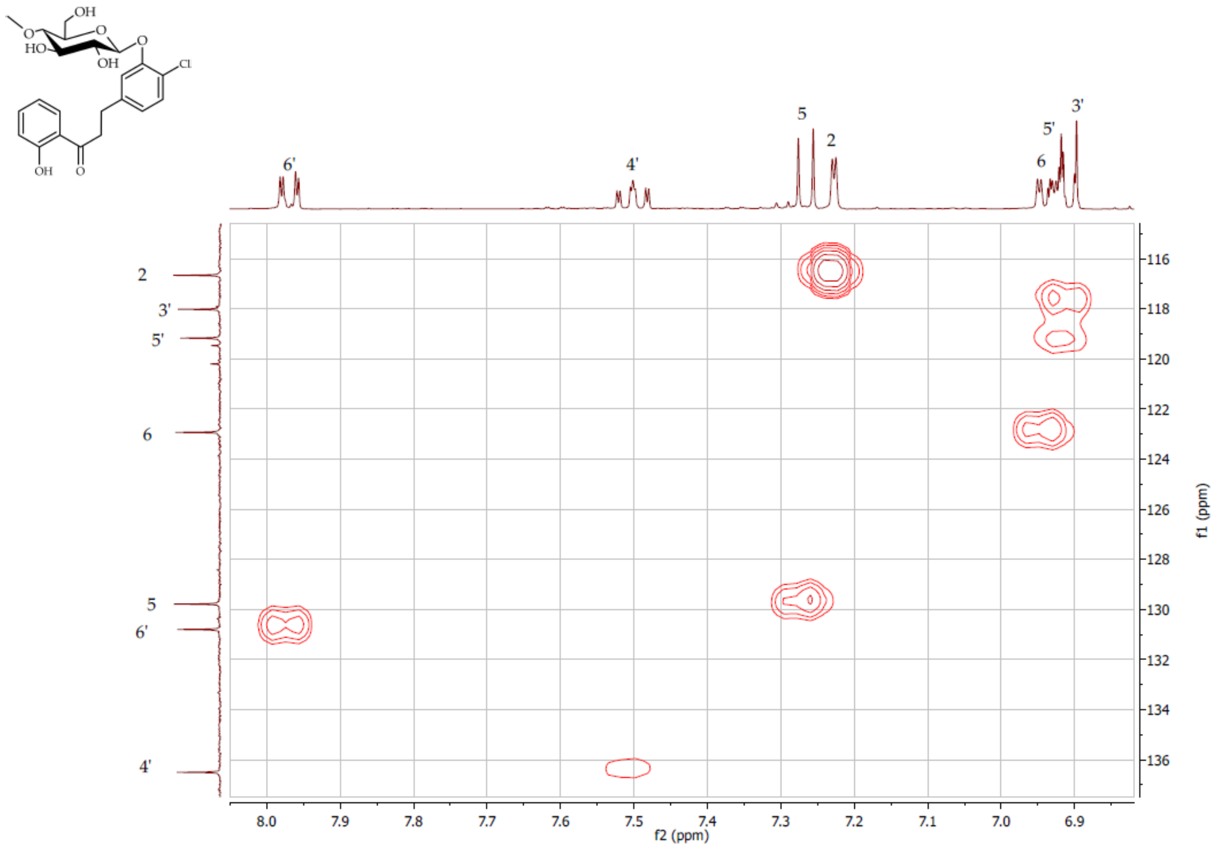

Figure S85. HMQC contour map – [1]H x ^{13}C expansion of 4-chloro-2′-hydroxydihydrochalcone 3-*O*-*β*-D-(4′′-*O*-methyl)-glucopyranoside (**3c**)

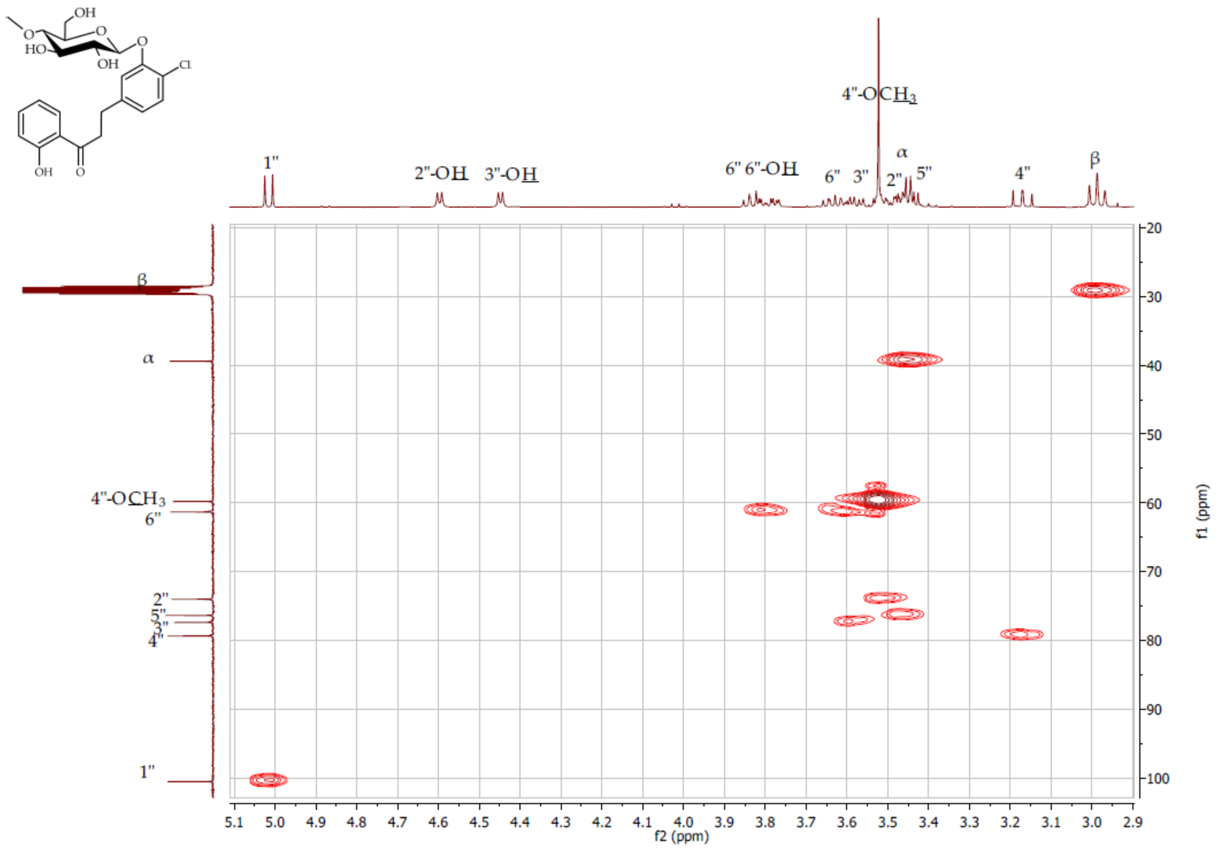

Figure S86. HMQC contour map – [1]H x ^{13}C expansion of 4-chloro-2′-hydroxydihydrochalcone 3-*O*-*β*-D-(4′′-*O*-methyl)-glucopyranoside (**3c**)

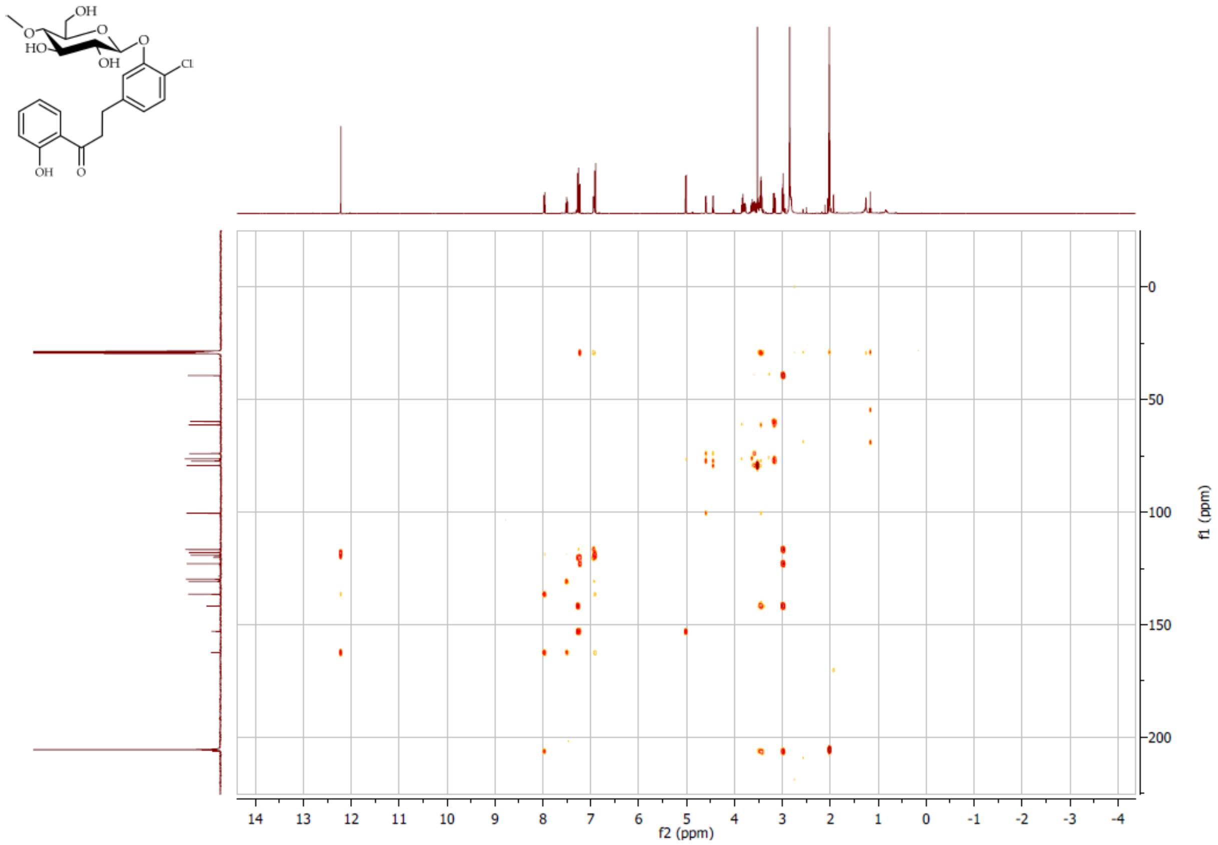

Figure S87. HMBC contour map – ^{1}H x ^{13}C of 4-chloro-2′-hydroxydihydrochalcone 3-*O*-*β*-D-(4′′-*O*-methyl)-glucopyranoside (**3c**)

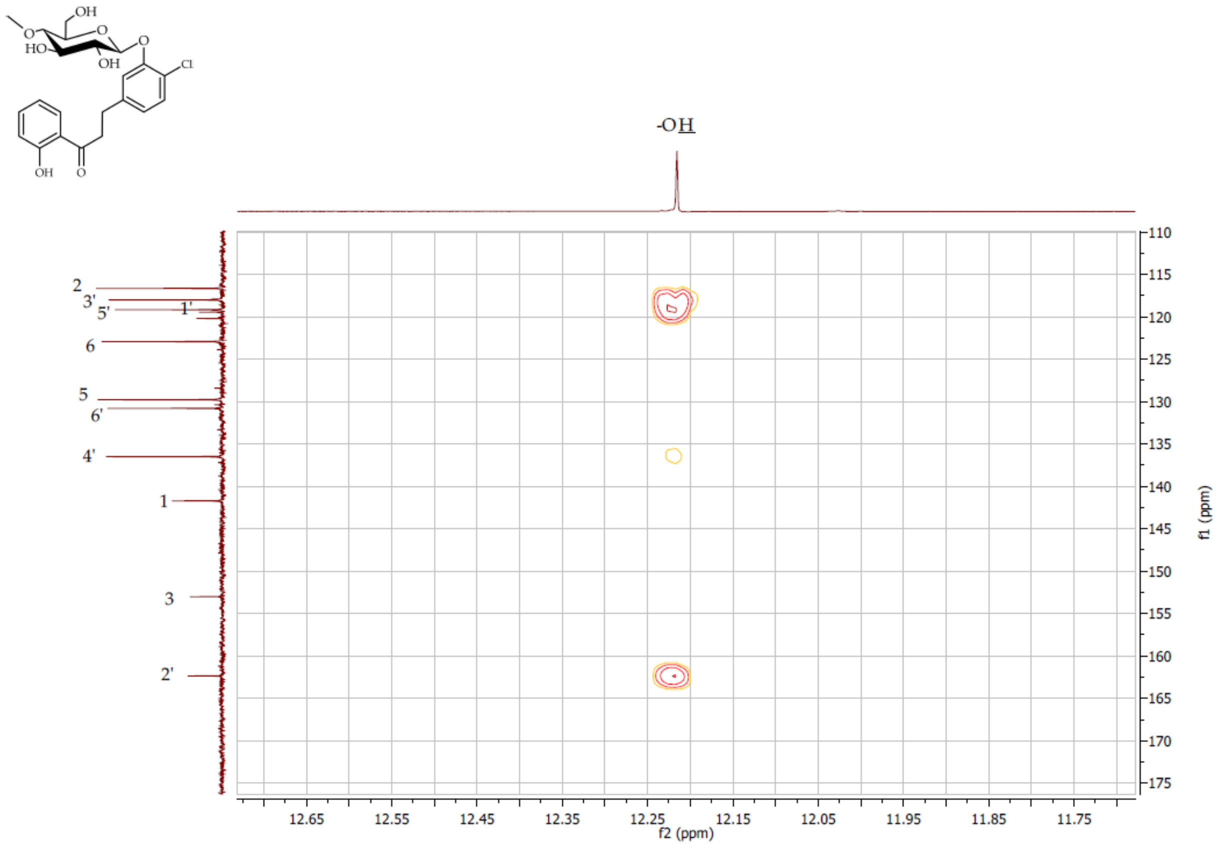

Figure S88. HMBC contour map – ^{1}H x ^{13}C expansion of 4-chloro-2′-hydroxydihydrochalcone 3-*O*-*β*-D-(4′′-*O*-methyl)-glucopyranoside (**3c**)

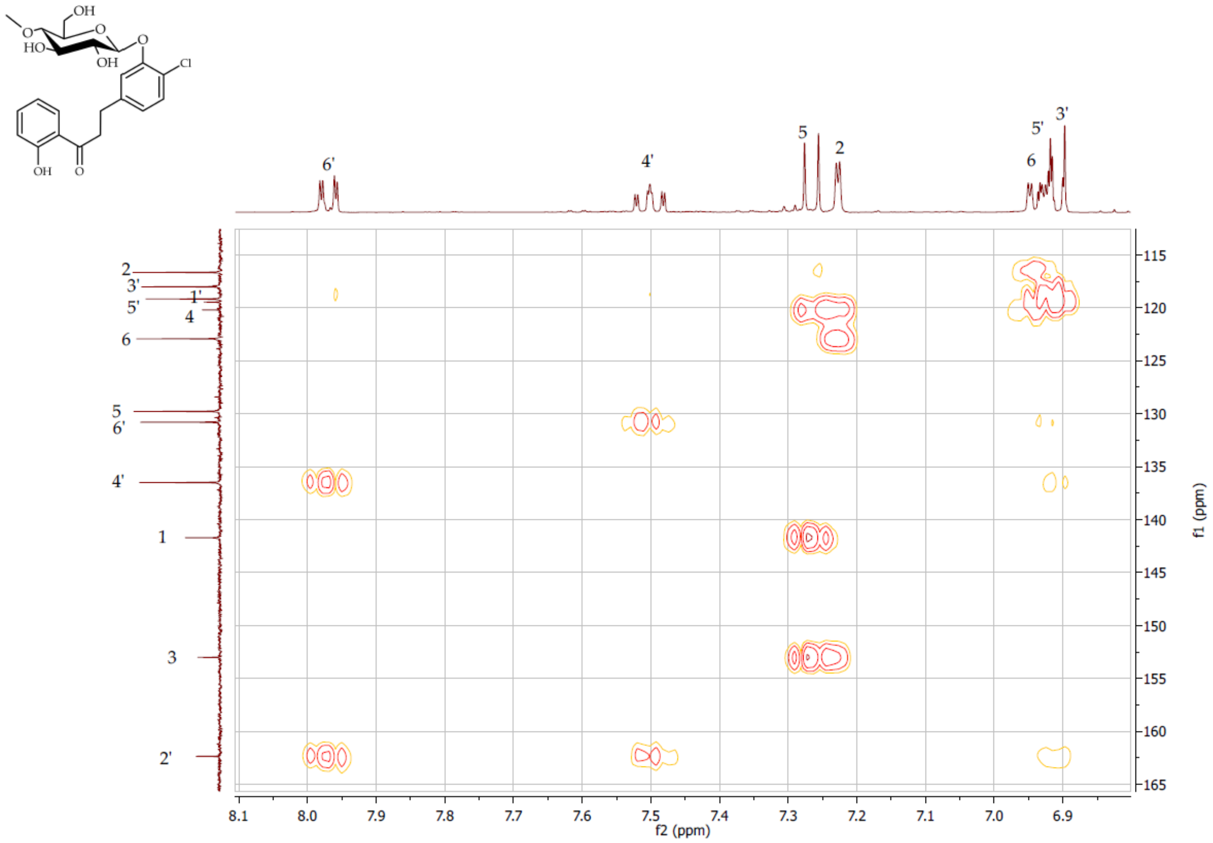

Figure S89. HMBC contour map – ^{1}H x ^{13}C expansion of 4-chloro-2′-hydroxydihydrochalcone 3-*O*-*β*-D-(4′′-*O*-methyl)-glucopyranoside (**3c**)

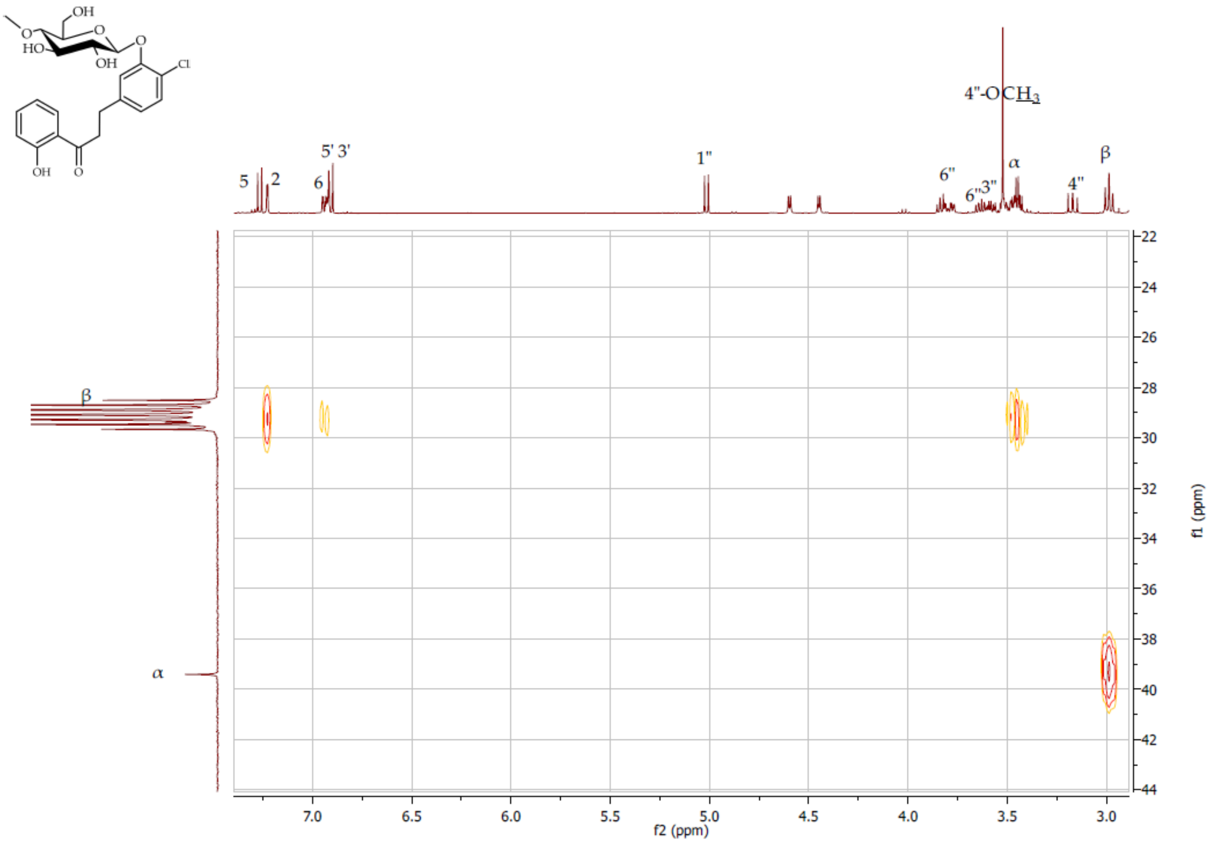

Figure S90. HMBC contour map – ^{1}H x ^{13}C expansion of 4-chloro-2′-hydroxydihydrochalcone 3-*O*-*β*-D-(4′′-*O*-methyl)-glucopyranoside (**3c**)

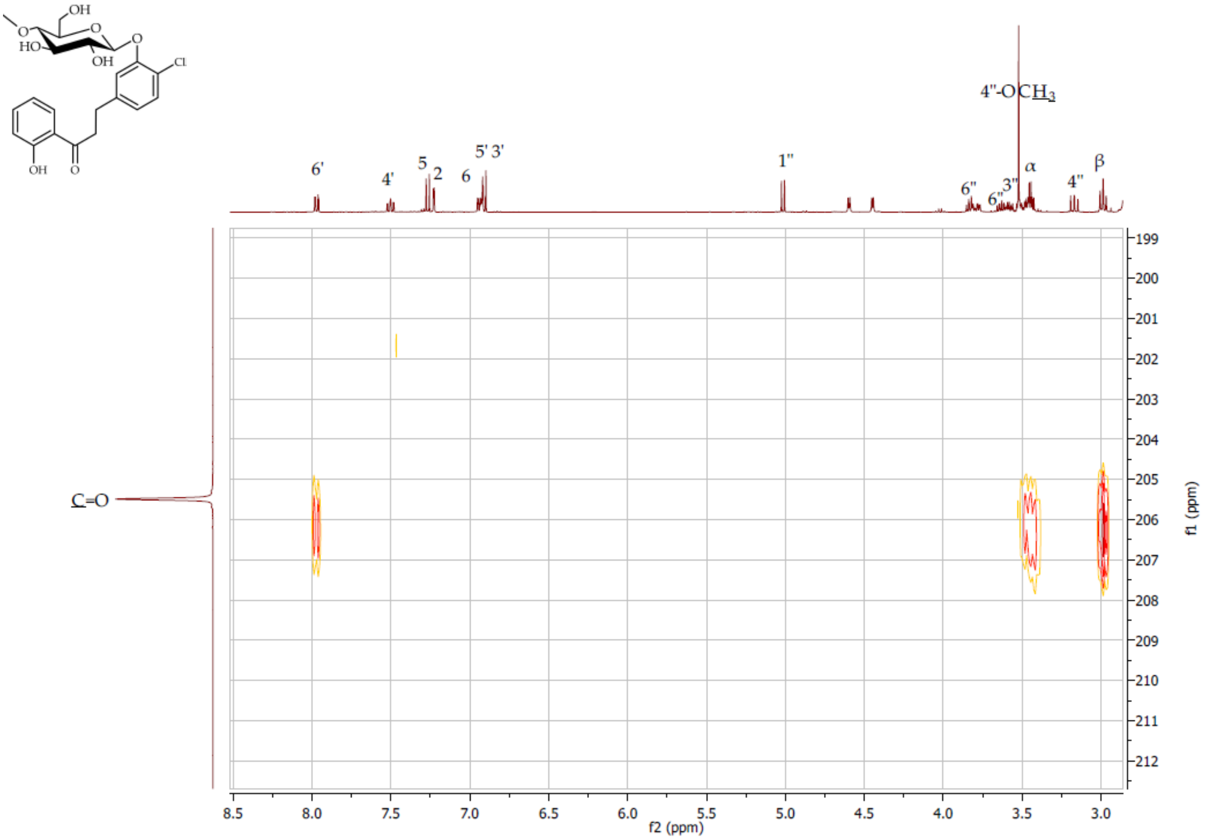

Figure S91. HMBC contour map – ^{1}H x ^{13}C expansion of 4-chloro-2′-hydroxydihydrochalcone 3-*O*-*β*-D-(4′′-*O*-methyl)-glucopyranoside (**3c**)

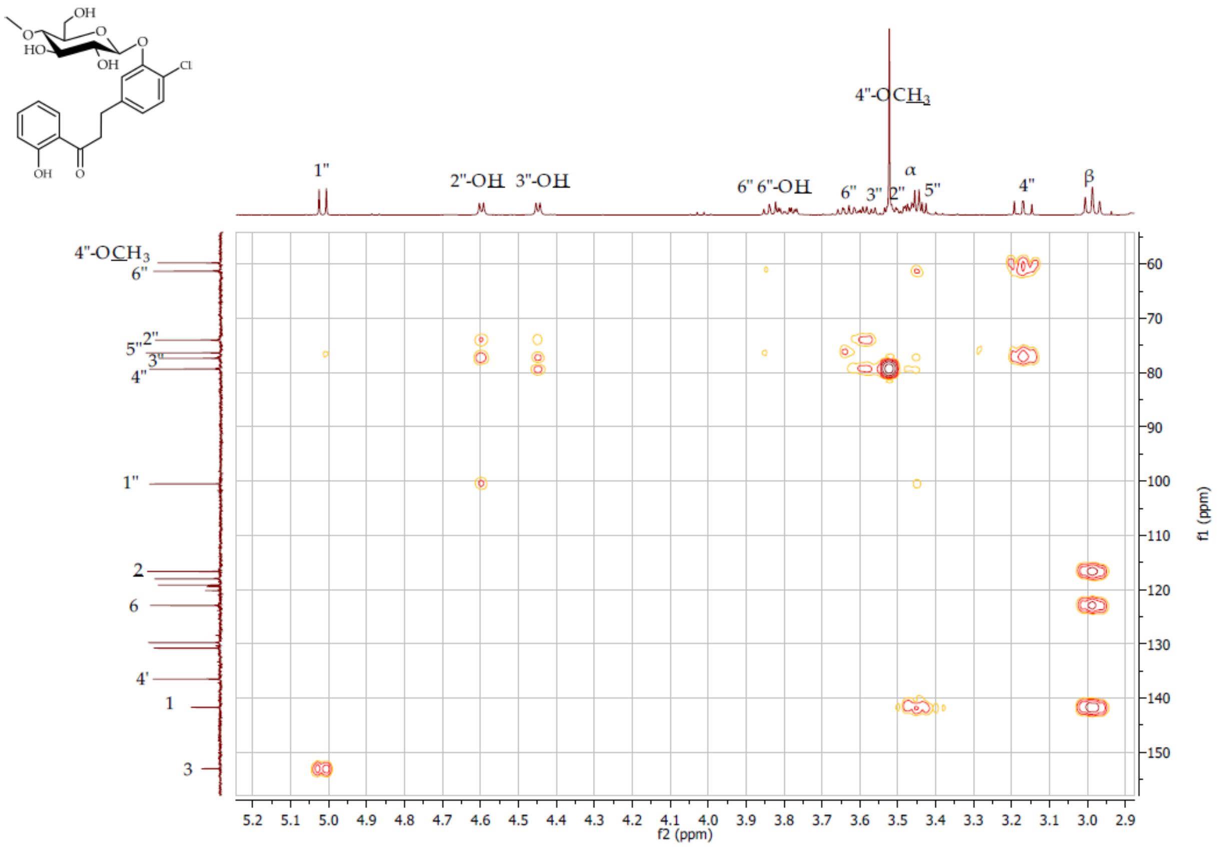

Figure S92. HMBC contour map – ^{1}H x ^{13}C expansion of 4-chloro-2′-hydroxydihydrochalcone 3-*O*-*β*-D-(4′′-*O*-methyl)-glucopyranoside (**3c**)

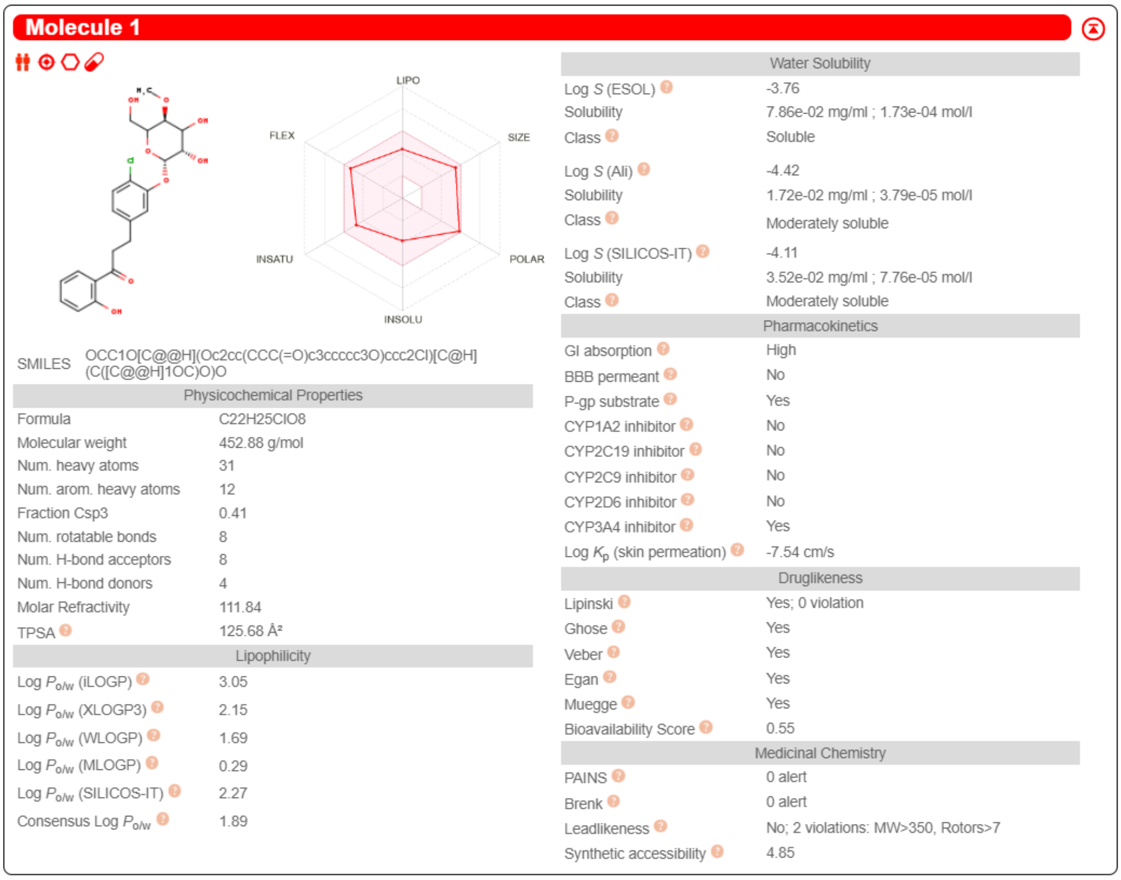

Figure S93. 4-Chloro-2′-hydroxydihydrochalcone 3-*O*-*β*-D-(4′′-*O*-methyl)-glucopyranoside (**3c**) physicochemical and ADME parameters prediction using the SwissADME modelling

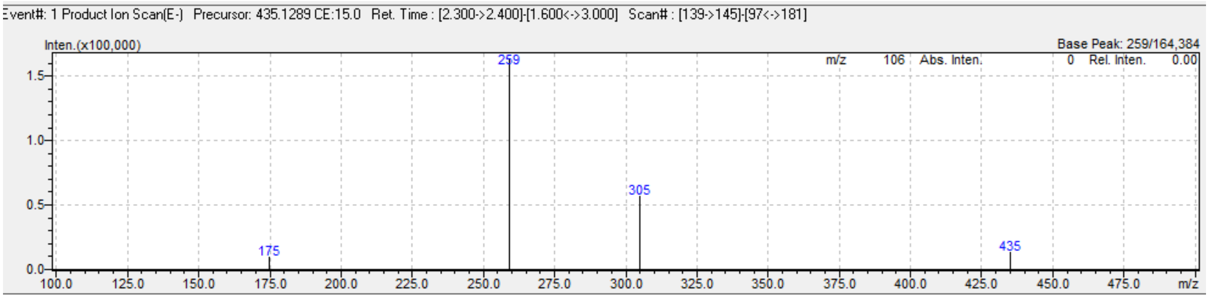

Figure S94. MS analysis of 5′-chlorodihydrochalcone 2′-*O*-*β*-D-(4′′-*O*-methyl)-glucopyranoside (**6a**)

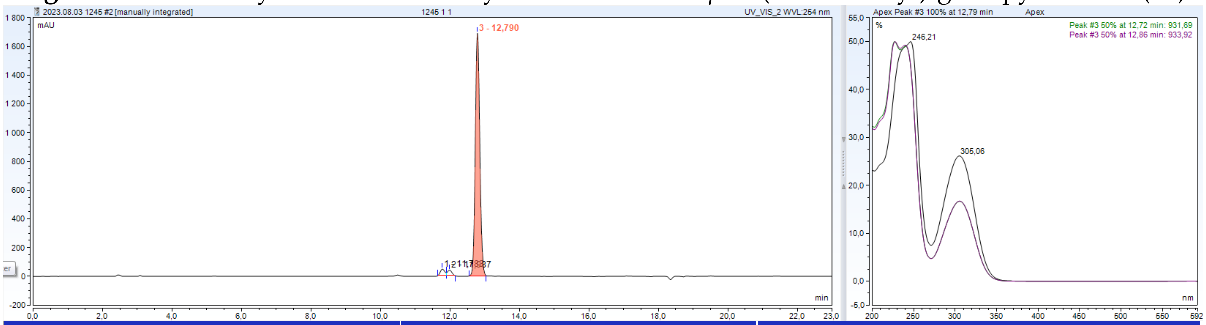

Peak No.	Peak Name	Ret.Time min	Rel.Area %	Area mAU*min	Height mAU	Match	RSD Match %	PPI nm	RSD PPI %
1		11,783	2,49	6,5172	45,34	1000	0,01	214,4	0,05
2		11,987	2,19	5,7189	38,21	997	0,49	215,1	0,62
3		*12,790*	*95,32*	*249,3451*	*1687,39*	*858*	*19,18*	*234,5*	*4,78*
Maximum			95,32	249,3451	1687,39	1000	19,18		4,78
Minimum			2,19	5,7189	38,21	858	0,01		0,05

Figure S95. HPLC analysis of 5′-chlorodihydrochalcone 2′-*O*-*β*-D-(4′′-*O*-methyl)-glucopyranoside (**6a**)

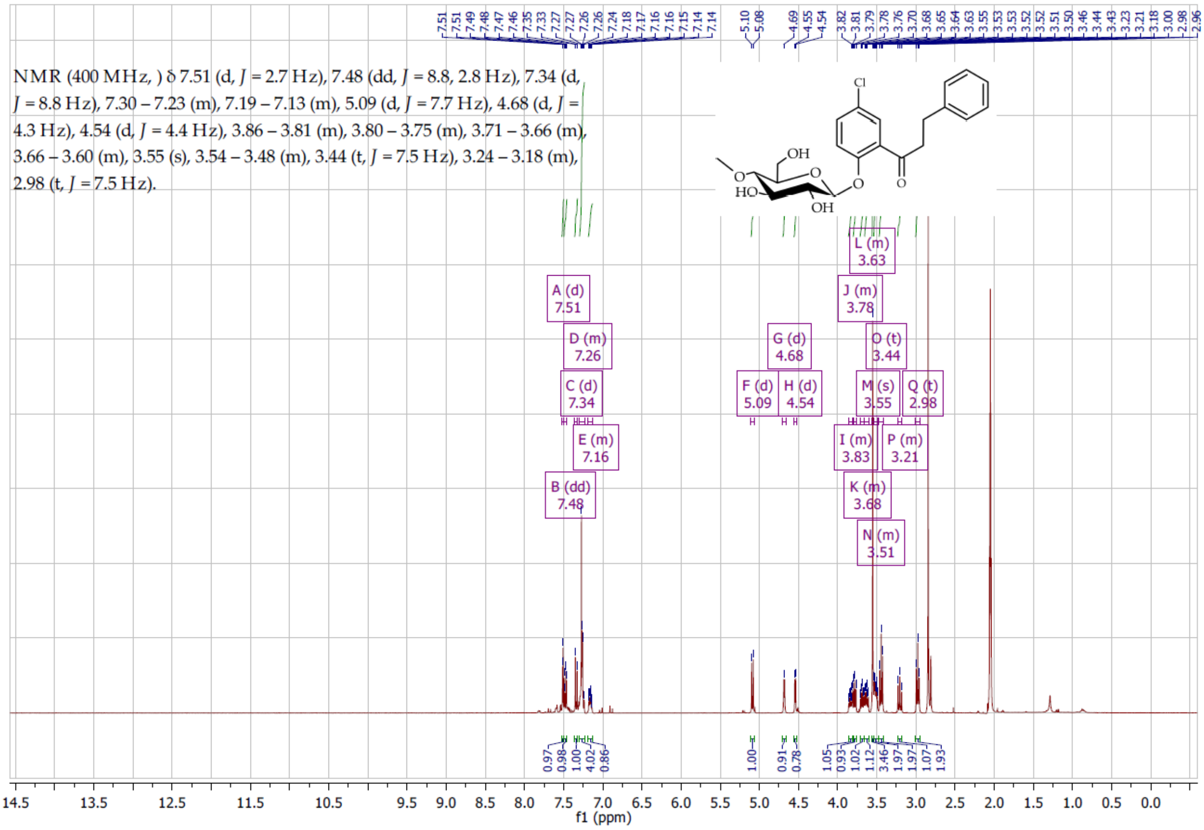

Figure S96. ^{1}H NMR spectrum (*δ*, acetone-d6, 600 MHz) of 5′-chlorodihydrochalcone 2′-*O*-*β*-D-(4′′-*O*-methyl)-glucopyranoside (**6a**)

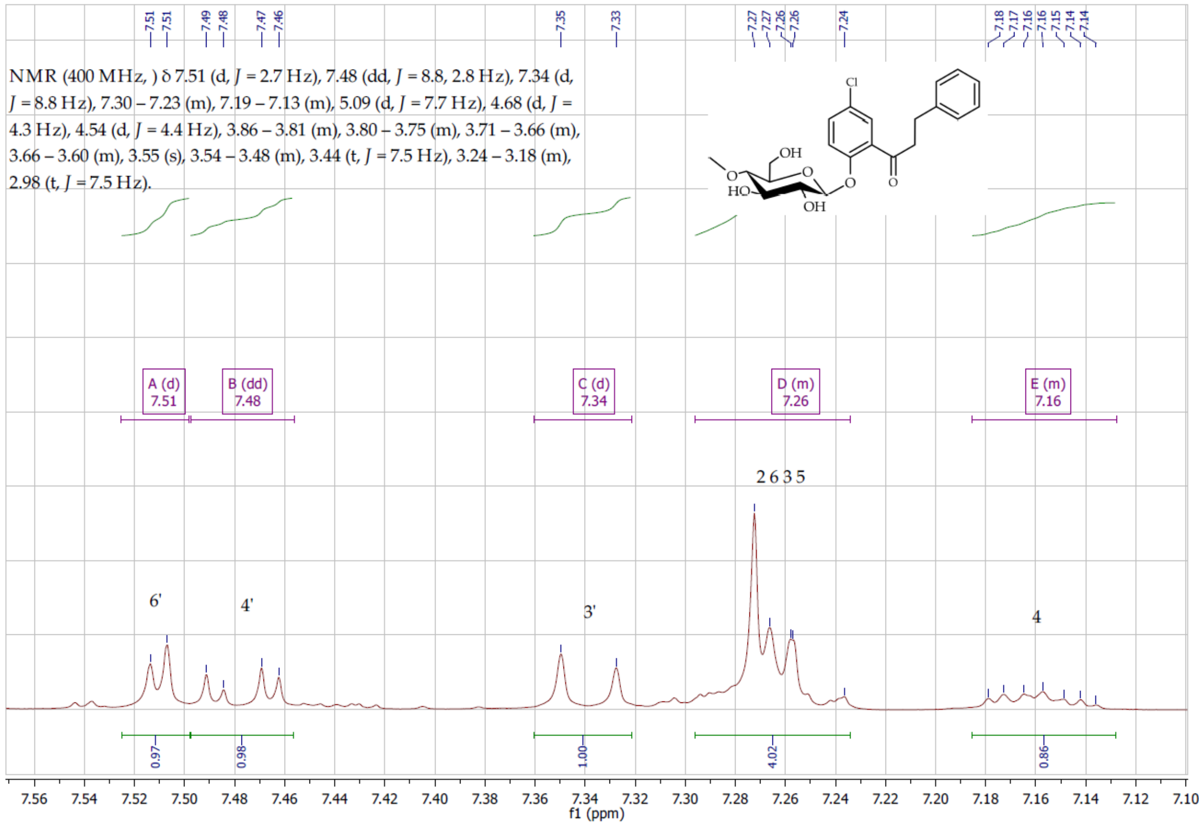

Figure S97. ^{1}H NMR spectrum expansion (*δ*, acetone-d6, 600 MHz) of 5′-chlorodihydrochalcone 2′-*O*-*β*-D-(4′′-*O*-methyl)-glucopyranoside (**6a**)

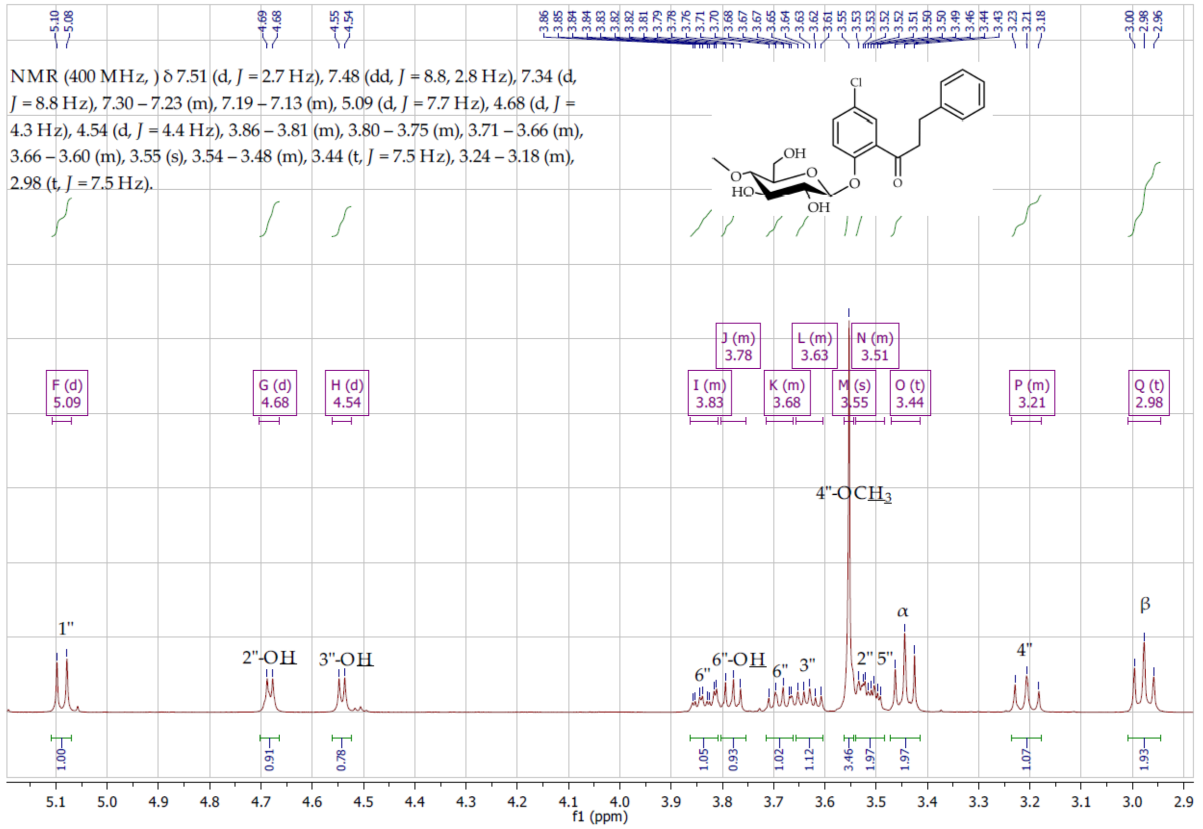

Figure S98. ^{1}H NMR spectrum expansion (*δ*, acetone-d6, 600 MHz) of 4-chlorodihydrochalcone 2′-*O*-*β*-D-(4′′-*O*-methyl)-glucopyranoside (**6a**)

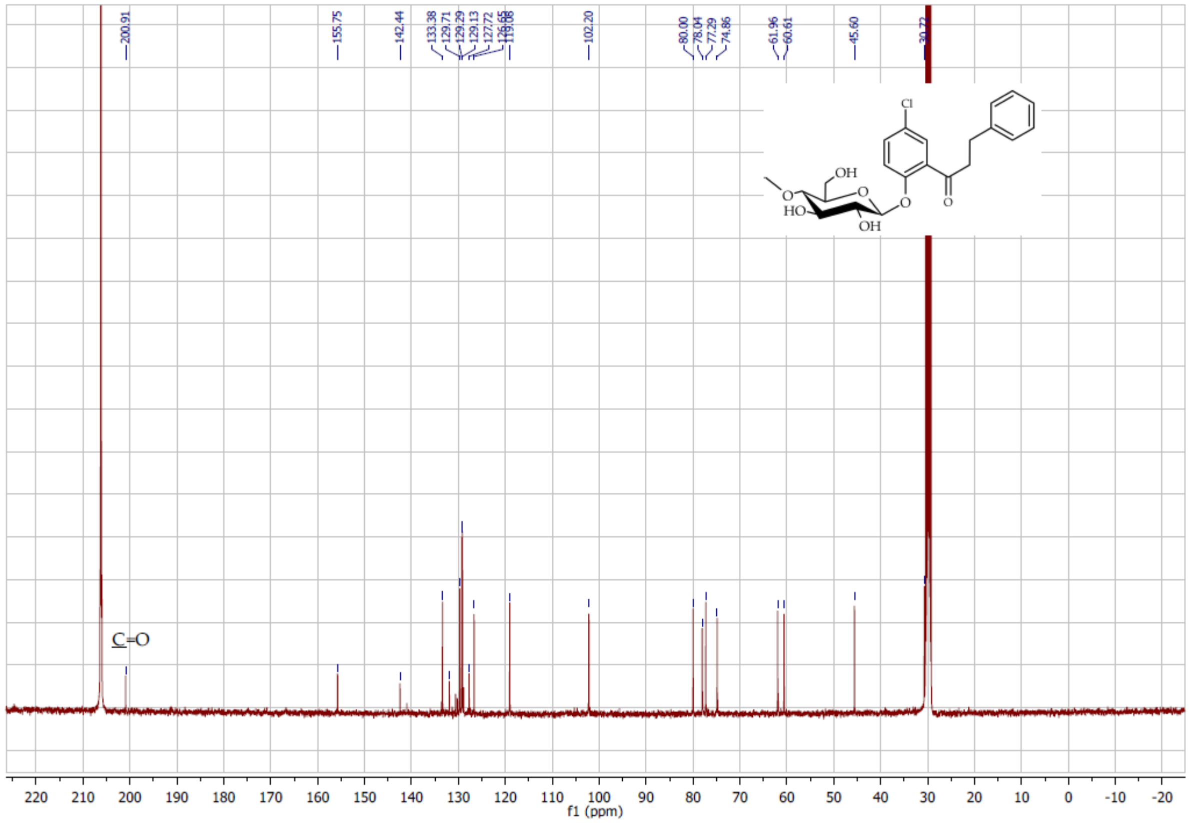

Figure S99. ^{13}C NMR spectrum (*δ*, acetone-d6, 151 MHz) of 5′-chlorodihydrochalcone 2′-*O*-*β*-D-(4′′-*O*-methyl)-glucopyranoside (**6a**)

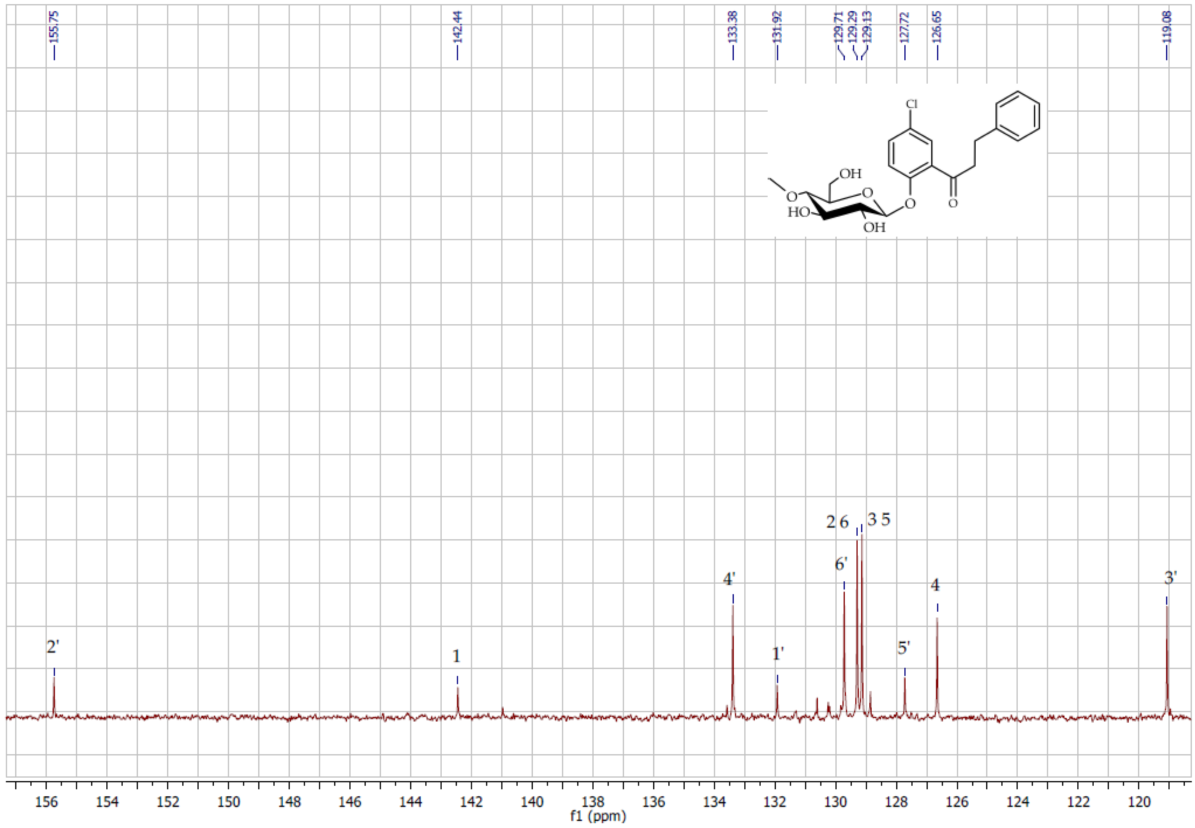

Figure S100. ^{13}C NMR spectrum expansion (*δ*, acetone-d6, 151 MHz) of 5′-chlorodihydrochalcone 2′-*O*-*β*-D-(4′′-*O*-methyl)-glucopyranoside (**6a**)

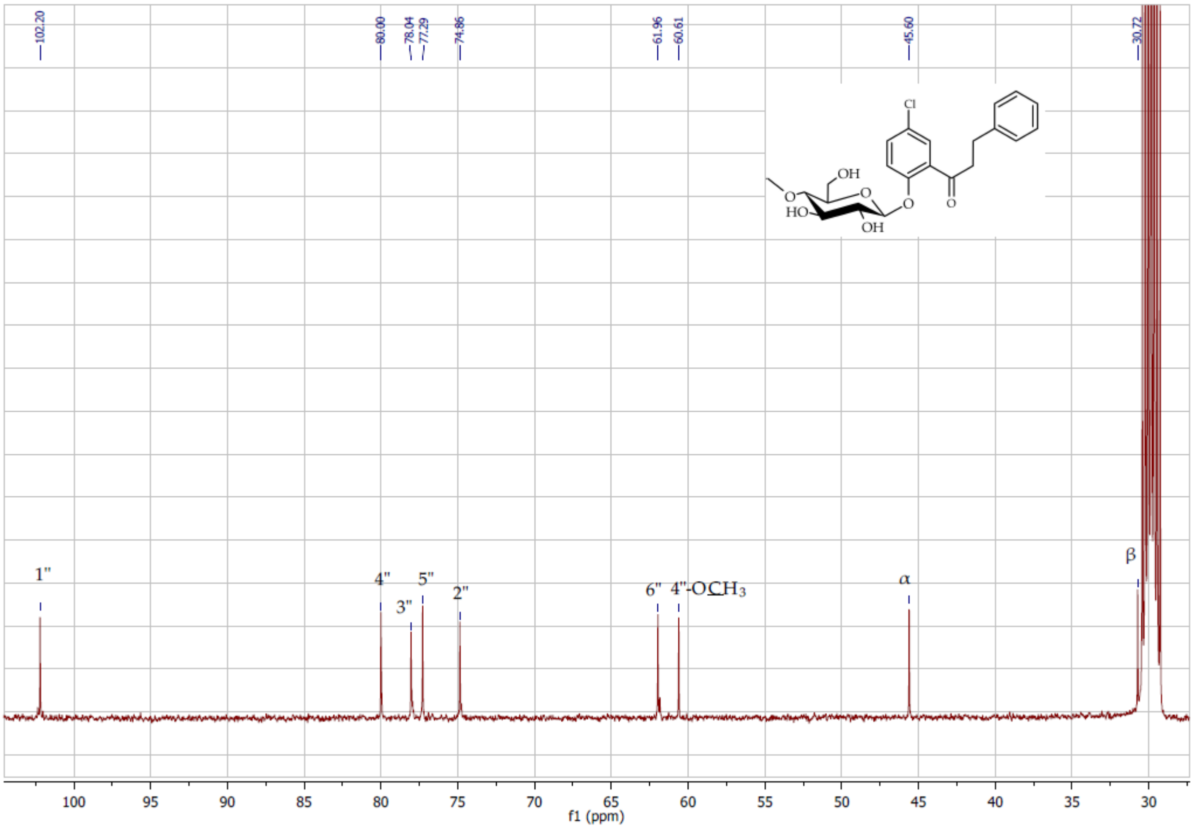

Figure S101. ^{13}C NMR spectrum expansion (*δ*, acetone-d6, 151 MHz) of 5′-chlorodihydrochalcone 2′-*O*-*β*-D-(4′′-*O*-methyl)-glucopyranoside (**6a**)

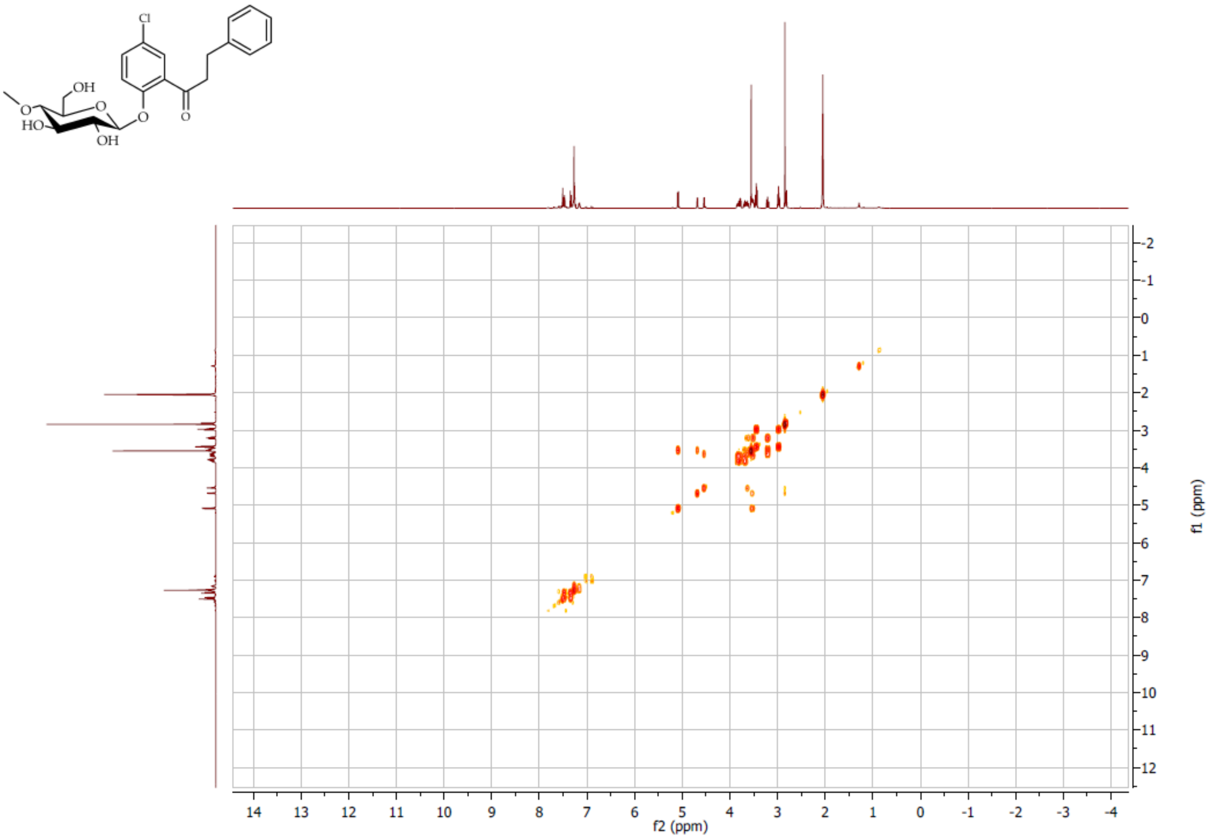

Figure S102. COSY contour map – ^{1}H x ^{1}H of 5′-chlorodihydrochalcone 2′-*O*-*β*-D-(4′′-*O*-methyl)-glucopyranoside (**6a**)

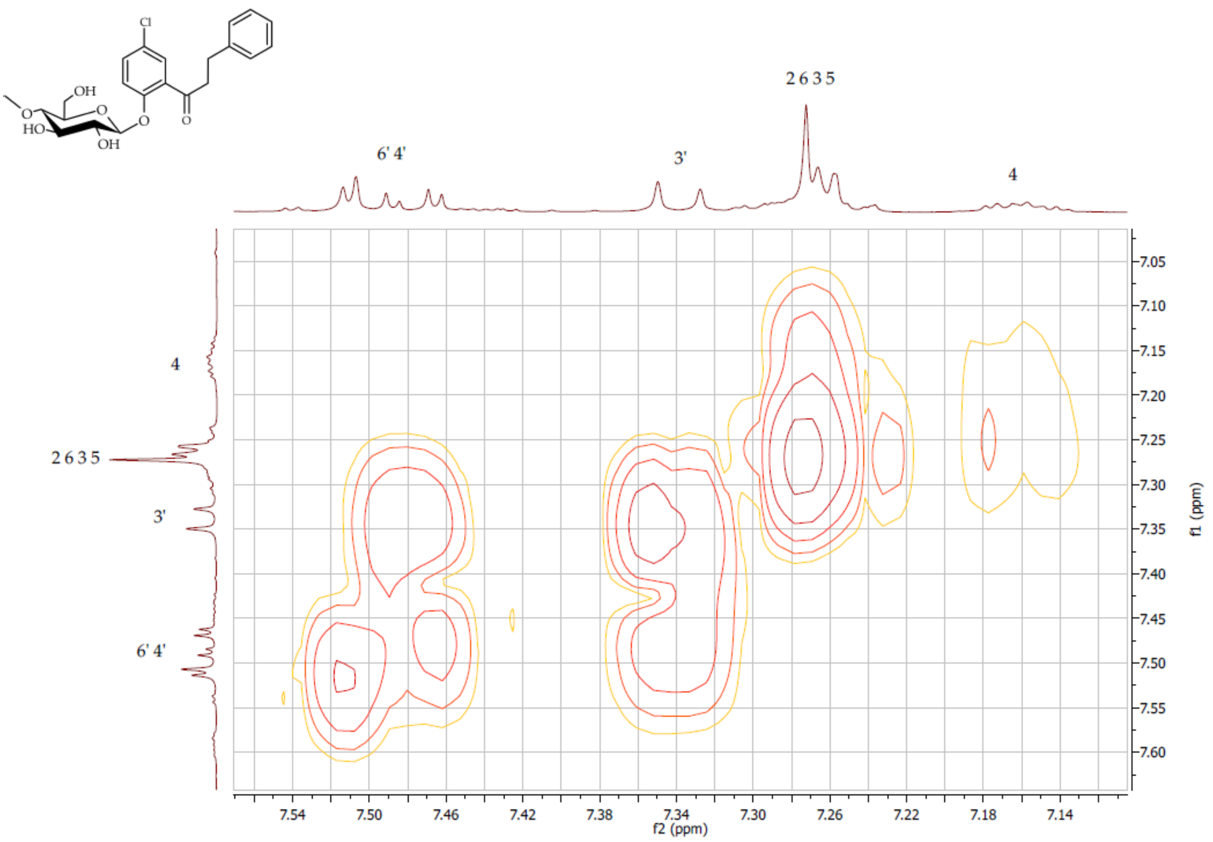

Figure S103. COSY contour map – ^{1}H x ^{1}H expansion of 5′-chlorodihydrochalcone 2′-*O*-*β*-D-(4′′-*O*-methyl)-glucopyranoside (**6a**)

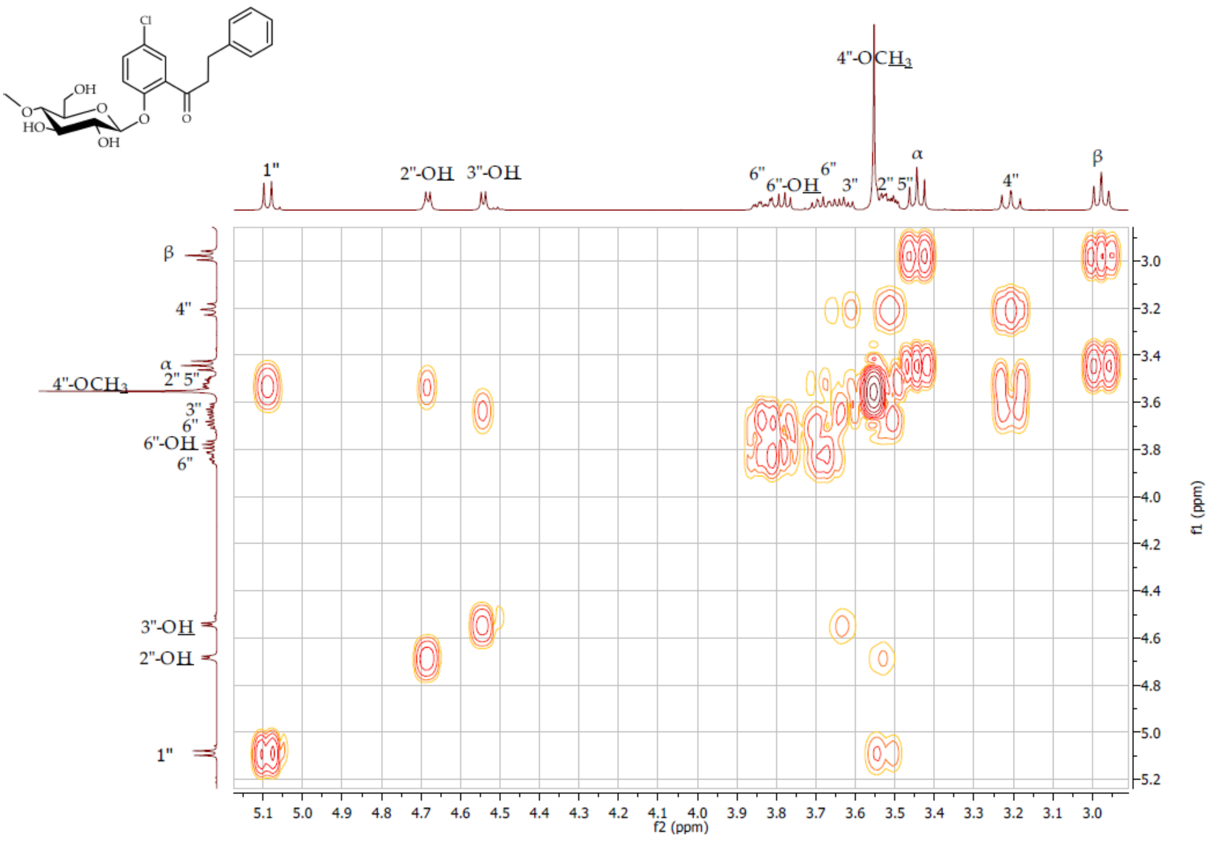

Figure S104. COSY contour map – ^{1}H x ^{1}H expansion of 4-chlorodihydrochalcone 2′-*O*-*β*-D-(4′′-*O*-methyl)-glucopyranoside (**6a**)

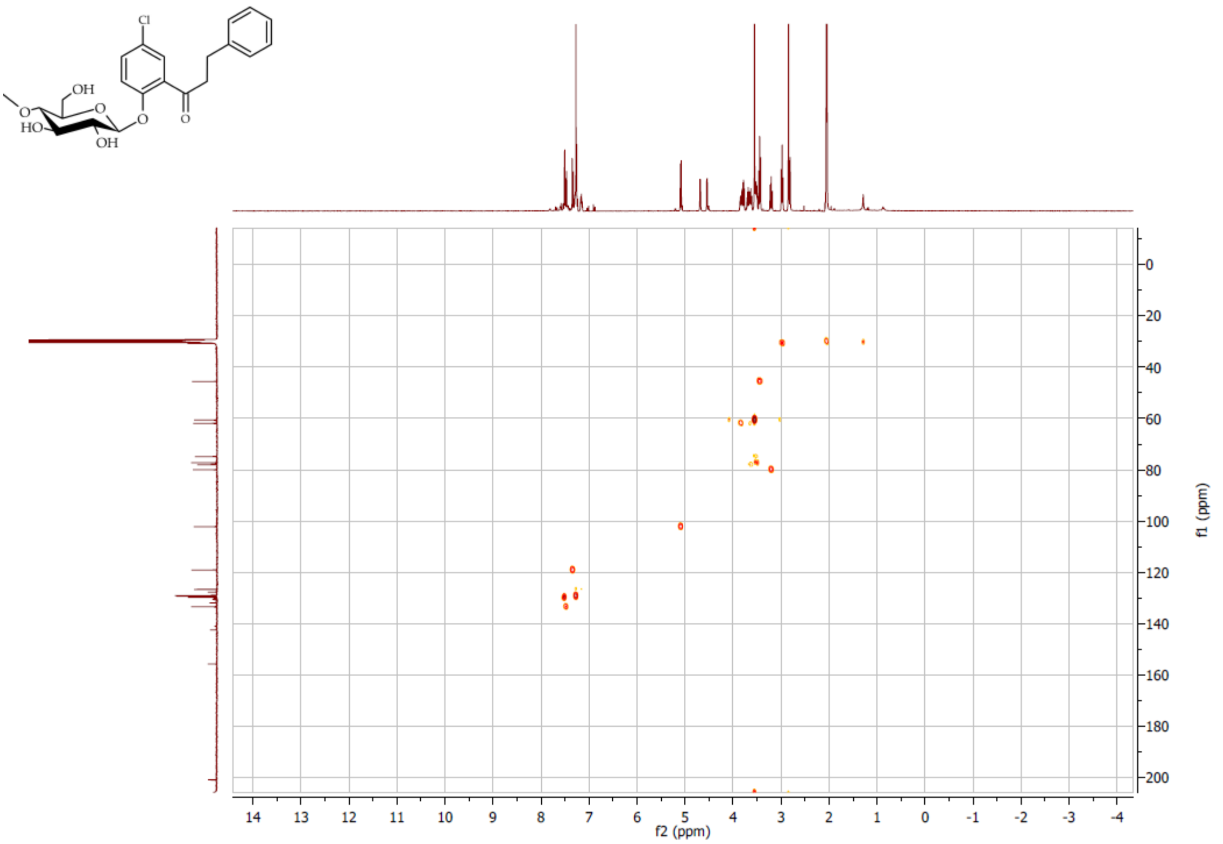

Figure S105. HMQC contour map – [1]H x ^{13}C of 5′-chlorodihydrochalcone 2′-*O*-*β*-D-(4′′-*O*-methyl)-glucopyranoside (**6a**)

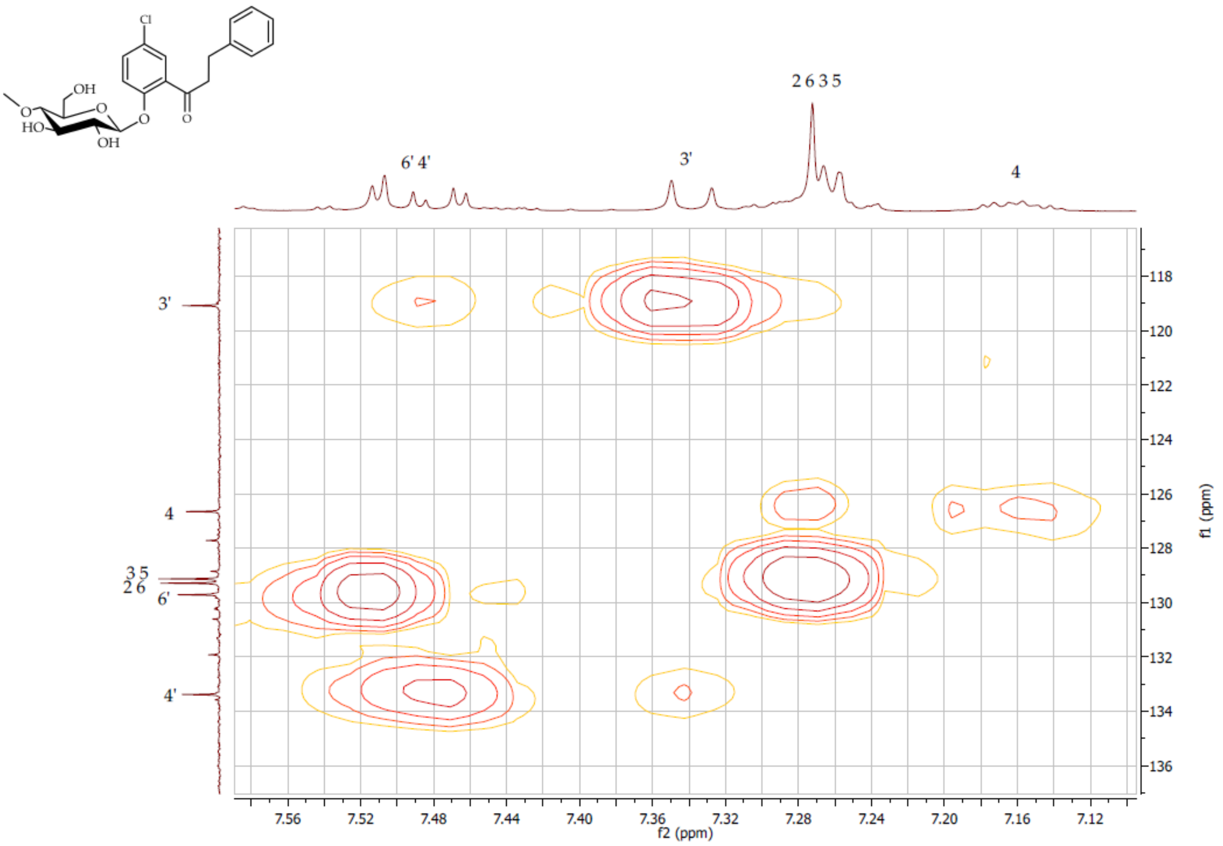

Figure S106. HMQC contour map – [1]H x ^{13}C expansion of 5′-chlorodihydrochalcone 2′-*O*-*β*-D-(4′′-*O*-methyl)-glucopyranoside (**6a**)

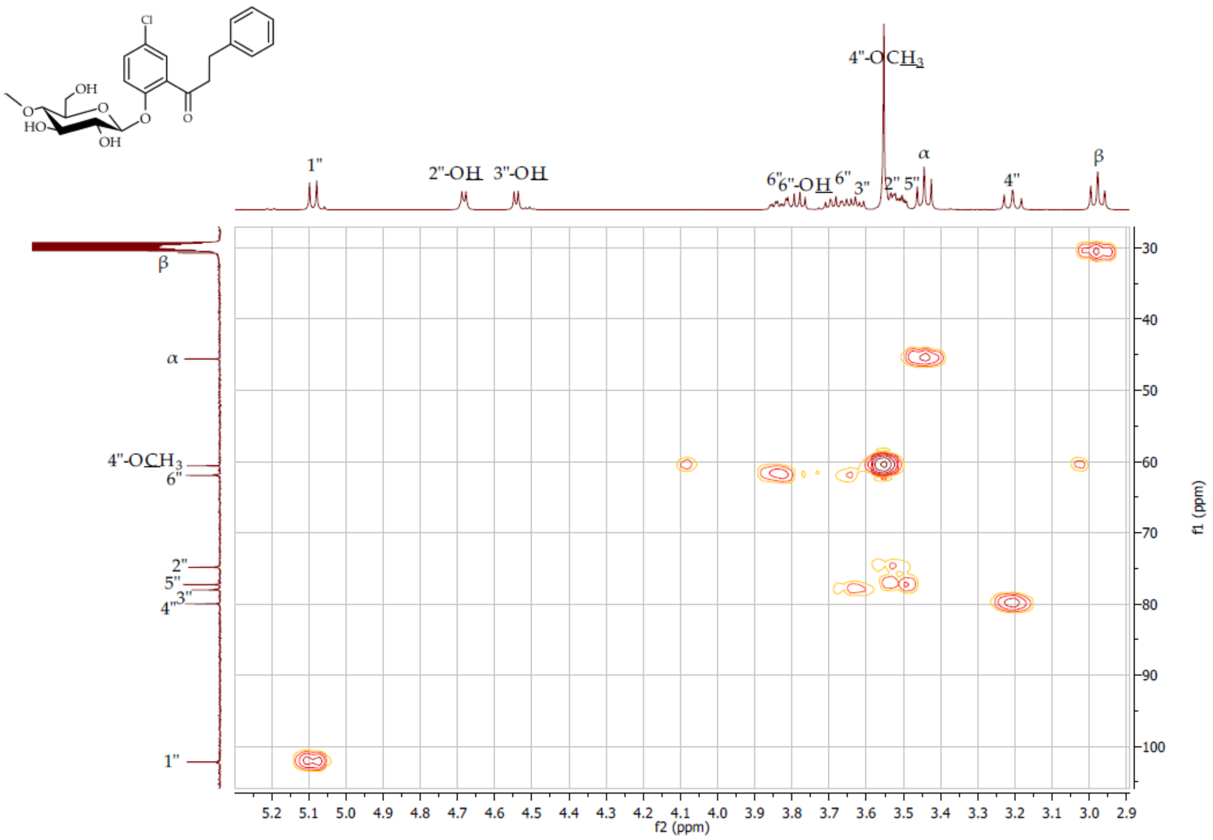

Figure S107. HMQC contour map – [1]H x ^{13}C expansion of 5′-chlorodihydrochalcone 2′-*O*-*β*-D-(4′′-*O*-methyl)-glucopyranoside (**6a**)

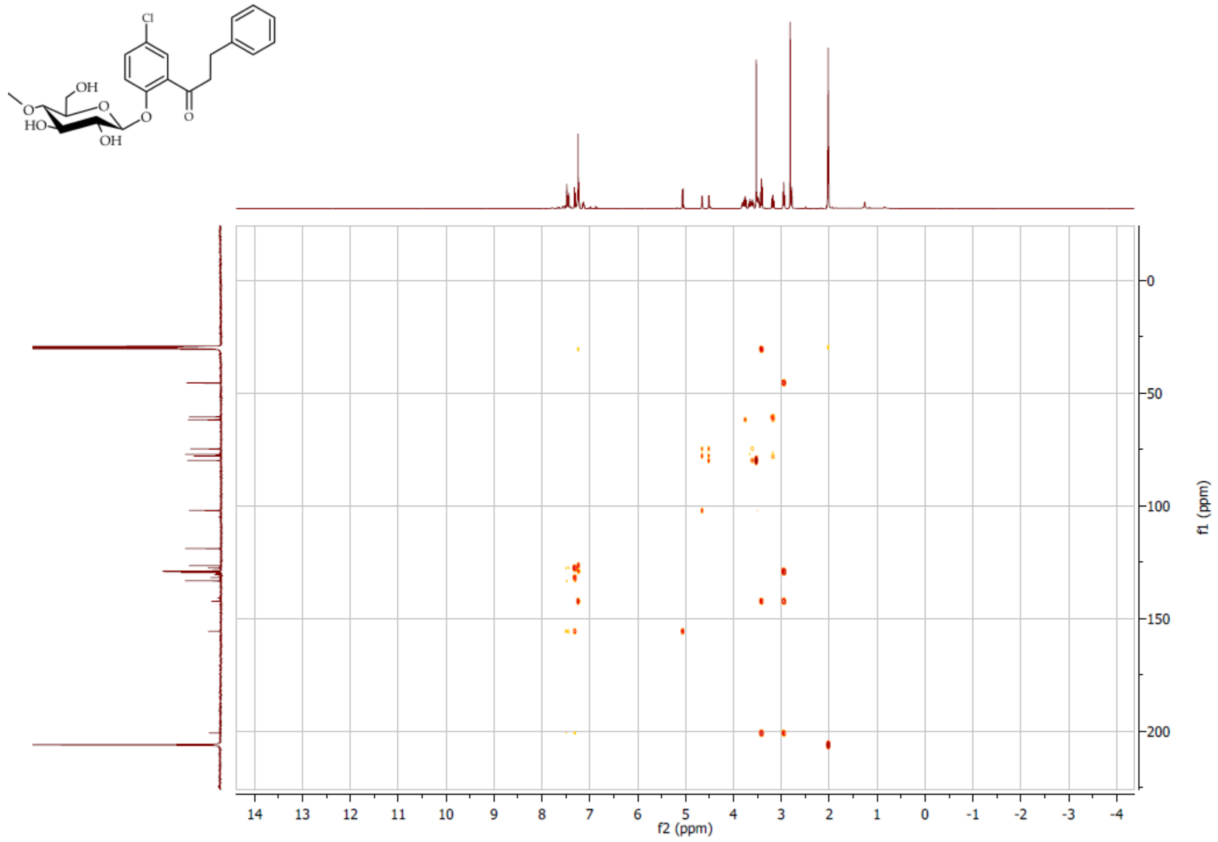

Figure S108. HMBC contour map – ^{1}H x ^{13}C of 5′-chlorodihydrochalcone 2′-*O*-*β*-D-(4′′-*O*-methyl)-glucopyranoside (**6a**)

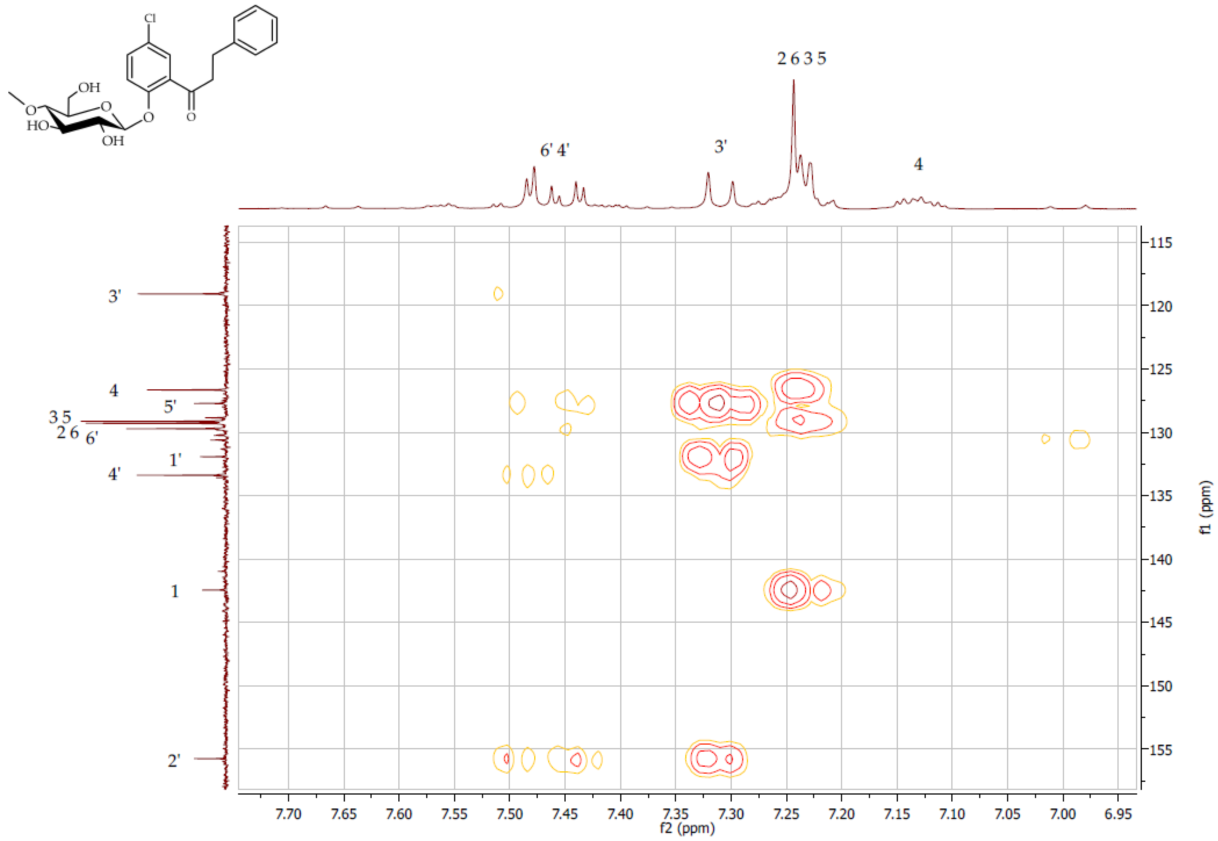

Figure S109. HMBC contour map – ^{1}H x ^{13}C expansion of 5′-chlorodihydrochalcone 2′-*O*-*β*-D-(4′′-*O*-methyl)-glucopyranoside (**6a**)

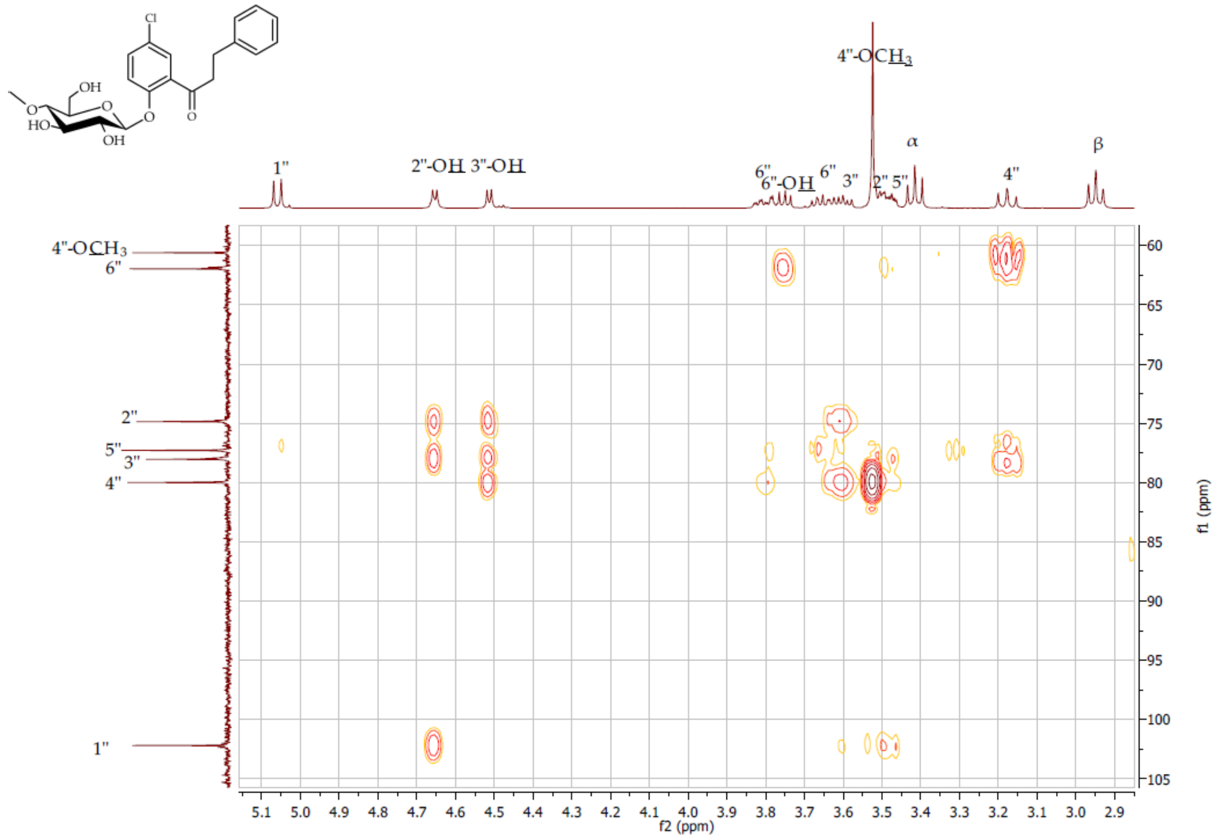

Figure S110. HMBC contour map – ^{1}H x ^{13}C expansion of 5′-chlorodihydrochalcone 2′-*O*-*β*-D-(4′′-*O*-methyl)-glucopyranoside (**6a**)

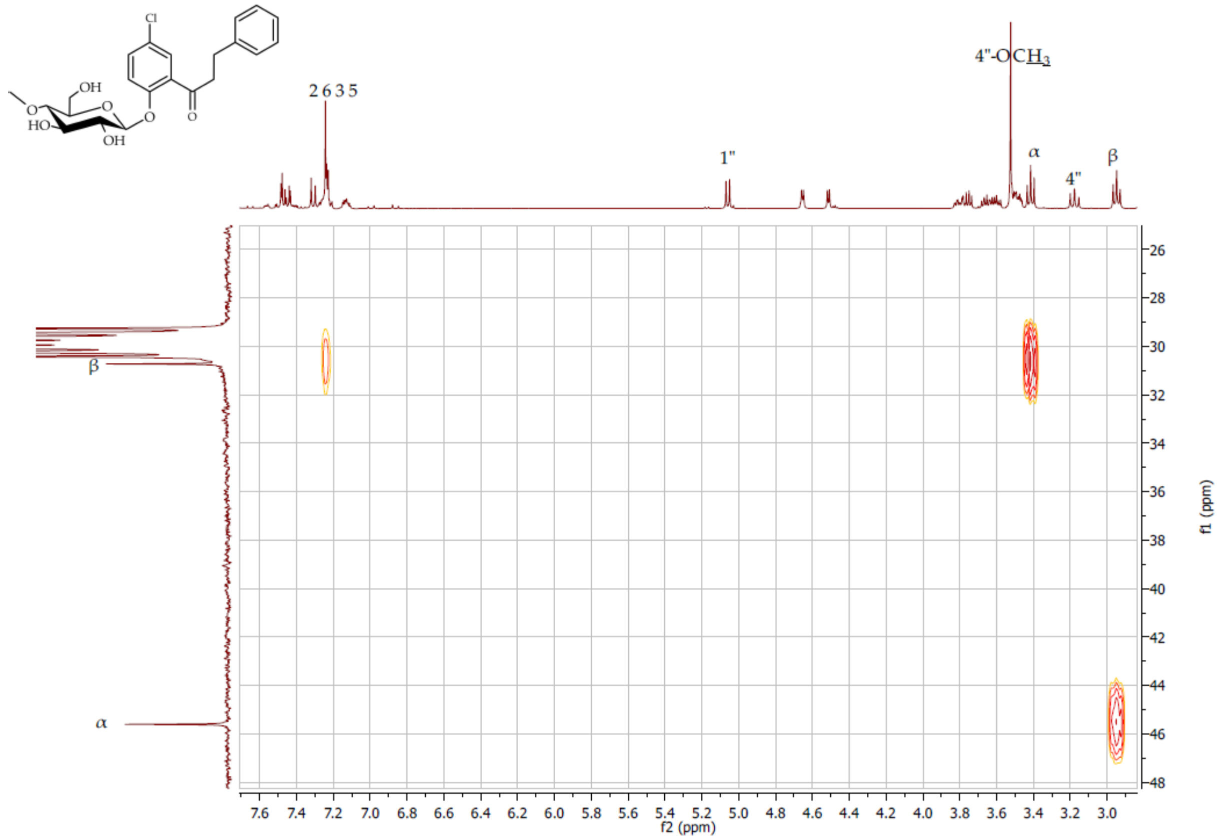

Figure S111. HMBC contour map – ^{1}H x ^{13}C expansion of 5′-chlorodihydrochalcone 2′-*O*-*β*-D-(4′′-*O*-methyl)-glucopyranoside (**6a**)

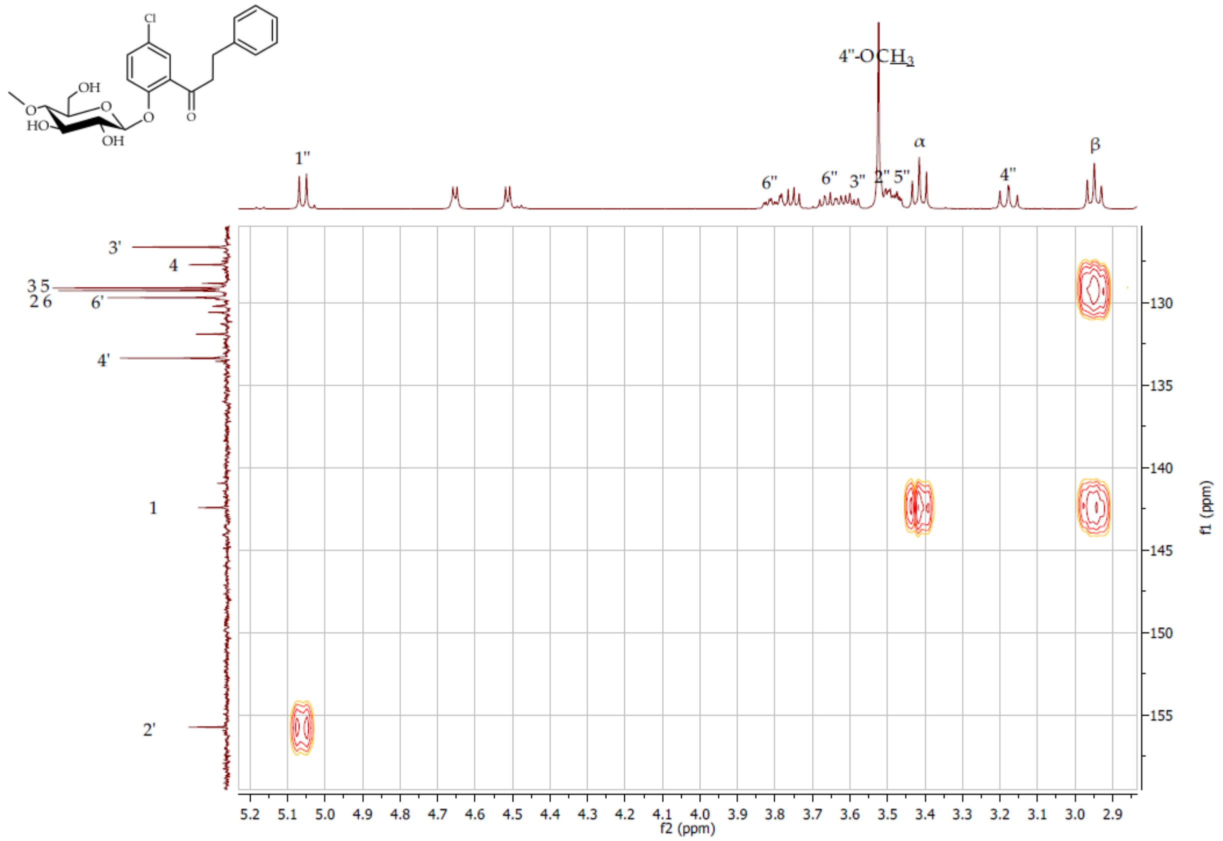

Figure S112. HMBC contour map – ^{1}H x ^{13}C expansion of 5′-chlorodihydrochalcone 2′-*O*-*β*-D-(4′′-*O*-methyl)-glucopyranoside (**6a**)

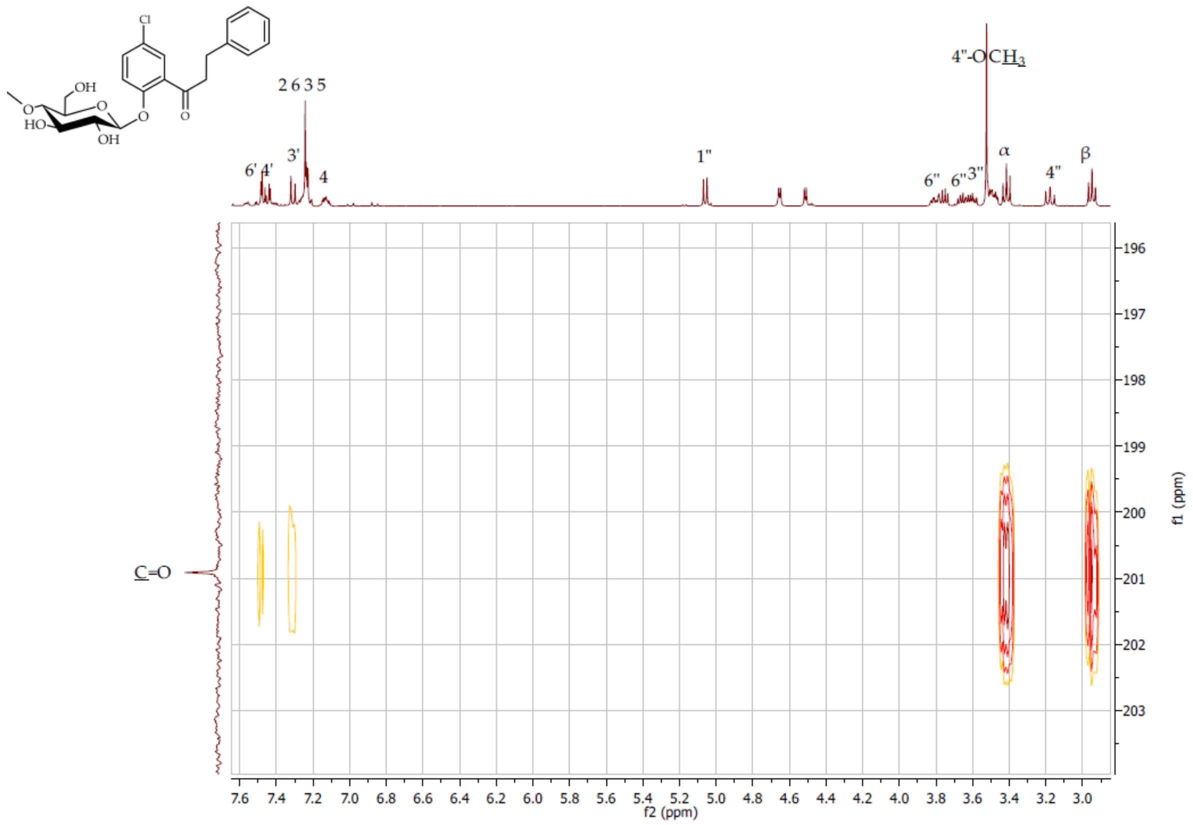

Figure S113. HMBC contour map – ^{1}H x ^{13}C expansion of 5′-chlorodihydrochalcone 2′-*O*-*β*-D-(4′′-*O*-methyl)-glucopyranoside (**6a**)

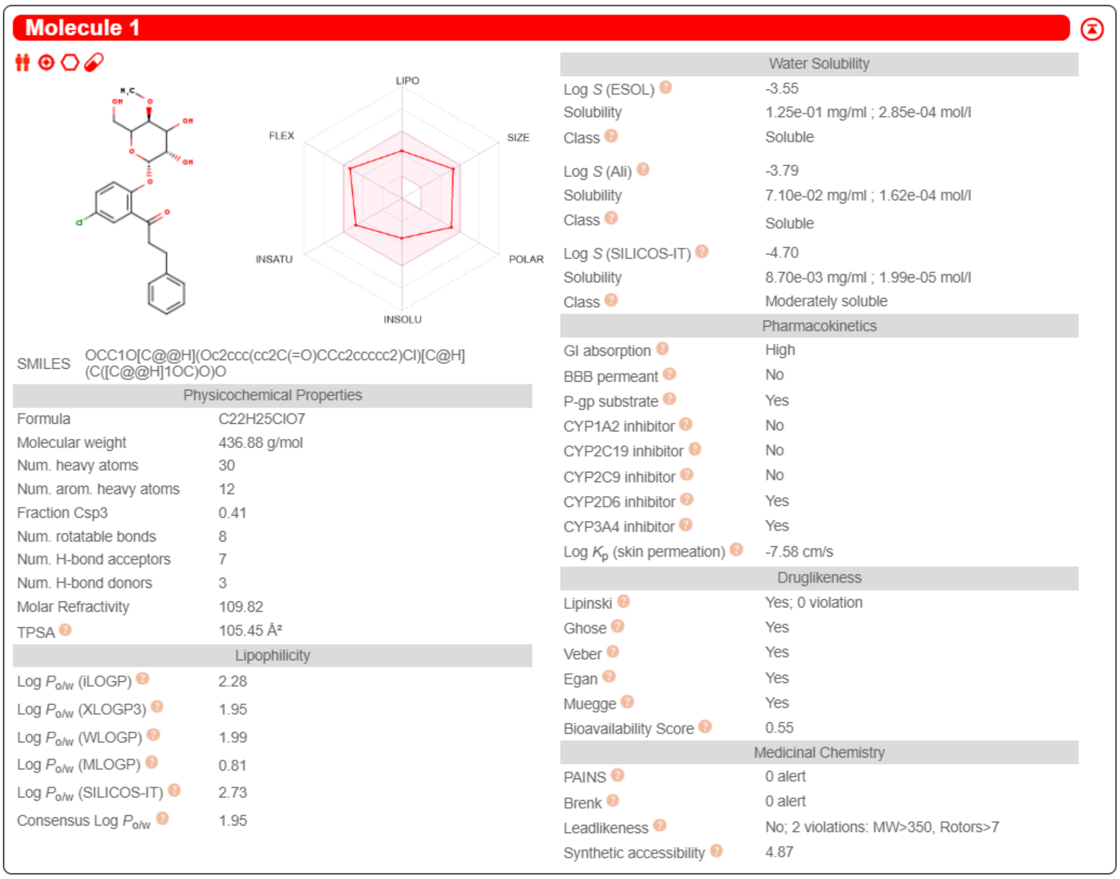

Molecule 1

SMILES OCC1O[C@@H](Oc2ccc(cc2C(=O)CCc2ccccc2)Cl)[C@H](C([C@@H]1OC)O)O

Physicochemical Properties	
Formula	C22H25ClO7
Molecular weight	436.88 g/mol
Num. heavy atoms	30
Num. arom. heavy atoms	12
Fraction Csp3	0.41
Num. rotatable bonds	8
Num. H-bond acceptors	7
Num. H-bond donors	3
Molar Refractivity	109.82
TPSA	105.45 Å²

Lipophilicity	
Log $P_{o/w}$ (iLOGP)	2.28
Log $P_{o/w}$ (XLOGP3)	1.95
Log $P_{o/w}$ (WLOGP)	1.99
Log $P_{o/w}$ (MLOGP)	0.81
Log $P_{o/w}$ (SILICOS-IT)	2.73
Consensus Log $P_{o/w}$	1.95

Water Solubility	
Log *S* (ESOL)	-3.55
Solubility	1.25e-01 mg/ml ; 2.85e-04 mol/l
Class	Soluble
Log *S* (Ali)	-3.79
Solubility	7.10e-02 mg/ml ; 1.62e-04 mol/l
Class	Soluble
Log *S* (SILICOS-IT)	-4.70
Solubility	8.70e-03 mg/ml ; 1.99e-05 mol/l
Class	Moderately soluble

Pharmacokinetics	
GI absorption	High
BBB permeant	No
P-gp substrate	Yes
CYP1A2 inhibitor	No
CYP2C19 inhibitor	No
CYP2C9 inhibitor	No
CYP2D6 inhibitor	Yes
CYP3A4 inhibitor	Yes
Log K_p (skin permeation)	-7.58 cm/s

Druglikeness	
Lipinski	Yes; 0 violation
Ghose	Yes
Veber	Yes
Egan	Yes
Muegge	Yes
Bioavailability Score	0.55

Medicinal Chemistry	
PAINS	0 alert
Brenk	0 alert
Leadlikeness	No; 2 violations: MW>350, Rotors>7
Synthetic accessibility	4.87

Figure S114. 5′-Chlorodihydrochalcone 2′-*O*-*β*-D-(4′′-*O*-methyl)-glucopyranoside (**6a**) physicochemical and ADME parameters prediction using the SwissADME modelling

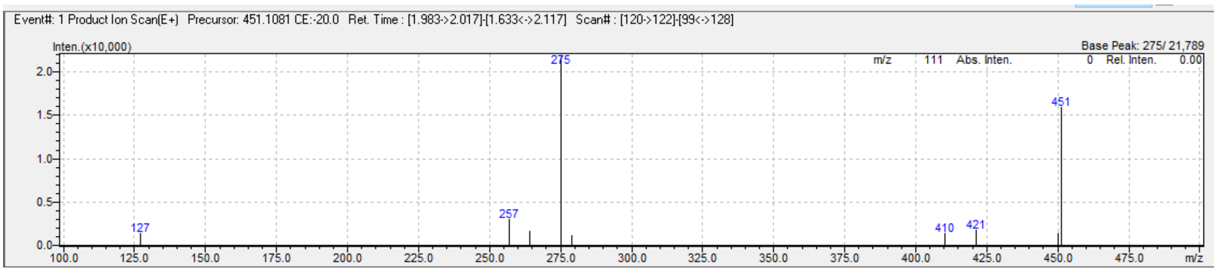

Figure S115. MS analysis of 5′-chloro-2′-hydroxychalcone 3-*O*-*β*-D-(4′′-*O*-methyl)-glucopyranoside (**6b**)

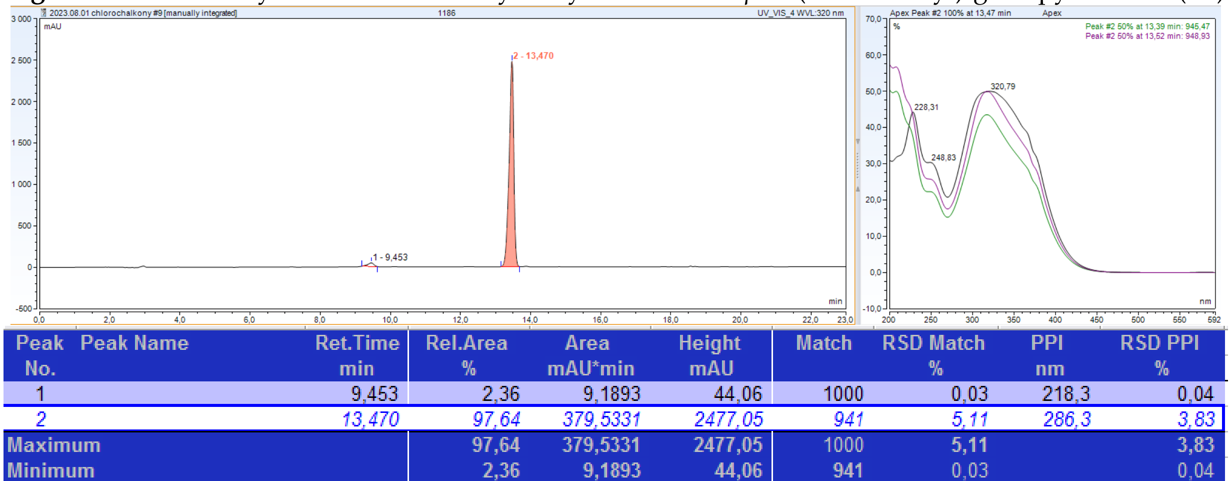

Peak No.	Peak Name	Ret.Time min	Rel.Area %	Area mAU*min	Height mAU	Match	RSD Match %	PPI nm	RSD PPI %
1		9,453	2,36	9,1893	44,06	1000	0,03	218,3	0,04
2		13,470	97,64	379,5331	2477,05	941	5,11	286,3	3,83
Maximum			97,64	379,5331	2477,05	1000	5,11		3,83
Minimum			2,36	9,1893	44,06	941	0,03		0,04

Figure S116. HPLC analysis of 5′-chloro-2′-hydroxychalcone 3-*O*-*β*-D-(4′′-*O*-methyl)-glucopyranoside (**6b**)

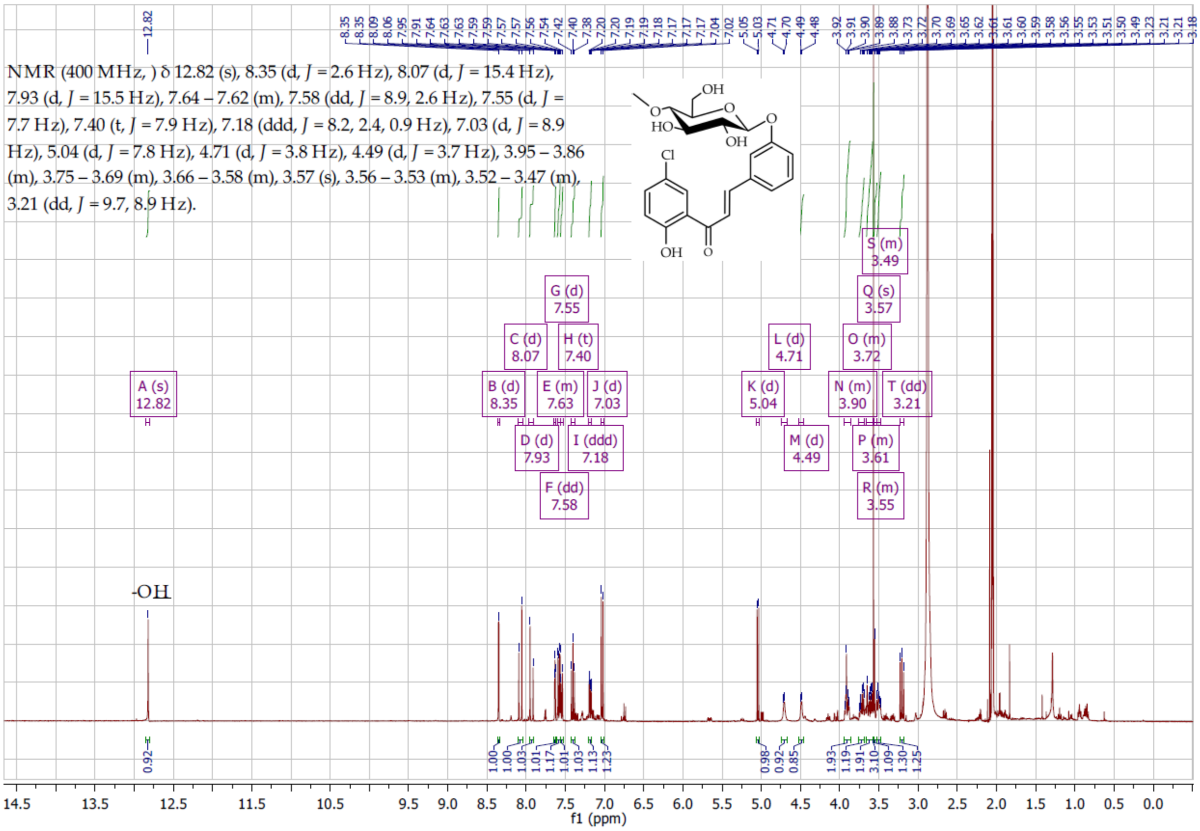

Figure S117. ^{1}H NMR spectrum (*δ*, acetone-d6, 600 MHz) of 5′-chloro-2′-hydroxychalcone 3-*O*-*β*-D-(4′′-*O*-methyl)-glucopyranoside (**6b**)

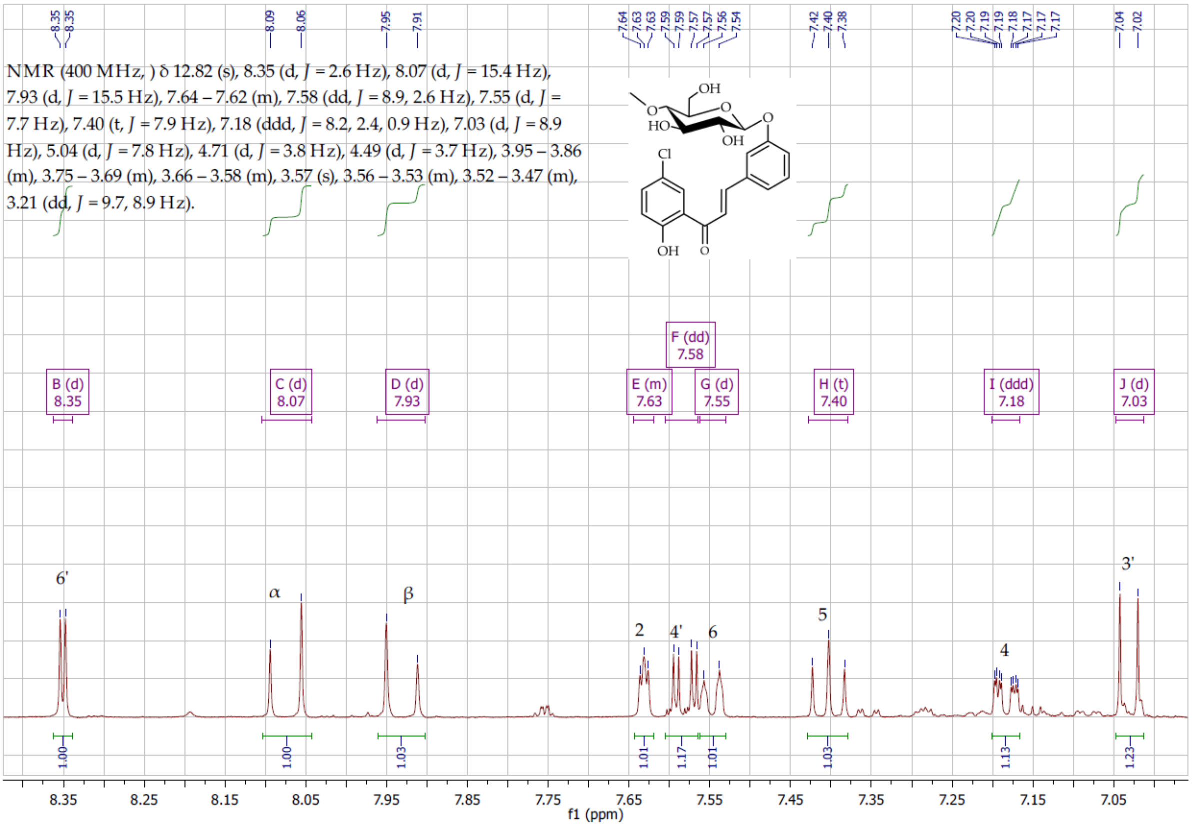

Figure S118. ^{1}H NMR spectrum expansion (*δ*, acetone-d6, 600 MHz) of 5′-chloro-2′-hydroxychalcone 3-*O*-*β*-D-(4′′-*O*-methyl)-glucopyranoside (**6b**)

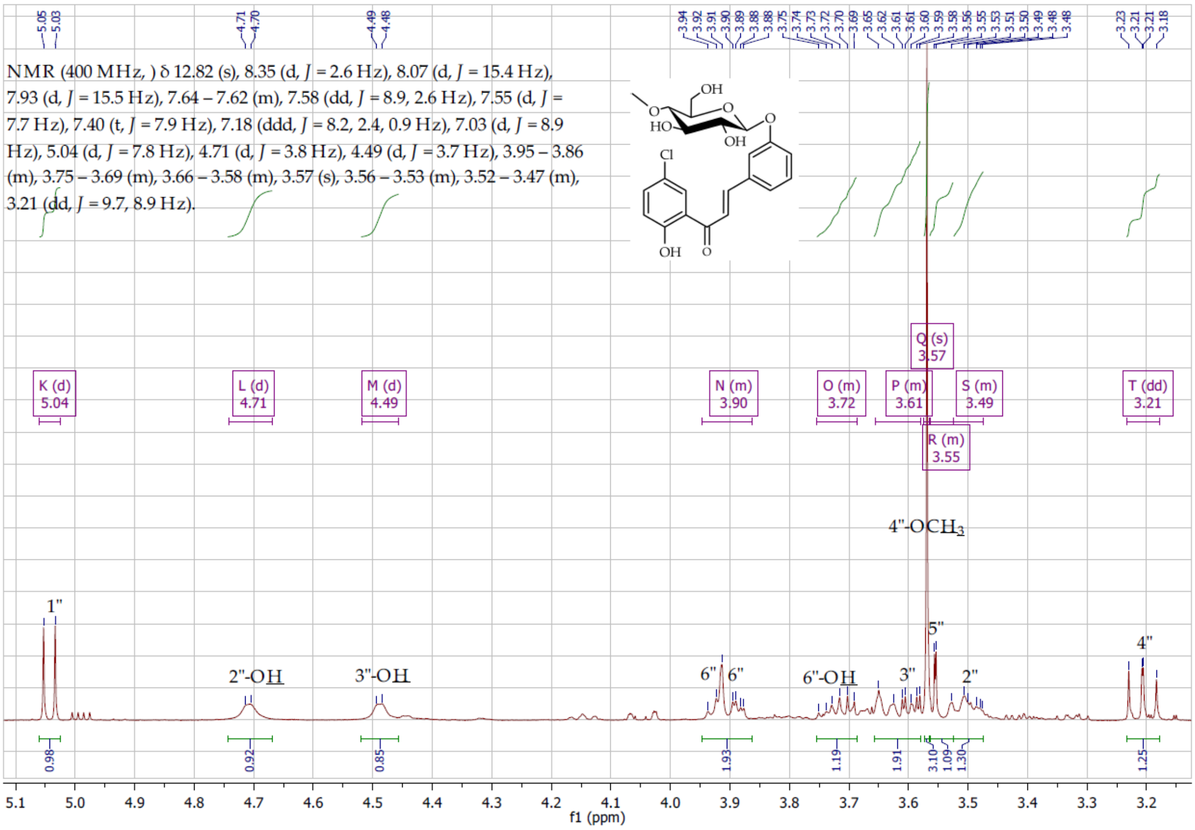

Figure S119. ^{1}H NMR spectrum expansion (*δ*, acetone-d6, 600 MHz) of 5′-chloro-2′-hydroxychalcone 3-*O*-*β*-D-(4′′-*O*-methyl)-glucopyranoside (**6b**)

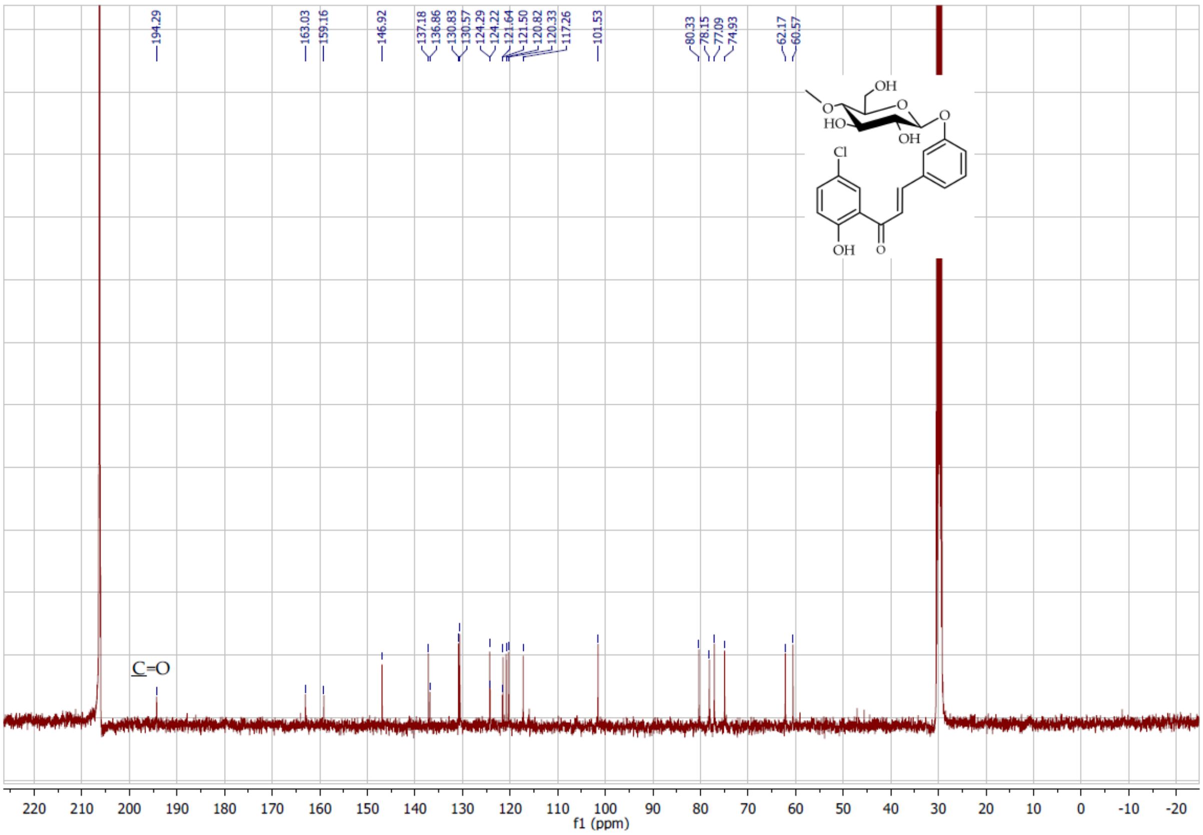

Figure S120. ^{13}C NMR spectrum (*δ*, acetone-d6, 151 MHz) of 5′-chloro-2′-hydroxychalcone 3-*O*-*β*-D-(4′′-*O*-methyl)-glucopyranoside (**6b**)

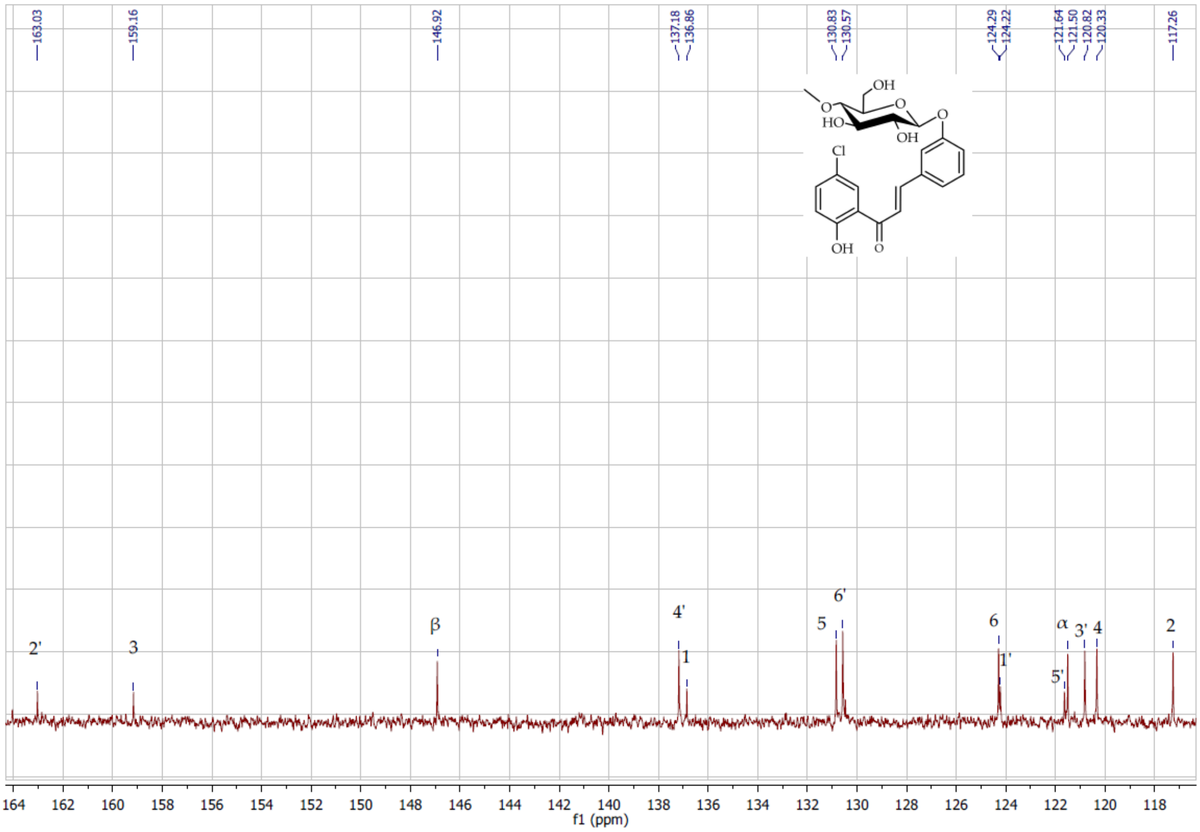

Figure S121. ^{13}C NMR spectrum expansion (*δ*, acetone-d6, 151 MHz) of 5′-chloro-2′-hydroxychalcone 3-*O*-*β*-D-(4′′-*O*-methyl)-glucopyranoside (**6b**)

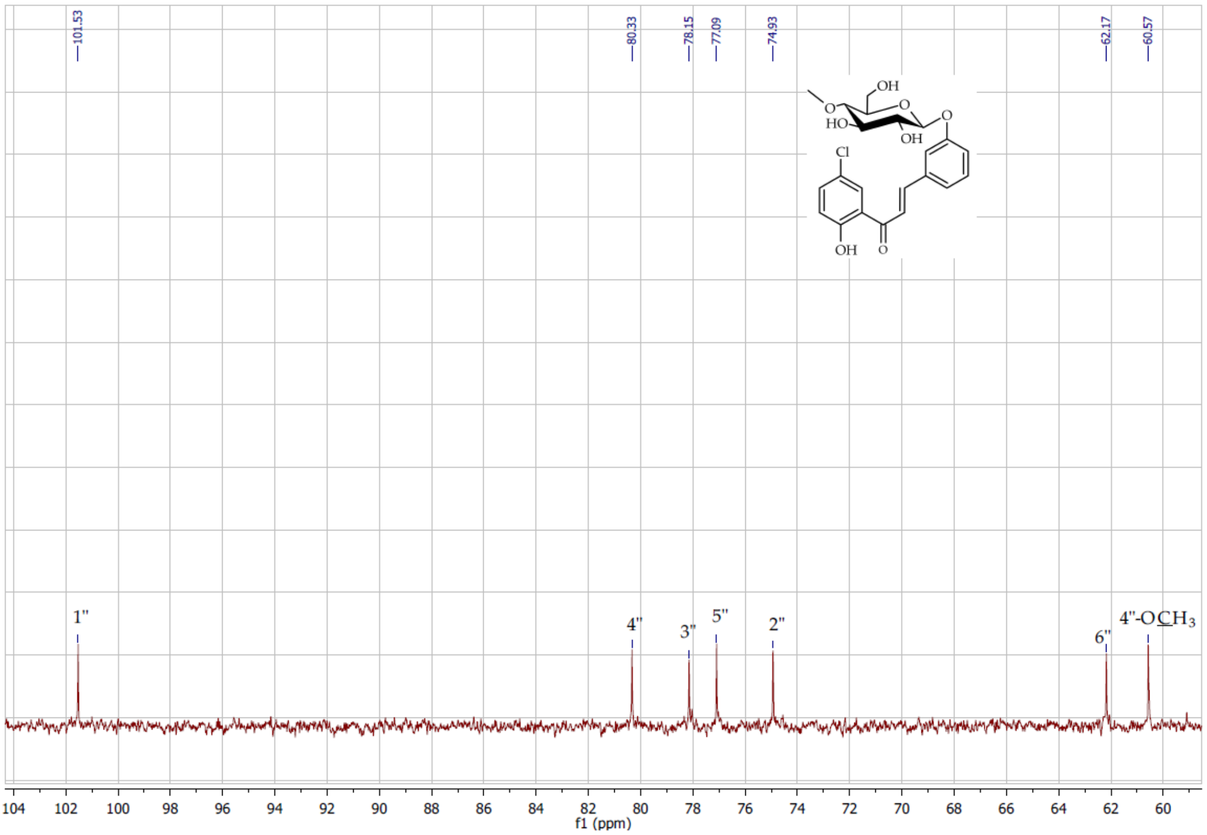

Figure S122. ^{13}C NMR spectrum expansion (*δ*, acetone-d6, 151 MHz) of 5′-chloro-2′-hydroxychalcone 3-*O*-*β*-D-(4′′-*O*-methyl)-glucopyranoside (**6b**)

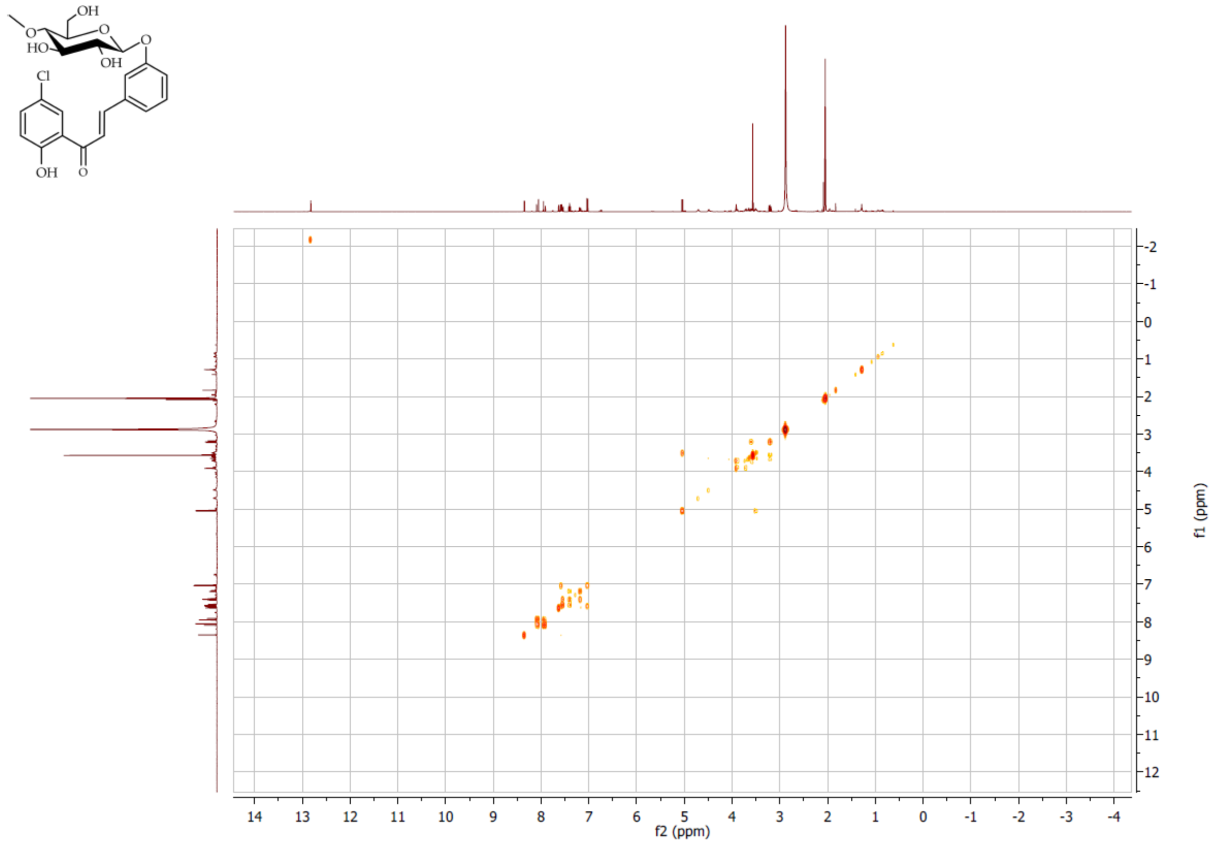

Figure S123. COSY contour map – ^{1}H x ^{1}H of 4-chloro-2′-hydroxydihydrochalcone 5′-chloro-2′-hydroxychalcone 3-*O*-*β*-D-(4′′-*O*-methyl)-glucopyranoside (**6b**)

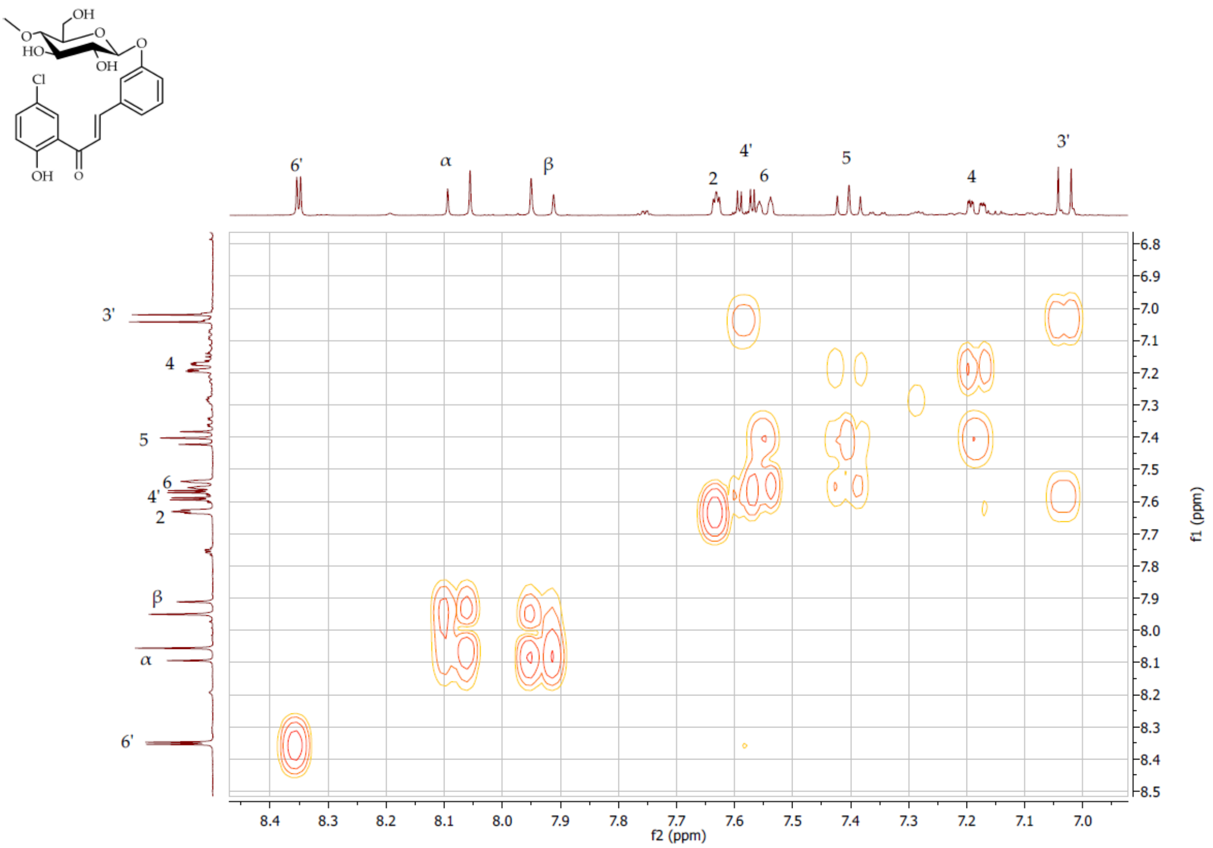

Figure S124. COSY contour map – ^{1}H x ^{1}H expansion of 5′-chloro-2′-hydroxychalcone 3-*O*-*β*-D-(4′′-*O*-methyl)-glucopyranoside (**6b**)

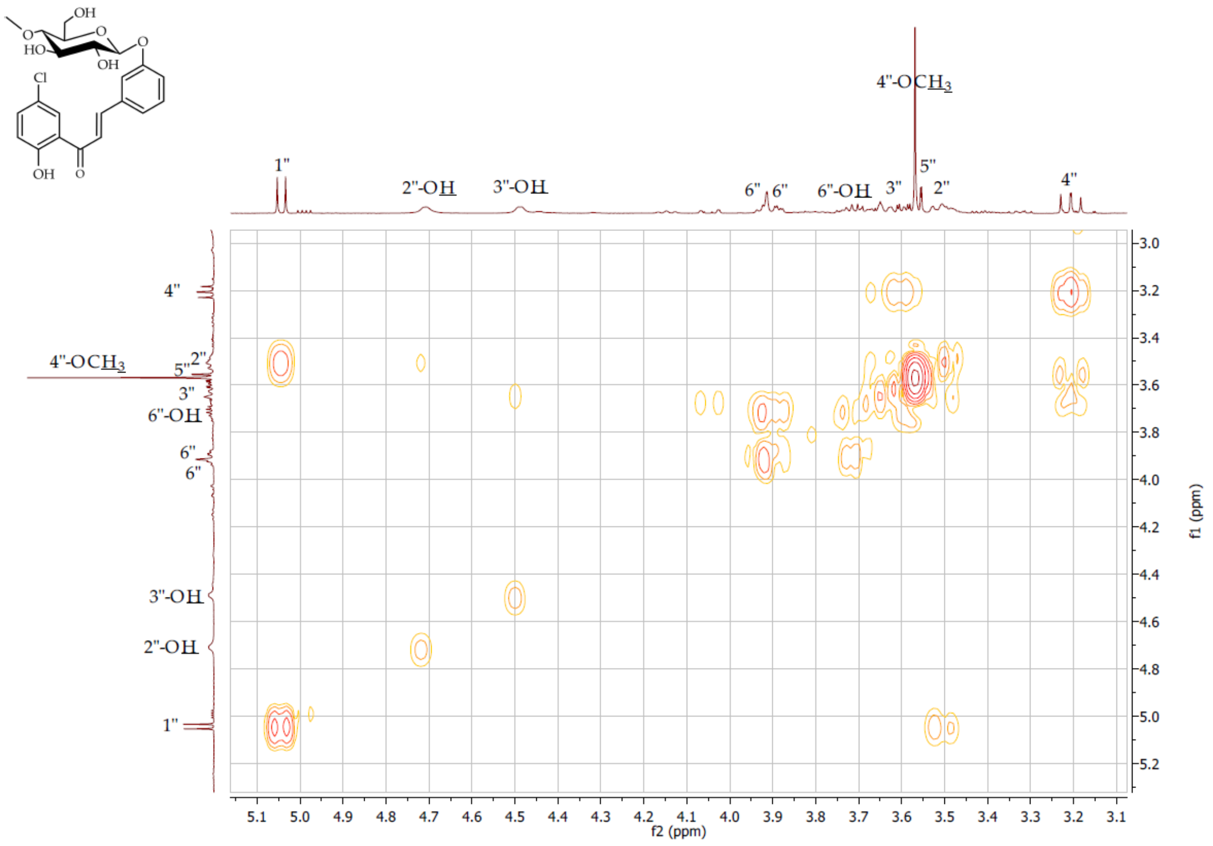

Figure S125. COSY contour map – ^{1}H x ^{1}H expansion of 5′-chloro-2′-hydroxychalcone 3-*O*-*β*-D-(4′′-*O*-methyl)-glucopyranoside (**6b**)

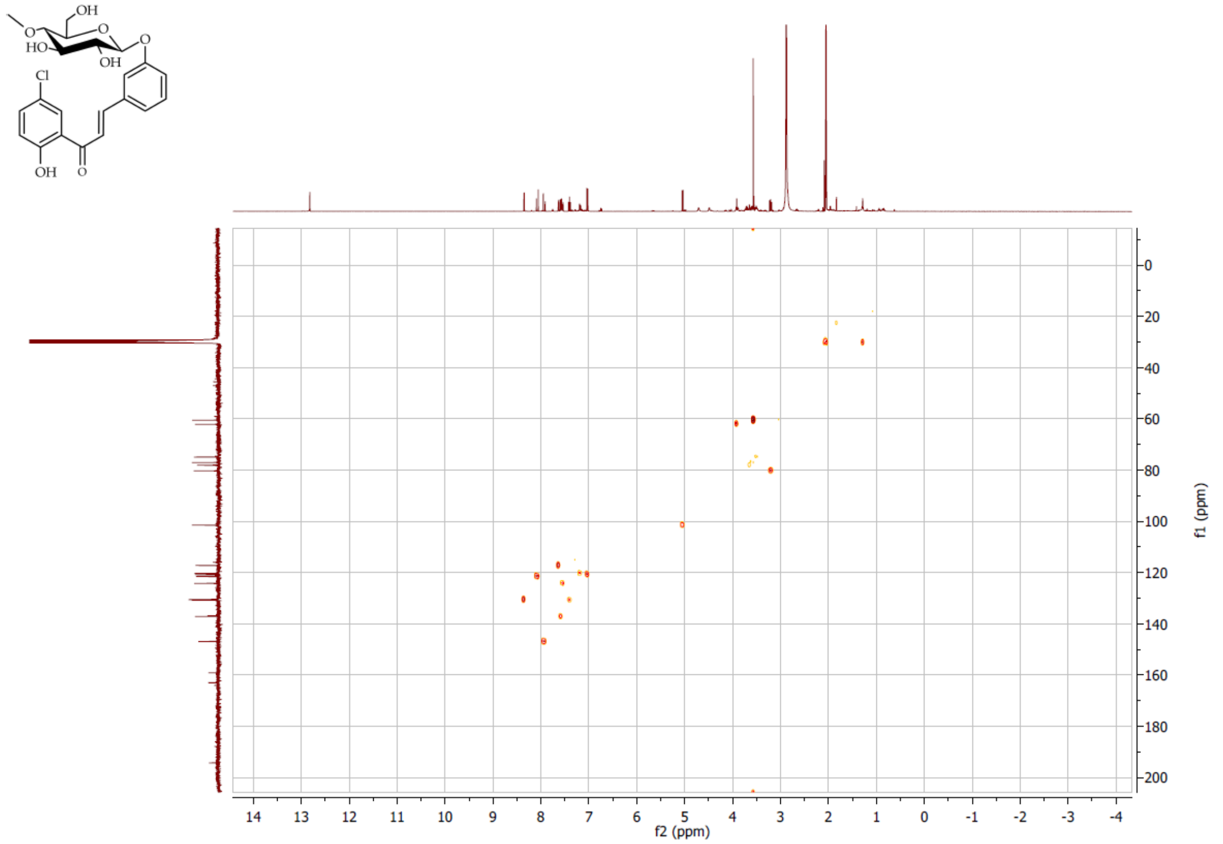

Figure S126. HMQC contour map – ^{1}H x ^{13}C of 5′-chloro-2′-hydroxychalcone 3-*O*-*β*-D-(4′′-*O*-methyl)-glucopyranoside (**6b**)

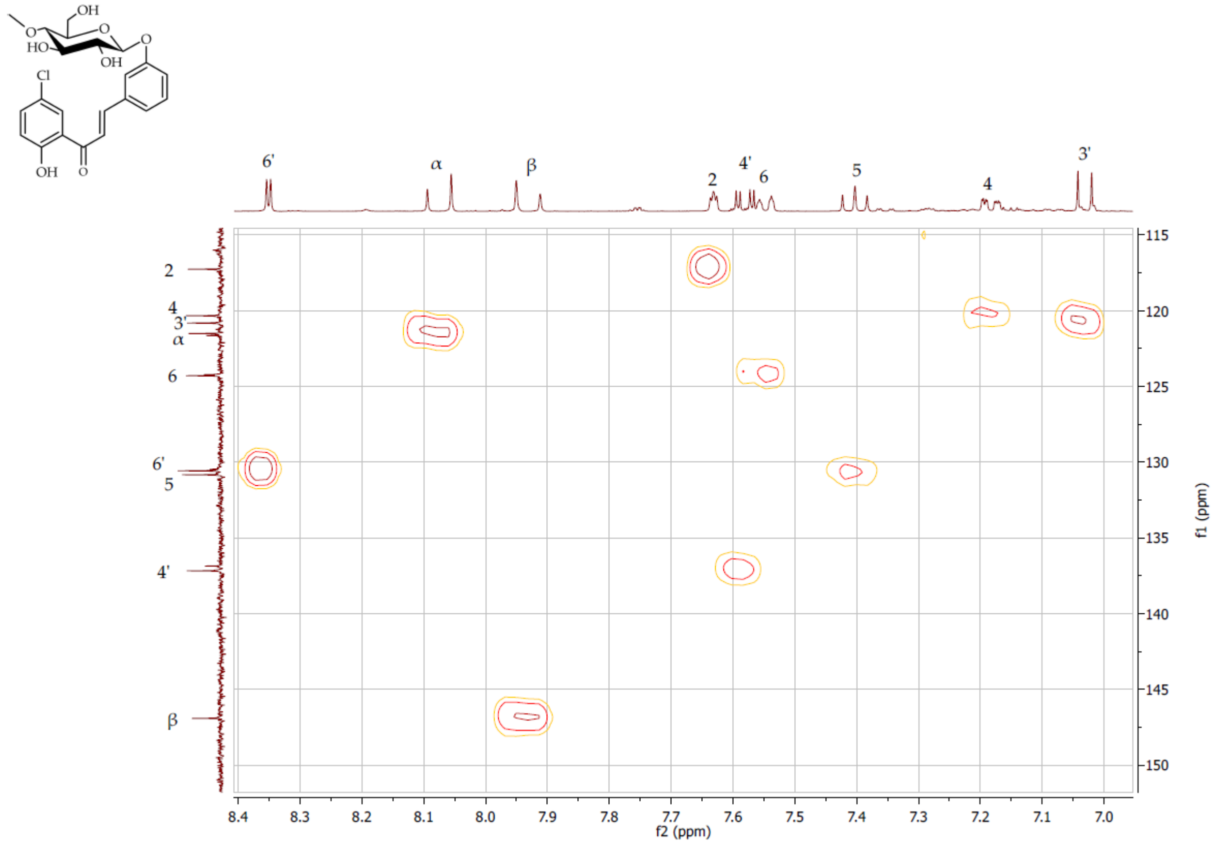

Figure S127. HMQC contour map – ^{1}H x ^{13}C expansion of 5′-chloro-2′-hydroxychalcone 3-*O*-*β*-D-(4′′-*O*-methyl)-glucopyranoside (**6b**)

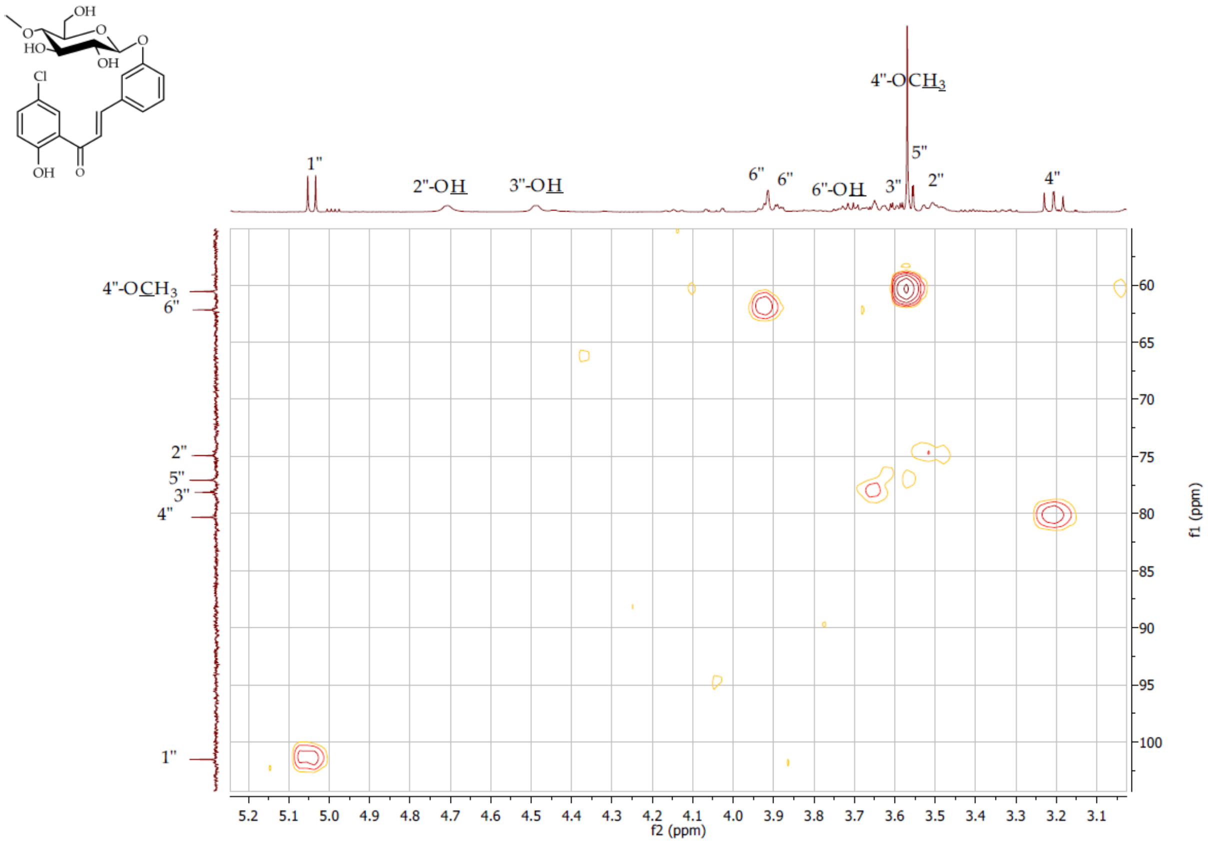

Figure S128. HMQC contour map – [1]H x ^{13}C expansion of 5′-chloro-2′-hydroxychalcone 3-*O*-*β*-D-(4′′-*O*-methyl)-glucopyranoside (**6b**)

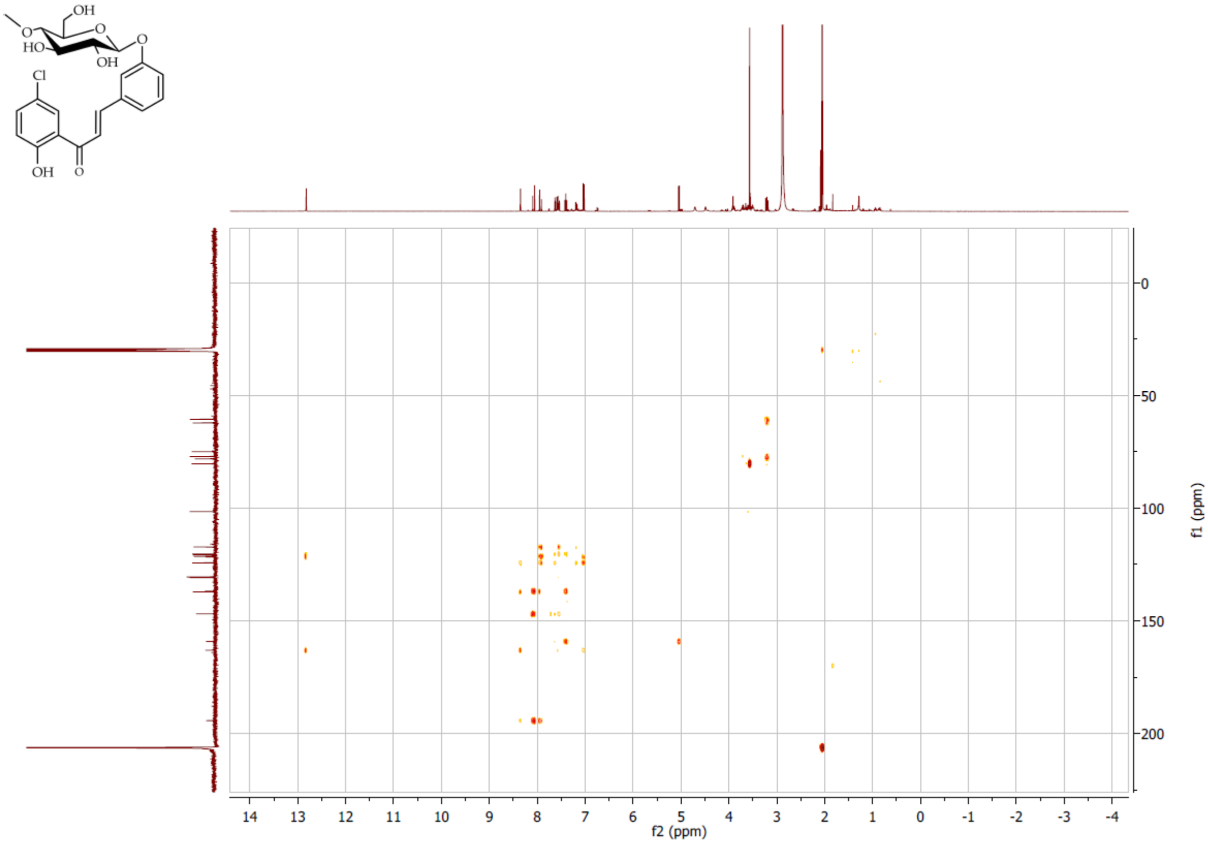

Figure S129. HMBC contour map – ^{1}H x ^{13}C of 5′-chloro-2′-hydroxychalcone 3-*O*-*β*-D-(4′′-*O*-methyl)-glucopyranoside (**6b**)

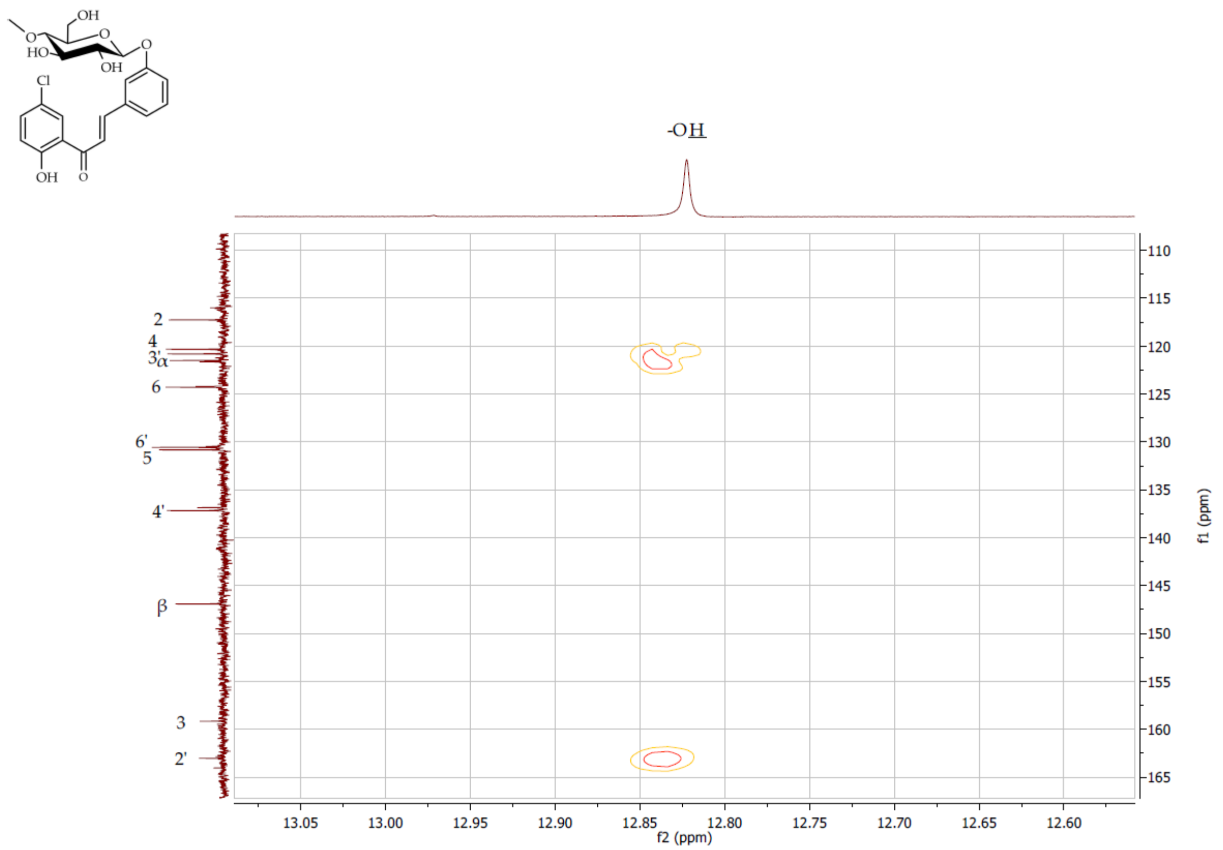

Figure S130. HMBC contour map – ^{1}H x ^{13}C expansion of 5′-chloro-2′-hydroxychalcone 3-*O*-*β*-D-(4′′-*O*-methyl)-glucopyranoside (**6b**)

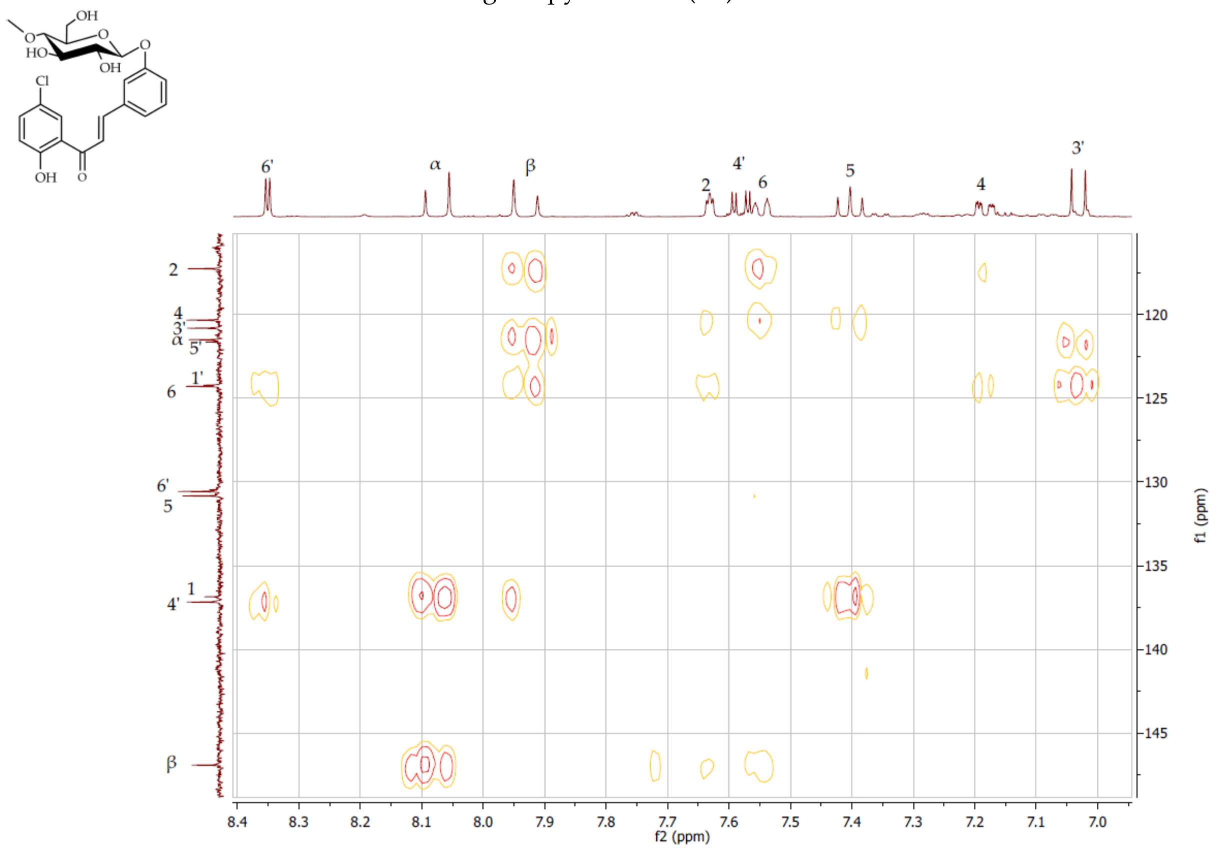

Figure S131. HMBC contour map – ^{1}H x ^{13}C expansion of 5′-chloro-2′-hydroxychalcone 3-*O*-*β*-D-(4′′-*O*-methyl)-glucopyranoside (**6b**)

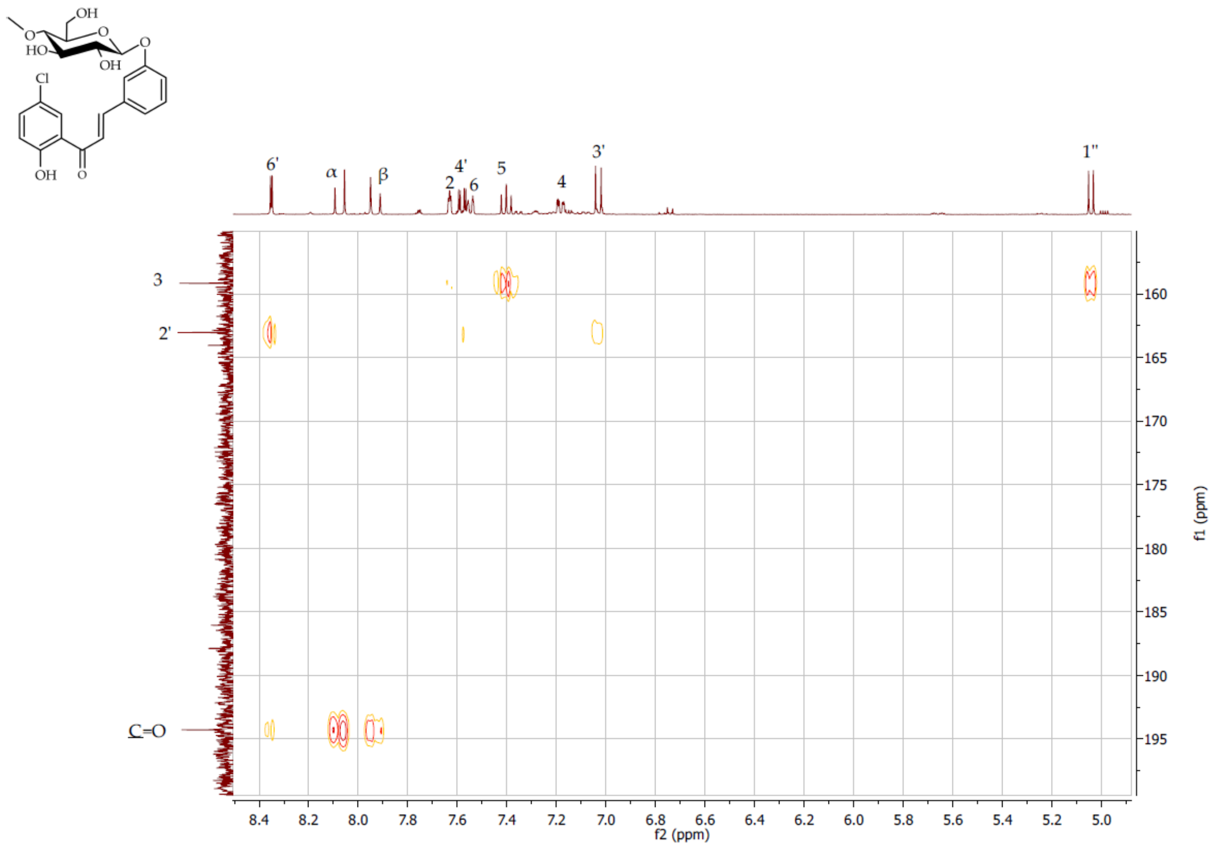

Figure S132. HMBC contour map – ^{1}H x ^{13}C expansion of 5′-chloro-2′-hydroxychalcone 3-*O*-*β*-D-(4′′-*O*-methyl)-glucopyranoside (**6b**)

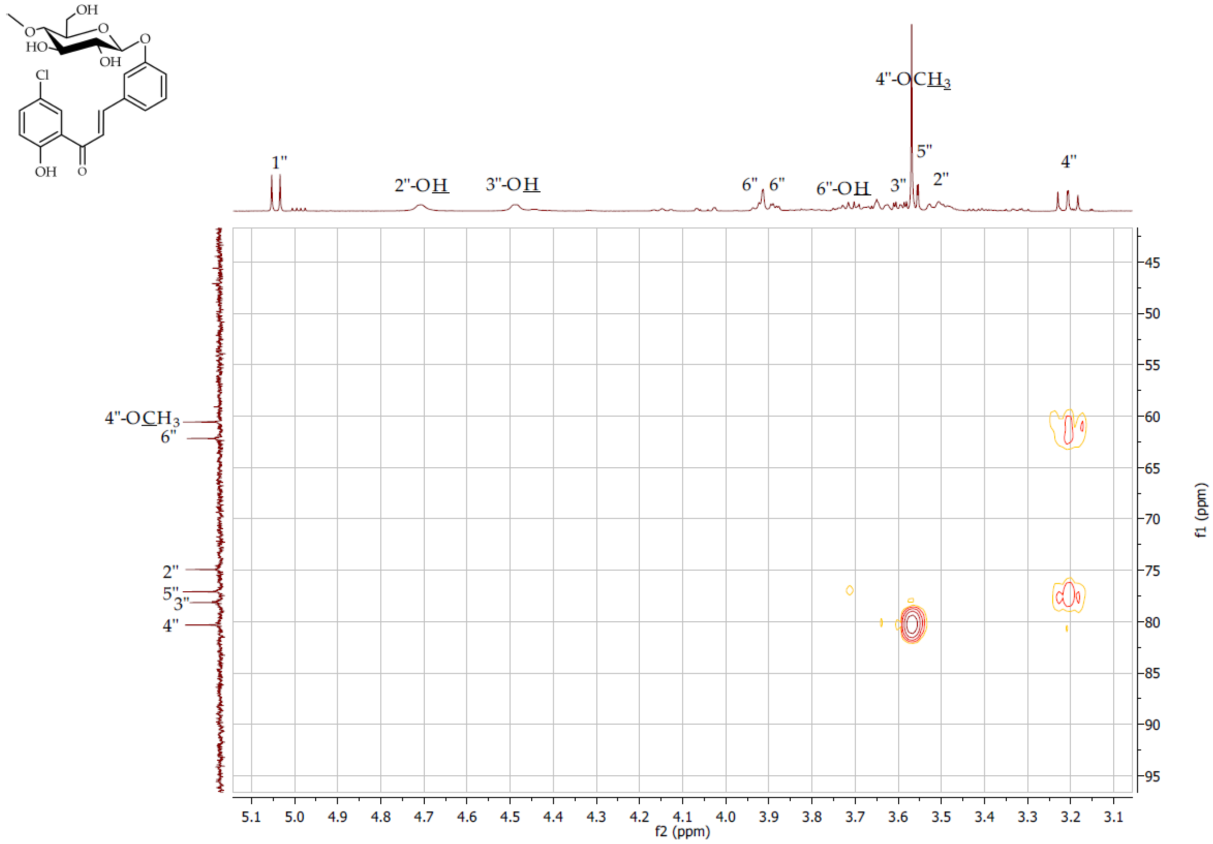

Figure S133. HMBC contour map – ^{1}H x ^{13}C expansion of 5′-chloro-2′-hydroxychalcone 3-*O*-*β*-D-(4′′-*O*-methyl)-glucopyranoside (**6b**)

Molecule 1

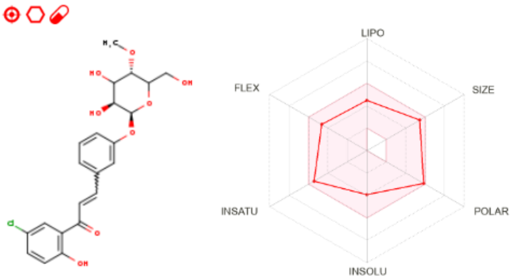

SMILES CO[C@@H]1C(CO)O[C@H]([C@H](C1O)O)Oc1cccc(c1)/C=C/C(=O)c1cc(Cl)ccc1O

Physicochemical Properties	
Formula	C22H23ClO8
Molecular weight	450.87 g/mol
Num. heavy atoms	31
Num. arom. heavy atoms	12
Fraction Csp3	0.32
Num. rotatable bonds	7
Num. H-bond acceptors	8
Num. H-bond donors	4
Molar Refractivity	112.16
TPSA	125.68 Å²

Lipophilicity	
Log $P_{o/w}$ (iLOGP)	2.59
Log $P_{o/w}$ (XLOGP3)	2.34
Log $P_{o/w}$ (WLOGP)	1.67
Log $P_{o/w}$ (MLOGP)	0.22
Log $P_{o/w}$ (SILICOS-IT)	2.09
Consensus Log $P_{o/w}$	1.78

Water Solubility	
Log *S* (ESOL)	-3.93
Solubility	5.25e-02 mg/ml ; 1.16e-04 mol/l
Class	Soluble
Log *S* (Ali)	-4.62
Solubility	1.08e-02 mg/ml ; 2.41e-05 mol/l
Class	Moderately soluble
Log *S* (SILICOS-IT)	-3.39
Solubility	1.82e-01 mg/ml ; 4.05e-04 mol/l
Class	Soluble

Pharmacokinetics	
GI absorption	High
BBB permeant	No
P-gp substrate	Yes
CYP1A2 inhibitor	No
CYP2C19 inhibitor	No
CYP2C9 inhibitor	No
CYP2D6 inhibitor	No
CYP3A4 inhibitor	Yes
Log K_p (skin permeation)	-7.39 cm/s

Druglikeness	
Lipinski	Yes; 0 violation
Ghose	Yes
Veber	Yes
Egan	Yes
Muegge	Yes
Bioavailability Score	0.55

Medicinal Chemistry	
PAINS	0 alert
Brenk	1 alert: michael_acceptor_1
Leadlikeness	No; 1 violation: MW>350
Synthetic accessibility	4.96

Figure S134. 5′-Chloro-2′-hydroxychalcone 3-*O*-*β*-D-(4′′-*O*-methyl)-glucopyranoside (**6b**) physicochemical and ADME parameters prediction using the SwissADME modelling

Molecule 1

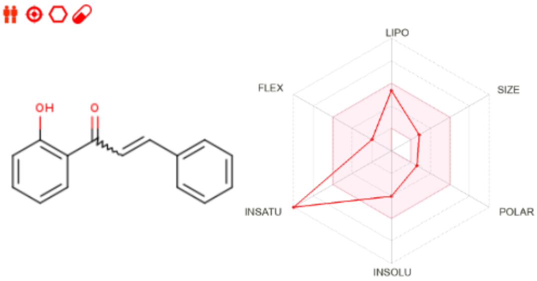

SMILES O=C(c1ccccc1O)/C=C/c1ccccc1

Physicochemical Properties	
Formula	C15H12O2
Molecular weight	224.25 g/mol
Num. heavy atoms	17
Num. arom. heavy atoms	12
Fraction Csp3	0.00
Num. rotatable bonds	3
Num. H-bond acceptors	2
Num. H-bond donors	1
Molar Refractivity	68.27
TPSA	37.30 Å²

Lipophilicity	
Log $P_{o/w}$ (iLOGP)	2.34
Log $P_{o/w}$ (XLOGP3)	3.89
Log $P_{o/w}$ (WLOGP)	3.18
Log $P_{o/w}$ (MLOGP)	2.78
Log $P_{o/w}$ (SILICOS-IT)	3.45
Consensus Log $P_{o/w}$	3.13

Water Solubility	
Log *S* (ESOL)	-4.01
Solubility	2.21e-02 mg/ml ; 9.88e-05 mol/l
Class	Moderately soluble
Log *S* (Ali)	-4.37
Solubility	9.54e-03 mg/ml ; 4.25e-05 mol/l
Class	Moderately soluble
Log *S* (SILICOS-IT)	-4.38
Solubility	9.24e-03 mg/ml ; 4.12e-05 mol/l
Class	Moderately soluble

Pharmacokinetics	
GI absorption	High
BBB permeant	Yes
P-gp substrate	No
CYP1A2 inhibitor	No
CYP2C19 inhibitor	Yes
CYP2C9 inhibitor	Yes
CYP2D6 inhibitor	No
CYP3A4 inhibitor	No
Log K_p (skin permeation)	-4.91 cm/s

Druglikeness	
Lipinski	Yes; 0 violation
Ghose	Yes
Veber	Yes
Egan	Yes
Muegge	Yes
Bioavailability Score	0.55

Medicinal Chemistry	
PAINS	0 alert
Brenk	1 alert: michael_acceptor_1
Leadlikeness	No; 2 violations: MW<250, XLOGP3>3.5
Synthetic accessibility	2.40

Figure S135. 2′-Hydroxychalcone (**7**) physicochemical and ADME parameters prediction using the SwissADME modelling
